# Shared pattern of impaired social communication and cognitive ability in the youth brain across diagnostic boundaries

**DOI:** 10.1016/j.dcn.2023.101219

**Published:** 2023-02-18

**Authors:** Irene Voldsbekk, Rikka Kjelkenes, Thomas Wolfers, Andreas Dahl, Martina J. Lund, Tobias Kaufmann, Sara Fernandez-Cabello, Ann-Marie G. de Lange, Christian K. Tamnes, Ole A. Andreassen, Lars T. Westlye, Dag Alnæs

**Affiliations:** a Norwegian Centre for Mental Disorders Research (NORMENT), Institute of Clinical Medicine, University of Oslo, & Division of Mental Health and Addiction, Oslo University Hospital, Oslo, Norway; b Department of Psychology, University of Oslo, Oslo, Norway; c Department of Psychiatry and Psychotherapy, University of Tübingen, Germany; d LREN, Centre for Research in Neurosciences, Department of Clinical Neurosciences, CHUV and University of Lausanne, Lausanne, Switzerland; e Department of Psychiatry, University of Oxford, Oxford, UK; f Department of Psychiatric Research, Diakonhjemmet Hospital, Oslo, Norway; g PROMENTA Research Center, Department of Psychology, University of Oslo, Oslo, Norway; h KG Jebsen Centre for Neurodevelopmental Disorders, University of Oslo, Oslo, Norway; i Kristiania University College, Oslo, Norway

**Keywords:** Psychopathology, Brain, Behavior, Risk, Multivariate, Youth

## Abstract

**Background:**

Abnormalities in brain structure are shared across diagnostic categories. Given the high rate of comorbidity, the interplay of relevant behavioural factors may also cross these classic boundaries.

**Methods:**

We aimed to detect brain-based dimensions of behavioural factors using canonical correlation and independent component analysis in a clinical youth sample (n = 1732, 64 % male, age: 5–21 years).

**Results:**

We identified two correlated patterns of brain structure and behavioural factors. The first mode reflected physical and cognitive maturation (*r* = 0.92, *p* = .005). The second mode reflected lower cognitive ability, poorer social skills, and psychological difficulties (*r* = 0.92, *p* = .006). Elevated scores on the second mode were a common feature across all diagnostic boundaries and linked to the number of comorbid diagnoses independently of age. Critically, this brain pattern predicted normative cognitive deviations in an independent population-based sample (n = 1253, 54 % female, age: 8–21 years), supporting the generalisability and external validity of the reported brain-behaviour relationships.

**Conclusions:**

These results reveal dimensions of brain-behaviour associations across diagnostic boundaries, highlighting potent disorder-general patterns as the most prominent. In addition to providing biologically informed patterns of relevant behavioural factors for mental illness, this contributes to a growing body of evidence in favour of transdiagnostic approaches to prevention and intervention.

## Highlights

• Two modes of brain-behaviour associations identified in a clinical sample of youth.
• First mode related to maturation, second mode to social and cognitive impairment.
• Patterns were cross-diagnostic and scaling with number of comorbidities.
• Patterns were generalisable to population-based sample.

## 1 Introduction

Mental illness typically manifest during childhood or adolescence (Caspi et al., 2020, Kessler et al., 2007), alluding to the importance of neurodevelopment for mental health. The interplay of a multitude of factors likely shapes the neurodevelopmental trajectory; however, most studies have typically investigated only one or a few such factors at a time. Associations that are relevant for brain development may in turn be elevated in clinical populations and subsequently relevant for psychopathology. A comprehensive mapping of behavioural factors and how they relate to measures of brain structure in a clinical sample of youth represents a critical step towards understanding the role of neurodevelopment in health and disease.

Empirically derived models of psychopathology point to common symptomatology (i.e. general vulnerability) across classic diagnostic categories. In line with this, abnormalities in both genetics (Lahey et al., 2011, Pettersson et al., 2016, Roelfs et al., 2021), brain structure (Goodkind et al., 2015, Opel et al., 2020) and cognition (Abramovitch et al., 2021, Caspi et al., 2014) are shared across diagnostic syndromes. Furthermore, general psychopathology is linked to deviations from normative cortical (Parkes et al., 2021) and cognitive (Kjelkenes et al., 2022) development, pointing to the relevance of mapping associated behavioural factors across diagnostic boundaries during neurodevelopment.

Multivariate approaches in adults reveal a positive-negative population dimension linking brain features with lifestyle, demographic, and psychometric measures (Smith et al., 2015), in which factors typically considered positive are linked to advantageous or healthy brain features, while negative factors exhibit the opposite pattern. This “positive-negative” axis of covariation has since been reported in studies of adolescents (Modabbernia et al., 2021a) and children (Alnæs et al., 2020, Modabbernia et al., 2021b), alluding to the presence of a link between brain and behaviour for advantageous development already early in life. However, the distribution of such brain-behaviour associations in relation to psychopathology is not well mapped. Investigating brain-behaviour associations in a clinical population of youth may elucidate the relevance of such patterns for mental health.

Symptoms of anxiety, irritability, and attention-deficit hyperactivity disorder (ADHD) have in a previous study been linked to both shared and unique patterns of brain connectivity (Linke et al., 2021). This finding was replicated across two independent clinical samples of youth, suggesting both disorder-general and disorder-specific patterns of psychopathology in the youth brain. Across children with and without an ADHD diagnosis (Ball et al., 2018), higher ADHD symptom load was linked with poorer academic performance, delayed pubertal development, and regional variability in cortical brain structure. However, less is known about how such patterns vary across diagnostic boundaries (Lynch et al., 2021). Identification of shared and distinct patterns of brain-behaviour associations across diagnostic boundaries may provide more informed models of psychopathology, illuminating the role of neurodevelopment and brain-behaviour associations. Such patterns can be determined by utilising multivariate approaches and dimensional clinical and behavioural phenotypes, as employed in several recent studies (Smith et al., 2015, Modabbernia et al., 2021a, Modabbernia et al., 2021b, Alnæs et al., 2020). However, few studies have employed this approach in clinical youth samples, thus the relevance of the reported brain-behaviour relationships remain to be determined.

In the current study we used canonical correlation analysis (CCA) in a sample of youth where the majority had at least one diagnosed psychiatric disorder. The aim was to identify latent dimensions of associations between brain structure and clinical, cognitive, and socio-environmental factors, and to reveal putative and empirically estimated cross-diagnostic and diagnosis-specific factors. By using symptom scores instead of categorical diagnostic information when decomposing the data, we modelled brain associations with dimensional measures of psychopathology (Caspi et al., 2014). Diagnostic information was used to assess the relevance of the detected patterns for clinical diagnosis. To improve interpretability (Smith et al., 2015, Alnæs et al., 2020, Miller et al., 2016), we submitted the CCA scores to independent component analysis (ICA). This procedure results in maximally correlated, maximally interpretable latent dimensions (i.e. modes) across the two high-dimensional datasets. As such, these dimensions link a broad range of behavioural factors that are present across diagnostic boundaries to individual differences in brain structure. If specific brain-behaviour patterns related to each diagnostic category exist, we expected these to appear as distinct modes for each diagnosis. While instead, if the strongest pattern is a cross-diagnostic vulnerability to psychopathology, we expected the analysis to yield one general clinical mode across diagnostic categories.

Finally, we assessed the generalisability and construct validity (Chaytor and Schmitter-Edgecombe, 2003) of the identified clinical brain pattern in an independent population-based sample. First, we derived out-of-sample brain scores using overlapping brain-imaging measures derived from a harmonised protocol across the two samples. We then associated these out-of-sample brain-scores to measures of overlapping clinical and cognitive constructs in the independent sample.

## 2 Materials and methods

### 2.1 Sample

**Fig. 1:**
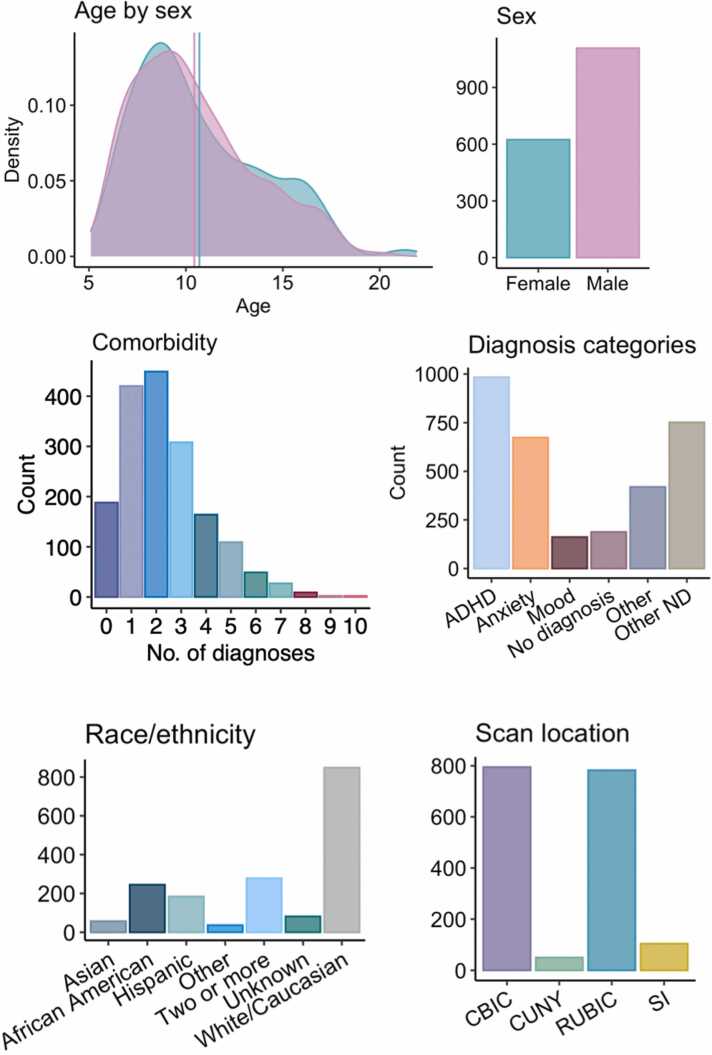
Demographics and clinical characteristics of the sample. Distributions of age by sex, sex, comorbidity, diagnosis categories, racial/ethnic background, and scanner location.

We accessed brian structural, clinical, cognitive, and socio-environmental variables from the Healthy Brain Network (HBN) (Alexander et al., 2017), a cohort consisting of children and adolescents from New York City, USA aged 5–21. The data collection is currently ongoing, with behavioural data from 3628 individuals and magnetic resonance imaging (MRI) data from 2645 individuals having been released by the time of analyses for this study. Individuals were recruited through community sampling in which children with clinical concerns were encouraged to participate. Then, they underwent extensive assessment of biological and behavioural characteristics, such as neuroimaging, neuropsychological testing, psychiatric evaluation, genetics, physical assessment, and interviews regarding environmental, demographical and lifestyle factors. After quality control and data cleaning (described in Section 2.2), the final sample, with both MRI and behavioural data available, consisted of 1732 participants (624 females; mean ± sd age: 10.52 ± 3.17 years). Sample demographics are provided in Fig. 1.

### 2.2 Data pre-processing

Behavioural data from 3628 participants in HBN were processed using R (https://cran.r-project.org). Categorical diagnostic information was removed from the data, keeping only symptom scores. Then variables were cleaned for extreme scores and large amounts of missing data (remaining n = 2603). See Supplementary methods in the Supplementary Material for more detail. MRI measures were obtained from T_1_-weighted scans of 2645 participants. Quality assurance was performed using the MRIQC classifier (Esteban et al., 2017) (n = 2479). For participants with more than one T_1_-weighted scan sequence, we selected the sequence with the best estimated quality. Distributions of imaging quality across scan sequences are shown in Fig. S1.

The selected T_1_-weighted data were then processed using FreeSurfer (Fischl, 2012, Iglesias et al., 2015a, Iglesias et al., 2015b, Iglesias et al., 2018, Saygin et al., 2017, Billot et al., 2020) (see Supp. methods). We extracted cortical thickness, area, and volume for 34 regions of interest per hemisphere using the Desikan-Killiany parcellation, in addition to gyrification indices, nuclei/subfield and subcortical volumes, as well as summary statistics (n = 2440). Next, MRI variables were cleaned and quality controlled (n = 2379, see Supp. methods) and the remaining variables residualised for scanner/site, and T_1_-weighted scan sequence. Volumetric features were residualised for estimated total intracranial volume (eTIV). To also capture associations with global volume, eTIV was included as a variable in the analysis. Both for behavioural and MRI data, remaining missing values were imputed with *knnimpute* and data was normalised using a rank-based normal transformation (*palm_inormal*) from FMRIB Software Library Permutation Analysis of Linear Models (Winkler et al., 2014). The final sample, with both behavioural and MRI data available, consisted of 1732 participants with 793 behavioural variables and 447 imaging variables (see Table S1 and S2 for a list).

### 2.3 CCA-ICA, split-half reliability, and permutation testing

To estimate modes of brain-behaviour-associations across participants, we used ICA with CCA as an intermediate step. The canonical variates from CCA represent linear combinations of the imaging variables that explain variance in linear combinations of the behavioural variables across participants. To facilitate interpretation of the resulting orthogonal canonical variates, and following previous applications of CCA in population imaging (Alnæs et al., 2020, Miller et al., 2016), we submitted the CCA scores to ICA, using the fastICA algorithm (Hyvärinen and Oja, 2000). See Supplementary methods for more detail. To increase robustness, while at the same time avoiding rank deficiency and fitting to noise, we submitted both imaging and behavioural data to principal component analysis (PCA) before running CCA-ICA. All analyses were performed using MATLAB R2020b (Inc, 2020). As part of the analysis, we estimated the optimal dimensionality and decomposition for PCA and ICA and selected the dimensionality yielding the highest split-half reliability for the least reliable component (see Fig. S2 and S3). These tests revealed that results were robust to the choice of dimensionality. Next, the significance of the resulting CCA-ICA modes was tested using permutations (n = 1000), which inherently controls the family-wise error (FWE). To ensure that the initial CCA variates were significant (i.e. prior to ICA), these were also tested using permutations (n = 1000).

### 2.4 Interpretation of CCA-ICA modes

For plotting and interpretation of the resulting CCA-ICA modes, we correlated the CCA-ICA participant weights (i.e. mode loadings) into the original de-confounded data. The resulting correlations reflect the strength with which each variable in the original data load onto the overarching pattern (akin to factor loadings), but do not inform us on the explicit strength of any bivariate relationships between individual variables. A lists of all variables, with correlations and CCA-ICA weights, are shown in Table S3 and S4.

### 2.5 Consistency across age, sex, racial/ethnic background, socioeconomic status, clinical diagnosis, and medication use

To assess the effect of age and sex on each mode, we plotted and regressed the mode loadings against age, age^2^, and sex using linear models. We also reran the CCA-ICA with all behavioural phenotypes residualised with respect to sex. These results revealed similar patterns of covariation as the original analysis (correlations between the original and sex-adjusted results were *r* = 0.94 and *r* = 0.80 for mode 1 and mode 2, respectively). Similarly, we reran the correlations between mode loadings and original data controlling for age, to check the specific influence of age on each mode. These results revealed an almost identical pattern of covariation for mode 2 (*r* = 0.98), indicating that mode 1 (r = 0.86) captured most of the age-related variance. In effect, this age-residualised the data driving an age-invariant mode 2. See Fig. S5 for partial correlations between mode 2 and original data controlling for age.

Considering that factors related to inequality and socioeconomics differ between ethnic groups, these variables were not regressed out of the data. To examine whether the detected modes were generalisable across racial/ethnic background, we plotted the mode loadings by ethnic group (see Fig. S6). Similarly, we plotted the mode loadings by median-split of household income, as a proxy for socioeconomic status (SES; see Fig. S7). We also reran the correlations between mode loadings and original data controlling for household income. These results revealed unchanged patterns of covariation (correlations between the original and income-adjusted results were *r* = 0.99 for both modes), indicating that our results are consistent across socioeconomic levels. The correlation between household income and mode 2 weights was *r* = 0.15.

Based on clinical diagnostic information provided in the HBN sample, each participant was categorised based on their first given diagnosis, as either “ADHD”, “anxiety disorders”, “mood disorders”, “other disorders”, “other neurodevelopmental disorders” or “no diagnosis”. Mode loadings were then regressed against diagnosis, with pairwise comparisons estimated using the *emmeans* package in R and adjusted for multiple comparisons using Tukey. “No diagnosis” was used as a reference group. We also regressed mode loadings against number of diagnoses. All associations were adjusted for age, age^2^, and sex.

As a cross check to investigate whether the dominance of ADHD in the sample influenced our findings, we then ran a leave-one-out-cross-validation of the CCA-ICA, excluding all those in the sample with an ADHD diagnosis. In this analysis, we decomposed the variables by multiplying them with the CCA-ICA weights estimated in the original analysis and then we correlated the mode loadings with the original data, as before. These results revealed similar patterns of covariation as the original analysis (the correlation between the original and leave-out-ADHD results was *r* = 0.97 for both modes), indicating that the dominance of ADHD did not unduly drive our findings. Finally, we also reran the correlations between mode loadings and original data controlling for medication use (yes/no; 288 participants reported yes). These results revealed unchanged patterns of covariation (correlations between the original and medication-adjusted results were *r* = 0.99 for both modes), indicating that our results are consistent across medication use.

### 2.6 Out-of-sample validation

For the validation sample*,* we accessed brain MRI, cognitive, and clinical data from the Philadelphia Neurodevelopmental Cohort (PNC), a large community-based study of brain development in youths aged 8–21 (Satterthwaite et al., 2016). As a sub-sample of the larger study, 1445 participants have undergone MRI. Participants were recruited from a larger genetic study at the Children’s Hospital of Philadelphia, stratified by sex, age, and ethnicity. After pre-processing and quality control, the final sample consisted of 1253 participants (681 females). Age distribution is provided in Fig. S8.

The MRI data was processed using the same analysis pipeline as described above for HBN. Clinical variables included 129 symptom scores decomposed into 7 components using ICA, as reported previously (Alnaes et al., 2018): Attention/ADHD, anxiety, conduct disorder, depression, psychosis prodrome, mania, and obsessive-compulsive disorder (Hettwer et al., 2022). These clinical symptom components reflect increased presence of symptoms. In addition, we included a general symptom burden measure (mean clinical ICA-score). As cognitive measures, we included a general cognitive ability factor (gF, first principal component from a PCA across 12 cognitive tests) (Alnaes et al., 2018) and a social cognitive score (the sum of the Penn Emotion Identification Test and Penn Emotion Differentiation Test) (Moore et al., 2015), in addition to a normative deviation score for cognitive abilities (Kjelkenes et al., 2022), which reflects the deviation of each participant’s cognitive ability relative to same-aged peers.

To assess whether the brain-side of the CCA-ICA results were replicable in the validation sample, we decomposed the PNC MRI variables by multiplying them with the imaging CCA-ICA weights estimated in HBN. To test whether the resulting MRI spatial maps in PNC overlapped with those of HBN, we correlated them and tested the significance of these correlations using spin permutations (Vos de Wael et al., 2020, Alexander-Bloch et al., 2018). Then, to investigate whether the brain-behaviour pattern was generalisable to the validation sample, we tested whether the detected brain pattern in PNC could predict scores on clinical and cognitive measures. To do this, we correlated the brain loadings with clinical and cognitive scores in the PNC sample. These scores were not overlapping with clinical and cognitive scores in HBN, so they could not be directly compared. However, if the clinical and cognitive variables in each sample are ecologically valid, they should yield comparable associations with the detected brain pattern, if the detected pattern is indeed generalisable. To assess the reliability of the associations between derived brain loadings and clinical and cognitive variables in PNC, we performed 1000 bootstraps using resampling with replacement. For each bootstrap iteration we decomposed the MRI variables and correlated the derived brain loadings with the clinical and cognitive measures. The resulting bootstrap distribution was used to calculate the 95 % confidence intervals for the out-of-sample brain scores vs cognitive-clinical correlations.

## 3 Results

### 3.1 Modes of covariation

**Fig. 2:**
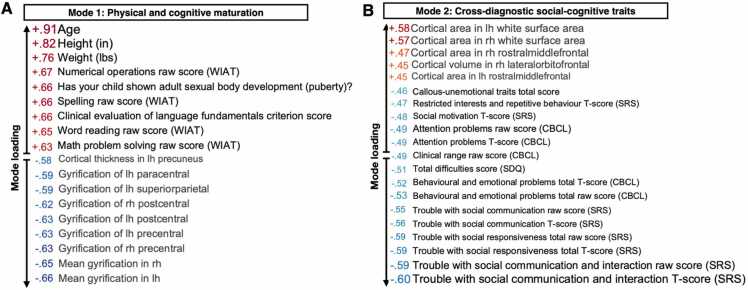
Multivariate pattern of brain-behaviour associations across diagnostic boundaries in youth. **Left:** Mode 1 captures a pattern linking age, physical, and cognitive maturation with lower cortical thickness and gyrification. **Right:** Mode 2 captures a pattern linking trouble with social communication, cognitive ability, and symptoms of psychopathology with lower white matter surface area and gyrification. The values represent correlations between original data values and participant CCA-ICA weights (i.e. mode loadings). Depicted here are the variables with the strongest associations with each mode. In; inches. Lbs; pounds. WIAT; Wechsler individual achievement test. Lh; left hemisphere. Rh; right hemisphere. CBCL; child behavior checklist. SDQ; strengths and difficulties questionnaire. SRS; social responsiveness scale.

By joint multivariate modelling using CCA-ICA, we aimed to delineate linked dimensions (i.e. modes of covariation) between brain structure and clinical, cognitive, and socio-environmental variables in a clinical sample of youth. This analysis identified two such modes of brain-behaviour covariation (both *r* = 0.92, *p*_corr_ = .005 and *p*_corr_ = .006 for mode 1 and mode 2, respectively). In the initial CCA (i.e. prior to ICA), *p*_corr_ = .001 for the two first variates. Each mode of brain-behaviour covariation represents a distinct pattern that relates a weighted set of cognitive, clinical, and socio-environmental factors to a weighted set of brain structures. As shown in Fig. 2A, mode 1 captured a pattern of associations linked to physical and cognitive maturation. The most heavily weighted variables included age, height, weight, pubertal development, and academic performance such as numerical operations, spelling, and word reading. Higher scores on these measures were linked to less parental supervision at home, less need for help with homework, lower prevalence of depressive symptoms, and being able to stay seated in the classroom. In relation to the brain, this mode was associated with lower cortical thickness and gyrification, specifically in the global gyrification index (GI), precentral, postcentral, and paracentral GI, as well as precuneus, superiorparietal, and mean cortical thickness.

Mode 2 captured a pattern of clinical and cognitive scores, independent of age. Specifically, mode 2 linked language skills, academic performance, and trouble with social communication to distinct patterns of brain structure (see Fig. 2B). Trouble with social communication and social cognition overall was associated with worse phonological processing and other indications of language fundamentals, worse academic performance, and having an individualised education plan. These measures were further linked to callous-unemotional traits, lower social status, and higher prevalence of psychological difficulties such as attention problems, externalisation, internalisation, and hyperactivity. This pattern of associations was linked to several brain features, such as lower global white matter surface area, rostral middle frontal cortical area, lateral orbitofrontal cortical volume, and regional as well as mean cortical gyrification. See Fig. S9 for loadings of all variables included in the analysis.

**Fig. 3:**
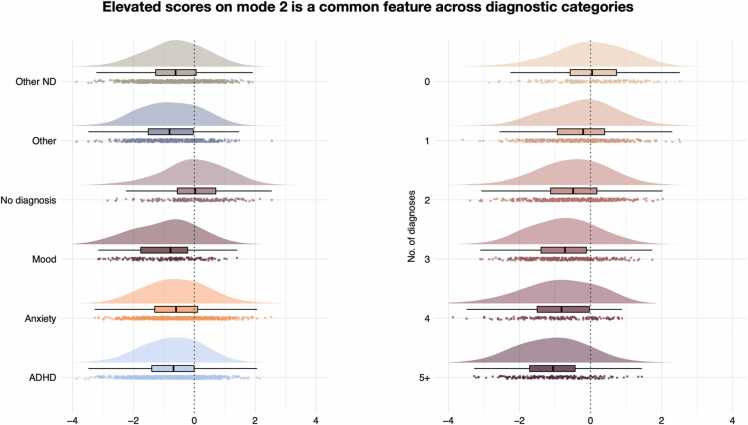
A larger, more negative score on mode 2 (linking social skills, cognitive ability, and psychopathology to brain structure) was a common feature across all diagnostic boundaries. **Left:** All diagnostic categories had a stronger, more negative loading on mode 2 compared to having no diagnosis. **Right:** Stronger, more negative loading on mode 2 by increasing number of diagnoses (comorbidities). Box plot notches exhibit 95 % confidence intervals for comparing medians. Centred around no diagnosis median. ADHD; attention-deficit hyperactivity disorders. Other ND; other neurodevelopmental disorders.

To understand the degree to which these linked dimensions were disorder-general or disorder-specific, we then investigated the extent to which diagnostic categories explained individual differences in loading on each mode. Fig. 3 shows loading on mode 2 by diagnostic category and by number of diagnoses (see Fig. S10 for loading on mode 1). Linear models (see Table 1, S5, S6, and S7) revealed that participants diagnosed with mood disorders showed a higher loading on mode 1, while all diagnostic categories, except anxiety disorders, were associated with more negative loading on mode 2 compared to participants without a diagnosis. Both mode 1 and mode 2 exhibited a significant linear association with the number of diagnoses (see Table S8 and S9). This was true when including “no diagnosis” in the model or not, suggesting that this effect was not driven by case-control effects.

**Table 1:** Pairwise comparisons of associations with each mode between no diagnosis and each diagnostic category. Age, age^2^, and sex are included as covariates.

Comparison	Beta	SE	df	LL	UL	t-value	corr p
**Mode 1**							
ADHD	0.03	0.04	1719	-0.08	0.13	0.73	0.978
Anxiety	0.01	0.04	1719	-0.11	0.13	0.28	1.000
Mood	0.27	0.06	1719	0.09	0.46	4.22	3.7 × 10^-4^
Other	< 0.01	0.06	1719	-0.18	0.17	-0.06	1.00
Other ND	0.06	0.04	1719	-0.05	0.17	1.58	0.611
**Mode 2**							
ADHD	-0.68	0.08	1719	-0.91	-0.46	-8.64	3.7 × 10^-12^
Anxiety	-0.24	0.09	1719	-0.51	0.02	-2.66	0.084
Mood	-0.74	0.14	1719	-1.15	-0.33	-5.15	4.3 × 10^-6^
Other	-0.51	0.14	1719	-0.90	-0.12	-3.71	0.003
Other ND	-0.57	0.09	1719	-0.81	-0.32	-6.59	8.7 × 10^-10^

*Note.* ADHD; attention-deficit hyperactivity disorders. ND; neurodevelopmental disorders. SE; standard error. df; degrees of freedom. LL; lower confidence level (2.5 %). UL; upper confidence level (97.5 %). corr p; p-value adjusted with Tukey.

### 3.2 Out-of-sample validation

**Fig. 4:**
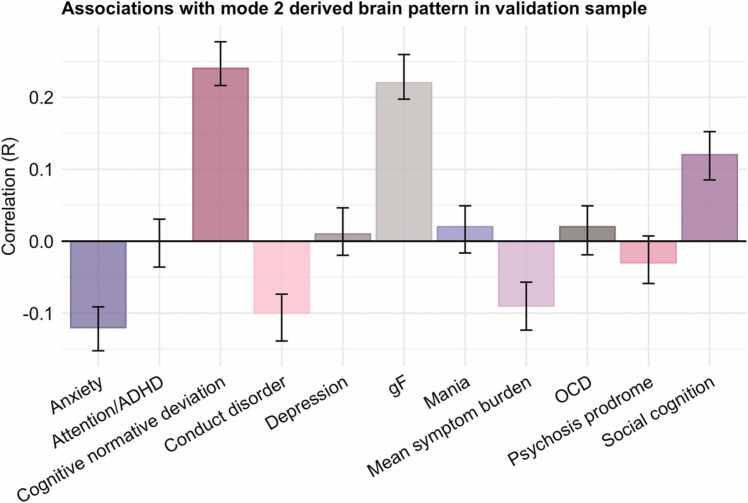
Mode 2 derived brain loadings in PNC correlate with comparable clinical and cognitive measures. A larger, more negative score on mode 2 is correlated with lower cognitive ability and negative deviations from normative cognitive development. A negative cognitive normative deviation indicates poorer cognitive development than expected. Error bars represent bootstrapped 95 % confidence intervals for correlations across 1000 bootstrap-decompositions of the PNC imaging data. ADHD; attention-deficit hyperactivity disorder. OCD; obsessive compulsive disorder. gF; general cognitive ability.

As a final step, we tested the replicability and generalisability of our findings using an independent sample. Using the brain pattern derived from the HBN sample, we estimated feature weights (i.e. loadings) across MRI variables in the PNC sample. Comparing these loadings, we found strong positive correlations between the two samples (*r* = 0.95, *p*_corr_ < .001 and *r* = 0.71, *p*_corr_ < .001 for mode 1 and mode 2, respectively; see Fig. S11 for null distributions of the spin permutation test). As shown in Fig. S12 and S13, the covariation structure across MRI variables in PNC highly resembled HBN. Next, to test the generalisability and predictive ability of the brain patterns to clinical and cognitive measures, we estimated correlations between the derived brain scores in PNC with cognitive and clinical variables. While the measured clinical and cognitive constructs were similar between the two samples, they were not assessed using identical instruments. Thus, this out-of-sample validation also constitutes a test of the external validity of the brain-behaviour relationship. This analysis revealed that a larger, more negative mode 2 brain loading was linked to greater negative deviation from a normative cognitive trajectory, lower cognitive abilities, higher average symptom burden, as well as higher symptoms of anxiety and conduct disorder (see Fig. 4). Mode 2 was largely age invariant, however, to further confirm the age-independence of mode 2, the scores were residualised with respect to age in this plot. Mode 1 exhibited positive associations with age and cognitive abilities, as well as higher average symptom burden (see Fig. S14).

## 4 Discussion

In this study we leveraged the HBN sample, a clinical youth cohort aged 5–21, to delineate dimensions of brain-behaviour associations across diagnostic boundaries in youth. We identified two modes of brain-behaviour covariation, linking maturation, cognitive ability, social skills, and symptoms of psychopathology to individual differences in brain structure. The dimension linking cognitive ability, social skills, and symptoms of psychopathology to brain structure was a common feature across all diagnostic boundaries, suggesting a disorder-general effect. We also demonstrated the generalisability and predictive ability of these patterns in an independent population-based sample with a similar age range. Together, these findings suggest that brain-behaviour associations in youth are broad and transdiagnostic, implicating factors such as cognitive ability and social skills and scaling with the number of comorbid illnesses.

The first mode linked lower cortical thickness and gyrification with age and measures of physical and cognitive maturation, reflecting age-related improvements in school performance, pubertal development, higher height, and weight. This mode replicates previous studies in youth showing lower cortical thickness (Shaw et al., 2008, Mills et al., 2016, Tamnes et al., 2010) and gyrification (Raznahan et al., 2011, Su et al., 2013) with increasing age, as well as cognitive maturation (Chung et al., 2017). Moreover, our results align with a previous multivariate investigation in a longitudinal sample of adolescents (Modabbernia et al., 2021a), identifying the strongest brain-behaviour associations to be between measures of brain structure and sex, age, and indices of maturation. This emphasises common maturational factors as the most important influences on neurodevelopment, also when environmental, demographical, and psychosocial influences were considered.

The second mode captured a pattern of socio-cognitive difficulties associated with lower cortical volume, surface area, and gyrification. Specifically, this pattern reflected difficulties with communicating and relating to peers, worse language development and school performance, and emerging psychological difficulties. Such a “positive-negative” dimension across behavioural, clinical, and socio-environmental factors has previously been linked to individual differences in brain morphology and connectivity in population-based samples (Smith et al., 2015, Alnæs et al., 2020, Modabbernia et al., 2021b). The current results extend these findings by demonstrating their relevance for characterising psychopathology in youth that already have a psychiatric diagnosis. Indeed, we established that the current pattern was detectable also in an independent population-based sample. This overlap between clinical and non-clinical populations lend support to the conceptualisation of psychopathology as existing on a continuum, such as the p-factor framework (Caspi and Moffitt, 2018). Importantly, the pattern we identified was common across all diagnostic boundaries, indicating a disorder-general or shared pattern. Having a higher number of diagnoses (i.e. comorbidities) was also associated with larger deviations (i.e. larger, more negative loading) on mode 2. This is in line with comorbidity as a prevalent feature of mental illness (Plana-Ripoll et al., 2019), as well as the finding that transdiagnostic symptom burden (i.e. the p-factor) is more predictive of clinical life trajectories than any specific diagnosis (Caspi et al., 2020, Caspi et al., 2014). This has implications for prevention and interventions targeting risk for mental illness in youth, as well as the understanding of psychopathology aetiology more broadly.

Previous work on shared brain structural abnormalities across diagnostic boundaries in adults found one latent factor to explain abnormalities associated with major depression, bipolar disorder, schizophrenia, and OCD, while abnormalities in ADHD and autism spectrum disorder (ASD) were largely independent (Opel et al., 2020). Contrary to this, we found a great degree of overlap in brain-behaviour associations across all disorders. In the current work, the brain associations across disorders were constrained by their link to the behaviour-associations, which may explain the different results. Whether neurodevelopmental disorders belong in the general psychopathology domain or rather represent separate entities remains a topic of discussion (Ronald, 2019). Our findings suggest that in terms of brain-behaviour associations, ADHD and ASD belong in the same terrain as other psychiatric disorders.

Cortical surface area was among the highest loading brain measures on mode 2, the dimension linked to cognitive ability, social skills, and psychopathology. Postnatal surface area expansion has been proposed to reflect local cellular events, such as intracortical myelination, gliogenesis, synaptogenesis and dendritic arborization (Hill et al., 2010). In typically developing children, surface area increases until late childhood or early adolescence (Amlien et al., 2016). As such, lower surface area may reflect disadvantageous or delayed brain development. Indeed, smaller surface area has been linked to poorer cognition, poorer physical development, and poorer social environment in children aged 9–10 relative to same-aged peers (Modabbernia et al., 2021b). Given that surface area was adjusted for eTIV in our analyses, the high loading of this brain feature likely reflect cortical folding, the only plausible avenue for expanding cortical surface area without a corresponding expansion of intracranial volume (Mota and Herculano-Houzel, 2015). Indeed, both global and regional cortical gyrification were also among the highest loading brain features on mode 2.

Gyrification typically decreases from middle childhood until young adulthood (Raznahan et al., 2011), and we replicated this age-related gyrification pattern in mode 1. Mode 2 was, however, only weakly associated with age, and the pattern of lower gyrification here was linked to individual differences in clinical and cognitive measures. Common age-related effects appear to be captured by mode 1, as shown by the fact that raw scores and t scores on cognitive tests exhibit overlapping loading on mode 2. Moreover, the pattern of variable loading in mode 2 when controlling for age was largely overlapping with the original uncorrected analysis, further supporting this interpretation. As such, the pattern of associations in mode 2 is to a large extent age invariant and represent other mechanisms than merely the effect of age.

Reduced cortical folding in individuals with socio-cognitive difficulties is in line with previous work relating lower gyrification to neurodevelopmental diagnoses such as ADHD (Wolosin et al., 2009), ASD (Bos et al., 2015), intellectual disability (Zhang et al., 2010), and dyslexia (Casanova et al., 2004). This association may thus represent an important neural correlate for social and neurocognitive difficulties. Indeed, our validation of the mode 2 brain pattern in an independent population-based sample revealed a robust association with deviations from normative cognitive development. These results suggests that cognitive problems represent a relevant characteristic of mental illness across diagnostic boundaries, which is compatible with previous findings identifying cognition as a common risk factor for psychopathology and a core characteristic of general vulnerability for psychopathology (Abramovitch et al., 2021, Caspi et al., 2014, Michelini et al., 2019). Interventions aimed at improving mental health in youth may thus benefit from targeting cognitive development and the environments supporting it, such as schools and education. In line with previous findings linking SES to vulnerability for mental illness (Reiss, 2013), mode 2 was associated with SES. However, the correlation was moderate, suggesting that brain-linked vulnerability cannot be simply explained as SES-driven individual differences.

Other studies have reported shared brain connectivity patterns across anxiety, irritability, and ADHD in other clinical samples of youth (Linke et al., 2021). While substantial evidence now points towards cross-diagnostic brain deviations in psychopathology (Goodkind et al., 2015, Sha et al., 2019), this does not rule out disorder-specific patterns, and a full account of the brain basis of mental illness require mapping of both (Linke et al., 2021, Buckholtz and Meyer-Lindenberg, 2012). Mood disorders predicted mode 1 in addition to mode 2, unlike the other diagnostic categories which were only linked to mode 2. This is likely driven by the fact that individuals with a mood disorder were older than the rest of the sample. Having a higher number of diagnoses was also associated with higher loading on mode 1, likely reflecting the increased prevalence of diagnoses with increasing age (Caspi et al., 2020).

Some limitations should be noted. Acquiring high-quality neuroimaging data in youth and clinical samples is challenging, especially in clinical cohorts. Here we utilised the MRIQC classifier to exclude participants with insufficient image quality and excluded any remining extreme data points from analysis. Both samples applied cross-sectional designs, while longitudinal studies are required to conclude whether the observed age-related individual differences reflect within-person developmental trajectories. Multiple measurements may also allow for determining the dynamic interplay between environmental factors, mental health symptoms, and brain changes, thereby illuminating whether brain changes precede or is a consequence of mental health symptoms (Muetzel et al., 2017). The current sample consisted of largely children with a clinical diagnosis. Although evidence suggests substantial overlap across diagnostic boundaries, we do not know whether those individuals who develop mental illness early in life represent a qualitatively different group in terms of aetiology compared to those developing mental illness during adolescence and early adulthood. Evidence suggests that age-of-onset is an important aspect of the p-factor, which is more predictive of clinical life trajectories than any specific diagnosis (Caspi et al., 2020, Caspi et al., 2014). The identified brain-behaviour patterns were detectable in an independent sample, which further supports the generalisability of our findings and is a strength of the current study.

## 5 Conclusions

In this study, we delineated dimensions of brain-behaviour associations across diagnostic boundaries in youth. In addition to expected patterns of maturation, we found that lower cognitive ability, poor social skills, and symptoms of psychopathology are linked to individual differences in brain structure, and that this is a common feature across diagnostic boundaries. These findings were detectable in an independent sample, supporting their generalisability and predictive ability. In line with the p-factor framework, this suggests that broad and transdiagnostic effects are the most potent patterns of brain-behaviour associations. This emphasises the importance of transdiagnostic approaches in the identification of shared and distinct patterns relevant for psychopathology, a critical step towards more informed models of psychopathology.

## Declaration of Competing Interest

The authors declare the following financial interests/personal relationships which may be considered as potential competing interests: OAA is a consultant to HealthLytix and received speaker’s honoraria from Lundbeck. All other authors report no biomedical financial interests or potential conflicts of interest.

## Data Availability

The data that forms the basis of this work were obtained from the open access Healthy Brain Network (https://healthybrainnetwork.org/) and The Philadelphia Neurodevelopmental Cohort resources (https://www.med.upenn.edu/bbl/philadelphianeurodevelopmentalcohort.html). The code used in the study is available in a public repository (Open Science Framework) (https://osf.io/cjerd/).
