# Supplementary material for: Shared pattern of impaired social communication and cognitive ability in the youth brain across diagnostic boundaries
Source: Dev Cogn Neurosci. 2023 Feb 18;60:101219. doi: 10.1016/j.dcn.2023.101219 (PMC9975702; doi:10.1016/j.dcn.2023.101219)
Supplement: Supplementary file 1 — Supplementary material [file mmc1.pdf]

Supplementary Material

Content

- **Supplementary methods**. Data cleaning and analysis.
- **Figure S1**.Imaging quality estimates across scan sequences in HBN
- **Figure S2**. PCA components and ICA dimensionality
- **Figure S3**. Correlations between differing numbers of PCA components
- **Figure S4**. Distribution of permuted split-half reliability
- **Figure S5**. Partial correlations with mode 2 controlling for age.
- **Figure S6**. Mode loadings across ethnicity
- **Figure S7**. Mode loadings across median split of household income
- **Figure S8**. Age by sex distribution in the PNC sample
- **Figure S9**. Correlations between CCA-ICA weights and variables
- **Figure S10**. Mode loadings across diagnostic boundaries for each mode
- **Figure S11**. Out-of-sample CCA-ICA correlations with PNC data
- **Figure S12**. Surface-based maps of out-of-sample derived brain patterns
- **Figure S13**. ICA-weight correlations with clinical and cognitive variables in PNC
- **Table S1**. List of behavioural phenotypes used in CCA-ICA
- **Table S2**. List of imaging phenotypes used in CCA-ICA
- **Table S3**. List of all variables and mode loadings for mode 1
- **Table S4**. List of all variables and mode loadings for mode 2
- **Table S5**. Linear models of associations of age, sex, and diagnosis with each mode. Diagnosis category included as a fixed factor
- **Table S6**. Linear models of associations of each diagnosis with mode. Age, age^2, and sex are included as covariates
- **Table S7.** Pairwise post hoc comparisons using emmeans and Tukey adjustment
- **Table S8**. Linear models of associations of age, sex, and number of diagnoses (0-10) with each mode. Number of diagnoses included as a continuous variable
- **Table S9**. Linear models of associations of age, sex, and number of diagnoses (1-10) with each mode. Number of diagnoses included as a continuous variable, excluding no diagnosis (0)
- **Supplementary references**

Supplementary methods. Data cleaning and analysis

HBN quality control and data cleaning steps

We identified and excluded any continuous variables with zero or near-zero variance using the nearZeroVar function from the R package caret (https://github.com/topepo/caret/). Categorical variables with a frequency of <5% responses for the least common response, were also excluded. Next, we derived robust *Z* scores for each remaining variable by calculating the deviations from the median absolute deviation (MAD) [1]. Extreme scores with a robust $Z>4$ were set to NaN, and variables with less than 90% of scores remaining were excluded from further analysis. Next, participants with less than 90% retained data were excluded (remaining n = 2603). Of these, 990 participants were excluded due to incomplete data before removing extreme scores, while 35 participants excluded due to extreme scores.

Freesurfer pipeline

The selected T_1-weighted data were then processed using a harmonised analysis pipeline, including automated surface-based morphometry, subcortical segmentation and thalamic, hypothalamic, brainstem, hippocampal, and amygdala nuclei/subfield segmentation from FreeSurfer [v. 7.1.0; v. 7.2 for the hypothalamic segmentation; 2, 3-7]. This segmentation approach has shown to be reliable in 5-year-old children [8], as well as adults. 39 participants were excluded due to missing one or more of the extracted measures. A detailed overview of the scan protocol is available elsewhere (http://fcon_1000.projects.nitrc.org/indi/cmi_healthy_brain_network/MRI%20Protocol.html).

MRI data cleaning steps

Next, we removed participants with a MAD-based $Z>4$ on either the MRIQC classifier composite quality estimate or on the number of surface holes in the uncorrected reconstructed cortical surfaces. As with the behavioural data, we then excluded any variables with less than 90% of values remaining after excluding scores with a $Z>4$, as well as any participants with less than 90% data retained after this procedure (n=2379).

CCA-ICA estimation

CCA-ICA was performed on the de-confounded imaging and behavioural HBN data using MATLAB R2020b. After running PCA and CCA, we summed the imaging and behavioural CCA scores and correlated these with the de-confounded data, before submitting the Fisher *Z*-transformed correlations to ICA. This was done to be able to take full advantage of the variation in the original data and input both imaging and behavioural CCA weights simultaneously to ICA. We estimated the optimal dimensionality and decomposition using a two-pass procedure [9]: in the first pass the optimal CCA-ICA dimensionality was estimated by varying the number of PCA-components submitted to CCA, and then testing the split-half reliability of ICA components to determine the optimal ICA dimensionality. We tested PCA-components and ICA-dimensionalities in two separate runs: between 2-30 varying PCA/CCA and ICA components independently, and between 2-250 using the corresponding number of components for PCA/CCA and ICA. Across 1000 iterations for each tested dimensionality, the sample was randomly split in two halves, and we selected the dimensionality yielding the highest split-half reliability for the least reliable component. We also ran a decomposition with dimensionality fixed at 2. Optimal dimensionality for the first run (2-30) was 10 PCA components and 2 ICA components, and for the second run 2 PCA and ICA components. The Spearman correlation between participant weights was *rho*=.99 and *rho*=.64 for PCA=10 vs PCA=2, *rho*=.98 and *rho*=.84 for PCA=10 vs PCA=30, and *rho*=.96 and *rho*=.74 for PCA=10 vs PCA=250 (canonical modes 1 and 2, respectively). See eFigure 3 for correlations between differing numbers of PCA components. A second pass of CCA-ICA was then run using the estimated optimal dimensionality to derive the ICA decomposition with the highest split-half reliability (n=3000 iterations).

Next, to test the significance of the resulting CCA-ICA modes, we ran permutations (n=1000), with each permutation iteration including the n=3000 iteration search for optimal dimensionality and collected the maximum split-half R-value to form a null distribution (see Figure S5). This was used to calculate familywise error-corrected *p*-values by dividing the count of permuted maximum R values (including the observed non-permuted value) ≥ the non-permuted R values by the number of permutations. Using the same permutation approach (n=1000), the significance of each canonical variate was also tested as an intermediate step. Of the 10 canonical variates estimated from 10 PCA components, the first two were significant (the familywise error-corrected *p*-value was $p=.001$ for each).

Figure S1. Imaging quality estimates across scan sequences in HBN

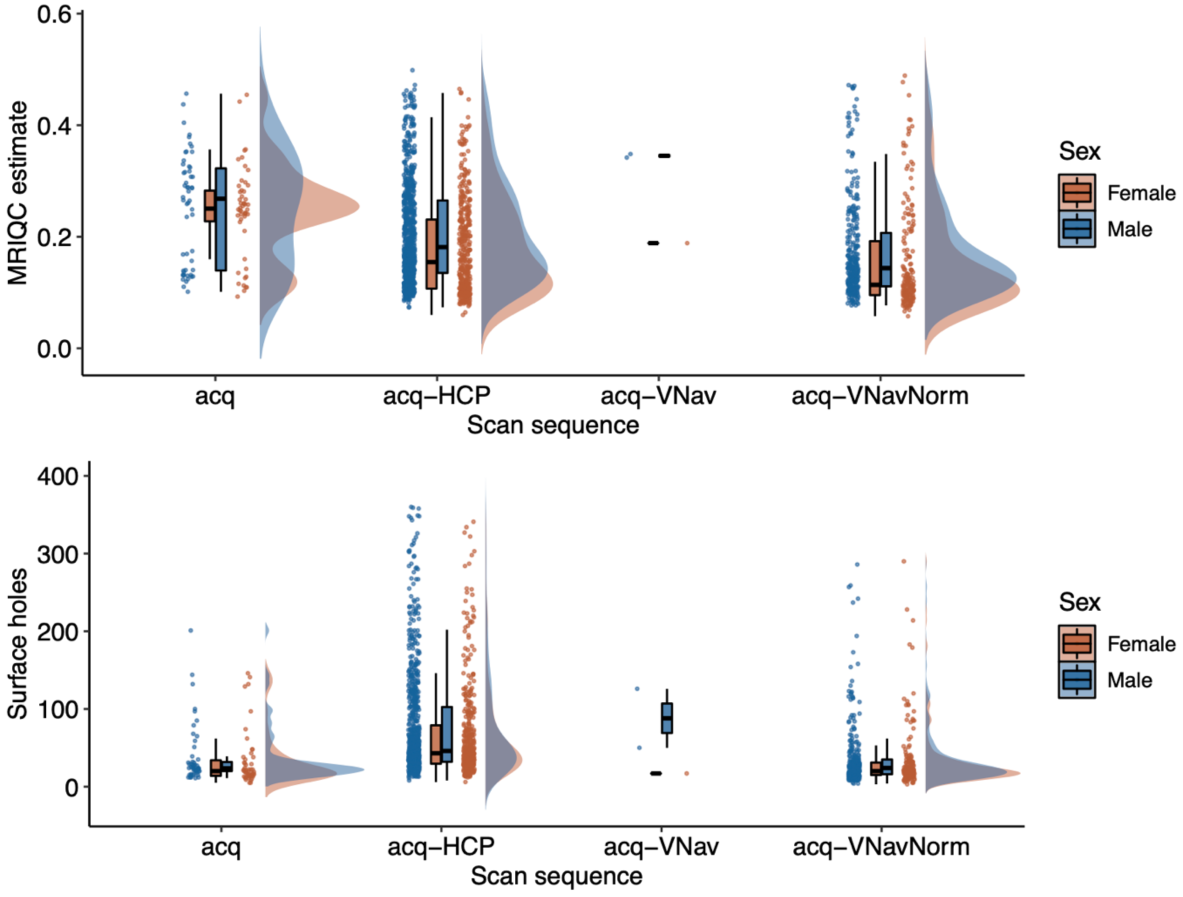

Figure S2. PCA components and ICA dimensionality

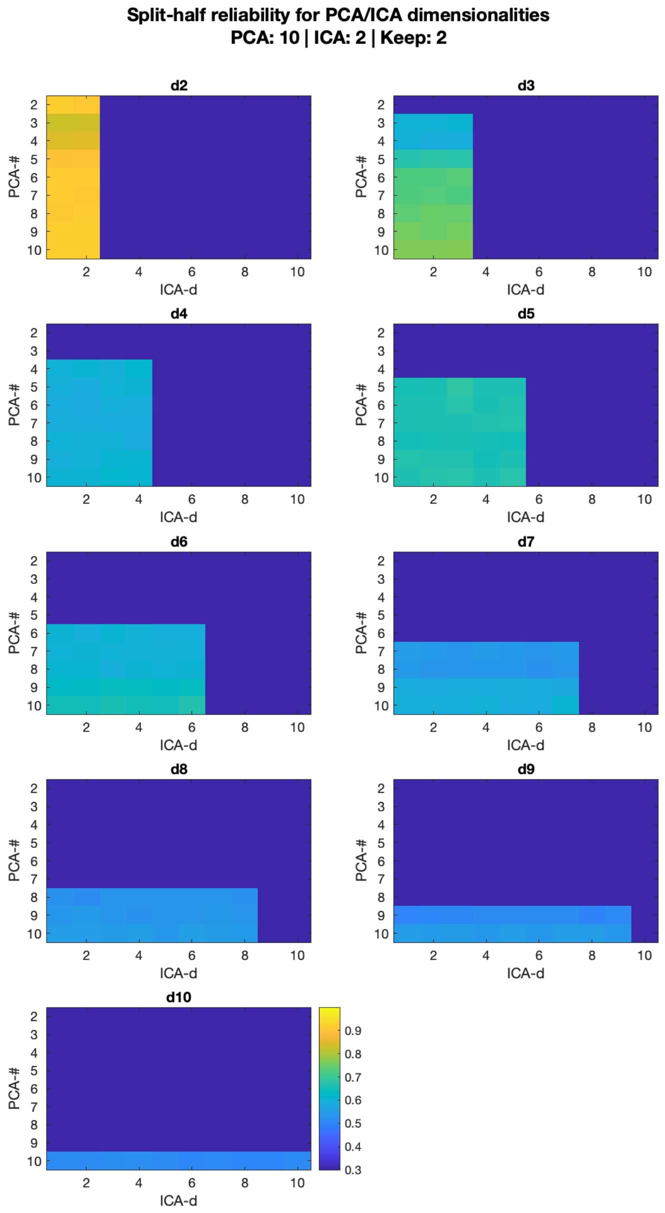

Figure S3. Correlations between differing numbers of PCA components

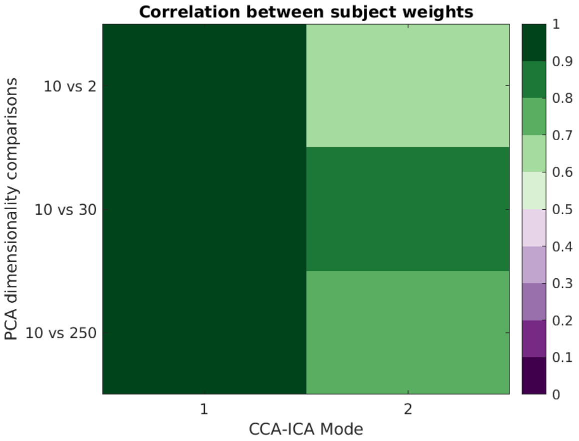

Figure S4. Distribution of permuted split-half reliability

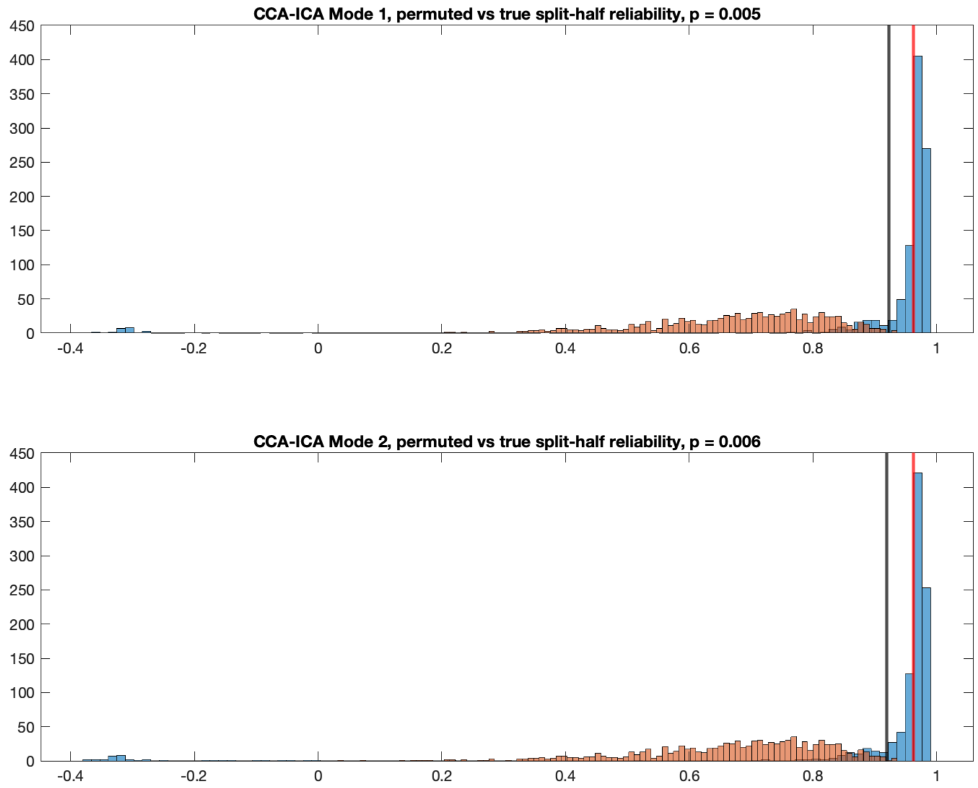

Figure S5. Partial correlations withmode 2 and original data adjusted for age

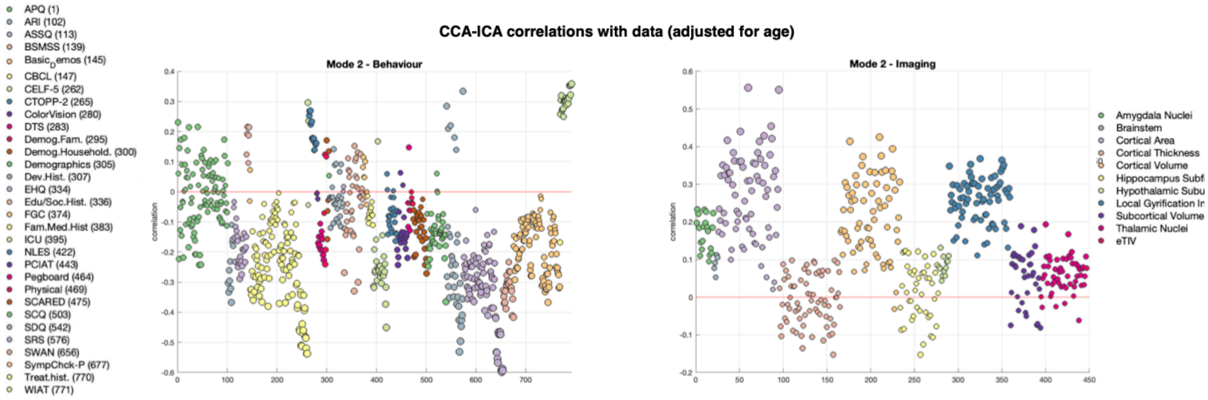

Figure S6. Mode loadings across ethnicity

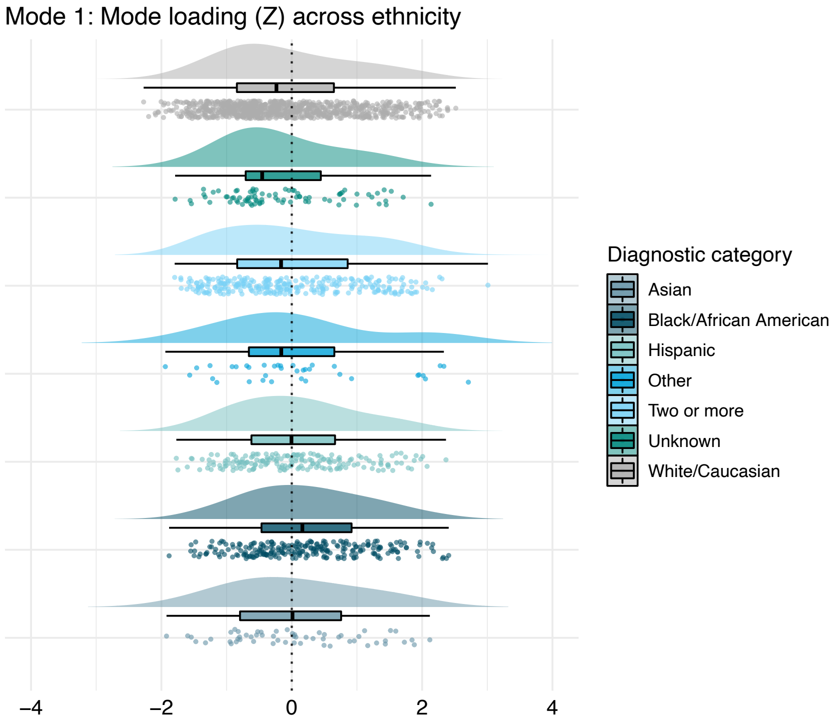

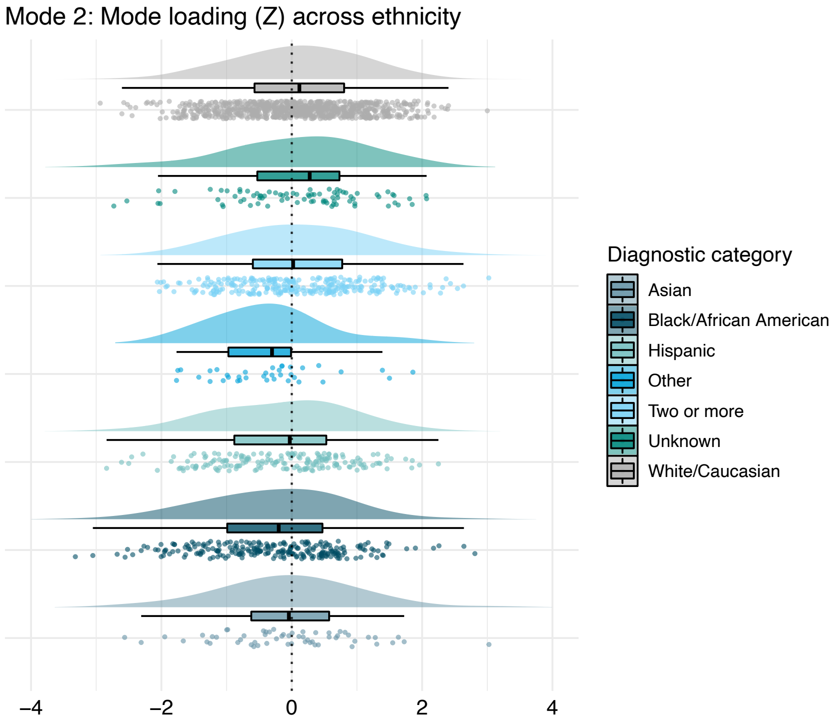

Figure S7. Mode loadings across median split of household income

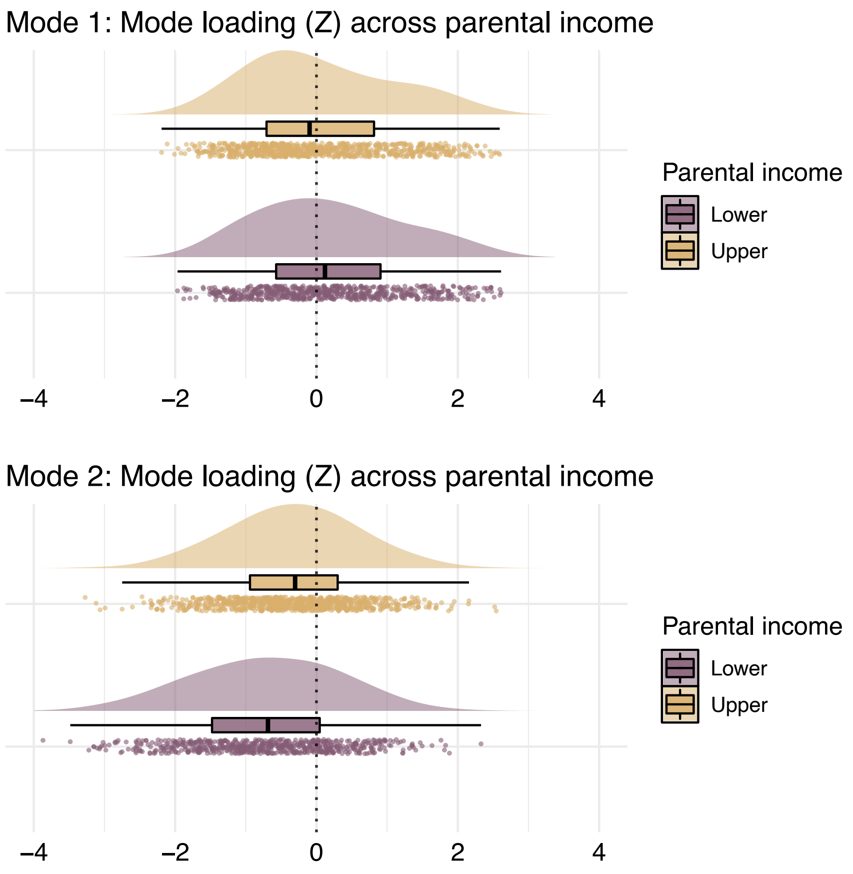

Figure S8. Age by sex distribution in the PNC sample

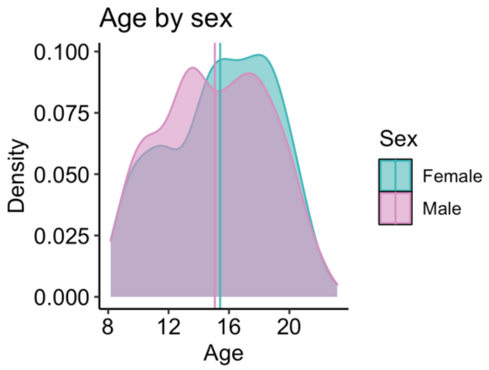

Figure S9. Correlations between CCA-ICA weights and variables

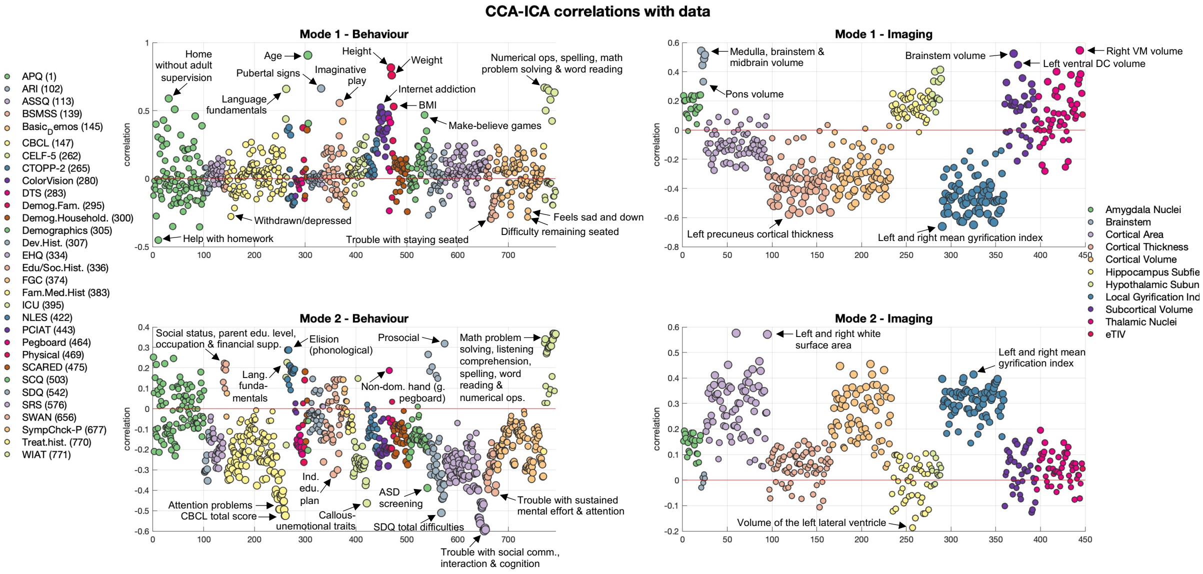

Figure S10. Mode loadings across diagnostic boundaries for each mode

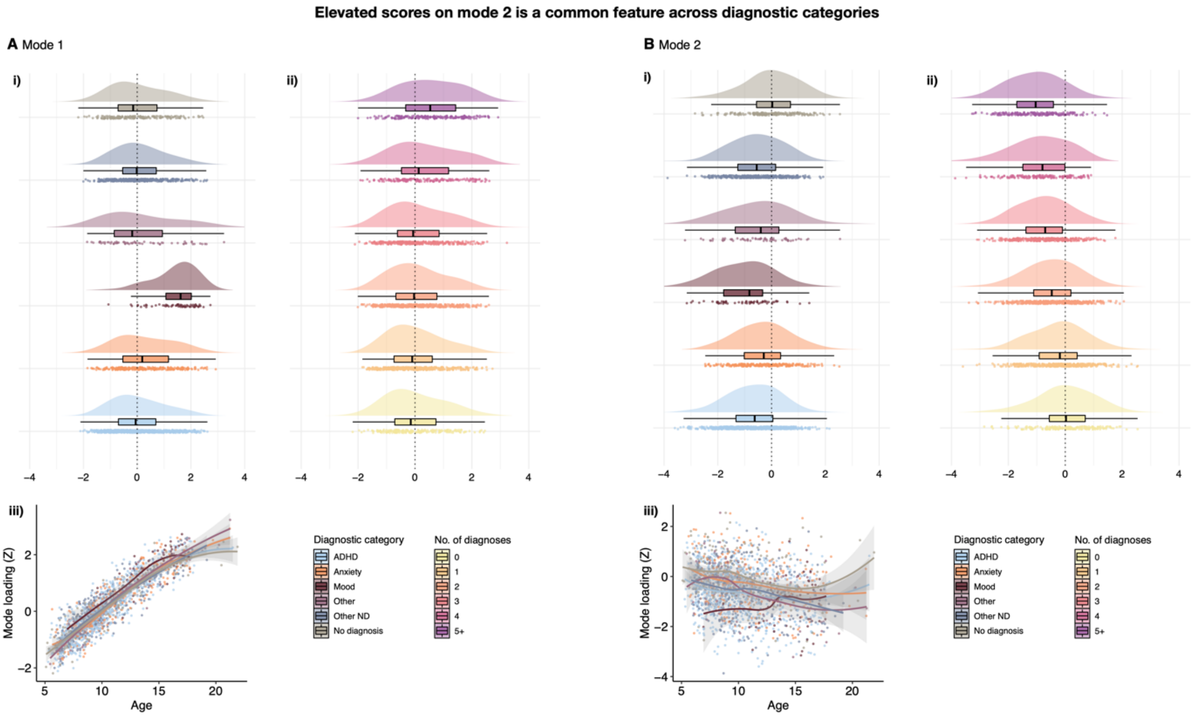

Figure S11. Null distributions from the spin permutation test of the correlation between HBN and PNC brain features

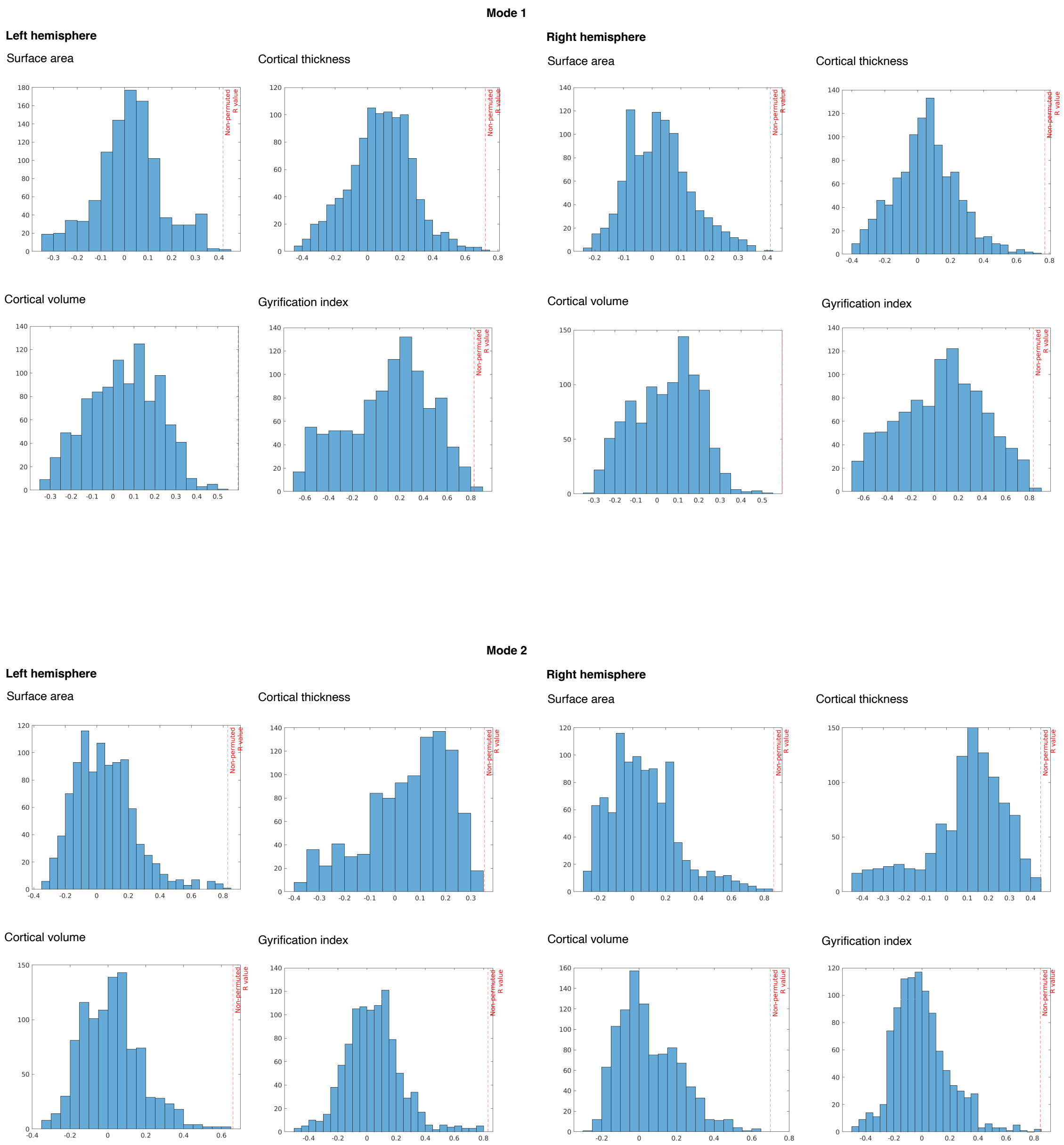

Figure S12. Correlations between CCA-ICA weights and PNC MRI variables

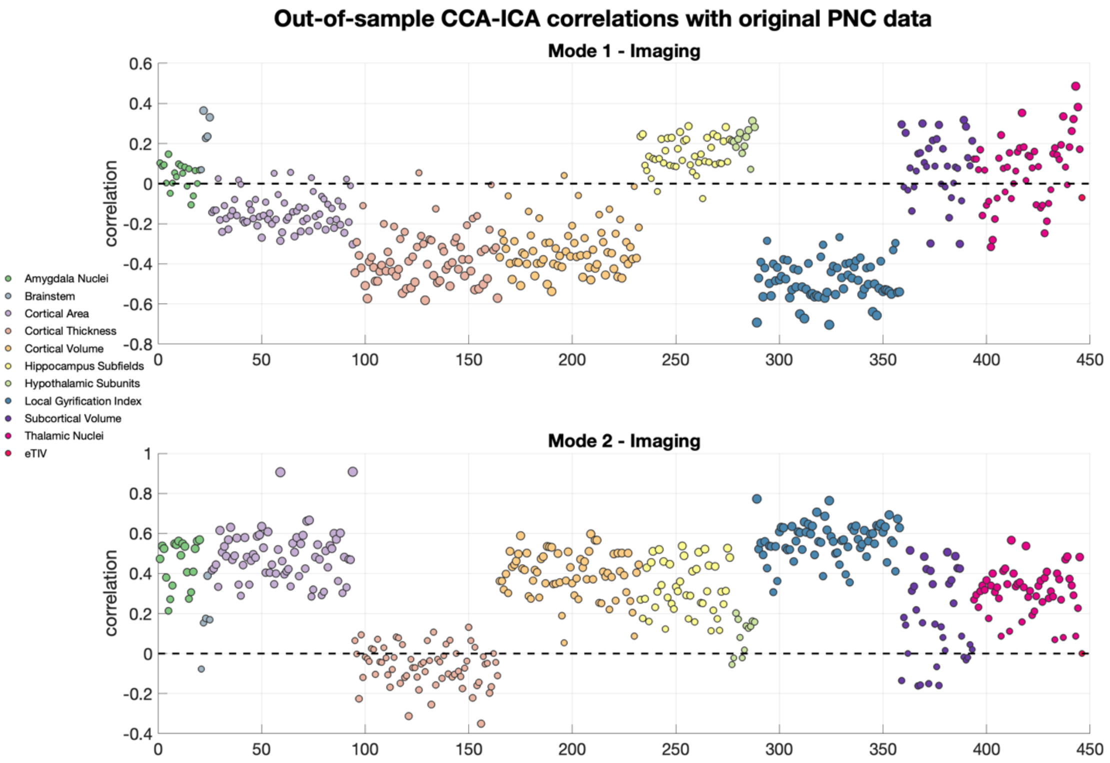

Figure S13. Surface-based maps of out-of-sample derived brain patterns

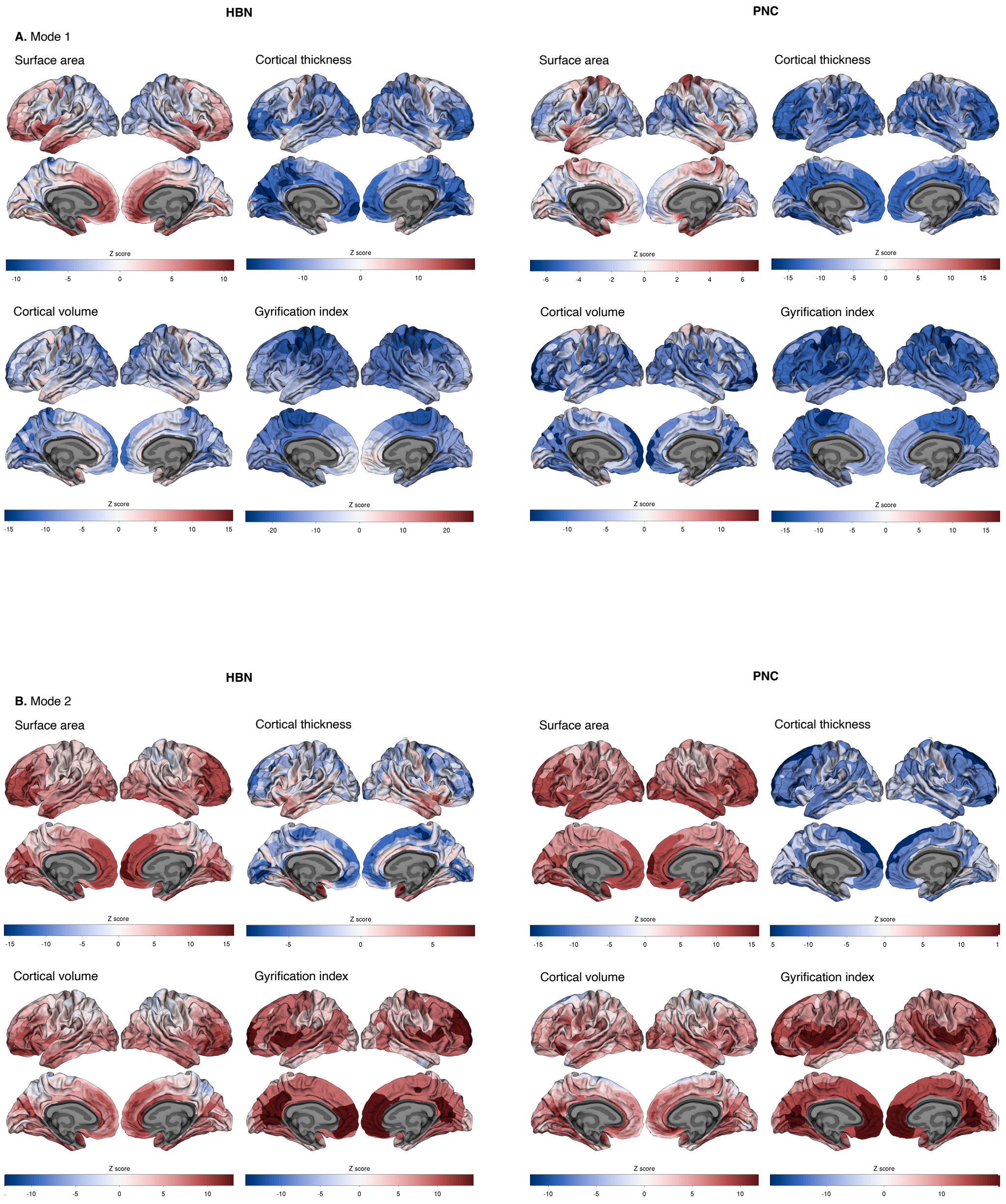

Figure S14. ICA-weight correlations with clinical and cognitive variables in PNC (not adjusted for age)

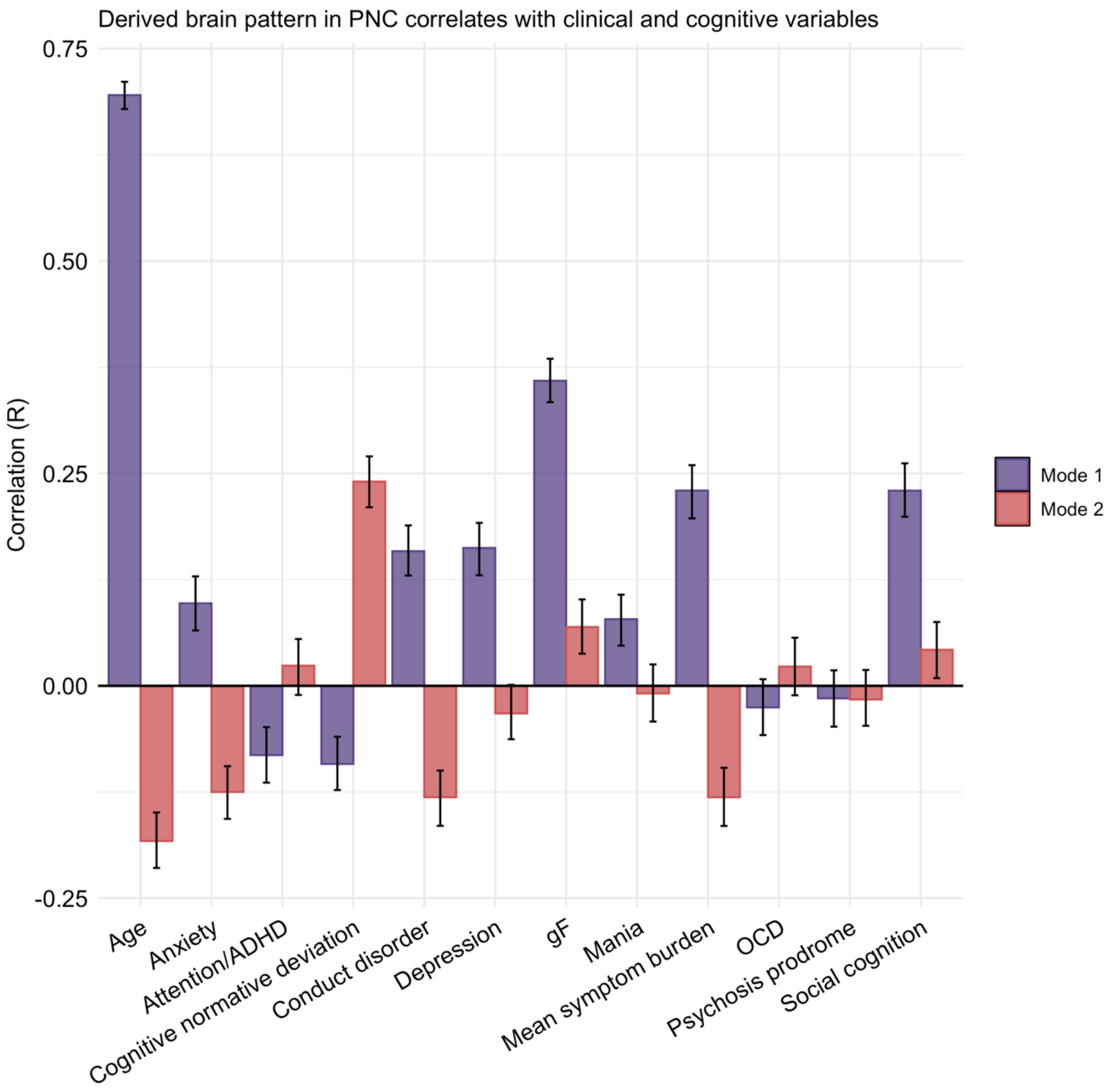

Table S1. List of behavioural phenotypes used in CCA-ICA

Variable name in data	Item text	Instrument name	Instrument abbreviation (if applicable)
Age	Age	Demographics	Demographics
Age2	Age squared	Demographics	Demographics
APQ_P_APQ_P_01	1. You have a friendly talk with your child	Alabama Parenting Questionnaire	APQ
APQ_P_APQ_P_02	2. You let your child know when he/she is doing a good job with something	Alabama Parenting Questionnaire	APQ
APQ_P_APQ_P_03	3. You threaten to punish your child and then do not actually punish him/her	Alabama Parenting Questionnaire	APQ
APQ_P_APQ_P_04	4. You volunteer to help with special activities that your child is involved with (such as sports, boy/girl scouts, church youth groups)	Alabama Parenting Questionnaire	APQ
APQ_P_APQ_P_05	5. You reward or give something extra to your child for obeying you or behaving well	Alabama Parenting Questionnaire	APQ
APQ_P_APQ_P_06	6. You child fails to leave a note to let you know where he/she is going	Alabama Parenting Questionnaire	APQ
APQ_P_APQ_P_07	7. You play games or do other fun things with your child	Alabama Parenting Questionnaire	APQ
APQ_P_APQ_P_08	8. Your child talks you out of being punished after he/she has done something wrong	Alabama Parenting Questionnaire	APQ
APQ_P_APQ_P_09	9. You ask your child about his/her day in school	Alabama Parenting Questionnaire	APQ
APQ_P_APQ_P_10	10. Your child stays out in the evening past the time that he/she is supposed to be home	Alabama Parenting Questionnaire	APQ
APQ_P_APQ_P_11	11. You help your child with his/her homework	Alabama Parenting Questionnaire	APQ
APQ_P_APQ_P_12	12. You give up trying to get your child to obey you because it's too much trouble	Alabama Parenting Questionnaire	APQ
APQ_P_APQ_P_13	13. You compliment your child when he/she has done something well	Alabama Parenting Questionnaire	APQ
APQ_P_APQ_P_14	14. You ask your child what his/her plans are for the coming day	Alabama Parenting Questionnaire	APQ

Variable name in data	Item text	Instrument name	Instrument abbreviation (if applicable)
APQ_P_APQ_P_15	15. You drive your child to a special activity	Alabama Parenting Questionnaire	APQ
APQ_P_APQ_P_16	16. You praise your child for behaving well	Alabama Parenting Questionnaire	APQ
APQ_P_APQ_P_17	17. You do not know the friends your child is with	Alabama Parenting Questionnaire	APQ
APQ_P_APQ_P_18	18. You hug or kiss your child when he/she has done something well	Alabama Parenting Questionnaire	APQ
APQ_P_APQ_P_19	19. Your child goed out with a set time to be home	Alabama Parenting Questionnaire	APQ
APQ_P_APQ_P_20	20. You talks to your child about his/her friends	Alabama Parenting Questionnaire	APQ
APQ_P_APQ_P_21	21. Your child goes out after dark without an adult	Alabama Parenting Questionnaire	APQ
APQ_P_APQ_P_22	22. You let your child out of a punishment early (like lift restrictions earlier than you originally said)	Alabama Parenting Questionnaire	APQ
APQ_P_APQ_P_23	23. Your child helps plan family activities	Alabama Parenting Questionnaire	APQ
APQ_P_APQ_P_24	24. You get so busy that you forget where your child is and what he/she is doing	Alabama Parenting Questionnaire	APQ
APQ_P_APQ_P_25	25. Your child is not punished when he/she has done something wrong	Alabama Parenting Questionnaire	APQ
APQ_P_APQ_P_26	26. you attend PTA meetings, parent/teacher conderences, or other meetings at your child's school	Alabama Parenting Questionnaire	APQ
APQ_P_APQ_P_28	28. You don't check that your child comes home at the time he/she was supposed to	Alabama Parenting Questionnaire	APQ
APQ_P_APQ_P_29	29. You don't tell your child where you are going	Alabama Parenting Questionnaire	APQ
APQ_P_APQ_P_30	30. Your child comes home from school more than an hour past the time you expect him/her to be home	Alabama Parenting Questionnaire	APQ

Variable name in data	Item text	Instrument name	Instrument abbreviation (if applicable)
APQ_P_APQ_P_31	31. The punishment you give your child depends on your mood	Alabama Parenting Questionnaire	APQ
APQ_P_APQ_P_32	32. Your child is at home without adult supervision	Alabama Parenting Questionnaire	APQ
APQ_P_APQ_P_33	33. You spank your child with your hand when he/she has done something wrong	Alabama Parenting Questionnaire	APQ
APQ_P_APQ_P_34	34. You ignore your child when he/she is misbehvaing	Alabama Parenting Questionnaire	APQ
APQ_P_APQ_P_35	35. You slap your child when he/she has done something wrong	Alabama Parenting Questionnaire	APQ
APQ_P_APQ_P_36	36. You take away privileges or money from your child as punishment	Alabama Parenting Questionnaire	APQ
APQ_P_APQ_P_37	37. You send your child to his/her room as punishment	Alabama Parenting Questionnaire	APQ
APQ_P_APQ_P_39	39. You yell or scream at your child when he/she has done something wrong	Alabama Parenting Questionnaire	APQ
APQ_P_APQ_P_40	40. You calmly explain to your child why his/her behavior was wrong when he/she misbehaves	Alabama Parenting Questionnaire	APQ
APQ_P_APQ_P_41	41. You use time out him/her sit or stand in a corner) as punishment	Alabama Parenting Questionnaire	APQ
APQ_P_APQ_P_42	42. You give your child extra chores as punishment	Alabama Parenting Questionnaire	APQ
APQ_P_APQ_P_INV	Involvement Score	Alabama Parenting Questionnaire	APQ
APQ_P_APQ_P_PP	Positive Parenting Score	Alabama Parenting Questionnaire	APQ
APQ_P_APQ_P_PM	Poor Monitoring/Supervision Score	Alabama Parenting Questionnaire	APQ
APQ_P_APQ_P_ID	Inconsistent Discipline Score	Alabama Parenting Questionnaire	APQ
APQ_P_APQ_P_CP	Corporal Punishment Score	Alabama Parenting Questionnaire	APQ
APQ_P_APQ_P_Total	APQ Total Score	Alabama Parenting Questionnaire	APQ
APQ_P_APQ_P_OPD	Other Discipline Practices Score (Not factored into total	Alabama Parenting Questionnaire	APQ

Variable name in data	Item text	Instrument name	Instrument abbreviation (if applicable)
	score but provides item level information)		
APQ_SR_APQ_SR_01	1. You have a friendly talk with your mom	Alabama Parenting Questionnaire	APQ
APQ_SR_APQ_SR_01A	1A. How about your dad?	Alabama Parenting Questionnaire	APQ
APQ_SR_APQ_SR_02	2. Your parents tell you that you are doing a good job	Alabama Parenting Questionnaire	APQ
APQ_SR_APQ_SR_03	3. Your parents threaten to punish you and then not do it	Alabama Parenting Questionnaire	APQ
APQ_SR_APQ_SR_04	4. Your mom helps you with some of your special acivities (such as sports, boy/girl scouts, church youth groups)	Alabama Parenting Questionnaire	APQ
APQ_SR_APQ_SR_04A	4A. How about your dad?	Alabama Parenting Questionnaire	APQ
APQ_SR_APQ_SR_05	5. Your parents reward or give something extra to you for behaving well	Alabama Parenting Questionnaire	APQ
APQ_SR_APQ_SR_06	6. You fail to leave a note or let your parents know where you are going	Alabama Parenting Questionnaire	APQ
APQ_SR_APQ_SR_07	7. You play games or do other fun things with your mom	Alabama Parenting Questionnaire	APQ
APQ_SR_APQ_SR_08	8. You talk your parents out of punishing you after you have done something wrong	Alabama Parenting Questionnaire	APQ
APQ_SR_APQ_SR_09	9. Your mom asks you about your day in school	Alabama Parenting Questionnaire	APQ
APQ_SR_APQ_SR_10	10. You stay out in the evening past the time you are supposed to be home	Alabama Parenting Questionnaire	APQ
APQ_SR_APQ_SR_11	11. Your mom helps you with your homework	Alabama Parenting Questionnaire	APQ
APQ_SR_APQ_SR_12	12. Your parents give up trying to get you to obey them because it's too much trouble	Alabama Parenting Questionnaire	APQ
APQ_SR_APQ_SR_13	13. Your parents compliment you when you have done something well	Alabama Parenting Questionnaire	APQ

Variable name in data	Item text	Instrument name	Instrument abbreviation (if applicable)
APQ_SR_APQ_SR_14	14. Your mom asks you what your plans are for the coming day	Alabama Parenting Questionnaire	APQ
APQ_SR_APQ_SR_14A	14A. How about your dad?	Alabama Parenting Questionnaire	APQ
APQ_SR_APQ_SR_15	15. Your mom drives you to a special activity	Alabama Parenting Questionnaire	APQ
APQ_SR_APQ_SR_15A	15A. How about your dad?	Alabama Parenting Questionnaire	APQ
APQ_SR_APQ_SR_16	16. Your parents praise you for behaving well	Alabama Parenting Questionnaire	APQ
APQ_SR_APQ_SR_17	17. Your parents do not know the friends you are with	Alabama Parenting Questionnaire	APQ
APQ_SR_APQ_SR_18	18. Your parents hug or kiss you when you have done something well	Alabama Parenting Questionnaire	APQ
APQ_SR_APQ_SR_19	19. You go out with a set time to be home	Alabama Parenting Questionnaire	APQ
APQ_SR_APQ_SR_20	20. Your mom talks to you about your friends	Alabama Parenting Questionnaire	APQ
APQ_SR_APQ_SR_22	22. Your parents let you out of a punishment early (like lift restrictions earlier than they originally said)	Alabama Parenting Questionnaire	APQ
APQ_SR_APQ_SR_23	23. You help plan family activities	Alabama Parenting Questionnaire	APQ
APQ_SR_APQ_SR_24	24. Your parents get so busy that they forget where you are and what you are doing	Alabama Parenting Questionnaire	APQ
APQ_SR_APQ_SR_25	25. Your parents do not punish you when you have done something wrong	Alabama Parenting Questionnaire	APQ
APQ_SR_APQ_SR_26	26. Your mom goes to a meeting at school, like a PTA meeting, or a parent/teacher conference	Alabama Parenting Questionnaire	APQ
APQ_SR_APQ_SR_26A	26A. How about your dad?	Alabama Parenting Questionnaire	APQ
APQ_SR_APQ_SR_27	27. Your parents tell you that they like it when you help out around the house	Alabama Parenting Questionnaire	APQ
APQ_SR_APQ_SR_28	28. You stay out later than you are supposed to and your parents don't know it	Alabama Parenting Questionnaire	APQ

Variable name in data	Item text	Instrument name	Instrument abbreviation (if applicable)
APQ_SR_APQ_SR_29	29. Your parents leave the house and don't tell you where they are going	Alabama Parenting Questionnaire	APQ
APQ_SR_APQ_SR_30	30. You come home from school more than an hour past the time your parents expect you to be home	Alabama Parenting Questionnaire	APQ
APQ_SR_APQ_SR_31	31. The punishment your parents give depends on their mood	Alabama Parenting Questionnaire	APQ
APQ_SR_APQ_SR_32	32. You are at home without an adult being with you	Alabama Parenting Questionnaire	APQ
APQ_SR_APQ_SR_33	33. Your parents spank you with their hand when you have done something wrong	Alabama Parenting Questionnaire	APQ
APQ_SR_APQ_SR_34	34. Your parents ignore you when you are misbehvaing	Alabama Parenting Questionnaire	APQ
APQ_SR_APQ_SR_35	35. Your parents slap you when you have done something wrong	Alabama Parenting Questionnaire	APQ
APQ_SR_APQ_SR_36	36. Your parents take away a privilege or money from you as punishment	Alabama Parenting Questionnaire	APQ
APQ_SR_APQ_SR_37	37. Your parents send you to your room as punishment	Alabama Parenting Questionnaire	APQ
APQ_SR_APQ_SR_38	38. Your parents hit you with a belt, switch, or other object when you have done something wrong	Alabama Parenting Questionnaire	APQ
APQ_SR_APQ_SR_39	39. Your parents yell or scream at you when you have done something wrong	Alabama Parenting Questionnaire	APQ
APQ_SR_APQ_SR_40	40. Your parents calmly explain to you why your behavior was wrong when you misbehave	Alabama Parenting Questionnaire	APQ
APQ_SR_APQ_SR_41	41. Your parents use time out (make you sit or stand in a corner) as punishment	Alabama Parenting Questionnaire	APQ
APQ_SR_APQ_SR_42	42. Your parents give you extra chores as punishment	Alabama Parenting Questionnaire	APQ
APQ_SR_APQ_SR_INV_D	Mother Involvement Score	Alabama Parenting Questionnaire	APQ

Variable name in data	Item text	Instrument name	Instrument abbreviation (if applicable)
APQ_SR_APQ_SR_INV_M	Father Involvement Score	Alabama Parenting Questionnaire	APQ
APQ_SR_APQ_SR_PP	Positive Parenting Score	Alabama Parenting Questionnaire	APQ
APQ_SR_APQ_SR_PM	Poor Monitoring/Supervision Score	Alabama Parenting Questionnaire	APQ
APQ_SR_APQ_SR_ID	Inconsistent Discipline Score	Alabama Parenting Questionnaire	APQ
APQ_SR_APQ_SR_CP	Corporal Punishment Score	Alabama Parenting Questionnaire	APQ
APQ_SR_APQ_SR_OPD	APQ Total Score	Alabama Parenting Questionnaire	APQ
APQ_SR_APQ_SR_Total	Other Discipline Practices Score (Not factored into total score but provides item level information)	Alabama Parenting Questionnaire	APQ
ARI_P_ARI_P_01	Is easily annoyed by others	Affective Reactivity Index	ARI
ARI_P_ARI_P_02	Often loses his/her temper	Affective Reactivity Index	ARI
ARI_P_ARI_P_03	Stays angry for a long time	Affective Reactivity Index	ARI
ARI_P_ARI_P_04	Is angry most of the time	Affective Reactivity Index	ARI
ARI_P_ARI_P_05	Gets angry frequently	Affective Reactivity Index	ARI
ARI_P_ARI_P_06	Loses temper easily	Affective Reactivity Index	ARI
ARI_P_ARI_P_Total_Score	Total Score	Affective Reactivity Index	ARI
ARI_S_ARI_S_01	I am easily annoyed by others	Affective Reactivity Index	ARI
ARI_S_ARI_S_02	I often lose my temper	Affective Reactivity Index	ARI
ARI_S_ARI_S_06	I lose my temper easily	Affective Reactivity Index	ARI
ARI_S_ARI_S_Total_Score	Total Score	Affective Reactivity Index	ARI
ASSQ_ASSQ_01	is old-fashioned or precocious	Autism Spectrum Screening Questionnaire	ASSQ
ASSQ_ASSQ_02	is regarded as an ‘eccentric professor’ by the other children	Autism Spectrum Screening Questionnaire	ASSQ
ASSQ_ASSQ_03	lives somewhat in a world of his/her own with restricted idiosyncratic intellectual interests	Autism Spectrum Screening Questionnaire	ASSQ
ASSQ_ASSQ_04	accumulates facts on certain subjects (good rote memory) but does not really understand the meaning	Autism Spectrum Screening Questionnaire	ASSQ

Variable name in data	Item text	Instrument name	Instrument abbreviation (if applicable)
ASSQ_ASSQ_05	has a literal understanding of ambiguous and metaphoric language (i.e. takes things literally; troubles understanding expressions or metaphors)	Autism Spectrum Screening Questionnaire	ASSQ
ASSQ_ASSQ_06	has a deviant style of communication with a formal, fussy, ‘old-fashioned’ or ‘robotlike’ language (i.e. talks differently than other children, in a formal or stilted way)	Autism Spectrum Screening Questionnaire	ASSQ
ASSQ_ASSQ_07	invents idiosyncratic words and expressions (i.e. makes up his or her own words, expressions or names for things)	Autism Spectrum Screening Questionnaire	ASSQ
ASSQ_ASSQ_08	has a different voice or speech	Autism Spectrum Screening Questionnaire	ASSQ
ASSQ_ASSQ_09	expresses sounds involuntarily; clears throat, grunts, smacks, cries or screams	Autism Spectrum Screening Questionnaire	ASSQ
ASSQ_ASSQ_10	is surprisingly good at some things and surprisingly poor at others	Autism Spectrum Screening Questionnaire	ASSQ
ASSQ_ASSQ_11	uses language freely but fails to make adjustments to fit social contexts or the needs of different listeners	Autism Spectrum Screening Questionnaire	ASSQ
ASSQ_ASSQ_12	lacks empathy (i.e. tends to see things only from his/her own perspective, and has troubles seeing things from other's perspective)	Autism Spectrum Screening Questionnaire	ASSQ
ASSQ_ASSQ_13	makes naïve and embarrassing remarks	Autism Spectrum Screening Questionnaire	ASSQ
ASSQ_ASSQ_14	has a deviant style of gaze (e.g. may range from not looking people in the eye, to the other extreme of staring directly at	Autism Spectrum Screening Questionnaire	ASSQ

Variable name in data	Item text	Instrument name	Instrument abbreviation (if applicable)
	people to the point it makes them uncomfortable)		
ASSQ_ASSQ_15	wishes to be sociable but fails to make relationships with peers	Autism Spectrum Screening Questionnaire	ASSQ
ASSQ_ASSQ_16	can be with other children but only on his/her terms	Autism Spectrum Screening Questionnaire	ASSQ
ASSQ_ASSQ_18	lacks common sense	Autism Spectrum Screening Questionnaire	ASSQ
ASSQ_ASSQ_19	is poor at games; no idea of cooperating in a team, scores ‘own goals’	Autism Spectrum Screening Questionnaire	ASSQ
ASSQ_ASSQ_20	has clumsy, ill coordinated, ungainly, awkward movements or gestures	Autism Spectrum Screening Questionnaire	ASSQ
ASSQ_ASSQ_21	has involuntary face or body movements (i.e. any tics?)	Autism Spectrum Screening Questionnaire	ASSQ
ASSQ_ASSQ_22	has difficulties in completing simple daily activities because of compulsory repetition of certain actions or thoughts (i.e. any habits that s/he just has to do?)	Autism Spectrum Screening Questionnaire	ASSQ
ASSQ_ASSQ_23	has special routines; insists on no change (i.e. may need to have exactly the same change; troubles with even the slightest change in his/her environment, or routines or activities)	Autism Spectrum Screening Questionnaire	ASSQ
ASSQ_ASSQ_24	shows idiosyncratic attachment to objects (i.e. may get strangely attached to objects as if they were people)	Autism Spectrum Screening Questionnaire	ASSQ
ASSQ_ASSQ_25	is bullied by other children	Autism Spectrum Screening Questionnaire	ASSQ
ASSQ_ASSQ_26	has markedly unusual facial expression	Autism Spectrum Screening Questionnaire	ASSQ
ASSQ_ASSQ_27	has markedly unusual posture	Autism Spectrum Screening Questionnaire	ASSQ

Variable name in data	Item text	Instrument name	Instrument abbreviation (if applicable)
Barratt_financialsupport	Who is providing financial support for the child?	Barratt Simplified Measure of Social Status	BSMSS
Barratt_Barratt_P1_Edu	Parent 1 level of education	Barratt Simplified Measure of Social Status	BSMSS
Barratt_Barratt_Total_Edu	Education total score	Barratt Simplified Measure of Social Status	BSMSS
Barratt_Barratt_P1_Occ	Parent 1 level of occupation	Barratt Simplified Measure of Social Status	BSMSS
Barratt_Barratt_Total_Occ	Occupation total score	Barratt Simplified Measure of Social Status	BSMSS
Barratt_Barratt_Total	Barratt Total Score	Barratt Simplified Measure of Social Status	BSMSS
Basic_Demos_Sex	Sex	Basic Demographic Information	Basic_Demos
Basic_Demos_Study_Site	Study Site	Basic Demographic Information	Basic_Demos
CBCL_CBCL_01	1. Acts too young for his/her age	Child Behavior Checklist	CBCL
CBCL_CBCL_03	3. Argues a lot	Child Behavior Checklist	CBCL
CBCL_CBCL_04	4. Fails to finish things he/she starts	Child Behavior Checklist	CBCL
CBCL_CBCL_05	5. There is very little he/she enjoys	Child Behavior Checklist	CBCL
CBCL_CBCL_07	7. Bragging, boasting	Child Behavior Checklist	CBCL
CBCL_CBCL_08	8. Can't concentrate, can't pay attention for long	Child Behavior Checklist	CBCL
CBCL_CBCL_09	9. Can't get his/her mind off certain thoughts; obsessions	Child Behavior Checklist	CBCL
CBCL_CBCL_10	10. Can't sit still, restless or hyperactive	Child Behavior Checklist	CBCL
CBCL_CBCL_11	11. Clings to adults or too dependent	Child Behavior Checklist	CBCL
CBCL_CBCL_12	12. Complains of loneliness	Child Behavior Checklist	CBCL
CBCL_CBCL_13	13. Confused or seems to be in a fog	Child Behavior Checklist	CBCL
CBCL_CBCL_14	14. Cries a lot	Child Behavior Checklist	CBCL
CBCL_CBCL_16	16. Cruelty, bullying, or meanness to others	Child Behavior Checklist	CBCL
CBCL_CBCL_17	17. Daydreams or gets lost in his/her thoughts	Child Behavior Checklist	CBCL
CBCL_CBCL_19	19. Demands a lot of attention	Child Behavior Checklist	CBCL
CBCL_CBCL_20	20. Destroys his/her own things	Child Behavior Checklist	CBCL

Variable name in data	Item text	Instrument name	Instrument abbreviation (if applicable)
CBCL_CBCL_21	21. Destroys things belonging to his/her family or others	Child Behavior Checklist	CBCL
CBCL_CBCL_22	22. Disobedient at home	Child Behavior Checklist	CBCL
CBCL_CBCL_23	23. Disobedient at school	Child Behavior Checklist	CBCL
CBCL_CBCL_24	24. Doesn't eat well	Child Behavior Checklist	CBCL
CBCL_CBCL_25	25. Doesn't get along well with other kids	Child Behavior Checklist	CBCL
CBCL_CBCL_26	26. Doesn't seem to feel guilty after misbehaving	Child Behavior Checklist	CBCL
CBCL_CBCL_27	27. Easily jealous	Child Behavior Checklist	CBCL
CBCL_CBCL_28	28. Breaks rules at home, school, or elsewhere	Child Behavior Checklist	CBCL
CBCL_CBCL_29	29. Fears certain animals, situations, or places, other than school	Child Behavior Checklist	CBCL
CBCL_CBCL_30	30. Fears going to school	Child Behavior Checklist	CBCL
CBCL_CBCL_31	31. Fears he/she might think or do something bad	Child Behavior Checklist	CBCL
CBCL_CBCL_32	32. Feels he/she has to be perfect	Child Behavior Checklist	CBCL
CBCL_CBCL_33	33. Feels or complains that no one loves him/her	Child Behavior Checklist	CBCL
CBCL_CBCL_34	34. Feels others are out to get him/her	Child Behavior Checklist	CBCL
CBCL_CBCL_35	35. Feels worthless or inferior	Child Behavior Checklist	CBCL
CBCL_CBCL_36	36. Gets hurt a lot, accident-prone	Child Behavior Checklist	CBCL
CBCL_CBCL_37	37. Gets in many fights	Child Behavior Checklist	CBCL
CBCL_CBCL_38	38. Gets teased a lot	Child Behavior Checklist	CBCL
CBCL_CBCL_39	39. Hangs around with others who get in trouble	Child Behavior Checklist	CBCL
CBCL_CBCL_41	41. Impulsive or acts without thinking	Child Behavior Checklist	CBCL
CBCL_CBCL_42	42. Would rather be along than with others	Child Behavior Checklist	CBCL
CBCL_CBCL_43	43. Lying or cheating	Child Behavior Checklist	CBCL
CBCL_CBCL_44	44. Bites fingernails	Child Behavior Checklist	CBCL

Variable name in data	Item text	Instrument name	Instrument abbreviation (if applicable)
CBCL_CBCL_46	46. Nervous movements or twitching	Child Behavior Checklist	CBCL
CBCL_CBCL_47	47. Nightmares	Child Behavior Checklist	CBCL
CBCL_CBCL_48	48. Not liked by other kids	Child Behavior Checklist	CBCL
CBCL_CBCL_49	49. Constipated, doesn't move bowels	Child Behavior Checklist	CBCL
CBCL_CBCL_50	50. Too fearful or anxious	Child Behavior Checklist	CBCL
CBCL_CBCL_51	51. Feels dizzy or lightheaded	Child Behavior Checklist	CBCL
CBCL_CBCL_52	52. Feels too guilty	Child Behavior Checklist	CBCL
CBCL_CBCL_53	53. Overeating	Child Behavior Checklist	CBCL
CBCL_CBCL_54	54. Overtired without good reason	Child Behavior Checklist	CBCL
CBCL_CBCL_55	55. Overweight	Child Behavior Checklist	CBCL
CBCL_CBCL_56A	56A. Aches or pains (not stomach or headaches)	Child Behavior Checklist	CBCL
CBCL_CBCL_56B	56B. Headaches	Child Behavior Checklist	CBCL
CBCL_CBCL_56C	56C. Nausea, feels sick	Child Behavior Checklist	CBCL
CBCL_CBCL_56D	56D.A. Problems with eyes (not if corrected by glasses	Child Behavior Checklist	CBCL
CBCL_CBCL_56E	56E. Rashes or other skin problems	Child Behavior Checklist	CBCL
CBCL_CBCL_56F	56F. Stomachaches	Child Behavior Checklist	CBCL
CBCL_CBCL_56G	56G. Vomiting, throwing up	Child Behavior Checklist	CBCL
CBCL_CBCL_56H	56H.A. Other	Child Behavior Checklist	CBCL
CBCL_CBCL_57	57. Physically attacks people	Child Behavior Checklist	CBCL
CBCL_CBCL_61	61. Poor school work	Child Behavior Checklist	CBCL
CBCL_CBCL_62	62. Poorly coordinated or clumsy	Child Behavior Checklist	CBCL
CBCL_CBCL_63	63. Prefers being with older kids	Child Behavior Checklist	CBCL
CBCL_CBCL_64	64. Prefers being with younger kids	Child Behavior Checklist	CBCL
CBCL_CBCL_65	65. Refuses to talk	Child Behavior Checklist	CBCL
CBCL_CBCL_66	66. Repeats certain acts over and over; compulsions	Child Behavior Checklist	CBCL
CBCL_CBCL_68	68. Screams a lot	Child Behavior Checklist	CBCL

Variable name in data	Item text	Instrument name	Instrument abbreviation (if applicable)
CBCL_CBCL_69	69. Secretive, keeps things to self	Child Behavior Checklist	CBCL
CBCL_CBCL_71	71. Self-conscious or easily embarrassed	Child Behavior Checklist	CBCL
CBCL_CBCL_75	75. Too shy or timid	Child Behavior Checklist	CBCL
CBCL_CBCL_76	76. Sleeps less than most kids	Child Behavior Checklist	CBCL
CBCL_CBCL_77	77. Sleeps more than most kids during day and/or night	Child Behavior Checklist	CBCL
CBCL_CBCL_78	78. Inattentive or easily distracted	Child Behavior Checklist	CBCL
CBCL_CBCL_80	80. Stares blankly	Child Behavior Checklist	CBCL
CBCL_CBCL_81	81. Steals at home	Child Behavior Checklist	CBCL
CBCL_CBCL_83	83. Stores up too many things he/she doesn't need	Child Behavior Checklist	CBCL
CBCL_CBCL_84	84. Strange behavior	Child Behavior Checklist	CBCL
CBCL_CBCL_85	85. Strange ideas	Child Behavior Checklist	CBCL
CBCL_CBCL_86	86. Stubborn, sullen, or irritable	Child Behavior Checklist	CBCL
CBCL_CBCL_87	87. Sudden changes in mood or feelings	Child Behavior Checklist	CBCL
CBCL_CBCL_88	88. Sulks a lot	Child Behavior Checklist	CBCL
CBCL_CBCL_89	89. Suspicious	Child Behavior Checklist	CBCL
CBCL_CBCL_90	90. Swearing or obscene language	Child Behavior Checklist	CBCL
CBCL_CBCL_91	91. Talks about killing self	Child Behavior Checklist	CBCL
CBCL_CBCL_92	92. Talks or walks in sleep	Child Behavior Checklist	CBCL
CBCL_CBCL_94	94. Teases a lot	Child Behavior Checklist	CBCL
CBCL_CBCL_95	95. Temper tantrums or hot temper	Child Behavior Checklist	CBCL
CBCL_CBCL_97	97. Threatens people	Child Behavior Checklist	CBCL
CBCL_CBCL_100	100. Trouble sleeping	Child Behavior Checklist	CBCL
CBCL_CBCL_102	102. Underactive, slow moving, or lacks energy	Child Behavior Checklist	CBCL
CBCL_CBCL_103	103. Unhappy, sad, or depressed	Child Behavior Checklist	CBCL
CBCL_CBCL_108	108. Wets the bed	Child Behavior Checklist	CBCL
CBCL_CBCL_111	111. Withdrawn, doesn't get inolved with others	Child Behavior Checklist	CBCL

Variable name in data	Item text	Instrument name	Instrument abbreviation (if applicable)
CBCL_CBCL_112	112. Worries	Child Behavior Checklist	CBCL
CBCL_CBCL_AD	Anxious/Depressed Raw Score	Child Behavior Checklist	CBCL
CBCL_CBCL_AD_T	Anxious/Depressed T Score	Child Behavior Checklist	CBCL
CBCL_CBCL_WD	Withdrawn/Depressed Raw Score	Child Behavior Checklist	CBCL
CBCL_CBCL_WD_T	Withdrawn/Depressed T Score	Child Behavior Checklist	CBCL
CBCL_CBCL_SC	Somatic Complaints Raw Score	Child Behavior Checklist	CBCL
CBCL_CBCL_SC_T	Somatic Complaints T Score	Child Behavior Checklist	CBCL
CBCL_CBCL_SP	Social Problems Raw Score	Child Behavior Checklist	CBCL
CBCL_CBCL_SP_T	Social Problems T Score	Child Behavior Checklist	CBCL
CBCL_CBCL_TP	Thought Problems Raw Score	Child Behavior Checklist	CBCL
CBCL_CBCL_TP_T	Thought Problems T Score	Child Behavior Checklist	CBCL
CBCL_CBCL_AP	Attention Problems Raw Score	Child Behavior Checklist	CBCL
CBCL_CBCL_AP_T	Attention Problems T Score	Child Behavior Checklist	CBCL
CBCL_CBCL_RBB	Rule Breaking Behavior Raw Score	Child Behavior Checklist	CBCL
CBCL_CBCL_AB	Aggressive Behavior Raw Score	Child Behavior Checklist	CBCL
CBCL_CBCL_AB_T	Aggressive Behavior T Score	Child Behavior Checklist	CBCL
CBCL_CBCL_OP	Other Problems Raw Score	Child Behavior Checklist	CBCL
CBCL_CBCL_Int	Internalizing Raw Score	Child Behavior Checklist	CBCL
CBCL_CBCL_Int_T	Internalizing T Score	Child Behavior Checklist	CBCL
CBCL_CBCL_Ext	Externalizing Raw Score	Child Behavior Checklist	CBCL
CBCL_CBCL_Ext_T	Externalizing T Score	Child Behavior Checklist	CBCL
CBCL_CBCL_C	C Score Raw Score	Child Behavior Checklist	CBCL
CBCL_CBCL_Total	Total Raw Score	Child Behavior Checklist	CBCL
CBCL_CBCL_Total_T	Total T Score	Child Behavior Checklist	CBCL
CELF_CELF_Total	CELF-5 Total Score	Clinical Evaluation of Language Fundamentals, Fifth Edition	CELF-5
CELF_CELF_Criterion Score	CELF-5 Criterion Score	Clinical Evaluation of Language Fundamentals, Fifth Edition	CELF-5

Variable name in data	Item text	Instrument name	Instrument abbreviation (if applicable)
CELF_CELF_Exceed Cutoff	Meets criterion score?	Clinical Evaluation of Language Fundamentals, Fifth Edition	CELF-5
CTOPP_CTOPP_EL_R	Elision raw score	Comprehensive Test of Phonological Processing	CTOPP-2
CTOPP_CTOPP_EL_P	Elision percentile score	Comprehensive Test of Phonological Processing	CTOPP-2
CTOPP_CTOPP_EL_S	Elision scaled score	Comprehensive Test of Phonological Processing	CTOPP-2
CTOPP_CTOPP_BW_R	Blending Words raw score	Comprehensive Test of Phonological Processing	CTOPP-2
CTOPP_CTOPP_BW_P	Blending Words percentile score	Comprehensive Test of Phonological Processing	CTOPP-2
CTOPP_CTOPP_BW_S	Blending Words scaled score	Comprehensive Test of Phonological Processing	CTOPP-2
CTOPP_CTOPP_NR_R	Nonword Repetition raw score	Comprehensive Test of Phonological Processing	CTOPP-2
CTOPP_CTOPP_NR_S	Nonword Repetition scaled score	Comprehensive Test of Phonological Processing	CTOPP-2
CTOPP_CTOPP_RD_P	Rapid Digit Naming percentile score	Comprehensive Test of Phonological Processing	CTOPP-2
CTOPP_CTOPP_RD_S	Rapid Digit Naming scaled score	Comprehensive Test of Phonological Processing	CTOPP-2
CTOPP_CTOPP_RL_P	Rapid Letter Naming percentile score	Comprehensive Test of Phonological Processing	CTOPP-2
CTOPP_CTOPP_RL_S	Rapid Letter Naming scaled score	Comprehensive Test of Phonological Processing	CTOPP-2
CTOPP_CTOPP_RSN_Sum	Rapid Symbolic Naming (RD+RL) sum score	Comprehensive Test of Phonological Processing	CTOPP-2
CTOPP_CTOPP_RSN_P	Rapid Symbolic Naming (RD+RL) percentile score	Comprehensive Test of Phonological Processing	CTOPP-2
CTOPP_CTOPP_RSN_Comp	Rapid Symbolic Naming (RD+RL) composite score	Comprehensive Test of Phonological Processing	CTOPP-2
ColorVision_CV_Plate_04_R	Plate 04 Result	Ishihara Color Vision Test	ColorVision
ColorVision_CV_Plate_09_R	Plate 09 Result	Ishihara Color Vision Test	ColorVision
ColorVision_CV_Score	Color Vision Score	Ishihara Color Vision Test	ColorVision

Variable name in data	Item text	Instrument name	Instrument abbreviation (if applicable)
DTS_DTS_01	1. Feeling distressed or upset is unbearable to me.	Distress Tolerance Scale	DTS
DTS_DTS_02	2. When I feel distressed or upset, all I can think about is how bad I feel	Distress Tolerance Scale	DTS
DTS_DTS_03	3. I can’t handle feeling distressed or upset.	Distress Tolerance Scale	DTS
DTS_DTS_05	5. There’s nothing worse than feeling distressed or upset	Distress Tolerance Scale	DTS
DTS_DTS_06	6. I can tolerate being distressed or upset as well as most people	Distress Tolerance Scale	DTS
DTS_DTS_10	10. Being distressed or upset is always a major ordeal for me	Distress Tolerance Scale	DTS
DTS_DTS_11	11. I am ashamed of myself when I feel distressed or upset	Distress Tolerance Scale	DTS
DTS_DTS_12	12. My feelings of distress or being upset scare me.	Distress Tolerance Scale	DTS
DTS_DTS_absorption	Absorption subscale	Distress Tolerance Scale	DTS
DTS_DTS_regulation	Regulation subscale	Distress Tolerance Scale	DTS
DTS_DTS_tolerance	Tolerance subscale	Distress Tolerance Scale	DTS
DTS_DTS_Total	DTS Total Score	Distress Tolerance Scale	DTS
EHQ_EHQ_08	Using a Broom (upper hand)	Edinburgh Handedness Questionnaire	EHQ
EHQ_EHQ_11	Holding a Computer Mouse	Edinburgh Handedness Questionnaire	EHQ
FGC_FGC_CU	Curl up total	FitnessGram Child	FGC
FGC_FGC_CU_Zone	Curl up fitness zone	FitnessGram Child	FGC
FGC_FGC_TL	Trunk lift total	FitnessGram Child	FGC
FGC_FGC_TL_Zone	Trunk lift fitness zone	FitnessGram Child	FGC
FGC_FGC_PU_Zone	Push-up fitness zone	FitnessGram Child	FGC
FGC_FGC_SRL	Sit & Reach total (left side)	FitnessGram Child	FGC
FGC_FGC_SRL_Zone	Sit & Reach fitness zone (left side)	FitnessGram Child	FGC
FGC_FGC_SRR	Sit & Reach total (right side)	FitnessGram Child	FGC
FGC_FGC_SRR_Zone	Sit & Reach fitness zone (right side)	FitnessGram Child	FGC
ICU_P_ICU_P_01	1. Expresses his/her feelings openly.	Inventory of Callous-Unemotional Traits Parent	ICU

Variable name in data	Item text	Instrument name	Instrument abbreviation (if applicable)
ICU_P_ICU_P_02	2. Does not seem to know “right” from “wrong”.	Inventory of Callous-Unemotional Traits Parent	ICU
ICU_P_ICU_P_03	3. Is concerned about schoolwork.	Inventory of Callous-Unemotional Traits Parent	ICU
ICU_P_ICU_P_04	4. Does not care who he/she hurts to get what he/she wants.	Inventory of Callous-Unemotional Traits Parent	ICU
ICU_P_ICU_P_05	5. Feels bad or guilty when he/she has done something wrong.	Inventory of Callous-Unemotional Traits Parent	ICU
ICU_P_ICU_P_06	6. Does not show emotions.	Inventory of Callous-Unemotional Traits Parent	ICU
ICU_P_ICU_P_07	7. Does not care about being on time.	Inventory of Callous-Unemotional Traits Parent	ICU
ICU_P_ICU_P_08	8. Is concerned about the feelings of others.	Inventory of Callous-Unemotional Traits Parent	ICU
ICU_P_ICU_P_09	9. Does not care if he/she is in trouble.	Inventory of Callous-Unemotional Traits Parent	ICU
ICU_P_ICU_P_10	10. Does not let feelings control him/her.	Inventory of Callous-Unemotional Traits Parent	ICU
ICU_P_ICU_P_11	11. Does not care about doing things well.	Inventory of Callous-Unemotional Traits Parent	ICU
ICU_P_ICU_P_12	12. Seems very cold and uncaring.	Inventory of Callous-Unemotional Traits Parent	ICU
ICU_P_ICU_P_13	13. Easily admits to being wrong.	Inventory of Callous-Unemotional Traits Parent	ICU
ICU_P_ICU_P_14	14. It is easy to tell how he/she is feeling.	Inventory of Callous-Unemotional Traits Parent	ICU
ICU_P_ICU_P_15	15. Always tries his/her best.	Inventory of Callous-Unemotional Traits Parent	ICU
ICU_P_ICU_P_16	16. Apologizes (“says he/she is sorry”) to persons he/she has hurt.	Inventory of Callous-Unemotional Traits Parent	ICU
ICU_P_ICU_P_17	17. Tries not to hurt others’ feelings.	Inventory of Callous-Unemotional Traits Parent	ICU
ICU_P_ICU_P_18	18. Shows no remorse when he/she has done something wrong.	Inventory of Callous-Unemotional Traits Parent	ICU
ICU_P_ICU_P_19	19. Is very expressive and emotional.	Inventory of Callous-Unemotional Traits Parent	ICU

Variable name in data	Item text	Instrument name	Instrument abbreviation (if applicable)
ICU_P_ICU_P_20	20. Does not like to put the time into doing things well.	Inventory of Callous-Unemotional Traits Parent	ICU
ICU_P_ICU_P_21	21. The feelings of others are unimportant to him/her.	Inventory of Callous-Unemotional Traits Parent	ICU
ICU_P_ICU_P_22	22. Hides his/her feelings from others.	Inventory of Callous-Unemotional Traits Parent	ICU
ICU_P_ICU_P_23	23. Works hard on everything.	Inventory of Callous-Unemotional Traits Parent	ICU
ICU_P_ICU_P_24	24. Does things to make others feel good.	Inventory of Callous-Unemotional Traits Parent	ICU
ICU_P_ICU_P_Uncaring	Uncaring Subscale Score	Inventory of Callous-Unemotional Traits Parent	ICU
ICU_P_ICU_P_Unemotional	Unemotional Subscale Score	Inventory of Callous-Unemotional Traits Parent	ICU
ICU_P_ICU_P_Total	Total Score	Inventory of Callous-Unemotional Traits Parent	ICU
NLES_P_NLES_P_02a	2a. The child's close friend had serious troubles, problems, illness, or injury	Negative Life Events Scale	NLES
NLES_P_NLES_P_03a	3a. The child suffered from a serious physical illness, injury, or extreme pain (something that required rest of one week in bed, hospitalization, or surgery).	Negative Life Events Scale	NLES
NLES_P_NLES_P_06a	6a. People in the child's family (such as his/her parents, brothers or sisters) physically hit each other hard or hurt each other.	Negative Life Events Scale	NLES
NLES_P_NLES_P_07a	7a. The child's parent suffered from serious illness, injury, or extreme pain, something that required rest for one week in bed, hospitalization, or surgery.	Negative Life Events Scale	NLES
NLES_P_NLES_P_08a	8a. The child's mother or father talked about having serious money troubles (being worried	Negative Life Events Scale	NLES

Variable name in data	Item text	Instrument name	Instrument abbreviation (if applicable)
	about bills for ordinary things).		
NLES_P_NLES_P_09 a	9a. The child's relatives such as aunts, uncles, grandparents said bad things about his/her mother or father.	Negative Life Events Scale	NLES
NLES_P_NLES_P_10 a	10a. The child's mother or father fought or argued with his/her relatives such as aunts, uncles, grandparents	Negative Life Events Scale	NLES
NLES_P_NLES_P_11 a	11a. The child's mother or father acted badly in front of the child's friends (did things like yelled at them or criticized them).	Negative Life Events Scale	NLES
NLES_P_NLES_P_12 a	12a. The child's mother or father was intoxicated in the child's presence	Negative Life Events Scale	NLES
NLES_P_NLES_P_13 a	13a. The child's mother or father forgot to do important things for him/her that they promised they would do, such as take him/her on a trip, take him/her to nice places, or come to his/her school or athletic activities.	Negative Life Events Scale	NLES
NLES_P_NLES_P_14 a	14a. The child's mother or father was arrested or sent to jail.	Negative Life Events Scale	NLES
NLES_P_NLES_P_15 a	15a. The child's mother or father lost a job	Negative Life Events Scale	NLES
NLES_P_NLES_P_16 a	16a. A close family member to the child died such as a parent, close uncle, grandparent, or some other relative.	Negative Life Events Scale	NLES
NLES_P_NLES_P_18 a	18a. A close friend of the child moved away	Negative Life Events Scale	NLES
NLES_P_NLES_P_19 a	19a. The child's father acted very worried, upset, or sad, not because of something the child did.	Negative Life Events Scale	NLES
NLES_P_NLES_P_20 a	20a. The child's mother acted very	Negative Life Events Scale	NLES

Variable name in data	Item text	Instrument name	Instrument abbreviation (if applicable)
	worried, upset, or sad, not because of something the child did.		
NLES_P_NLES_P_21a	21a. The child changed schools.	Negative Life Events Scale	NLES
NLES_P_NLES_P_TotalEvents	Total # of Negative Events	Negative Life Events Scale	NLES
NLES_P_NLES_P_Aware	# of Negative Events the Child was aware of	Negative Life Events Scale	NLES
NLES_P_NLES_P_Upset_Total	Child's Total Upsetness/Negative Events	Negative Life Events Scale	NLES
NLES_P_NLES_P_Upset_Avg	Child's Average Upsetness/Negative Events	Negative Life Events Scale	NLES
PCIAT_PCIAT_01	1. How often does your child disobey time limits you set for online use?	Parent-Child Internet Addiction Test	PCIAT
PCIAT_PCIAT_02	2. How often does your child neglect household chores to spend more time online?	Parent-Child Internet Addiction Test	PCIAT
PCIAT_PCIAT_03	3. How often does your child prefer to spend time online rather than with the rest of your family?	Parent-Child Internet Addiction Test	PCIAT
PCIAT_PCIAT_04	4. How often does your child form new relationships with fellow online users?	Parent-Child Internet Addiction Test	PCIAT
PCIAT_PCIAT_05	5. How often do you complain about the amount of time your child spends online?	Parent-Child Internet Addiction Test	PCIAT
PCIAT_PCIAT_06	6. How often do your child's grades suffer because of the amount of time he or she spends online?	Parent-Child Internet Addiction Test	PCIAT
PCIAT_PCIAT_07	7. How often does your child check his or her e-mail before doing something else?	Parent-Child Internet Addiction Test	PCIAT
PCIAT_PCIAT_08	8. How often does your child seem withdrawn from others since discovering the Internet?	Parent-Child Internet Addiction Test	PCIAT
PCIAT_PCIAT_09	9. How often does your child become defensive or secretive	Parent-Child Internet Addiction Test	PCIAT

Variable name in data	Item text	Instrument name	Instrument abbreviation (if applicable)
	when asked what he or she does online?		
PCIAT_PCIAT_10	10. How often have you caught your child sneaking online against your wishes?	Parent-Child Internet Addiction Test	PCIAT
PCIAT_PCIAT_11	11. How often does your child spend time along in his or her room playing on the computer?	Parent-Child Internet Addiction Test	PCIAT
PCIAT_PCIAT_12	12. How often does your child receive strange phone calls from new "online" friends?	Parent-Child Internet Addiction Test	PCIAT
PCIAT_PCIAT_13	13. How often does your child snap, yell, or act annoyed if bothered while online?	Parent-Child Internet Addiction Test	PCIAT
PCIAT_PCIAT_14	14. How often does your child seem more tired and fatigued than he or she did before the Internet came along?	Parent-Child Internet Addiction Test	PCIAT
PCIAT_PCIAT_15	15. How often does your child seem preoccupied with being back online when off-line?	Parent-Child Internet Addiction Test	PCIAT
PCIAT_PCIAT_16	16. How often does your child throw tantrums with your interference about how long he or she spends online?	Parent-Child Internet Addiction Test	PCIAT
PCIAT_PCIAT_17	17. How often does your child choose to spend time online rather than doing once enjoyed hobbies and/or outside interests?	Parent-Child Internet Addiction Test	PCIAT
PCIAT_PCIAT_18	18. How often does your child become angry or belligerent when your place time limits on how much time he or shes is allowed to spend online?	Parent-Child Internet Addiction Test	PCIAT
PCIAT_PCIAT_19	19. How often does your child choose to spend more time online than going out with friends?	Parent-Child Internet Addiction Test	PCIAT

Variable name in data	Item text	Instrument name	Instrument abbreviation (if applicable)
PCIAT_PCIAT_20	20. How often does your child feel depressed, moody, or nervous when off-line which seems to go away once back online?	Parent-Child Internet Addiction Test	PCIAT
PCIAT_PCIAT_Total	Total Score	Parent-Child Internet Addiction Test	PCIAT
Pegboard_peg_drops_d	Dominant Hand - Number of drops	Grooved Pegboard	Pegboard
Pegboard_peg_time_d	Dominant Hand - Completion time (sec)	Grooved Pegboard	Pegboard
Pegboard_peg_z_d	Dominant Hand - z-score	Grooved Pegboard	Pegboard
Pegboard_peg_drops_nd	Non-dominant Hand - Number of drops	Grooved Pegboard	Pegboard
Pegboard_peg_time_nd	Non-dominant Hand - Completion time (sec)	Grooved Pegboard	Pegboard
Physical_Height	Height (in)	Physical Measures	Physical
Physical_Weight	Weight (lbs)	Physical Measures	Physical
Physical_HeartRate	Heart rate (beats/min)	Physical Measures	Physical
Physical_Diastolic_BP	Diastolic BP (mmHg)	Physical Measures	Physical
Physical_Systolic_BP	Systolic BP (mmHg)	Physical Measures	Physical
Physical_BMI	BMI (kg/m^2)	Physical Measures	Physical
PreInt_Demos_Fam_P1_Sex	Sex	Interview-Demographics/Family	Demog.Fam.
PreInt_Demos_Fam_P1_Age	Age	Interview-Demographics/Family	Demog.Fam.
PreInt_Demos_Fam_P1_RelQuality	How is the quality of the relationship between you and the child?	Interview-Demographics/Family	Demog.Fam.
PreInt_Demos_Fam_guardian_maritalstatus	Parent(s)/Guardian(s) Marital Status:	Interview-Demographics/Family	Demog.Fam.
PreInt_Demos_Fam_P2_LegalGuardian	Parent 2 has legal guardianship over the child	Interview-Demographics/Family	Demog.Fam.
PreInt_Demos_Home_living_01	Child lives with Both biological parents	Interview-Demographic and Household Information	Demog.Household.
PreInt_Demos_Home_living_03	Child lives with Biological mother	Interview-Demographic and Household Information	Demog.Household.
PreInt_Demos_Home_living_06	Child lives with Grandparents	Interview-Demographic and Household Information	Demog.Household.
PreInt_Demos_Home_living_17	Child lives with Other	Interview-Demographic and Household Information	Demog.Household.
PreInt_Demos_Home_fam_01_age	Person 1- age	Interview-Demographic and Household Information	Demog.Household.
PreInt_DevHx_preg_dur	Duration of pregnancy (weeks)	Interview-Developmental History	Dev.Hist.

Variable name in data	Item text	Instrument name	Instrument abbreviation (if applicable)
PreInt_DevHx_preg_symp_01	Spotting or vaginal bleeding	Interview-Developmental History	Dev.Hist.
PreInt_DevHx_preg_symp_02	Emotional problems	Interview-Developmental History	Dev.Hist.
PreInt_DevHx_preg_symp_04	Diabetes	Interview-Developmental History	Dev.Hist.
PreInt_DevHx_preg_symp_05	High blood pressure	Interview-Developmental History	Dev.Hist.
PreInt_DevHx_preg_symp_07	Took any prescription	Interview-Developmental History	Dev.Hist.
PreInt_DevHx_preg_symp_11	Swollen ankles	Interview-Developmental History	Dev.Hist.
PreInt_DevHx_preg_symp_12	Family stress	Interview-Developmental History	Dev.Hist.
PreInt_DevHx_m_birthage	Mother's age at birth of child	Interview-Developmental History	Dev.Hist.
PreInt_DevHx_delivery	Delivery:	Interview-Developmental History	Dev.Hist.
PreInt_DevHx_birthweight_lbs	Birth weight of child (lbs)	Interview-Developmental History	Dev.Hist.
PreInt_DevHx_complications	Compications at birth	Interview-Developmental History	Dev.Hist.
PreInt_DevHx_newborn_problems	Newborn period:	Interview-Developmental History	Dev.Hist.
PreInt_DevHx_temp_01	Easy to soothe when upset	Interview-Developmental History	Dev.Hist.
PreInt_DevHx_temp_02	Difficult to soothe when upset	Interview-Developmental History	Dev.Hist.
PreInt_DevHx_temp_03	Colic	Interview-Developmental History	Dev.Hist.
PreInt_DevHx_temp_04	Eating difficulties	Interview-Developmental History	Dev.Hist.
PreInt_DevHx_temp_05	Sleeping difficulties	Interview-Developmental History	Dev.Hist.
PreInt_DevHx_temp_07	Overly sensitive to sound	Interview-Developmental History	Dev.Hist.
PreInt_DevHx_temp_08	Baby was "limp" or stiff	Interview-Developmental History	Dev.Hist.
PreInt_DevHx_temp_09	Easily adaptable	Interview-Developmental History	Dev.Hist.
PreInt_DevHx_temp_10	Slow to warm up	Interview-Developmental History	Dev.Hist.
PreInt_DevHx_temp_11	Problems with social relatedness	Interview-Developmental History	Dev.Hist.
PreInt_DevHx_dev_normal	All developmental milestones within normal limits	Interview-Developmental History	Dev.Hist.
PreInt_DevHx_puberty	Has your child shown adult sexual body development (puberty)?	Interview-Developmental History	Dev.Hist.
PreInt_DevHx_growth_concerns	Have there been any concerns about your child's growth?	Interview-Developmental History	Dev.Hist.
PreInt_DevHx_lost_skills	Has your child lost any skills or abilities?	Interview-Developmental History	Dev.Hist.

Variable name in data	Item text	Instrument name	Instrument abbreviation (if applicable)
PreInt_EduHx_IEP	Does your child have an Individualized Education Plan (IEP)?	Interview- Education and Social History	Edu/Soc.Hist.
PreInt_EduHx_NeuroPsych	Has your child ever had any neuropsychological testing?	Interview- Education and Social History	Edu/Soc.Hist.
PreInt_EduHx_EI	Does your child have EI services?	Interview- Education and Social History	Edu/Soc.Hist.
PreInt_EduHx_CPSE	Does your child have CPSE services?	Interview- Education and Social History	Edu/Soc.Hist.
PreInt_EduHx_school_difficulty	Did your child experience any difficulty starting school?	Interview- Education and Social History	Edu/Soc.Hist.
PreInt_EduHx_learning_disability	Were any learning disabilities identified?	Interview- Education and Social History	Edu/Soc.Hist.
PreInt_EduHx_repeated_grades	Were any grades repeated?	Interview- Education and Social History	Edu/Soc.Hist.
PreInt_EduHx_tutor	Has your child had tutoring outside of school?	Interview- Education and Social History	Edu/Soc.Hist.
PreInt_EduHx_strength_english	English	Interview- Education and Social History	Edu/Soc.Hist.
PreInt_EduHx_strength_history	Social studies/history	Interview- Education and Social History	Edu/Soc.Hist.
PreInt_EduHx_strength_science	Science	Interview- Education and Social History	Edu/Soc.Hist.
PreInt_EduHx_strength_math	Math	Interview- Education and Social History	Edu/Soc.Hist.
PreInt_EduHx_strength_other	Other	Interview- Education and Social History	Edu/Soc.Hist.
PreInt_EduHx_weakness_english	English	Interview- Education and Social History	Edu/Soc.Hist.
PreInt_EduHx_weakness_history	Social studies/history	Interview- Education and Social History	Edu/Soc.Hist.
PreInt_EduHx_weakness_science	Science	Interview- Education and Social History	Edu/Soc.Hist.
PreInt_EduHx_weakness_math	Math	Interview- Education and Social History	Edu/Soc.Hist.
PreInt_EduHx_weakness_other	Other	Interview- Education and Social History	Edu/Soc.Hist.
PreInt_EduHx_detention	Detentions (past year)	Interview- Education and Social History	Edu/Soc.Hist.
PreInt_EduHx_suspension	Suspensions (past year)	Interview- Education and Social History	Edu/Soc.Hist.
PreInt_EduHx_recent_grades	Recent typical academic performance:	Interview- Education and Social History	Edu/Soc.Hist.
PreInt_EduHx_afterschoolteams	Does your child belong to any groups, sports teams, or organizations?	Interview- Education and Social History	Edu/Soc.Hist.
PreInt_EduHx_music	Music	Interview- Education and Social History	Edu/Soc.Hist.

Variable name in data	Item text	Instrument name	Instrument abbreviation (if applicable)
PreInt_EduHx_dance	Dance	Interview- Education and Social History	Edu/Soc.Hist.
PreInt_EduHx_school_sports	Sports	Interview- Education and Social History	Edu/Soc.Hist.
PreInt_EduHx_martial_arts	Martial Arts	Interview- Education and Social History	Edu/Soc.Hist.
PreInt_EduHx_afterschool_other	Other	Interview- Education and Social History	Edu/Soc.Hist.
PreInt_EduHx_bestfriend	Does your child have a best friend?	Interview- Education and Social History	Edu/Soc.Hist.
PreInt_EduHx_getalongkids_school	How does your child get along with other children at school?	Interview- Education and Social History	Edu/Soc.Hist.
PreInt_EduHx_videocomputergames	Playing video/computer games	Interview- Education and Social History	Edu/Soc.Hist.
PreInt_EduHx_reading	Books/reading	Interview- Education and Social History	Edu/Soc.Hist.
PreInt_EduHx_crafts	Drawing/painting/crafts	Interview- Education and Social History	Edu/Soc.Hist.
PreInt_EduHx_imaginativeplay	Imaginative play	Interview- Education and Social History	Edu/Soc.Hist.
PreInt_EduHx_sports	Playing sports	Interview- Education and Social History	Edu/Soc.Hist.
PreInt_EduHx_homework	Homework	Interview- Education and Social History	Edu/Soc.Hist.
PreInt_EduHx_dancing	Dancing	Interview- Education and Social History	Edu/Soc.Hist.
PreInt_EduHx_family_religious	Was your child raised in a particular religious faith?	Interview- Education and Social History	Edu/Soc.Hist.
PreInt_EduHx_current_religious	Is he/she religious now?	Interview- Education and Social History	Edu/Soc.Hist.
PreInt_FamHx_RDC_omoves1	Number of times changed address (age 0-5):	Family History/Research Diagnostic Criteria	Fam.Med.Hist
PreInt_FamHx_RDC_fmdk	Don't know father's mother's current age	Family History/Research Diagnostic Criteria	Fam.Med.Hist
PreInt_FamHx_RDC_ffdk	Don't know father's father's current age	Family History/Research Diagnostic Criteria	Fam.Med.Hist
PreInt_FamHx_RDC_fmmdk	Don't know father's maternal grandmother's current age	Family History/Research Diagnostic Criteria	Fam.Med.Hist
PreInt_FamHx_RDC_fmfdk	Don't know father's maternal grandfather's current age	Family History/Research Diagnostic Criteria	Fam.Med.Hist
PreInt_FamHx_RDC_ffmfdk	Don't know father's paternal grandmother's current age	Family History/Research Diagnostic Criteria	Fam.Med.Hist
PreInt_FamHx_RDC_fffdk	Don't know father's paternal grandfather's current age	Family History/Research Diagnostic Criteria	Fam.Med.Hist

Variable name in data	Item text	Instrument name	Instrument abbreviation (if applicable)
PreInt_FamHx_RDC_mfdk	Don't know mother's mother's current age	Family History/Research Diagnostic Criteria	Fam.Med.Hist
PreInt_FamHx_RDC_mfmdk	Don't know mother's maternal grandmother's current age	Family History/Research Diagnostic Criteria	Fam.Med.Hist
PreInt_FamHx_RDC_mmmdk	Don't know mother's maternal grandmother's current age	Family History/Research Diagnostic Criteria	Fam.Med.Hist
PreInt_FamHx_RDC_mmfdk	Don't know mother's paternal grandmother's current age	Family History/Research Diagnostic Criteria	Fam.Med.Hist
PreInt_FamHx_RDC_mffdk	Mother's paternal grandmother current age range, or age range at death:	Family History/Research Diagnostic Criteria	Fam.Med.Hist
PreInt_TxHx_Past_DX	Has your chid ever been diagnosed with a psychiatric or learning disorder?	Interview- Treatment History	Treat.hist.
SCARED_P_SCARED_P_01	1. When my child feels frightened, it is hard to breathe	Screen for Child Anxiety Related Disorders	SCARED
SCARED_P_SCARED_P_02	2. My child gets headaches when he/she is at school	Screen for Child Anxiety Related Disorders	SCARED
SCARED_P_SCARED_P_03	3. My child doesn’t like to be with people he/she does't know well	Screen for Child Anxiety Related Disorders	SCARED
SCARED_P_SCARED_P_04	4. My child gets scared if he/she sleeps away from home	Screen for Child Anxiety Related Disorders	SCARED
SCARED_P_SCARED_P_05	5. My child worries about other people liking him/her	Screen for Child Anxiety Related Disorders	SCARED
SCARED_P_SCARED_P_08	8. My child follows me wherever I go	Screen for Child Anxiety Related Disorders	SCARED
SCARED_P_SCARED_P_09	9. People tell me that my child looks nervous	Screen for Child Anxiety Related Disorders	SCARED
SCARED_P_SCARED_P_11	11. My child gets stomachaches at school	Screen for Child Anxiety Related Disorders	SCARED
SCARED_P_SCARED_P_12	12. When my child gets frightened, he/she feels like he/she is going crazy	Screen for Child Anxiety Related Disorders	SCARED
SCARED_P_SCARED_P_16	16. My child has nightmares about something bad	Screen for Child Anxiety Related Disorders	SCARED

Variable name in data	Item text	Instrument name	Instrument abbreviation (if applicable)
	happening to his/her parents		
SCARED_P_SCARED_P_17	17. My child worries about going to school	Screen for Child Anxiety Related Disorders	SCARED
SCARED_P_SCARED_P_19	19. He/she child gets shaky	Screen for Child Anxiety Related Disorders	SCARED
SCARED_P_SCARED_P_20	20. My child has nightmares about something bad happening to him/her	Screen for Child Anxiety Related Disorders	SCARED
SCARED_P_SCARED_P_22	22. When my child gets frightened, he/she sweats a lot	Screen for Child Anxiety Related Disorders	SCARED
SCARED_P_SCARED_P_24	24. My child gets really frightened for no reason at all	Screen for Child Anxiety Related Disorders	SCARED
SCARED_P_SCARED_P_28	28. People tell me that my child worries too much	Screen for Child Anxiety Related Disorders	SCARED
SCARED_P_SCARED_P_30	30. My child is afraid of having anxiety (or panic) attacks	Screen for Child Anxiety Related Disorders	SCARED
SCARED_P_SCARED_P_31	31. My child worries that something bad will happen to his/her parents	Screen for Child Anxiety Related Disorders	SCARED
SCARED_P_SCARED_P_32	32. My child feels shy with people he/she doesn't know well	Screen for Child Anxiety Related Disorders	SCARED
SCARED_P_SCARED_P_34	34. When my child gets frightened, he/she feels like throwing up	Screen for Child Anxiety Related Disorders	SCARED
SCARED_P_SCARED_P_35	35. My child worries about how well he/she does things	Screen for Child Anxiety Related Disorders	SCARED
SCARED_P_SCARED_P_36	36. My child is scared to go to school	Screen for Child Anxiety Related Disorders	SCARED
SCARED_P_SCARED_P_37	37. My child worries about things that have already happened	Screen for Child Anxiety Related Disorders	SCARED
SCARED_P_SCARED_P_GD	Generalized Anxiety Disorder Score	Screen for Child Anxiety Related Disorders	SCARED
SCARED_P_SCARED_P_SC	Social Anxiety Disorder Score	Screen for Child Anxiety Related Disorders	SCARED
SCARED_P_SCARED_P_SH	Significant School Avoidance Score	Screen for Child Anxiety Related Disorders	SCARED
SCARED_P_SCARED_P_SP	Separation Anxiety SOC Score	Screen for Child Anxiety Related Disorders	SCARED

Variable name in data	Item text	Instrument name	Instrument abbreviation (if applicable)
SCARED_P_SCARED_P_Total	Total Score	Screen for Child Anxiety Related Disorders	SCARED
SCQ_SCQ_02	2. Do you have a to and fro "conversation" with her/him that involves taking turns or building on what you have said?	Social Communication Questionnaire	SCQ
SCQ_SCQ_03	3. Does she/he ever use odd phrases or say the same thing over and over in almost exactly the same way (either phrases that she/he hears other people use or ones that she/he makes up?	Social Communication Questionnaire	SCQ
SCQ_SCQ_04	4. Does she/he ever use socially inappropriate questions or statements? For example, does she/he ever regularly ask personal questions or make personal comments at awkward times?	Social Communication Questionnaire	SCQ
SCQ_SCQ_05	5. Does she/he ever get her/his pronouns mixed up (e.g., saying you or she/he for I)?	Social Communication Questionnaire	SCQ
SCQ_SCQ_06	6. Does she/he ever use words that she/he seems to have invented or made up her/himself; put things in odd, indirect ways; or use metaphorical ways of saying things (e.g., saying hot rain for steam)?	Social Communication Questionnaire	SCQ
SCQ_SCQ_07	7. Does she/he ever say the same thing over and over again?	Social Communication Questionnaire	SCQ
SCQ_SCQ_08	8. Does she/he ever have things that she/he seems to have to do in a very particular way or order or rituals that she/he insists that you go though?	Social Communication Questionnaire	SCQ
SCQ_SCQ_09	9. Does her/his facial expression usually	Social Communication Questionnaire	SCQ

Variable name in data	Item text	Instrument name	Instrument abbreviation (if applicable)
	seem appropriate to the particular situation, as far as you can tell?		
SCQ_SCQ_10	10. Does she/he ever use your hand like a tool or as if it were part of her/his own body (e.g., pointing with your finder or putting your hand on a doorknob to get you to open the door?)	Social Communication Questionnaire	SCQ
SCQ_SCQ_11	11. Does she/he ever have any interests that preoccupy her/him and might seem off to other people (e.g., traffic lights, drainpipes, or timetables?)	Social Communication Questionnaire	SCQ
SCQ_SCQ_12	12. Does she/he ever seem to be more interested in parts of a toy or an object (e.g., spinning the wheels of a car), rather than in using the object as it was intended?	Social Communication Questionnaire	SCQ
SCQ_SCQ_13	13. Does she/he ever have any special interests that are unusual in their intensity but otherwise appropriate for her/his age and peer group (e.g., trains or dinosaurs)?	Social Communication Questionnaire	SCQ
SCQ_SCQ_14	14. Does she/he ever seem to be unusually interested in the sight, feel, sound, taske, or smell of things or people?	Social Communication Questionnaire	SCQ
SCQ_SCQ_15	15. Does she/he ever have any mannerisms or odd ways of moving her/his hands or fingers, such as flapping or moving her/his fingers in front or her/his eyes?	Social Communication Questionnaire	SCQ
SCQ_SCQ_16	16. Does she/he ever have any complicated movements of her/his whole body, such as spinning or repeatedly	Social Communication Questionnaire	SCQ

Variable name in data	Item text	Instrument name	Instrument abbreviation (if applicable)
	bouncing up and down?		
SCQ_SCQ_17	17. Does she/he ever injure her/himself deliberately, such as by biting her/his arm or banging her/his head?	Social Communication Questionnaire	SCQ
SCQ_SCQ_18	18. Does she/he ever have any objects (other than a soft toy or comfort blanket) that she/he has to carry around?	Social Communication Questionnaire	SCQ
SCQ_SCQ_19	19. Does she/he have any particular friends or a best friend?	Social Communication Questionnaire	SCQ
SCQ_SCQ_20	20. Does she/he ever talk with you just to be friendly (rather than to get something)?	Social Communication Questionnaire	SCQ
SCQ_SCQ_21	21. Does she/he ever spontaneously copy you (or other people) or what you are doing (such as vacuuming, gardening, or mending things)?	Social Communication Questionnaire	SCQ
SCQ_SCQ_22	22. Does she/he ever spontaneously point at things around her/him just to show you things (not because she/he wants them)?	Social Communication Questionnaire	SCQ
SCQ_SCQ_23	23. Does she/he ever use gestures, other than pointing or pulling your hand, to let you know what she/he wants?	Social Communication Questionnaire	SCQ
SCQ_SCQ_24	24. Does she/he nod her/his head to indicate yes?	Social Communication Questionnaire	SCQ
SCQ_SCQ_25	25. Does she/he shake her/his head to indicate no?	Social Communication Questionnaire	SCQ
SCQ_SCQ_26	26. Does she/he usually look at you directly in the face when doing things with you or talking with you?	Social Communication Questionnaire	SCQ
SCQ_SCQ_27	27. Does she/he smile back if someone smiles at her/him?	Social Communication Questionnaire	SCQ
SCQ_SCQ_29	29. Does she/he ever offer to share things	Social Communication Questionnaire	SCQ

Variable name in data	Item text	Instrument name	Instrument abbreviation (if applicable)
	other than food with you?		
SCQ_SCQ_30	30. Does she/he ever seem to want you to join in her/his enjoyment of something?	Social Communication Questionnaire	SCQ
SCQ_SCQ_31	31. Does she/he ever try to comfort you if you are sad or hurt?	Social Communication Questionnaire	SCQ
SCQ_SCQ_32	32. If she/he wants something or wants help, does she/he look at you and use gestures with sounds or words to get your attention?	Social Communication Questionnaire	SCQ
SCQ_SCQ_33	33. Does she/he show a normal range of facial expressions?	Social Communication Questionnaire	SCQ
SCQ_SCQ_34	34. Does she/he ever spontaenously join in and try to copy the actions in social games, such as The Mulberry Bush or London Bridge is Falling Down?	Social Communication Questionnaire	SCQ
SCQ_SCQ_35	35. Does she/he play any pretend or make-believe games?	Social Communication Questionnaire	SCQ
SCQ_SCQ_36	36. Does she/he seem interested in other children of approximately the same age whom she/he does not know?	Social Communication Questionnaire	SCQ
SCQ_SCQ_37	37. Does she/he respond positively when another child approaches her/him?	Social Communication Questionnaire	SCQ
SCQ_SCQ_38	38. If you come into a room and start talkking to her/him without calling her/his name, does she/he usually look up and pay attention to you?	Social Communication Questionnaire	SCQ
SCQ_SCQ_39	39. Does she/he ever play imaginative games with another child in such a way that you can tell that each child understands what the other is pretending?	Social Communication Questionnaire	SCQ

Variable name in data	Item text	Instrument name	Instrument abbreviation (if applicable)
SCQ_SCQ_40	40. Does she/he play cooperatively in games that need some form of joining in with a group of other children, such as hide-and-seek or ball games?	Social Communication Questionnaire	SCQ
SCQ_SCQ_Total	Total Score	Social Communication Questionnaire	SCQ
SDQ_SDQ_01	Considerate of other people's feelings	Strength and Difficulties Questionnaire	SDQ
SDQ_SDQ_02	Restless, overactive, cannot stay still for long	Strength and Difficulties Questionnaire	SDQ
SDQ_SDQ_03	Often complains of headaches, stomach-aches or sickness	Strength and Difficulties Questionnaire	SDQ
SDQ_SDQ_04	Shares readily with other children, for example toys, treats, pencils (for 11-17 year olds: Shares readily with other youth, for example CD's, games, food)	Strength and Difficulties Questionnaire	SDQ
SDQ_SDQ_05	Often loses temper	Strength and Difficulties Questionnaire	SDQ
SDQ_SDQ_06	Rather solitary, prefers to play alone (for 11-17 year olds: Would rather be alone than with other youth)	Strength and Difficulties Questionnaire	SDQ
SDQ_SDQ_07	Generally well behaved, usually does what adults request	Strength and Difficulties Questionnaire	SDQ
SDQ_SDQ_08	Many worries or often seems worried	Strength and Difficulties Questionnaire	SDQ
SDQ_SDQ_09	Helpful if someone is hurt, upset or feeling ill	Strength and Difficulties Questionnaire	SDQ
SDQ_SDQ_10	Constantly fidgeting or squirming	Strength and Difficulties Questionnaire	SDQ
SDQ_SDQ_11	Has at least one good friend	Strength and Difficulties Questionnaire	SDQ
SDQ_SDQ_12	Often fights with other children or bullies them (for 11-17 year olds: Often fights with other youth or bullies them)	Strength and Difficulties Questionnaire	SDQ

Variable name in data	Item text	Instrument name	Instrument abbreviation (if applicable)
SDQ_SDQ_13	Often unhappy, depressed or tearful	Strength and Difficulties Questionnaire	SDQ
SDQ_SDQ_14	Generally liked by other children (for 11-17 year olds: Generally liked by other youth)	Strength and Difficulties Questionnaire	SDQ
SDQ_SDQ_15	Easily distracted, concentration wanders	Strength and Difficulties Questionnaire	SDQ
SDQ_SDQ_16	Nervous in new situations, easily loses confidence	Strength and Difficulties Questionnaire	SDQ
SDQ_SDQ_17	Kind to younger children	Strength and Difficulties Questionnaire	SDQ
SDQ_SDQ_18	Often lies or cheats	Strength and Difficulties Questionnaire	SDQ
SDQ_SDQ_19	Picked on or bullied by other children (for 11-17 year olds: Picked on or bullied by other youth)	Strength and Difficulties Questionnaire	SDQ
SDQ_SDQ_20	Often offers to help others (parents, teachers, children)	Strength and Difficulties Questionnaire	SDQ
SDQ_SDQ_21	Thinks things out before acting	Strength and Difficulties Questionnaire	SDQ
SDQ_SDQ_22	Steals from home, school or elsewhere	Strength and Difficulties Questionnaire	SDQ
SDQ_SDQ_24	Many fears, easily scared	Strength and Difficulties Questionnaire	SDQ
SDQ_SDQ_25	Good attention span, sees chores or homework through to the end	Strength and Difficulties Questionnaire	SDQ
SDQ_SDQ_26	Overall, do you think that your child has difficulties in one or more of the following areas: emotions, concentration, behavior or being able to get on with other people?	Strength and Difficulties Questionnaire	SDQ
SDQ_SDQ_Conduct_Problems	Conduct problems scale	Strength and Difficulties Questionnaire	SDQ
SDQ_SDQ_Difficulties_Total	Total Difficulties Score	Strength and Difficulties Questionnaire	SDQ

Variable name in data	Item text	Instrument name	Instrument abbreviation (if applicable)
SDQ_SDQ_Emotional_Problems	Emotional Problems Scale	Strength and Difficulties Questionnaire	SDQ
SDQ_SDQ_Externalizing	Externalizing Score	Strength and Difficulties Questionnaire	SDQ
SDQ_SDQ_Generating_Impact	Generating Impact Scores	Strength and Difficulties Questionnaire	SDQ
SDQ_SDQ_Hyperactivity	Hyperactivity Scale	Strength and Difficulties Questionnaire	SDQ
SDQ_SDQ_Internalizing	Internalizing Score	Strength and Difficulties Questionnaire	SDQ
SDQ_SDQ_Peer_Problems	Peer Problems Scale	Strength and Difficulties Questionnaire	SDQ
SDQ_SDQ_Prosocial	Prosocial Scale	Strength and Difficulties Questionnaire	SDQ
SRS_SRS_01	1. Seems much more fidgety in social situations than when alone.	Social Responsiveness Scale	SRS
SRS_SRS_02	2. Expressions on his or her face don't match what he or she is saying.	Social Responsiveness Scale	SRS
SRS_SRS_03	3. Seems self-confident when interacting with others.	Social Responsiveness Scale	SRS
SRS_SRS_04	4. When under stress, he or she shows rigid or inflexible patterns of behavior that seem odd.	Social Responsiveness Scale	SRS
SRS_SRS_05	5. Doesn't recognize when others are trying to take advantage of him or her.	Social Responsiveness Scale	SRS
SRS_SRS_06	6. Would rather be alone than with others.	Social Responsiveness Scale	SRS
SRS_SRS_07	7. Is aware of what others are thinking or feeling.	Social Responsiveness Scale	SRS
SRS_SRS_08	8. Behaves in ways that seem strange or bizarre.	Social Responsiveness Scale	SRS
SRS_SRS_09	9. Clings to adults, seems too dependent on them.	Social Responsiveness Scale	SRS
SRS_SRS_10	10. Takes things too literally and doesn't get the real meaning of a conversation.	Social Responsiveness Scale	SRS

Variable name in data	Item text	Instrument name	Instrument abbreviation (if applicable)
SRS_SRS_11	11. Has good self-confidence.	Social Responsiveness Scale	SRS
SRS_SRS_12	12. Is able to communicate his or her feelings to others.	Social Responsiveness Scale	SRS
SRS_SRS_13	13. Is awkward is turn-taking interactions with peers (e.g., doesn't seem to understand the give-and-take of conversations)	Social Responsiveness Scale	SRS
SRS_SRS_14	14. Is not well coordinated.	Social Responsiveness Scale	SRS
SRS_SRS_15	15. Is able to understand the meaning of other people's tone of voice and facial expressions.	Social Responsiveness Scale	SRS
SRS_SRS_16	16. Avoids eye contact or has unusual eye contact.	Social Responsiveness Scale	SRS
SRS_SRS_17	17. Recognizes when something is unfair.	Social Responsiveness Scale	SRS
SRS_SRS_18	18. Has difficulty making friends, even when trying his or her best.	Social Responsiveness Scale	SRS
SRS_SRS_19	19. Gets frustrated trying to get ideas across in conversations.	Social Responsiveness Scale	SRS
SRS_SRS_20	20. Shows unusual sensory interests (e.g., mouthing or spinning objects) or strange ways of playing with toys	Social Responsiveness Scale	SRS
SRS_SRS_21	21. Is able to imitate others' actions.	Social Responsiveness Scale	SRS
SRS_SRS_22	22. Plays appropriately with children his or her age.	Social Responsiveness Scale	SRS
SRS_SRS_23	23. Does not join group activities unless told to do so.	Social Responsiveness Scale	SRS
SRS_SRS_24	24. Has more difficulty than other children with changes in his or her routine.	Social Responsiveness Scale	SRS
SRS_SRS_25	25. Doesn't seem to mind being out of step with or "not on the same wavelength" as others.	Social Responsiveness Scale	SRS

Variable name in data	Item text	Instrument name	Instrument abbreviation (if applicable)
SRS_SRS_26	26. Offers comfort to others when they are sad.	Social Responsiveness Scale	SRS
SRS_SRS_27	27. Avoids starting social interactions with peers or adults.	Social Responsiveness Scale	SRS
SRS_SRS_28	28. Thinks or talks about the same thing over and over.	Social Responsiveness Scale	SRS
SRS_SRS_29	29. Is regarded by other children as odd or weird.	Social Responsiveness Scale	SRS
SRS_SRS_30	30. Becomes upset in a situation with lots of things going on.	Social Responsiveness Scale	SRS
SRS_SRS_31	31. Can't get his or her mind off something once he or she starts thinking about it.	Social Responsiveness Scale	SRS
SRS_SRS_32	32. Has good personal hygiene.	Social Responsiveness Scale	SRS
SRS_SRS_33	33. Is socially awkward, even when he or she is trying to be polite.	Social Responsiveness Scale	SRS
SRS_SRS_34	34. Avoids people who want to be emotionally close to him or her.	Social Responsiveness Scale	SRS
SRS_SRS_35	35. Has trouble keeping up with the flow of a normal conversation.	Social Responsiveness Scale	SRS
SRS_SRS_36	36. Has difficulty relating to adults.	Social Responsiveness Scale	SRS
SRS_SRS_37	37. Has difficulty relating to peers.	Social Responsiveness Scale	SRS
SRS_SRS_38	38. Responds appropriately to mood changes in others (e.g., when a friend's or playmate's mood changes from happy to sad).	Social Responsiveness Scale	SRS
SRS_SRS_39	39. Has an unusally narrow range of interests.	Social Responsiveness Scale	SRS
SRS_SRS_40	40. Is imaginative, good at pretending (without losing touch with reality).	Social Responsiveness Scale	SRS
SRS_SRS_41	41. Wanders aimlessly from one activity to another.	Social Responsiveness Scale	SRS
SRS_SRS_42	42. Seems overly sensitive to sounds, textures, or smells.	Social Responsiveness Scale	SRS

Variable name in data	Item text	Instrument name	Instrument abbreviation (if applicable)
SRS_SRS_43	43. Separates easily from caregivers.	Social Responsiveness Scale	SRS
SRS_SRS_44	44. Doesn't understand how events relate to one another (cause and effect) the way other children his or her age do.	Social Responsiveness Scale	SRS
SRS_SRS_45	45. Focuses his or her attention to where others are looking or listening.	Social Responsiveness Scale	SRS
SRS_SRS_47	47. Is too silly or laughs inappropriately.	Social Responsiveness Scale	SRS
SRS_SRS_48	48. Has a sense of humor, understands jokes.	Social Responsiveness Scale	SRS
SRS_SRS_49	49. Does extremely well at a few tasks, but does not do as well at most other tasks.	Social Responsiveness Scale	SRS
SRS_SRS_50	50. Has repetitive, odd behaviors such as hand flapping or rocking.	Social Responsiveness Scale	SRS
SRS_SRS_51	51. Has difficulty answering questions directly and ends up talking around the subject.	Social Responsiveness Scale	SRS
SRS_SRS_52	52. Knows when he or she is talking too loud or making too much noise.	Social Responsiveness Scale	SRS
SRS_SRS_53	53. Talks to people with an unusual tone of voice (e.g., talks like a robot or like he or she is giving a lecture).	Social Responsiveness Scale	SRS
SRS_SRS_54	54. Seems to react to people as if they are objects.	Social Responsiveness Scale	SRS
SRS_SRS_55	55. Knows when he or she is talking too loud or making too much noise.	Social Responsiveness Scale	SRS
SRS_SRS_56	56. Walks in between two people who are talking.	Social Responsiveness Scale	SRS
SRS_SRS_57	57. Gets teased a lot.	Social Responsiveness Scale	SRS
SRS_SRS_58	58. Concentrates too much on parts of things rather than	Social Responsiveness Scale	SRS

Variable name in data	Item text	Instrument name	Instrument abbreviation (if applicable)
	seeing the whole picture. For example, if asked to describe what happened in a story, he or she may talk only about the kind of clothes the characters were wearing.		
SRS_SRS_59	59. Is overly suspicious.	Social Responsiveness Scale	SRS
SRS_SRS_60	60. Is emotionally distant, doesn't show his or her feelings.	Social Responsiveness Scale	SRS
SRS_SRS_61	61. Is inflexible, has a hard time changing his or her mind.	Social Responsiveness Scale	SRS
SRS_SRS_62	62. Give unusual or illogical reasons for doing things.	Social Responsiveness Scale	SRS
SRS_SRS_63	63. Touches others in an unusual way (e.g., he or she may touch someone just to make contact and then walk away without saying anything).	Social Responsiveness Scale	SRS
SRS_SRS_64	64. Is too tense in social settings.	Social Responsiveness Scale	SRS
SRS_SRS_65	65. Stares or gazes off into space.	Social Responsiveness Scale	SRS
SRS_SRS_AWR	Social Awareness Raw Score	Social Responsiveness Scale	SRS
SRS_SRS_AWR_T	Social Awareness T-Score	Social Responsiveness Scale	SRS
SRS_SRS_COG	Social Cognition Raw Score	Social Responsiveness Scale	SRS
SRS_SRS_COG_T	Social Cognition T-Score	Social Responsiveness Scale	SRS
SRS_SRS_COM	Social Communication Raw Score	Social Responsiveness Scale	SRS
SRS_SRS_COM_T	Social Communication T-Score	Social Responsiveness Scale	SRS
SRS_SRS_DSMRRB	Restricted Interests and Repetitive Behavior Raw Score	Social Responsiveness Scale	SRS
SRS_SRS_DSMRRB_T	Restricted Interests and Repetitive Behavior T-Score	Social Responsiveness Scale	SRS
SRS_SRS_MOT	Social Motivation Raw Score	Social Responsiveness Scale	SRS
SRS_SRS_MOT_T	Social Motivation T-Score	Social Responsiveness Scale	SRS
SRS_SRS_RRB	Restricted Interests and Repetitive Behavior Raw Score	Social Responsiveness Scale	SRS

Variable name in data	Item text	Instrument name	Instrument abbreviation (if applicable)
SRS_SRS_RRB_T	Restricted Interests and Repetitive Behavior T-Score	Social Responsiveness Scale	SRS
SRS_SRS_SCI	Social Communication and Interaction Raw Score	Social Responsiveness Scale	SRS
SRS_SRS_SCI_T	Social Communication and Interaction T-Score	Social Responsiveness Scale	SRS
SRS_SRS_Total	Total Raw Score	Social Responsiveness Scale	SRS
SRS_SRS_Total_T	Total T-Score	Social Responsiveness Scale	SRS
SWAN_SWAN_01	1. Gives close attention to detail and avoids careless mistakes	The Strengths and Weaknesses Assessment of Normal Behavior	SWAN
SWAN_SWAN_02	2. Sustains attention on tasks or play activities	The Strengths and Weaknesses Assessment of Normal Behavior	SWAN
SWAN_SWAN_03	3. Listens when spoken to directly	The Strengths and Weaknesses Assessment of Normal Behavior	SWAN
SWAN_SWAN_04	4. Follows through on instructions and finishes school work and chores	The Strengths and Weaknesses Assessment of Normal Behavior	SWAN
SWAN_SWAN_05	5. Organizes tasks and activities	The Strengths and Weaknesses Assessment of Normal Behavior	SWAN
SWAN_SWAN_06	6. Engages in tasks that require sustained mental effort	The Strengths and Weaknesses Assessment of Normal Behavior	SWAN
SWAN_SWAN_07	7. Keeps track of things necessary for activities (doesn't lose them)	The Strengths and Weaknesses Assessment of Normal Behavior	SWAN
SWAN_SWAN_08	8. Ignores extraneous stimuli	The Strengths and Weaknesses Assessment of Normal Behavior	SWAN
SWAN_SWAN_09	9. Remembers daily activities	The Strengths and Weaknesses Assessment of Normal Behavior	SWAN
SWAN_SWAN_10	10. Sits still (controls movement of hands or feet or controls squirming)	The Strengths and Weaknesses Assessment of Normal Behavior	SWAN
SWAN_SWAN_11	11. Stays seated (when required by	The Strengths and Weaknesses	SWAN

Variable name in data	Item text	Instrument name	Instrument abbreviation (if applicable)
	class rules or social conventions)	Assessment of Normal Behavior	
SWAN_SWAN_12	12. Modulates motor activity (inhibits inappropriate running or climbing)	The Strengths and Weaknesses Assessment of Normal Behavior	SWAN
SWAN_SWAN_13	13. Plays quietly (keeps noise level reasonable)	The Strengths and Weaknesses Assessment of Normal Behavior	SWAN
SWAN_SWAN_14	14. Settles down and rests (controls excessive talking)	The Strengths and Weaknesses Assessment of Normal Behavior	SWAN
SWAN_SWAN_15	15. Modulates verbal activity (controls excessive talking)	The Strengths and Weaknesses Assessment of Normal Behavior	SWAN
SWAN_SWAN_16	16. Reflects on questions (controls blurting out answers)	The Strengths and Weaknesses Assessment of Normal Behavior	SWAN
SWAN_SWAN_17	17. Awaits turn (stands in line and takes turns)	The Strengths and Weaknesses Assessment of Normal Behavior	SWAN
SWAN_SWAN_18	18. Enters into conversation and games without interrupting or intruding	The Strengths and Weaknesses Assessment of Normal Behavior	SWAN
SWAN_SWAN_IN	Inattention Average	The Strengths and Weaknesses Assessment of Normal Behavior	SWAN
SWAN_SWAN_HY	Hyperactivity Average	The Strengths and Weaknesses Assessment of Normal Behavior	SWAN
SWAN_SWAN_Total	SWAN Average	The Strengths and Weaknesses Assessment of Normal Behavior	SWAN
SympChck_CSC_01C	Feels sad and down most days for at least 1 week (current)	Symptom Checklist Parent report	SympChck-P
SympChck_CSC_01P	Feels sad and down most days for at least 1 week (past	Symptom Checklist Parent report	SympChck-P
SympChck_CSC_02C	Often feels annoyed and irritated (current)	Symptom Checklist Parent report	SympChck-P
SympChck_CSC_02P	Often feels annoyed and irritated (past	Symptom Checklist Parent report	SympChck-P
SympChck_CSC_03C	Has a loss of interest in previously enjoyable activities (current)	Symptom Checklist Parent report	SympChck-P

Variable name in data	Item text	Instrument name	Instrument abbreviation (if applicable)
SympChck_CSC_03P	Has a loss of interest in previously enjoyable activities (past	Symptom Checklist Parent report	SympChck-P
SympChck_CSC_04C	Often feels overly happy and silly, above and beyond a normal feeling (current)	Symptom Checklist Parent report	SympChck-P
SympChck_CSC_04P	Often feels overly happy and silly, above and beyond a normal feeling (past	Symptom Checklist Parent report	SympChck-P
SympChck_CSC_05C	Has strong and explosive feelings of anger (current)	Symptom Checklist Parent report	SympChck-P
SympChck_CSC_05P	Has strong and explosive feelings of anger (past	Symptom Checklist Parent report	SympChck-P
SympChck_CSC_06C	Has periods of unusual energy and activity (current)	Symptom Checklist Parent report	SympChck-P
SympChck_CSC_06P	Has periods of unusual energy and activity (past	Symptom Checklist Parent report	SympChck-P
SympChck_CSC_07C	Has times when he/she sleeps much less than usual but still feels rested (current)	Symptom Checklist Parent report	SympChck-P
SympChck_CSC_07P	Has times when he/she sleeps much less than usual but still feels rested (past	Symptom Checklist Parent report	SympChck-P
SympChck_CSC_09P	Hears, sees, or smells things that other people cannot (current)	Symptom Checklist Parent report	SympChck-P
SympChck_CSC_09C	Hears, sees, or smells things that other people cannot (past	Symptom Checklist Parent report	SympChck-P
SympChck_CSC_10C	Has unusual thoughts or beliefs that others cannot understand or believe (current)	Symptom Checklist Parent report	SympChck-P
SympChck_CSC_10P	Has unusual thoughts or beliefs that others cannot understand or believe (past	Symptom Checklist Parent report	SympChck-P
SympChck_CSC_11C	Has anxiety attacks, where out of the blue he/she suddenly feels scared (current)	Symptom Checklist Parent report	SympChck-P
SympChck_CSC_11P	Has anxiety attacks, where out of the blue he/she suddenly feels scared (past	Symptom Checklist Parent report	SympChck-P
SympChck_CSC_12C	(current)	Symptom Checklist Parent report	SympChck-P

Variable name in data	Item text	Instrument name	Instrument abbreviation (if applicable)
SympChck_CSC_12P	(past	Symptom Checklist Parent report	SympChck-P
SympChck_CSC_14C	Worries about being separated from parent/guardian because of getting lost or kidnapped (current)	Symptom Checklist Parent report	SympChck-P
SympChck_CSC_14P	Worries about being separated from parent/guardian because of getting lost or kidnapped (past	Symptom Checklist Parent report	SympChck-P
SympChck_CSC_15C	Worries excessively about harm occuring to parents/guardians (current)	Symptom Checklist Parent report	SympChck-P
SympChck_CSC_15P	Worries excessively about harm occuring to parents/guardians (past	Symptom Checklist Parent report	SympChck-P
SympChck_CSC_16P	Often does not want to go to school, due to worry about parent/guardian separation (past	Symptom Checklist Parent report	SympChck-P
SympChck_CSC_17C	Often has trouble going to sleep without parent/guardian nearby (current)	Symptom Checklist Parent report	SympChck-P
SympChck_CSC_17P	Often has trouble going to sleep without parent/guardian nearby (past	Symptom Checklist Parent report	SympChck-P
SympChck_CSC_18C	Is afraid of being alone at home or in a different room than parent/guardian (current)	Symptom Checklist Parent report	SympChck-P
SympChck_CSC_18P	Is afraid of being alone at home or in a different room than parent/guardian (past	Symptom Checklist Parent report	SympChck-P
SympChck_CSC_19C	Often feels very nervous around people (current)	Symptom Checklist Parent report	SympChck-P
SympChck_CSC_19P	Often feels very nervous around people (past	Symptom Checklist Parent report	SympChck-P
SympChck_CSC_20C	Often feels very nervous when he/she has to do things in front of others (current)	Symptom Checklist Parent report	SympChck-P
SympChck_CSC_20P	Often feels very nervous when he/she has to do things in front of others (past	Symptom Checklist Parent report	SympChck-P

Variable name in data	Item text	Instrument name	Instrument abbreviation (if applicable)
SympChck_CSC_21C	Is unable to speak in specific situations, such as school, despite being able to speak without a problem in other situations (current)	Symptom Checklist Parent report	SympChck-P
SympChck_CSC_21P	Is unable to speak in specific situations, such as school, despite being able to speak without a problem in other situations (past	Symptom Checklist Parent report	SympChck-P
SympChck_CSC_22C	Has intense fears of specific animals, situations, or anything else (current)	Symptom Checklist Parent report	SympChck-P
SympChck_CSC_22P	Has intense fears of specific animals, situations, or anything else (past	Symptom Checklist Parent report	SympChck-P
SympChck_CSC_23C	Worries most days of the week (current)	Symptom Checklist Parent report	SympChck-P
SympChck_CSC_23P	Worries most days of the week (past	Symptom Checklist Parent report	SympChck-P
SympChck_CSC_24C	Often misses school or other activities because he/she doesn't feel well (current)	Symptom Checklist Parent report	SympChck-P
SympChck_CSC_24P	Often misses school or other activities because he/she doesn't feel well (past	Symptom Checklist Parent report	SympChck-P
SympChck_CSC_25C	Feels very nervous and unable to relax most days of the week (current)	Symptom Checklist Parent report	SympChck-P
SympChck_CSC_25P	Feels very nervous and unable to relax most days of the week (past	Symptom Checklist Parent report	SympChck-P
SympChck_CSC_26C	Is bothered by thoughts which keep coming into his/her head for no reason (current)	Symptom Checklist Parent report	SympChck-P
SympChck_CSC_26P	Is bothered by thoughts which keep coming into his/her head for no reason (past	Symptom Checklist Parent report	SympChck-P
SympChck_CSC_27C	Feels like he/she has to do certain things in a very specific way (handwashing,	Symptom Checklist Parent report	SympChck-P

Variable name in data	Item text	Instrument name	Instrument abbreviation (if applicable)
	checking, doing things multiple times) (current)		
SympChck_CSC_27P	Feels like he/she has to do certain things in a very specific way (handwashing, checking, doing things multiple times) (past	Symptom Checklist Parent report	SympChck-P
SympChck_CSC_28C	Often has accidents when he/she wets the bed or self during the day (current)	Symptom Checklist Parent report	SympChck-P
SympChck_CSC_28P	Often has accidents when he/she wets the bed or self during the day (past	Symptom Checklist Parent report	SympChck-P
SympChck_CSC_29P	Often has accidents when he/she soild the bed or self during the day (past	Symptom Checklist Parent report	SympChck-P
SympChck_CSC_30C	Feels extremely worried about gaining weight or becoming fat (current)	Symptom Checklist Parent report	SympChck-P
SympChck_CSC_30P	Feels extremely worried about gaining weight or becoming fat (past	Symptom Checklist Parent report	SympChck-P
SympChck_CSC_31C	Is underweight for his/her age and height (current)	Symptom Checklist Parent report	SympChck-P
SympChck_CSC_31P	Is underweight for his/her age and height (past	Symptom Checklist Parent report	SympChck-P
SympChck_CSC_33C	Has frequent eating binges, and it feels out of control (current)	Symptom Checklist Parent report	SympChck-P
SympChck_CSC_34C	Has trouble paying attention, and it affects school work or performance (current)	Symptom Checklist Parent report	SympChck-P
SympChck_CSC_34P	Has trouble paying attention, and it affects school work or performance (past	Symptom Checklist Parent report	SympChck-P
SympChck_CSC_35C	Is often easily distracted (current)	Symptom Checklist Parent report	SympChck-P
SympChck_CSC_35P	Is often easily distracted (past	Symptom Checklist Parent report	SympChck-P
SympChck_CSC_36C	Has difficulty remaining seated at home or school (current)	Symptom Checklist Parent report	SympChck-P

Variable name in data	Item text	Instrument name	Instrument abbreviation (if applicable)
SympChck_CSC_36P	Has difficulty remaining seated at home or school (past	Symptom Checklist Parent report	SympChck-P
SympChck_CSC_37C	Often acts before thinking (current)	Symptom Checklist Parent report	SympChck-P
SympChck_CSC_37P	Often acts before thinking (past	Symptom Checklist Parent report	SympChck-P
SympChck_CSC_38C	Often becomes really upset and loses his/her temper (current)	Symptom Checklist Parent report	SympChck-P
SympChck_CSC_38P	Often becomes really upset and loses his/her temper (past	Symptom Checklist Parent report	SympChck-P
SympChck_CSC_39C	Argues or talks back to adults, more than others his/her age (current)	Symptom Checklist Parent report	SympChck-P
SympChck_CSC_39P	Argues or talks back to adults, more than others his/her age (past	Symptom Checklist Parent report	SympChck-P
SympChck_CSC_40C	Actively disobeys or doesn't listen to adult rules (current)	Symptom Checklist Parent report	SympChck-P
SympChck_CSC_40P	Actively disobeys or doesn't listen to adult rules (past	Symptom Checklist Parent report	SympChck-P
SympChck_CSC_41C	Frequently lies (current)	Symptom Checklist Parent report	SympChck-P
SympChck_CSC_41P	Frequently lies (past	Symptom Checklist Parent report	SympChck-P
SympChck_CSC_42C	Has skipped a part or a whole day of school (current)	Symptom Checklist Parent report	SympChck-P
SympChck_CSC_42P	Has skipped a part or a whole day of school (past	Symptom Checklist Parent report	SympChck-P
SympChck_CSC_43P	Frequently starts physical fights with peers (past	Symptom Checklist Parent report	SympChck-P
SympChck_CSC_44C	Bullies, threatens, or intimidates others (current)	Symptom Checklist Parent report	SympChck-P
SympChck_CSC_44P	Bullies, threatens, or intimidates others (past	Symptom Checklist Parent report	SympChck-P
SympChck_CSC_45P	Has stolen or shoplifted items (past	Symptom Checklist Parent report	SympChck-P
SympChck_CSC_46C	Notices muscles moving uncontrollably (blinking a lot, shrugging) (current)	Symptom Checklist Parent report	SympChck-P
SympChck_CSC_46P	Notices muscles moving uncontrollably	Symptom Checklist Parent report	SympChck-P

Variable name in data	Item text	Instrument name	Instrument abbreviation (if applicable)
	(blinking a lot, shrugging) (past		
SympChck_CSC_47C	Makes noises that he/she can't control (repeating sounds, sniffing) (current)	Symptom Checklist Parent report	SympChck-P
SympChck_CSC_47P	Makes noises that he/she can't control (repeating sounds, sniffing) (past	Symptom Checklist Parent report	SympChck-P
SympChck_CSC_48C	Has unusual physical mannerisms (rocked body or flapping hands) (current)	Symptom Checklist Parent report	SympChck-P
SympChck_CSC_48P	Has unusual physical mannerisms (rocked body or flapping hands) (past	Symptom Checklist Parent report	SympChck-P
SympChck_CSC_49C	Feels really upset when there is an unexpected change in his/her schedule (current)	Symptom Checklist Parent report	SympChck-P
SympChck_CSC_49P	Feels really upset when there is an unexpected change in his/her schedule (past	Symptom Checklist Parent report	SympChck-P
SympChck_CSC_50C	Is preoccupied with very specific objects, routines, or interests (current)	Symptom Checklist Parent report	SympChck-P
SympChck_CSC_50P	Is preoccupied with very specific objects, routines, or interests (past	Symptom Checklist Parent report	SympChck-P
SympChck_CSC_51C	Often has a difficult time making eye contact (current)	Symptom Checklist Parent report	SympChck-P
SympChck_CSC_51P	Often has a difficult time making eye contact (past	Symptom Checklist Parent report	SympChck-P
SympChck_CSC_52C	Has a hard time expressing feelings on his/her face during social interactions (current)	Symptom Checklist Parent report	SympChck-P
SympChck_CSC_52P	Has a hard time expressing feelings on his/her face during social interactions (past	Symptom Checklist Parent report	SympChck-P
WIAT_WIAT_Num_Raw	Numerical Operations Raw Score	Wechsler Individual Achievement Test	WIAT
WIAT_WIAT_Num_Stnd	Numerical Operations Standard Score	Wechsler Individual Achievement Test	WIAT

Variable name in data	Item text	Instrument name	Instrument abbreviation (if applicable)
WIAT_WIAT_Num_P	Numerical Operations Percentile Rank	Wechsler Individual Achievement Test	WIAT
WIAT_WIAT_Pseudo_Raw	Pseudo-word Decoding Raw Score	Wechsler Individual Achievement Test	WIAT
WIAT_WIAT_Pseudo_Stnd	Pseudo-word Decoding Standard Score	Wechsler Individual Achievement Test	WIAT
WIAT_WIAT_Pseudo_P	Pseudo-word Decoding Percentile Rank	Wechsler Individual Achievement Test	WIAT
WIAT_WIAT_Spell_Raw	Spelling Raw Score	Wechsler Individual Achievement Test	WIAT
WIAT_WIAT_Spell_Stnd	Spelling Standard Score	Wechsler Individual Achievement Test	WIAT
WIAT_WIAT_Spell_P	Spelling Percentile Rank	Wechsler Individual Achievement Test	WIAT
WIAT_WIAT_Word_Raw	Word Reading Raw Score	Wechsler Individual Achievement Test	WIAT
WIAT_WIAT_Word_Stnd	Word Reading Standard Score	Wechsler Individual Achievement Test	WIAT
WIAT_WIAT_Word_P	Word Reading Percentile Rank	Wechsler Individual Achievement Test	WIAT
WIAT_WIAT_LCRV_Raw	Listening Comprehension Receptive Vocabulary Raw Score	Wechsler Individual Achievement Test	WIAT
WIAT_WIAT_LCRV_Std	Listening Comprehension Receptive Vocabulary Standard Score	Wechsler Individual Achievement Test	WIAT
WIAT_WIAT_LCRV_P	Listening Comprehension Receptive Vocabulary Percentile Rank	Wechsler Individual Achievement Test	WIAT
WIAT_WIAT_LCODC_Raw	Listening Comprehension Oral Discourse Comprehension Raw Score	Wechsler Individual Achievement Test	WIAT
WIAT_WIAT_LCODC_Stnd	Listening Comprehension Oral Discourse Comprehension Standard Score	Wechsler Individual Achievement Test	WIAT
WIAT_WIAT_LCODC_P	Listening Comprehension Oral Discourse Comprehension Percentile Rank	Wechsler Individual Achievement Test	WIAT
WIAT_WIAT_LC_Stnd	Listening Comprehension Standard Score	Wechsler Individual Achievement Test	WIAT
WIAT_WIAT_LC_P	Listening Comprehension Percentile Rank	Wechsler Individual Achievement Test	WIAT

Variable name in data	Item text	Instrument name	Instrument abbreviation (if applicable)
WIAT_WIAT_MP_Raw	Math Problem Solving Raw Score	Wechsler Individual Achievement Test	WIAT
WIAT_WIAT_MP_Stnd	Math Problem Solving Standard Score	Wechsler Individual Achievement Test	WIAT
WIAT_WIAT_MP_P	Math Problem Solving Percentile Rank	Wechsler Individual Achievement Test	WIAT

Table S2. List of imaging phenotypes used in CCA-ICA

Variable name in data	Item text	Instrument name
lh_bankssts_area	Cortical area in lh bankssts	Cortical Area
lh_caudalanteriorcingulate_area	Cortical area in lh caudalanteriorcingulate	Cortical Area
lh_caudalmiddlefrontal_area	Cortical area in lh caudalmiddlefrontal	Cortical Area
lh_cuneus_area	Cortical area in lh cuneus	Cortical Area
lh_entorhinal_area	Cortical area in lh entorhinal	Cortical Area
lh_fusiform_area	Cortical area in lh fusiform	Cortical Area
lh_inferiorparietal_area	Cortical area in lh inferiorparietal	Cortical Area
lh_inferiortemporal_area	Cortical area in lh inferiortemporal	Cortical Area
lh_isthmuscingulate_area	Cortical area in lh isthmuscingulate	Cortical Area
lh_lateraloccipital_area	Cortical area in lh lateraloccipital	Cortical Area
lh_lateralorbitofrontal_area	Cortical area in lh lateralorbitofrontal	Cortical Area
lh_lingual_area	Cortical area in lh lingual	Cortical Area
lh_medialorbitofrontal_area	Cortical area in lh medialorbitofrontal	Cortical Area
lh_middletemporal_area	Cortical area in lh middletemporal	Cortical Area
lh_parahippocampal_area	Cortical area in lh parahippocampal	Cortical Area
lh_paracentral_area	Cortical area in lh paracentral	Cortical Area
lh_parsopercularis_area	Cortical area in lh parsopercularis	Cortical Area
lh_parsorbitalis_area	Cortical area in lh parsorbitalis	Cortical Area
lh_parstriangularis_area	Cortical area in lh parstriangularis	Cortical Area
lh_pericalcarine_area	Cortical area in lh pericalcarine	Cortical Area
lh_postcentral_area	Cortical area in lh postcentral	Cortical Area
lh_posteriorcingulate_area	Cortical area in lh posteriorcingulate	Cortical Area
lh_precentral_area	Cortical area in lh precentral	Cortical Area
lh_precuneus_area	Cortical area in lh precuneus	Cortical Area
lh_rostralanteriorcingulate_area	Cortical area in lh rostralanteriorcingulate	Cortical Area
lh_rostralmiddlefrontal_area	Cortical area in lh rostralmiddlefrontal	Cortical Area
lh_superiorfrontal_area	Cortical area in lh superiorfrontal	Cortical Area
lh_superiorparietal_area	Cortical area in lh superiorparietal	Cortical Area
lh_superiortemporal_area	Cortical area in lh superiortemporal	Cortical Area
lh_supramarginal_area	Cortical area in lh supramarginal	Cortical Area
lh_frontalpole_area	Cortical area in lh frontalpole	Cortical Area
lh_temporalpole_area	Cortical area in lh temporalpole	Cortical Area
lh_transversetemporal_area	Cortical area in lh transversetemporal	Cortical Area
lh_insula_area	Cortical area in lh insula	Cortical Area

Variable name in data	Item text	Instrument name
lh_WhiteSurfArea_area	Cortical area in lh WhiteSurfArea	Cortical Area
lh_bankssts_thickness	Cortical thickness in lh bankssts	Cortical Thickness
lh_caudalanteriorcingulate_thickness	Cortical thickness in lh caudalanteriorcingulate	Cortical Thickness
lh_caudalmiddlefrontal_thickness	Cortical thickness in lh caudalmiddlefrontal	Cortical Thickness
lh_cuneus_thickness	Cortical thickness in lh cuneus	Cortical Thickness
lh_entorhinal_thickness	Cortical thickness in lh entorhinal	Cortical Thickness
lh_fusiform_thickness	Cortical thickness in lh fusiform	Cortical Thickness
lh_inferiorparietal_thickness	Cortical thickness in lh inferiorparietal	Cortical Thickness
lh_inferiortemporal_thickness	Cortical thickness in lh inferiortemporal	Cortical Thickness
lh_isthmuscingulate_thickness	Cortical thickness in lh isthmuscingulate	Cortical Thickness
lh_lateraloccipital_thickness	Cortical thickness in lh lateraloccipital	Cortical Thickness
lh_lateralorbitofrontal_thickness	Cortical thickness in lh lateralorbitofrontal	Cortical Thickness
lh_lingual_thickness	Cortical thickness in lh lingual	Cortical Thickness
lh_medialorbitofrontal_thickness	Cortical thickness in lh medialorbitofrontal	Cortical Thickness
lh_middletemporal_thickness	Cortical thickness in lh middletemporal	Cortical Thickness
lh_parahippocampal_thickness	Cortical thickness in lh parahippocampal	Cortical Thickness
lh_paracentral_thickness	Cortical thickness in lh paracentral	Cortical Thickness
lh_parsopercularis_thickness	Cortical thickness in lh parsopercularis	Cortical Thickness
lh_parsorbitalis_thickness	Cortical thickness in lh parsorbitalis	Cortical Thickness
lh_parstriangularis_thickness	Cortical thickness in lh parstriangularis	Cortical Thickness
lh_pericalcarine_thickness	Cortical thickness in lh pericalcarine	Cortical Thickness
lh_postcentral_thickness	Cortical thickness in lh postcentral	Cortical Thickness
lh_posteriorcingulate_thickness	Cortical thickness in lh posteriorcingulate	Cortical Thickness
lh_precentral_thickness	Cortical thickness in lh precentral	Cortical Thickness
lh_precuneus_thickness	Cortical thickness in lh precuneus	Cortical Thickness
lh_rostralanteriorcingulate_thickness	Cortical thickness in lh rostralanteriorcingulate	Cortical Thickness
lh_rostralmiddlefrontal_thickness	Cortical thickness in lh rostralmiddlefrontal	Cortical Thickness
lh_superiorfrontal_thickness	Cortical thickness in lh superiorfrontal	Cortical Thickness
lh_superiorparietal_thickness	Cortical thickness in lh superiorparietal	Cortical Thickness
lh_superiortemporal_thickness	Cortical thickness in lh superiortemporal	Cortical Thickness

Variable name in data	Item text	Instrument name
lh_supramarginal_thickness	Cortical thickness in lh supramarginal	Cortical Thickness
lh_frontalpole_thickness	Cortical thickness in lh frontalpole	Cortical Thickness
lh_temporalpole_thickness	Cortical thickness in lh temporalpole	Cortical Thickness
lh_transversetemporal_thickness	Cortical thickness in lh transversetemporal	Cortical Thickness
lh_insula_thickness	Cortical thickness in lh insula	Cortical Thickness
lh_MeanThickness_thickness	Cortical thickness in lh MeanThickness	Cortical Thickness
lh_bankssts_volume	Cortical volume in lh bankssts	Cortical Volume
lh_caudalanteriorcingulate_volume	Cortical volume in lh caudalanteriorcingulate	Cortical Volume
lh_caudalmiddlefrontal_volume	Cortical volume in lh caudalmiddlefrontal	Cortical Volume
lh_cuneus_volume	Cortical volume in lh cuneus	Cortical Volume
lh_entorhinal_volume	Cortical volume in lh entorhinal	Cortical Volume
lh_fusiform_volume	Cortical volume in lh fusiform	Cortical Volume
lh_inferiorparietal_volume	Cortical volume in lh inferiorparietal	Cortical Volume
lh_inferiortemporal_volume	Cortical volume in lh inferiortemporal	Cortical Volume
lh_isthmuscingulate_volume	Cortical volume in lh isthmuscingulate	Cortical Volume
lh_lateraloccipital_volume	Cortical volume in lh lateraloccipital	Cortical Volume
lh_lateralorbitofrontal_volume	Cortical volume in lh lateralorbitofrontal	Cortical Volume
lh_lingual_volume	Cortical volume in lh lingual	Cortical Volume
lh_medialorbitofrontal_volume	Cortical volume in lh medialorbitofrontal	Cortical Volume
lh_middletemporal_volume	Cortical volume in lh middletemporal	Cortical Volume
lh_parahippocampal_volume	Cortical volume in lh parahippocampal	Cortical Volume
lh_paracentral_volume	Cortical volume in lh paracentral	Cortical Volume
lh_parsopercularis_volume	Cortical volume in lh parsopercularis	Cortical Volume
lh_parsorbitalis_volume	Cortical volume in lh parsorbitalis	Cortical Volume
lh_parstriangularis_volume	Cortical volume in lh parstriangularis	Cortical Volume
lh_pericalcarine_volume	Cortical volume in lh pericalcarine	Cortical Volume
lh_postcentral_volume	Cortical volume in lh postcentral	Cortical Volume
lh_posteriorcingulate_volume	Cortical volume in lh posteriorcingulate	Cortical Volume
lh_precentral_volume	Cortical volume in lh precentral	Cortical Volume
lh_precuneus_volume	Cortical volume in lh precuneus	Cortical Volume
lh_rostralanteriorcingulate_volume	Cortical volume in lh rostralanteriorcingulate	Cortical Volume
lh_rostralmiddlefrontal_volume	Cortical volume in lh rostralmiddlefrontal	Cortical Volume

Variable name in data	Item text	Instrument name
lh_superiorfrontal_volume	Cortical volume in lh superiorfrontal	Cortical Volume
lh_superiorparietal_volume	Cortical volume in lh superiorparietal	Cortical Volume
lh_superiortemporal_volume	Cortical volume in lh superiortemporal	Cortical Volume
lh_supramarginal_volume	Cortical volume in lh supramarginal	Cortical Volume
lh_frontalpole_volume	Cortical volume in lh frontalpole	Cortical Volume
lh_temporalpole_volume	Cortical volume in lh temporalpole	Cortical Volume
lh_transversetemporal_volume	Cortical volume in lh transversetemporal	Cortical Volume
lh_insula_volume	Cortical volume in lh insula	Cortical Volume
rh_bankssts_area	Cortical area in rh bankssts	Cortical Area
rh_caudalanteriorcingulate_area	Cortical area in rh caudalanteriorcingulate	Cortical Area
rh_caudalmiddlefrontal_area	Cortical area in rh caudalmiddlefrontal	Cortical Area
rh_cuneus_area	Cortical area in rh cuneus	Cortical Area
rh_entorhinal_area	Cortical area in rh entorhinal	Cortical Area
rh_fusiform_area	Cortical area in rh fusiform	Cortical Area
rh_inferiorparietal_area	Cortical area in rh inferiorparietal	Cortical Area
rh_inferiortemporal_area	Cortical area in rh inferiortemporal	Cortical Area
rh_isthmuscingulate_area	Cortical area in rh isthmuscingulate	Cortical Area
rh_lateraloccipital_area	Cortical area in rh lateraloccipital	Cortical Area
rh_lateralorbitofrontal_area	Cortical area in rh lateralorbitofrontal	Cortical Area
rh_lingual_area	Cortical area in rh lingual	Cortical Area
rh_medialorbitofrontal_area	Cortical area in rh medialorbitofrontal	Cortical Area
rh_middletemporal_area	Cortical area in rh middletemporal	Cortical Area
rh_parahippocampal_area	Cortical area in rh parahippocampal	Cortical Area
rh_paracentral_area	Cortical area in rh paracentral	Cortical Area
rh_parsopercularis_area	Cortical area in rh parsopercularis	Cortical Area
rh_parsorbitalis_area	Cortical area in rh parsorbitalis	Cortical Area
rh_parstriangularis_area	Cortical area in rh parstriangularis	Cortical Area
rh_pericalcarine_area	Cortical area in rh pericalcarine	Cortical Area
rh_postcentral_area	Cortical area in rh postcentral	Cortical Area
rh_posteriorcingulate_area	Cortical area in rh posteriorcingulate	Cortical Area
rh_precentral_area	Cortical area in rh precentral	Cortical Area
rh_precuneus_area	Cortical area in rh precuneus	Cortical Area
rh_rostralanteriorcingulate_area	Cortical area in rh rostralanteriorcingulate	Cortical Area
rh_rostralmiddlefrontal_area	Cortical area in rh rostralmiddlefrontal	Cortical Area
rh_superiorfrontal_area	Cortical area in rh superiorfrontal	Cortical Area

Variable name in data	Item text	Instrument name
rh_superiorparietal_area	Cortical area in rh superiorparietal	Cortical Area
rh_superiortemporal_area	Cortical area in rh superiortemporal	Cortical Area
rh_supramarginal_area	Cortical area in rh supramarginal	Cortical Area
rh_frontalpole_area	Cortical area in rh frontalpole	Cortical Area
rh_temporalpole_area	Cortical area in rh temporalpole	Cortical Area
rh_transversetemporal_area	Cortical area in rh transversetemporal	Cortical Area
rh_insula_area	Cortical area in rh insula	Cortical Area
rh_WhiteSurfArea_area	Cortical area in rh WhiteSurfArea	Cortical Area
rh_bankssts_thickness	Cortical thickness in rh bankssts	Cortical Thickness
rh_caudalanteriorcingulate_thickness	Cortical thickness in rh caudalanteriorcingulate	Cortical Thickness
rh_caudalmiddlefrontal_thickness	Cortical thickness in rh caudalmiddlefrontal	Cortical Thickness
rh_cuneus_thickness	Cortical thickness in rh cuneus	Cortical Thickness
rh_entorhinal_thickness	Cortical thickness in rh entorhinal	Cortical Thickness
rh_fusiform_thickness	Cortical thickness in rh fusiform	Cortical Thickness
rh_inferiorparietal_thickness	Cortical thickness in rh inferiorparietal	Cortical Thickness
rh_inferiortemporal_thickness	Cortical thickness in rh inferiortemporal	Cortical Thickness
rh_isthmuscingulate_thickness	Cortical thickness in rh isthmuscingulate	Cortical Thickness
rh_lateraloccipital_thickness	Cortical thickness in rh lateraloccipital	Cortical Thickness
rh_lateralorbitofrontal_thickness	Cortical thickness in rh lateralorbitofrontal	Cortical Thickness
rh_lingual_thickness	Cortical thickness in rh lingual	Cortical Thickness
rh_medialorbitofrontal_thickness	Cortical thickness in rh medialorbitofrontal	Cortical Thickness
rh_middletemporal_thickness	Cortical thickness in rh middletemporal	Cortical Thickness
rh_parahippocampal_thickness	Cortical thickness in rh parahippocampal	Cortical Thickness
rh_paracentral_thickness	Cortical thickness in rh paracentral	Cortical Thickness
rh_parsopercularis_thickness	Cortical thickness in rh parsopercularis	Cortical Thickness
rh_parsorbitalis_thickness	Cortical thickness in rh parsorbitalis	Cortical Thickness
rh_parstriangularis_thickness	Cortical thickness in rh parstriangularis	Cortical Thickness
rh_pericalcarine_thickness	Cortical thickness in rh pericalcarine	Cortical Thickness
rh_postcentral_thickness	Cortical thickness in rh postcentral	Cortical Thickness
rh_posteriorcingulate_thickness	Cortical thickness in rh posteriorcingulate	Cortical Thickness
rh_precentral_thickness	Cortical thickness in rh precentral	Cortical Thickness

Variable name in data	Item text	Instrument name
rh_precuneus_thickness	Cortical thickness in rh precuneus	Cortical Thickness
rh_rostralanteriorcingulate_thickness	Cortical thickness in rh rostralanteriorcingulate	Cortical Thickness
rh_rostralmiddlefrontal_thickness	Cortical thickness in rh rostralmiddlefrontal	Cortical Thickness
rh_superiorfrontal_thickness	Cortical thickness in rh superiorfrontal	Cortical Thickness
rh_superiorparietal_thickness	Cortical thickness in rh superiorparietal	Cortical Thickness
rh_superiortemporal_thickness	Cortical thickness in rh superiortemporal	Cortical Thickness
rh_supramarginal_thickness	Cortical thickness in rh supramarginal	Cortical Thickness
rh_frontalpole_thickness	Cortical thickness in rh frontalpole	Cortical Thickness
rh_temporalpole_thickness	Cortical thickness in rh temporalpole	Cortical Thickness
rh_transversetemporal_thickness	Cortical thickness in rh transversetemporal	Cortical Thickness
rh_insula_thickness	Cortical thickness in rh insula	Cortical Thickness
rh_MeanThickness_thickness	Cortical thickness in rh MeanThickness	Cortical Thickness
rh_bankssts_volume	Cortical volume in rh bankssts	Cortical Volume
rh_caudalanteriorcingulate_volume	Cortical volume in rh caudalanteriorcingulate	Cortical Volume
rh_caudalmiddlefrontal_volume	Cortical volume in rh caudalmiddlefrontal	Cortical Volume
rh_cuneus_volume	Cortical volume in rh cuneus	Cortical Volume
rh_entorhinal_volume	Cortical volume in rh entorhinal	Cortical Volume
rh_fusiform_volume	Cortical volume in rh fusiform	Cortical Volume
rh_inferiorparietal_volume	Cortical volume in rh inferiorparietal	Cortical Volume
rh_inferiortemporal_volume	Cortical volume in rh inferiortemporal	Cortical Volume
rh_isthmuscingulate_volume	Cortical volume in rh isthmuscingulate	Cortical Volume
rh_lateraloccipital_volume	Cortical volume in rh lateraloccipital	Cortical Volume
rh_lateralorbitofrontal_volume	Cortical volume in rh lateralorbitofrontal	Cortical Volume
rh_lingual_volume	Cortical volume in rh lingual	Cortical Volume
rh_medialorbitofrontal_volume	Cortical volume in rh medialorbitofrontal	Cortical Volume
rh_middletemporal_volume	Cortical volume in rh middletemporal	Cortical Volume
rh_parahippocampal_volume	Cortical volume in rh parahippocampal	Cortical Volume
rh_paracentral_volume	Cortical volume in rh paracentral	Cortical Volume
rh_parsopercularis_volume	Cortical volume in rh parsopercularis	Cortical Volume
rh_parsorbitalis_volume	Cortical volume in rh parsorbitalis	Cortical Volume
rh_parstriangularis_volume	Cortical volume in rh parstriangularis	Cortical Volume
rh_pericalcarine_volume	Cortical volume in rh pericalcarine	Cortical Volume

Variable name in data	Item text	Instrument name
rh_postcentral_volume	Cortical volume in rh postcentral	Cortical Volume
rh_posteriorcingulate_volume	Cortical volume in rh posteriorcingulate	Cortical Volume
rh_precentral_volume	Cortical volume in rh precentral	Cortical Volume
rh_precuneus_volume	Cortical volume in rh precuneus	Cortical Volume
rh_rostralanteriorcingulate_volume	Cortical volume in rh rostralanteriorcingulate	Cortical Volume
rh_rostralmiddlefrontal_volume	Cortical volume in rh rostralmiddlefrontal	Cortical Volume
rh_superiorfrontal_volume	Cortical volume in rh superiorfrontal	Cortical Volume
rh_superiorparietal_volume	Cortical volume in rh superiorparietal	Cortical Volume
rh_superiortemporal_volume	Cortical volume in rh superiortemporal	Cortical Volume
rh_supramarginal_volume	Cortical volume in rh supramarginal	Cortical Volume
rh_frontalpole_volume	Cortical volume in rh frontalpole	Cortical Volume
rh_temporalpole_volume	Cortical volume in rh temporalpole	Cortical Volume
rh_transversetemporal_volume	Cortical volume in rh transversetemporal	Cortical Volume
rh_insula_volume	Cortical volume in rh insula	Cortical Volume
Left_Lateral_Ventricle	Volume of the Left Lateral Ventricle	Subcortical Volume
Left_Inf_Lat_Vent	Volume of the Left Inf Lat Vent	Subcortical Volume
Left_Cerebellum_White_Matter	Volume of the Left Cerebellum White Matter	Subcortical Volume
Left_Cerebellum_Cortex	Volume of the Left Cerebellum Cortex	Subcortical Volume
Left_Thalamus	Volume of the Left Thalamus	Subcortical Volume
Left_Caudate	Volume of the Left Caudate	Subcortical Volume
Left_Putamen	Volume of the Left Putamen	Subcortical Volume
Left_Pallidum	Volume of the Left Pallidum	Subcortical Volume
x3rd_Ventricle	Volume of the x3rd Ventricle	Subcortical Volume
x4th_Ventricle	Volume of the x4th Ventricle	Subcortical Volume
Brain_Stem	Volume of the Brain Stem	Subcortical Volume
Left_Hippocampus	Volume of the Left Hippocampus	Subcortical Volume
Left_Amygdala	Volume of the Left Amygdala	Subcortical Volume
CSF	Volume of the CSF	Subcortical Volume
Left_Accumbens_area	Volume of the Left Accumbens area	Subcortical Volume
Left_VentralDC	Volume of the Left VentralDC	Subcortical Volume
Left_vessel	Volume of the Left vessel	Subcortical Volume
Left_choroid_plexus	Volume of the Left choroid plexus	Subcortical Volume
Right_Lateral_Ventricle	Volume of the Right Lateral Ventricle	Subcortical Volume
Right_Inf_Lat_Vent	Volume of the Right Inf Lat Vent	Subcortical Volume
Right_Cerebellum_White_Matter	Volume of the Right Cerebellum White Matter	Subcortical Volume
Right_Cerebellum_Cortex	Volume of the Right Cerebellum Cortex	Subcortical Volume

Variable name in data	Item text	Instrument name
Right_Thalamus	Volume of the Right Thalamus	Subcortical Volume
Right_Caudate	Volume of the Right Caudate	Subcortical Volume
Right_Putamen	Volume of the Right Putamen	Subcortical Volume
Right_Pallidum	Volume of the Right Pallidum	Subcortical Volume
Right_Hippocampus	Volume of the Right Hippocampus	Subcortical Volume
Right_Amygdala	Volume of the Right Amygdala	Subcortical Volume
Right_Accumbens_area	Volume of the Right Accumbens area	Subcortical Volume
Optic_Chiasm	Volume of the Optic Chiasm	Subcortical Volume
CC_Posterior	Volume of the CC Posterior	Subcortical Volume
CC_Mid_Posterior	Volume of the CC Mid Posterior	Subcortical Volume
CC_Central	Volume of the CC Central	Subcortical Volume
CC_Mid_Anterior	Volume of the CC Mid Anterior	Subcortical Volume
CC_Anterior	Volume of the CC Anterior	Subcortical Volume
left_Hippocampal_tail	Volume of the left Hippocampal tail	Hippocampus Subfields
left_subiculum_body	Volume of the left subiculum body	Hippocampus Subfields
left_CA1_body	Volume of the left CA1 body	Hippocampus Subfields
left_subiculum_head	Volume of the left subiculum head	Hippocampus Subfields
left_hippocampal_fissure	Volume of the left hippocampal fissure	Hippocampus Subfields
left_presubiculum_head	Volume of the left presubiculum head	Hippocampus Subfields
left_CA1_head	Volume of the left CA1 head	Hippocampus Subfields
left_presubiculum_body	Volume of the left presubiculum body	Hippocampus Subfields
left_parasubiculum	Volume of the left parasubiculum	Hippocampus Subfields
left_molecular_layer_HP_head	Volume of the left molecular layer HP head	Hippocampus Subfields
left_molecular_layer_HP_body	Volume of the left molecular layer HP body	Hippocampus Subfields
left_GC_ML_DG_head	Volume of the left GC ML DG head	Hippocampus Subfields
left_CA3_body	Volume of the left CA3 body	Hippocampus Subfields
left_GC_ML_DG_body	Volume of the left GC ML DG body	Hippocampus Subfields
left_CA4_head	Volume of the left CA4 head	Hippocampus Subfields
left_CA4_body	Volume of the left CA4 body	Hippocampus Subfields
left_fimbria	Volume of the left fimbria	Hippocampus Subfields
left_CA3_head	Volume of the left CA3 head	Hippocampus Subfields
left_HATA	Volume of the left HATA	Hippocampus Subfields
left_Whole_hippocampal_body	Volume of the left Whole hippocampal body	Hippocampus Subfields
left_Whole_hippocampal_head	Volume of the left Whole hippocampal head	Hippocampus Subfields
left_Whole_hippocampus	Volume of the left Whole hippocampus	Hippocampus Subfields
right_Hippocampal_tail	Volume of the right Hippocampal tail	Hippocampus Subfields
right_subiculum_body	Volume of the right subiculum body	Hippocampus Subfields
right_CA1_body	Volume of the right CA1 body	Hippocampus Subfields

Variable name in data	Item text	Instrument name
right_subiculum_head	Volume of the right subiculum head	Hippocampus Subfields
right_hippocampal_fissure	Volume of the right hippocampal fissure	Hippocampus Subfields
right_presubiculum_head	Volume of the right presubiculum head	Hippocampus Subfields
right_CA1_head	Volume of the right CA1 head	Hippocampus Subfields
right_presubiculum_body	Volume of the right presubiculum body	Hippocampus Subfields
right_parasubiculum	Volume of the right parasubiculum	Hippocampus Subfields
right_molecular_layer_HP_head	Volume of the right molecular layer HP head	Hippocampus Subfields
right_molecular_layer_HP_body	Volume of the right molecular layer HP body	Hippocampus Subfields
right_GC_ML_DG_head	Volume of the right GC ML DG head	Hippocampus Subfields
right_CA3_body	Volume of the right CA3 body	Hippocampus Subfields
right_GC_ML_DG_body	Volume of the right GC ML DG body	Hippocampus Subfields
right_CA4_head	Volume of the right CA4 head	Hippocampus Subfields
right_CA4_body	Volume of the right CA4 body	Hippocampus Subfields
right_fimbria	Volume of the right fimbria	Hippocampus Subfields
right_CA3_head	Volume of the right CA3 head	Hippocampus Subfields
right_HATA	Volume of the right HATA	Hippocampus Subfields
right_Whole_hippocampal_body	Volume of the right Whole hippocampal body	Hippocampus Subfields
right_Whole_hippocampal_head	Volume of the right Whole hippocampal head	Hippocampus Subfields
right_Whole_hippocampus	Volume of the right Whole hippocampus	Hippocampus Subfields
left_Lateral_nucleus	Volume of the left Lateral nucleus	Amygdala Nuclei
left_Basal_nucleus	Volume of the left Basal nucleus	Amygdala Nuclei
left_Accessory_Basal_nucleus	Volume of the left Accessory Basal nucleus	Amygdala Nuclei
left_Anterior_amygdaloid_area_AAA	Volume of the left Anterior amygdaloid area AAA	Amygdala Nuclei
left_Central_nucleus	Volume of the left Central nucleus	Amygdala Nuclei
left_Medial_nucleus	Volume of the left Medial nucleus	Amygdala Nuclei
left_Cortical_nucleus	Volume of the left Cortical nucleus	Amygdala Nuclei
left_Corticoamygdaloid_transitio	Volume of the left Corticoamygdaloid transitio	Amygdala Nuclei
left_Paralaminar_nucleus	Volume of the left Paralaminar nucleus	Amygdala Nuclei
left_Whole_amygdala	Volume of the left Whole amygdala	Amygdala Nuclei
right_Lateral_nucleus	Volume of the right Lateral nucleus	Amygdala Nuclei
right_Basal_nucleus	Volume of the right Basal nucleus	Amygdala Nuclei
right_Accessory_Basal_nucleus	Volume of the right Accessory Basal nucleus	Amygdala Nuclei

Variable name in data	Item text	Instrument name
right_Anterior_amygdaloid_are a_AAA	Volume of the right Anterior amygdaloid area AAA	Amygdala Nuclei
right_Central_nucleus	Volume of the right Central nucleus	Amygdala Nuclei
right_Medial_nucleus	Volume of the right Medial nucleus	Amygdala Nuclei
right_Cortical_nucleus	Volume of the right Cortical nucleus	Amygdala Nuclei
right_Corticoamygdaloid_transi tio	Volume of the right Corticoamygdaloid transitio	Amygdala Nuclei
right_Paralaminar_nucleus	Volume of the right Paralaminar nucleus	Amygdala Nuclei
right_Whole_amygdala	Volume of the right Whole amygdala	Amygdala Nuclei
leftAnterior_inferior	Volume of the left Anterior inferior	Hypothalamic Subunits
leftAnterior_superior	Volume of the left Anterior superior	Hypothalamic Subunits
leftPosterior	Volume of the left Posterior	Hypothalamic Subunits
leftTubularInferior	Volume of the left TubularInferior	Hypothalamic Subunits
leftTubularSuperior	Volume of the left TubularSuperior	Hypothalamic Subunits
rightAnterior_inferior	Volume of the right Anterior inferior	Hypothalamic Subunits
rightAnterior_superior	Volume of the right Anterior superior	Hypothalamic Subunits
rightPosterior	Volume of the right Posterior	Hypothalamic Subunits
rightTubularInferior	Volume of the right TubularInferior	Hypothalamic Subunits
rightTubularSuperior	Volume of the right TubularSuperior	Hypothalamic Subunits
wholeLeft	Volume of the wholeLeft	Hypothalamic Subunits
wholeRight	Volume of the wholeRight	Hypothalamic Subunits
lh_AV	Volume of the left AV	Thalamic Nuclei
lh_CeM	Volume of the left CeM	Thalamic Nuclei
lh_CL	Volume of the left CL	Thalamic Nuclei
lh_CM	Volume of the left CM	Thalamic Nuclei
lh_LD	Volume of the left LD	Thalamic Nuclei
lh_LGN	Volume of the left LGN	Thalamic Nuclei
lh_LP	Volume of the left LP	Thalamic Nuclei
lh_L_Sg	Volume of the left L Sg	Thalamic Nuclei
lh_MDl	Volume of the left MDl	Thalamic Nuclei
lh_MDm	Volume of the left MDm	Thalamic Nuclei
lh_MGN	Volume of the left MGN	Thalamic Nuclei
lh_MV_Re_	Volume of the left MV Re	Thalamic Nuclei
lh_Pc	Volume of the left Pc	Thalamic Nuclei
lh_Pf	Volume of the left Pf	Thalamic Nuclei
lh_Pt	Volume of the left Pt	Thalamic Nuclei
lh_PuA	Volume of the left PuA	Thalamic Nuclei
lh_PuI	Volume of the left PuI	Thalamic Nuclei
lh_PuL	Volume of the left PuL	Thalamic Nuclei
lh_PuM	Volume of the left PuM	Thalamic Nuclei
lh_VA	Volume of the left VA	Thalamic Nuclei
lh_VAmc	Volume of the left VAmc	Thalamic Nuclei
lh_VLa	Volume of the left VLa	Thalamic Nuclei
lh_VLp	Volume of the left VLp	Thalamic Nuclei

Variable name in data	Item text	Instrument name
lh_VM	Volume of the left VM	Thalamic Nuclei
lh_VPL	Volume of the left VPL	Thalamic Nuclei
lh_Whole_thalamus	Volume of the left Whole thalamus	Thalamic Nuclei
rh_AV	Volume of the right AV	Thalamic Nuclei
rh_CeM	Volume of the right CeM	Thalamic Nuclei
rh_CL	Volume of the right CL	Thalamic Nuclei
rh_CM	Volume of the right CM	Thalamic Nuclei
rh_LD	Volume of the right LD	Thalamic Nuclei
rh_LGN	Volume of the right LGN	Thalamic Nuclei
rh_LP	Volume of the right LP	Thalamic Nuclei
rh_L_Sg	Volume of the right L Sg	Thalamic Nuclei
rh_MDl	Volume of the right MDl	Thalamic Nuclei
rh_MDm	Volume of the right MDm	Thalamic Nuclei
rh_MGN	Volume of the right MGN	Thalamic Nuclei
rh_MV_Re_	Volume of the right MV Re	Thalamic Nuclei
rh_Pc	Volume of the right Pc	Thalamic Nuclei
rh_Pf	Volume of the right Pf	Thalamic Nuclei
rh_Pt	Volume of the right Pt	Thalamic Nuclei
rh_PuA	Volume of the right PuA	Thalamic Nuclei
rh_PuI	Volume of the right PuI	Thalamic Nuclei
rh_PuL	Volume of the right PuL	Thalamic Nuclei
rh_PuM	Volume of the right PuM	Thalamic Nuclei
rh_VA	Volume of the right VA	Thalamic Nuclei
rh_VAmc	Volume of the right VAmc	Thalamic Nuclei
rh_VLa	Volume of the right VLa	Thalamic Nuclei
rh_VLp	Volume of the right VLp	Thalamic Nuclei
rh_VM	Volume of the right VM	Thalamic Nuclei
rh_VPL	Volume of the right VPL	Thalamic Nuclei
rh_Whole_thalamus	Volume of the right Whole thalamus	Thalamic Nuclei
Medulla	Volume of the Medulla	Brainstem
Pons	Volume of the Pons	Brainstem
SCP	Volume of the SCP	Brainstem
Midbrain	Volume of the Midbrain	Brainstem
Whole_brainstem	Volume of the Whole_brainstem	Brainstem
lh_meanGI_LH_GI	Mean GI in left hemisphere	Local Gyrification Index
lh_bankssts_GI	Gyrification of lh bankssts GI	Local Gyrification Index
lh_caudalanteriorcingulate_GI	Gyrification of lh caudalanteriorcingulate GI	Local Gyrification Index
lh_caudalmiddlefrontal_GI	Gyrification of lh caudalmiddlefrontal GI	Local Gyrification Index
lh_cuneus_GI	Gyrification of lh cuneus GI	Local Gyrification Index
lh_entorhinal_GI	Gyrification of lh entorhinal GI	Local Gyrification Index
lh_fusiform_GI	Gyrification of lh fusiform GI	Local Gyrification Index
lh_inferiorparietal_GI	Gyrification of lh inferiorparietal GI	Local Gyrification Index
lh_inferiortemporal_GI	Gyrification of lh inferiortemporal GI	Local Gyrification Index
lh_isthmuscingulate_GI	Gyrification of lh isthmuscingulate GI	Local Gyrification Index
lh_lateraloccipital_GI	Gyrification of lh lateraloccipital GI	Local Gyrification Index
lh_lateralorbitofrontal_GI	Gyrification of lh lateralorbitofrontal GI	Local Gyrification Index
lh_lingual_GI	Gyrification of lh lingual GI	Local Gyrification Index

Variable name in data	Item text	Instrument name
lh_medialorbitofrontal_GI	Gyrification of lh medialorbitofrontal GI	Local Gyrification Index
lh_middletemporal_GI	Gyrification of lh middletemporal GI	Local Gyrification Index
lh_parahippocampal_GI	Gyrification of lh parahippocampal GI	Local Gyrification Index
lh_paracentral_GI	Gyrification of lh paracentral GI	Local Gyrification Index
lh_parsopercularis_GI	Gyrification of lh parsopercularis GI	Local Gyrification Index
lh_parsorbitalis_GI	Gyrification of lh parsorbitalis GI	Local Gyrification Index
lh_parstriangularis_GI	Gyrification of lh parstriangularis GI	Local Gyrification Index
lh_pericalcarine_GI	Gyrification of lh pericalcarine GI	Local Gyrification Index
lh_postcentral_GI	Gyrification of lh postcentral GI	Local Gyrification Index
lh_posteriorcingulate_GI	Gyrification of lh posteriorcingulate GI	Local Gyrification Index
lh_precentral_GI	Gyrification of lh precentral GI	Local Gyrification Index
lh_precuneus_GI	Gyrification of lh precuneus GI	Local Gyrification Index
lh_rostralanteriorcingulate_GI	Gyrification of lh rostralanteriorcingulate GI	Local Gyrification Index
lh_rostralmiddlefrontal_GI	Gyrification of lh rostralmiddlefrontal GI	Local Gyrification Index
lh_superiorfrontal_GI	Gyrification of lh superiorfrontal GI	Local Gyrification Index
lh_superiorparietal_GI	Gyrification of lh superiorparietal GI	Local Gyrification Index
lh_superiortemporal_GI	Gyrification of lh superiortemporal GI	Local Gyrification Index
lh_supramarginal_GI	Gyrification of lh supramarginal GI	Local Gyrification Index
lh_frontalpole_GI	Gyrification of lh frontalpole GI	Local Gyrification Index
lh_temporalpole_GI	Gyrification of lh temporalpole GI	Local Gyrification Index
lh_transversetemporal_GI	Gyrification of lh transversetemporal GI	Local Gyrification Index
lh_insula_GI	Gyrification of lh insula GI	Local Gyrification Index
rh_meanGI_RH_GI	Mean GI in right hemisphere	Local Gyrification Index
rh_bankssts_GI	Gyrification of rh bankssts GI	Local Gyrification Index
rh_caudalanteriorcingulate_GI	Gyrification of rh caudalanteriorcingulate GI	Local Gyrification Index
rh_caudalmiddlefrontal_GI	Gyrification of rh caudalmiddlefrontal GI	Local Gyrification Index
rh_cuneus_GI	Gyrification of rh cuneus GI	Local Gyrification Index
rh_entorhinal_GI	Gyrification of rh entorhinal GI	Local Gyrification Index
rh_fusiform_GI	Gyrification of rh fusiform GI	Local Gyrification Index
rh_inferiorparietal_GI	Gyrification of rh inferiorparietal GI	Local Gyrification Index
rh_inferiortemporal_GI	Gyrification of rh inferiortemporal GI	Local Gyrification Index
rh_isthmuscingulate_GI	Gyrification of rh isthmuscingulate GI	Local Gyrification Index
rh_lateraloccipital_GI	Gyrification of rh lateraloccipital GI	Local Gyrification Index
rh_lateralorbitofrontal_GI	Gyrification of rh lateralorbitofrontal GI	Local Gyrification Index
rh_lingual_GI	Gyrification of rh lingual GI	Local Gyrification Index

Variable name in data	Item text	Instrument name
rh_medialorbitofrontal_GI	Gyrification of rh medialorbitofrontal GI	Local Gyrification Index
rh_middletemporal_GI	Gyrification of rh middletemporal GI	Local Gyrification Index
rh_parahippocampal_GI	Gyrification of rh parahippocampal GI	Local Gyrification Index
rh_paracentral_GI	Gyrification of rh paracentral GI	Local Gyrification Index
rh_parsopercularis_GI	Gyrification of rh parsopercularis GI	Local Gyrification Index
rh_parsorbitalis_GI	Gyrification of rh parsorbitalis GI	Local Gyrification Index
rh_parstriangularis_GI	Gyrification of rh parstriangularis GI	Local Gyrification Index
rh_pericalcarine_GI	Gyrification of rh pericalcarine GI	Local Gyrification Index
rh_postcentral_GI	Gyrification of rh postcentral GI	Local Gyrification Index
rh_posteriorcingulate_GI	Gyrification of rh posteriorcingulate GI	Local Gyrification Index
rh_precentral_GI	Gyrification of rh precentral GI	Local Gyrification Index
rh_precuneus_GI	Gyrification of rh precuneus GI	Local Gyrification Index
rh_rostralanteriorcingulate_GI	Gyrification of rh rostralanteriorcingulate GI	Local Gyrification Index
rh_rostralmiddlefrontal_GI	Gyrification of rh rostralmiddlefrontal GI	Local Gyrification Index
rh_superiorfrontal_GI	Gyrification of rh superiorfrontal GI	Local Gyrification Index
rh_superiorparietal_GI	Gyrification of rh superiorparietal GI	Local Gyrification Index
rh_superiortemporal_GI	Gyrification of rh superiortemporal GI	Local Gyrification Index
rh_supramarginal_GI	Gyrification of rh supramarginal GI	Local Gyrification Index
rh_frontalpole_GI	Gyrification of rh frontalpole GI	Local Gyrification Index
rh_temporalpole_GI	Gyrification of rh temporalpole GI	Local Gyrification Index
rh_transversetemporal_GI	Gyrification of rh transversetemporal GI	Local Gyrification Index
rh_insula_GI	Gyrification of rh insula GI	Local Gyrification Index
EstimatedTotalIntraCranialVol	Estimated Intracranial Volume	eTIV

Table S3. List of all variables and mode loadings for mode 1

Correlation coefficient (R)	Variable name in data	Item text	Instrument name	Instrument abbreviation (if applicable)
0,905725244	Age	Age	Demographics	Demographics
0,815495281	Physical_Height	Height (in)	Physical Measures	Physical
0,759985709	Physical_Weight	Weight (lbs)	Physical Measures	Physical
0,667199949	WIAT_WIAT_Num_Raw	Numerical Operations Raw Score	Wechsler Individual Achievement Test	WIAT
0,663329856	PreInt_DevHx_puberty	Has your child shown adult sexual body development (puberty)?	Interview-Developmental History	Dev.Hist.
0,662891249	WIAT_WIAT_Spell_Raw	Spelling Raw Score	Wechsler Individual Achievement Test	WIAT
-0,661667058	lh_meanGI_LH_GI	Mean GI in left hemisphere	Local Gyrification Index	NA
0,658863639	CELF_CELF_CriterionScore	CELF-5 Criterion Score	Clinical Evaluation of Language Fundamentals, Fifth Edition	CELF-5
-0,650960876	rh_meanGI_RH_GI	Mean GI in right hemisphere	Local Gyrification Index	NA
0,649105325	WIAT_WIAT_Word_Raw	Word Reading Raw Score	Wechsler Individual Achievement Test	WIAT
-0,633360697	rh_precentral_GI	Gyrification of rh precentral GI	Local Gyrification Index	NA
0,633349132	WIAT_WIAT_MP_Raw	Math Problem Solving Raw Score	Wechsler Individual Achievement Test	WIAT
-0,626874347	lh_precentral_GI	Gyrification of lh precentral GI	Local Gyrification Index	NA
-0,62557232	lh_postcentral_GI	Gyrification of lh postcentral GI	Local Gyrification Index	NA
-0,622214882	rh_postcentral_GI	Gyrification of rh postcentral GI	Local Gyrification Index	NA
-0,590255883	lh_superiorparietal_GI	Gyrification of lh superiorparietal GI	Local Gyrification Index	NA
0,590062862	APQ_P_APQ_P_32	32. Your child is at home without adult supervision	Alabama Parenting Questionnaire	APQ
-0,585819077	lh_paracentral_GI	Gyrification of lh paracentral GI	Local Gyrification Index	NA
0,579042206	WIAT_WIAT_LCRV_Raw	Listening Comprehension Receptive	Wechsler Individual	WIAT

Correlation coefficient (R)	Variable name in data	Item text	Instrument name	Instrument abbreviation (if applicable)
		Vocabulary Raw Score	Achievement Test	
-0,577633206	lh_precuneus_thickness	Cortical thickness in lh precuneus	Cortical Thickness	NA
-0,575380724	rh_paracentral_GI	Gyrification of rh paracentral GI	Local Gyrification Index	NA
-0,573160479	lh_supramarginal_GI	Gyrification of lh supramarginal GI	Local Gyrification Index	NA
-0,572316048	lh_caudalmiddlefrontal_GI	Gyrification of lh caudalmiddlefrontal GI	Local Gyrification Index	NA
-0,571059934	rh_superiorparietal_GI	Gyrification of rh superiorparietal GI	Local Gyrification Index	NA
-0,57099588	rh_inferiorparietal_GI	Gyrification of rh inferiorparietal GI	Local Gyrification Index	NA
-0,570312125	lh_inferiorparietal_GI	Gyrification of lh inferiorparietal GI	Local Gyrification Index	NA
-0,566801212	lh_MeanThickness_thickness	Cortical thickness in lh MeanThickness	Cortical Thickness	NA
-0,563539676	rh_MeanThickness_thickness	Cortical thickness in rh MeanThickness	Cortical Thickness	NA
0,555053367	PreInt_EduHx_imaginativeplay	Imaginative play	Interview-Education and Social History	Edu/Soc.Hist.
-0,552570717	rh_caudalmiddlefrontal_GI	Gyrification of rh caudalmiddlefrontal GI	Local Gyrification Index	NA
-0,550088774	rh_lingual_thickness	Cortical thickness in rh lingual	Cortical Thickness	NA
-0,549262514	rh_supramarginal_GI	Gyrification of rh supramarginal GI	Local Gyrification Index	NA
0,546544694	rh_VM	Volume of the right VM	Thalamic Nuclei	NA
-0,543514152	rh_precuneus_thickness	Cortical thickness in rh precuneus	Cortical Thickness	NA
0,543408939	Medulla	Volume of the Medulla	Brainstem	NA
-0,541592766	lh_superiorparietal_thickness	Cortical thickness in lh superiorparietal	Cortical Thickness	NA
-0,540577964	rh_fusiform_GI	Gyrification of rh fusiform GI	Local Gyrification Index	NA
-0,529968686	lh_fusiform_GI	Gyrification of lh fusiform GI	Local Gyrification Index	NA
-0,529518368	rh_superiorparietal_thickness	Cortical thickness in rh superiorparietal	Cortical Thickness	NA
0,528806371	Physical_BMI	BMI (kg/m^2)	Physical Measures	Physical
-0,524750939	lh_precuneus_volume	Cortical volume in lh precuneus	Cortical Volume	NA
0,524744557	Brain_Stem	Volume of the Brain Stem	Subcortical Volume	NA

Correlation coefficient (R)	Variable name in data	Item text	Instrument name	Instrument abbreviation (if applicable)
0,523750766	PCIAT_PCIAT_07	7. How often does your child check his or her e-mail before doing something else?	Parent-Child Internet Addiction Test	PCIAT
-0,520133045	lh_inferiorparietal_thickness	Cortical thickness in lh inferiorparietal	Cortical Thickness	NA
-0,519295164	lh_lingual_thickness	Cortical thickness in lh lingual	Cortical Thickness	NA
0,514891389	Whole_brainstem	Volume of the Whole_brainstem	Brainstem	NA
-0,513660343	lh_precuneus_GI	Gyrification of lh precuneus GI	Local Gyrification Index	NA
0,508583997	APQ_P_APQ_P_PM	Poor Monitoring/Supervision Score	Alabama Parenting Questionnaire	APQ
-0,500937721	rh_cuneus_thickness	Cortical thickness in rh cuneus	Cortical Thickness	NA
-0,50079527	lh_superiorfrontal_GI	Gyrification of lh superiorfrontal GI	Local Gyrification Index	NA
-0,500323213	lh_superiorparietal_volume	Cortical volume in lh superiorparietal	Cortical Volume	NA
0,499852819	WIAT_WIAT_Pseudo_Raw	Pseudo-word Decoding Raw Score	Wechsler Individual Achievement Test	WIAT
-0,49625353	lh_cuneus_thickness	Cortical thickness in lh cuneus	Cortical Thickness	NA
-0,49492242	rh_middletemporal_GI	Gyrification of rh middletemporal GI	Local Gyrification Index	NA
-0,493428319	rh_precuneus_GI	Gyrification of rh precuneus GI	Local Gyrification Index	NA
-0,493226861	rh_bankssts_GI	Gyrification of rh bankssts GI	Local Gyrification Index	NA
-0,489391766	rh_lateraloccipital_GI	Gyrification of rh lateraloccipital GI	Local Gyrification Index	NA
0,489249833	Midbrain	Volume of the Midbrain	Brainstem	NA
-0,48865578	rh_superiorfrontal_GI	Gyrification of rh superiorfrontal GI	Local Gyrification Index	NA
-0,486749309	rh_posteriorcingulate_GI	Gyrification of rh posteriorcingulate GI	Local Gyrification Index	NA
0,485835869	PCIAT_PCIAT_06	6. How often do your child's grades suffer because of the amount of time he or she spends online?	Parent-Child Internet Addiction Test	PCIAT

Correlation coefficient (R)	Variable name in data	Item text	Instrument name	Instrument abbreviation (if applicable)
-0,485323032	lh_posteriorcingulate_GI	Gyrification of lh posteriorcingulate GI	Local Gyrification Index	NA
-0,484414135	lh_bankssts_GI	Gyrification of lh bankssts GI	Local Gyrification Index	NA
0,484384255	PCIAT_PCIAT_09	9. How often does your child become defensive or secretive when asked what he or she does online?	Parent-Child Internet Addiction Test	PCIAT
-0,484200442	rh_transversetemporal_GI	Gyrification of rh transversetemporal GI	Local Gyrification Index	NA
-0,484055103	lh_rostralmiddlefrontal_thickness	Cortical thickness in lh rostralmiddlefrontal	Cortical Thickness	NA
-0,481508959	rh_inferiortemporal_GI	Gyrification of rh inferiortemporal GI	Local Gyrification Index	NA
0,480311347	PCIAT_PCIAT_11	11. How often does your child spend time along in his or her room playing on the computer?	Parent-Child Internet Addiction Test	PCIAT
-0,479171902	rh_rostralmiddlefrontal_GI	Gyrification of rh rostralmiddlefrontal GI	Local Gyrification Index	NA
-0,478579865	rh_superiortemporal_GI	Gyrification of rh superiortemporal GI	Local Gyrification Index	NA
-0,475582765	lh_lateraloccipital_GI	Gyrification of lh lateraloccipital GI	Local Gyrification Index	NA
-0,474701845	lh_inferiorparietal_volume	Cortical volume in lh inferiorparietal	Cortical Volume	NA
0,472827127	PCIAT_PCIAT_Total	Total Score	Parent-Child Internet Addiction Test	PCIAT
-0,471972657	rh_precuneus_volume	Cortical volume in rh precuneus	Cortical Volume	NA
0,467904706	SCQ_SCQ_35	35. Does she/he play any pretend or make-believe games?	Social Communication Questionnaire	SCQ
-0,467101124	lh_rostralmiddlefrontal_GI	Gyrification of lh rostralmiddlefrontal GI	Local Gyrification Index	NA
-0,464049332	rh_rostralmiddlefrontal_thickness	Cortical thickness in rh rostralmiddlefrontal	Cortical Thickness	NA

Correlation coefficient (R)	Variable name in data	Item text	Instrument name	Instrument abbreviation (if applicable)
-0,461690571	lh_isthmuscingulate_GI	Gyrification of lh isthmuscingulate GI	Local Gyrification Index	NA
-0,46157698	lh_lateralorbitofrontal_thickness	Cortical thickness in lh lateralorbitofrontal	Cortical Thickness	NA
-0,460351631	lh_superiortemporal_GI	Gyrification of lh superiortemporal GI	Local Gyrification Index	NA
-0,459345543	lh_lingual_GI	Gyrification of lh lingual GI	Local Gyrification Index	NA
0,45887781	APQ_SR_APQ_SR_32	32. You are at home without an adult being with you	Alabama Parenting Questionnaire	APQ
-0,458792667	lh_transversetemporal_GI	Gyrification of lh transversetemporal GI	Local Gyrification Index	NA
-0,458235303	rh_inferiorparietal_thickness	Cortical thickness in rh inferiorparietal	Cortical Thickness	NA
-0,452200135	rh_inferiorparietal_volume	Cortical volume in rh inferiorparietal	Cortical Volume	NA
-0,451718654	lh_middletemporal_GI	Gyrification of lh middletemporal GI	Local Gyrification Index	NA
-0,45146428	rh_isthmuscingulate_GI	Gyrification of rh isthmuscingulate GI	Local Gyrification Index	NA
-0,451309713	lh_inferiortemporal_GI	Gyrification of lh inferiortemporal GI	Local Gyrification Index	NA
-0,450893912	APQ_P_APQ_P_11	11. You help your child with his/her homework	Alabama Parenting Questionnaire	APQ
-0,450595618	lh_postcentral_volume	Cortical volume in lh postcentral	Cortical Volume	NA
-0,449151898	rh_superiorparietal_volume	Cortical volume in rh superiorparietal	Cortical Volume	NA
0,44803511	Left_VentralDC	Volume of the Left VentralDC	Subcortical Volume	NA
-0,446942998	lh_lateraloccipital_volume	Cortical volume in lh lateraloccipital	Cortical Volume	NA
0,446128332	PCIAT_PCIAT_04	4. How often does your child form new relationships with fellow online users?	Parent-Child Internet Addiction Test	PCIAT
-0,445678631	rh_parsopercularis_GI	Gyrification of rh parsopercularis GI	Local Gyrification Index	NA

Correlation coefficient (R)	Variable name in data	Item text	Instrument name	Instrument abbreviation (if applicable)
0,445146161	PCIAT_PCIAT_14	14. How often does your child seem more tired and fatigued than he or she did before the Internet came along?	Parent-Child Internet Addiction Test	PCIAT
0,44390598	Pons	Volume of the Pons	Brainstem	NA
-0,443050443	rh_insula_GI	Gyrification of rh insula GI	Local Gyrification Index	NA
0,440615783	Pegboard_peg_time_d	Dominant Hand - Completion time (sec)	Grooved Pegboard	Pegboard
0,437381411	CELF_CELF_Total	CELF-5 Total Score	Clinical Evaluation of Language Fundamentals, Fifth Edition	CELF-5
-0,434701839	lh_cuneus_GI	Gyrification of lh cuneus GI	Local Gyrification Index	NA
-0,431766874	lh_supramarginal_thickness	Cortical thickness in lh supramarginal	Cortical Thickness	NA
0,429708643	APQ_P_APQ_P_21	21. Your child goes out after dark without an adult	Alabama Parenting Questionnaire	APQ
-0,428759442	lh_insula_GI	Gyrification of lh insula GI	Local Gyrification Index	NA
-0,427832913	rh_lingual_GI	Gyrification of rh lingual GI	Local Gyrification Index	NA
-0,426238093	rh_supramarginal_thickness	Cortical thickness in rh supramarginal	Cortical Thickness	NA
0,422989445	WIAT_WIAT_LCODC_Raw	Listening Comprehension Oral Discourse Comprehension Raw Score	Wechsler Individual Achievement Test	WIAT
0,421587681	FGC_FGC_CU	Curl up total	FitnessGram Child	FGC
-0,421082089	lh_rostralmiddlefrontal_volume	Cortical volume in lh rostralmiddlefrontal	Cortical Volume	NA
-0,420170282	rh_medialorbitofrontal_thickness	Cortical thickness in rh medialorbitofrontal	Cortical Thickness	NA
0,4198065	PCIAT_PCIAT_08	8. How often does your child seem withdrawn from others since	Parent-Child Internet Addiction Test	PCIAT

Correlation coefficient (R)	Variable name in data	Item text	Instrument name	Instrument abbreviation (if applicable)
		discovering the Internet?		
-0,418115279	lh_isthmuscingulate_volume	Cortical volume in lh isthmuscingulate	Cortical Volume	NA
-0,417381594	rh_parstriangularis_thickness	Cortical thickness in rh parstriangularis	Cortical Thickness	NA
-0,416342631	rh_parahippocampal_GI	Gyrification of rh parahippocampal GI	Local Gyrification Index	NA
-0,416207873	lh_pericalcarine_GI	Gyrification of lh pericalcarine GI	Local Gyrification Index	NA
-0,415879076	rh_lateraloccipital_volume	Cortical volume in rh lateraloccipital	Cortical Volume	NA
0,414391253	wholeLeft	Volume of the wholeLeft	Hypothalamic Subunits	NA
-0,41284236	rh_lateralorbitofrontal_thickness	Cortical thickness in rh lateralorbitofrontal	Cortical Thickness	NA
-0,412582633	rh_fusiform_thickness	Cortical thickness in rh fusiform	Cortical Thickness	NA
0,411070451	Pegboard_peg_time_nd	Non-dominant Hand - Completion time (sec)	Grooved Pegboard	Pegboard
-0,409494682	lh_superiorfrontal_volume	Cortical volume in lh superiorfrontal	Cortical Volume	NA
-0,409405602	lh_insula_thickness	Cortical thickness in lh insula	Cortical Thickness	NA
0,407582973	Age2	Age squared	Demographics	Demographics
-0,40754265	rh_cuneus_GI	Gyrification of rh cuneus GI	Local Gyrification Index	NA
-0,406388742	rh_postcentral_volume	Cortical volume in rh postcentral	Cortical Volume	NA
-0,406192479	lh_parstriangularis_thickness	Cortical thickness in lh parstriangularis	Cortical Thickness	NA
-0,405575074	lh_parsopercularis_GI	Gyrification of lh parsopercularis GI	Local Gyrification Index	NA
-0,40344661	lh_lateraloccipital_thickness	Cortical thickness in lh lateraloccipital	Cortical Thickness	NA
-0,403409335	rh_parstriangularis_GI	Gyrification of rh parstriangularis GI	Local Gyrification Index	NA
-0,402643843	rh_cuneus_volume	Cortical volume in rh cuneus	Cortical Volume	NA
-0,401684883	lh_lingual_volume	Cortical volume in lh lingual	Cortical Volume	NA
-0,401579235	lh_medialorbitofrontal_thickness	Cortical thickness in lh	Cortical Thickness	NA

Correlation coefficient (R)	Variable name in data	Item text	Instrument name	Instrument abbreviation (if applicable)
		medialorbitofrontal		
-0,401449699	rh_lingual_volume	Cortical volume in rh lingual	Cortical Volume	NA
0,401410636	lh_VM	Volume of the left VM	Thalamic Nuclei	NA
0,400134972	leftTubularSuperior	Volume of the left TubularSuperior	Hypothalamic Subunits	NA
0,399909059	APQ_P_APQ_P_06	6. You child fails to leave a note to let you know where he/she is going	Alabama Parenting Questionnaire	APQ
-0,399399142	lh_superiorfrontal_thickness	Cortical thickness in lh superiorfrontal	Cortical Thickness	NA
-0,398271553	lh_paracentral_thickness	Cortical thickness in lh paracentral	Cortical Thickness	NA
-0,396177576	rh_superiorfrontal_volume	Cortical volume in rh superiorfrontal	Cortical Volume	NA
-0,394014255	lh_cuneus_volume	Cortical volume in lh cuneus	Cortical Volume	NA
-0,392471912	rh_isthmuscingulate_volume	Cortical volume in rh isthmuscingulate	Cortical Volume	NA
0,388698218	APQ_SR_APQ_SR_PM	Poor Monitoring/Supervision Score	Alabama Parenting Questionnaire	APQ
-0,38829733	lh_parahippocampal_GI	Gyrification of lh parahippocampal GI	Local Gyrification Index	NA
-0,387802185	rh_superiorfrontal_thickness	Cortical thickness in rh superiorfrontal	Cortical Thickness	NA
-0,386916309	rh_middletemporal_volume	Cortical volume in rh middletemporal	Cortical Volume	NA
-0,386795177	lh_fusiform_thickness	Cortical thickness in lh fusiform	Cortical Thickness	NA
-0,384967498	rh_rostralmiddlefrontal_volume	Cortical volume in rh rostralmiddlefrontal	Cortical Volume	NA
-0,384081898	lh_lateralorbitofrontal_GI	Gyrification of lh lateralorbitofrontal GI	Local Gyrification Index	NA
0,384040116	rh_VPL	Volume of the right VPL	Thalamic Nuclei	NA
-0,383479863	rh_middletemporal_thickness	Cortical thickness in rh middletemporal	Cortical Thickness	NA
0,37958809	rh_Pf	Volume of the right Pf	Thalamic Nuclei	NA

Correlation coefficient (R)	Variable name in data	Item text	Instrument name	Instrument abbreviation (if applicable)
-0,378818646	rh_lateralorbitofrontal_GI	Gyrification of rh lateralorbitofrontal GI	Local Gyrification Index	NA
-0,377841685	rh_pericalcarine_GI	Gyrification of rh pericalcarine GI	Local Gyrification Index	NA
0,375799215	CTOPP_CTOPP_BW_R	Blending Words raw score	Comprehensive Test of Phonological Processing	CTOPP-2
-0,375733776	rh_lateraloccipital_thickness	Cortical thickness in rh lateraloccipital	Cortical Thickness	NA
0,374737603	PreInt_Demos_Fam_P1_Age	Age	Interview-Demographics/Family	Demog.Fam.
-0,371892799	lh_parsorbitalis_thickness	Cortical thickness in lh parsorbitalis	Cortical Thickness	NA
0,371492851	PCIAT_PCIAT_19	19. How often does your child choose to spend more time online than going out with friends?	Parent-Child Internet Addiction Test	PCIAT
0,371049771	APQ_P_APQ_P_30	30. Your child comes home from school more than an hour past the time you expect him/her to be home	Alabama Parenting Questionnaire	APQ
0,367592259	PCIAT_PCIAT_20	20. How often does your child feel depressed, moody, or nervous when off-line which seems to go away once back online?	Parent-Child Internet Addiction Test	PCIAT
0,366936201	PCIAT_PCIAT_05	5. How often do you complain about the amount of time your child spends online?	Parent-Child Internet Addiction Test	PCIAT
-0,366210633	rh_postcentral_thickness	Cortical thickness in rh postcentral	Cortical Thickness	NA
0,365955788	PCIAT_PCIAT_03	3. How often does your child prefer to spend time online rather than with the rest of your family?	Parent-Child Internet Addiction Test	PCIAT
-0,365444803	lh_parstriangularis_GI	Gyrification of lh parstriangularis GI	Local Gyrification Index	NA
-0,360898322	lh_paracentral_volume	Cortical volume in lh paracentral	Cortical Volume	NA

Correlation coefficient (R)	Variable name in data	Item text	Instrument name	Instrument abbreviation (if applicable)
-0,360766325	lh_postcentral_thickness	Cortical thickness in lh postcentral	Cortical Thickness	NA
0,359082689	PCIAT_PCIAT_15	15. How often does your child seem preoccupied with being back online when off-line?	Parent-Child Internet Addiction Test	PCIAT
0,358671588	PreInt_Demos_Home_fam_01_age	Person 1- age	Interview-Demographic and Household Information	Demog.Household.
-0,357563899	rh_parsorbitalis_thickness	Cortical thickness in rh parsorbitalis	Cortical Thickness	NA
-0,356967561	rh_parsorbitalis_GI	Gyrification of rh parsorbitalis GI	Local Gyrification Index	NA
0,356522148	CTOPP_CTOPP_EL_R	Elision raw score	Comprehensive Test of Phonological Processing	CTOPP-2
-0,356304178	lh_frontalpole_volume	Cortical volume in lh frontalpole	Cortical Volume	NA
0,356002161	Right_Cerebellum_White_Matter	Volume of the Right Cerebellum White Matter	Subcortical Volume	NA
-0,353393343	APQ_SR_APQ_SR_41	41. Your parents use time out (make you sit or stand in a corner) as punishment	Alabama Parenting Questionnaire	APQ
0,351380217	PCIAT_PCIAT_12	12. How often does your child receive strange phone calls from new "online" friends?	Parent-Child Internet Addiction Test	PCIAT
0,351119241	SCQ_SCQ_39	39. Does she/he ever play imaginative games with another child in such a way that you can tell that each child understands what the other is pretending?	Social Communication Questionnaire	SCQ
0,350673013	wholeRight	Volume of the wholeRight	Hypothalamic Subunits	NA
0,350535739	rh_VLp	Volume of the right VLp	Thalamic Nuclei	NA
-0,350511217	rh_superiortemporal_volume	Cortical volume in rh superiortemporal	Cortical Volume	NA

Correlation coefficient (R)	Variable name in data	Item text	Instrument name	Instrument abbreviation (if applicable)
-0,350340154	APQ_P_APQ_P_41	41. You use time out him/her sit or stand in a corner) as punishment	Alabama Parenting Questionnaire	APQ
-0,350265892	rh_posteriorcingulate_thickness	Cortical thickness in rh posteriorcingulate	Cortical Thickness	NA
0,345153715	PCIAT_PCIAT_17	17. How often does your child choose to spend time online rather than doing once enjoyed hobbies and/or outside interests?	Parent-Child Internet Addiction Test	PCIAT
-0,344513254	rh_frontalpole_volume	Cortical volume in rh frontalpole	Cortical Volume	NA
-0,343063677	lh_supramarginal_volume	Cortical volume in lh supramarginal	Cortical Volume	NA
-0,339290478	lh_middletemporal_thickness	Cortical thickness in lh middletemporal	Cortical Thickness	NA
-0,339125225	rh_paracentral_thickness	Cortical thickness in rh paracentral	Cortical Thickness	NA
-0,33829322	lh_middletemporal_volume	Cortical volume in lh middletemporal	Cortical Volume	NA
-0,337551646	rh_posteriorcingulate_volume	Cortical volume in rh posteriorcingulate	Cortical Volume	NA
-0,337383344	rh_medialorbitofrontal_volume	Cortical volume in rh medialorbitofrontal	Cortical Volume	NA
-0,336405461	lh_posteriorcingulate_volume	Cortical volume in lh posteriorcingulate	Cortical Volume	NA
-0,33580875	lh_posteriorcingulate_thickness	Cortical thickness in lh posteriorcingulate	Cortical Thickness	NA
0,335560134	leftTubularInferior	Volume of the left TubularInferior	Hypothalamic Subunits	NA
0,334821695	PCIAT_PCIAT_02	2. How often does your child neglect household chores to spend more time online?	Parent-Child Internet Addiction Test	PCIAT
-0,33371059	rh_isthmuscingulate_thickness	Cortical thickness in rh isthmuscingulate	Cortical Thickness	NA
-0,332633638	rh_bankssts_thickness	Cortical thickness in rh bankssts	Cortical Thickness	NA
0,33220168	SCP	Volume of the SCP	Brainstem	NA

Correlation coefficient (R)	Variable name in data	Item text	Instrument name	Instrument abbreviation (if applicable)
-0,331836434	lh_frontalpole_thickness	Cortical thickness in lh frontalpole	Cortical Thickness	NA
-0,331003034	rh_pericalcarine_thickness	Cortical thickness in rh pericalcarine	Cortical Thickness	NA
0,330721455	lh_Pf	Volume of the left Pf	Thalamic Nuclei	NA
-0,330664998	lh_pericalcarine_thickness	Cortical thickness in lh pericalcarine	Cortical Thickness	NA
0,330498526	rightTubularInferior	Volume of the right TubularInferior	Hypothalamic Subunits	NA
-0,328761139	rh_transversetemporal_volume	Cortical volume in rh transversetemporal	Cortical Volume	NA
-0,328136703	rh_transversetemporal_thickness	Cortical thickness in rh transversetemporal	Cortical Thickness	NA
0,327036793	Left_Cerebellum_White_Matter	Volume of the Left Cerebellum White Matter	Subcortical Volume	NA
-0,326325127	rh_parsorbitalis_volume	Cortical volume in rh parsorbitalis	Cortical Volume	NA
-0,32533729	lh_parsopercularis_thickness	Cortical thickness in lh parsopercularis	Cortical Thickness	NA
-0,325310032	lh_parsorbitalis_volume	Cortical volume in lh parsorbitalis	Cortical Volume	NA
-0,322582334	lh_isthmuscingulate_thickness	Cortical thickness in lh isthmuscingulate	Cortical Thickness	NA
-0,322126662	lh_caudalanteriorcingulate_GI	Gyrification of lh caudalanteriorcingulate GI	Local Gyrification Index	NA
0,321316219	CTOPP_CTOPP_NR_R	Nonword Repetition raw score	Comprehensive Test of Phonological Processing	CTOPP-2
0,321173396	APQ_SR_APQ_SR_06	6. You fail to leave a note or let your parents know where you are going	Alabama Parenting Questionnaire	APQ
0,320629996	PCIAT_PCIAT_10	10. How often have you caught your child sneaking online against your wishes?	Parent-Child Internet Addiction Test	PCIAT
-0,320563412	lh_bankssts_thickness	Cortical thickness in lh bankssts	Cortical Thickness	NA
-0,320240065	lh_transversetemporal_thickness	Cortical thickness in lh transversetemporal	Cortical Thickness	NA

Correlation coefficient (R)	Variable name in data	Item text	Instrument name	Instrument abbreviation (if applicable)
-0,319951593	rh_paracentral_volume	Cortical volume in rh paracentral	Cortical Volume	NA
0,318847172	rh_VLa	Volume of the right VLa	Thalamic Nuclei	NA
-0,318484169	rh_superiortemporal_thickness	Cortical thickness in rh superiortemporal	Cortical Thickness	NA
-0,317675286	lh_lateralorbitofrontal_volume	Cortical volume in lh lateralorbitofrontal	Cortical Volume	NA
-0,317153823	lh_medialorbitofrontal_volume	Cortical volume in lh medialorbitofrontal	Cortical Volume	NA
0,316389348	CC_Posterior	Volume of the CC Posterior	Subcortical Volume	NA
-0,315293265	rh_rostralanteriorcingulate_thickness	Cortical thickness in rh rostralanteriorcingulate	Cortical Thickness	NA
-0,315292199	lh_inferiortemporal_thickness	Cortical thickness in lh inferiortemporal	Cortical Thickness	NA
0,31461034	PreInt_EduHx_crafts	Drawing/painting/crafts	Interview-Education and Social History	Edu/Soc.Hist.
-0,313908134	rh_caudalanteriorcingulate_GI	Gyrification of rh caudalanteriorcingulate GI	Local Gyrification Index	NA
0,313656142	CBCL_CBCL_WD	Withdrawn/Depressed Raw Score	Child Behavior Checklist	CBCL
-0,310220877	lh_parsorbitalis_GI	Gyrification of lh parsorbitalis GI	Local Gyrification Index	NA
-0,309661376	rh_entorhinal_GI	Gyrification of rh entorhinal GI	Local Gyrification Index	NA
0,306769965	APQ_P_APQ_P_10	10. Your child stays out in the evening past the time that he/she is supposed to be home	Alabama Parenting Questionnaire	APQ
0,305186974	ICU_P_ICU_P_Unemotional	Unemotional Subscale Score	Inventory of Callous-Unemotional Traits Parent	ICU
-0,303583254	lh_superiortemporal_volume	Cortical volume in lh superiortemporal	Cortical Volume	NA
-0,301680659	rh_insula_thickness	Cortical thickness in rh insula	Cortical Thickness	NA
-0,298951838	rh_bankssts_volume	Cortical volume in rh bankssts	Cortical Volume	NA
-0,297894249	rh_temporalpole_GI	Gyrification of rh temporalpole GI	Local Gyrification Index	NA

Correlation coefficient (R)	Variable name in data	Item text	Instrument name	Instrument abbreviation (if applicable)
-0,296867239	SWAN_SWAN_11	11. Stays seated (when required by class rules or social conventions)	The Strengths and Weaknesses Assessment of Normal Behavior	SWAN
0,294789739	rh_CM	Volume of the right CM	Thalamic Nuclei	NA
-0,29383133	SWAN_SWAN_10	10. Sits still (controls movement of hands or feet or controls squirming)	The Strengths and Weaknesses Assessment of Normal Behavior	SWAN
-0,293614117	lh_parstriangularis_volume	Cortical volume in lh parstriangularis	Cortical Volume	NA
-0,293316142	lh_rostralanteriorcingulate_thickness	Cortical thickness in lh rostralanteriorcingulate	Cortical Thickness	NA
-0,29221553	rh_parstriangularis_volume	Cortical volume in rh parstriangularis	Cortical Volume	NA
-0,289871373	rh_fusiform_volume	Cortical volume in rh fusiform	Cortical Volume	NA
-0,288644093	rh_inferiortemporal_thickness	Cortical thickness in rh inferiortemporal	Cortical Thickness	NA
0,288529723	APQ_P_APQ_P_17	17. You do not know the friends your child is with	Alabama Parenting Questionnaire	APQ
0,286895409	SCQ_SCQ_34	34. Does she/he ever spontaenously join in and try to copy the actions in social games, such as The Mulberry Bush or London Bridge is Falling Down?	Social Communication Questionnaire	SCQ
-0,286581228	rh_supramarginal_volume	Cortical volume in rh supramarginal	Cortical Volume	NA
0,285578715	PCIAT_PCIAT_13	13. How often does your child snap, yell, or act annoyed if bothered while online?	Parent-Child Internet Addiction Test	PCIAT
-0,283956063	rh_lateralorbitofrontal_volume	Cortical volume in rh lateralorbitofrontal	Cortical Volume	NA
-0,283339813	SympChck_CSC_36C	Has difficulty remaining seated	Symptom Checklist Parent report	SympChck-P

Correlation coefficient (R)	Variable name in data	Item text	Instrument name	Instrument abbreviation (if applicable)
		at home or school (current)		
-0,281861143	lh_MGN	Volume of the left MGN	Thalamic Nuclei	NA
-0,281119803	rh_parsoperculari s_thickness	Cortical thickness in rh parsopercularis	Cortical Thickness	NA
0,280555411	Physical_Systolic _BP	Systolic BP (mmHg)	Physical Measures	Physical
0,279023763	NLES_P_NLES_ P_Aware	# of Negative Events the Child was aware of	Negative Life Events Scale	NLES
-0,278018065	CBCL_CBCL_10	10. Can't sit still, restless or hyperactive	Child Behavior Checklist	CBCL
-0,276259386	lh_fusiform_volu me	Cortical volume in lh fusiform	Cortical Volume	NA
-0,275234733	lh_lateraloccipital _area	Cortical area in lh lateraloccipital	Cortical Area	NA
-0,27492272	APQ_P_APQ_P_ 07	7. You play games or do other fun things with your child	Alabama Parenting Questionnaire	APQ
0,273939447	APQ_SR_APQ_ SR_30	30. You come home from school more than an hour past the time your parents expect you to be home	Alabama Parenting Questionnaire	APQ
-0,273179402	rh_frontalpole_thi ckness	Cortical thickness in rh frontalpole	Cortical Thickness	NA
-0,272213049	rh_WhiteSurfAre a_area	Cortical area in rh WhiteSurfArea	Cortical Area	NA
0,270894631	lh_VLp	Volume of the left VLp	Thalamic Nuclei	NA
-0,270399049	SWAN_SWAN_H Y	Hyperactivity Average	The Strengths and Weaknesses Assessment of Normal Behavior	SWAN
-0,267752806	lh_entorhinal_GI	Gyrification of lh entorhinal GI	Local Gyrification Index	NA
0,267588522	CBCL_CBCL_54	54. Overtired without good reason	Child Behavior Checklist	CBCL
-0,267586143	SDQ_SDQ_02	Restless, overactive, cannot stay still for long	Strength and Difficulties Questionnaire	SDQ
-0,266656862	SympChck_CSC _17C	Often has trouble going to sleep without parent/guardian nearby (current)	Symptom Checklist Parent report	SympChck-P
0,264504869	right_Whole_hipp ocampal_body	Volume of the right Whole	Hippocampus Subfields	NA

Correlation coefficient (R)	Variable name in data	Item text	Instrument name	Instrument abbreviation (if applicable)
		hippocampal body		
0,263857608	CC_Mid_Posterior	Volume of the CC Mid Posterior	Subcortical Volume	NA
0,261985319	SRS_SRS_60	60. Is emotionally distant, doesn't show his or her feelings.	Social Responsiveness Scale	SRS
0,261705554	right_subiculum_body	Volume of the right subiculum body	Hippocampus Subfields	NA
-0,261543427	SWAN_SWAN_12	12. Modulates motor activity (inhibits inappropriate running or climbing)	The Strengths and Weaknesses Assessment of Normal Behavior	SWAN
-0,259045298	rh_lateraloccipital_area	Cortical area in rh lateraloccipital	Cortical Area	NA
-0,257553679	lh_bankssts_volume	Cortical volume in lh bankssts	Cortical Volume	NA
-0,25749976	lh_temporalpole_GI	Gyrification of lh temporalpole GI	Local Gyrification Index	NA
-0,25721707	lh_parsopercularis_volume	Cortical volume in lh parsopercularis	Cortical Volume	NA
0,256251361	NLES_P_NLES_P_Upset_Total	Child's Total Upsetness/Negative Events	Negative Life Events Scale	NLES
0,256236069	ICU_P_ICU_P_14	14. It is easy to tell how he/she is feeling.	Inventory of Callous-Unemotional Traits Parent	ICU
-0,255952075	lh_superiortemporal_thickness	Cortical thickness in lh superiortemporal	Cortical Thickness	NA
-0,255870862	lh_medialorbitofrontal_GI	Gyrification of lh medialorbitofrontal GI	Local Gyrification Index	NA
0,254709804	APQ_P_APQ_P_28	28. You don't check that your child comes home at the time he/she was supposed to	Alabama Parenting Questionnaire	APQ
-0,254646746	lh_WhiteSurfArea_area	Cortical area in lh WhiteSurfArea	Cortical Area	NA
-0,254263584	rh_L_Sg	Volume of the right L Sg	Thalamic Nuclei	NA
0,252849421	rh_Whole_thalamus	Volume of the right Whole thalamus	Thalamic Nuclei	NA
-0,251792338	SWAN_SWAN_14	14. Settles down and rests (controls excessive talking)	The Strengths and Weaknesses Assessment of Normal Behavior	SWAN

Correlation coefficient (R)	Variable name in data	Item text	Instrument name	Instrument abbreviation (if applicable)
0,250551527	left_subiculum_body	Volume of the left subiculum body	Hippocampus Subfields	NA
0,249722702	CBCL_CBCL_42	42. Would rather be along than with others	Child Behavior Checklist	CBCL
0,248310295	left_Whole_hippocampal_body	Volume of the left Whole hippocampal body	Hippocampus Subfields	NA
0,247665212	ICU_P_ICU_P_19	19. Is very expressive and emotional.	Inventory of Callous-Unemotional Traits Parent	ICU
-0,24678243	lh_transversetemporal_volume	Cortical volume in lh transversetemporal	Cortical Volume	NA
-0,246463233	rh_parsopercularis_volume	Cortical volume in rh parsopercularis	Cortical Volume	NA
0,244511719	ICU_P_ICU_P_22	22. Hides his/her feelings from others.	Inventory of Callous-Unemotional Traits Parent	ICU
-0,243531738	lh_pericalcarine_volume	Cortical volume in lh pericalcarine	Cortical Volume	NA
-0,243493546	SCARED_P_SCARED_P_SP	Separation Anxiety SOC Score	Screen for Child Anxiety Related Disorders	SCARED
0,243470547	SCQ_SCQ_Total	Total Score	Social Communication Questionnaire	SCQ
0,242618999	leftAnterior_superior	Volume of the left Anterior superior	Hypothalamic Subunits	NA
-0,241267793	APQ_SR_APQ_SR_11	11. Your mom helps you with your homework	Alabama Parenting Questionnaire	APQ
-0,240567019	rh_inferiorparietal_area	Cortical area in rh inferiorparietal	Cortical Area	NA
0,240249289	SRS_SRS_06	6. Would rather be alone than with others.	Social Responsiveness Scale	SRS
0,239905558	right_Whole_amygdala	Volume of the right Whole amygdala	Amygdala Nuclei	NA
0,239891425	SRS_SRS_27	27. Avoids starting social interactions with peers or adults.	Social Responsiveness Scale	SRS
0,239787816	SCQ_SCQ_21	21. Does she/he ever spontaneously copy you (or other people) or what you are doing (such as	Social Communication Questionnaire	SCQ

Correlation coefficient (R)	Variable name in data	Item text	Instrument name	Instrument abbreviation (if applicable)
		vacuuming, gardening, or mending things)?		
0,239582514	lh_VLa	Volume of the left VLa	Thalamic Nuclei	NA
-0,239338205	rh_caudalanteriorcingulate_thickness	Cortical thickness in rh caudalanteriorcingulate	Cortical Thickness	NA
-0,239049243	lh_L_Sg	Volume of the left L Sg	Thalamic Nuclei	NA
0,238952992	SRS_SRS_40	40. Is imaginative, good at pretending (without losing touch with reality).	Social Responsiveness Scale	SRS
0,238781919	APQ_P_APQ_P_19	19. Your child goed out with a set time to be home	Alabama Parenting Questionnaire	APQ
0,237972377	right_Corticoamygdaloid_transitio	Volume of the right Corticoamygdaloid transitio	Amygdala Nuclei	NA
0,237969393	PreInt_TxHx_Past_DX	Has your chid ever been diagnosed with a psychiatric or learning disorder?	Interview-Treatment History	Treat.hist.
-0,237584202	lh_inferiorparietal_area	Cortical area in lh inferiorparietal	Cortical Area	NA
0,236655429	left_Whole_amygdala	Volume of the left Whole amygdala	Amygdala Nuclei	NA
0,236653685	SCQ_SCQ_22	22. Does she/he ever spontaneously point at things around her/him just to show you things (not because she/he wants them)?	Social Communication Questionnaire	SCQ
0,2365253	left_molecular_layer_HP_body	Volume of the left molecular layer HP body	Hippocampus Subfields	NA
-0,235661315	SympChck_CSC_36P	Has difficulty remaining seated at home or school (past	Symptom Checklist Parent report	SympChck-P
-0,235153641	Pegboard_peg_z_d	Dominant Hand - z-score	Grooved Pegboard	Pegboard
0,235129562	left_Lateral_nucleus	Volume of the left Lateral nucleus	Amygdala Nuclei	NA

Correlation coefficient (R)	Variable name in data	Item text	Instrument name	Instrument abbreviation (if applicable)
0,23410014	Right_Thalamus	Volume of the Right Thalamus	Subcortical Volume	NA
0,233927795	right_Accessory_Basal_nucleus	Volume of the right Accessory Basal nucleus	Amygdala Nuclei	NA
-0,232990371	lh_postcentral_area	Cortical area in lh postcentral	Cortical Area	NA
0,232965996	right_molecular_layer_HP_body	Volume of the right molecular layer HP body	Hippocampus Subfields	NA
0,232055219	SympChck_CSC_01P	Feels sad and down most days for at least 1 week (past	Symptom Checklist Parent report	SympChck-P
0,231751939	PCIAT_PCIAT_18	18. How often does your child become angry or belligerent when your place time limits on how much time he or shes is allowed to spend online?	Parent-Child Internet Addiction Test	PCIAT
0,229760217	lh_CM	Volume of the left CM	Thalamic Nuclei	NA
0,229687095	PreInt_EduHx_dancing	Dancing	Interview-Education and Social History	Edu/Soc.Hist.
-0,228277311	lh_superiorparietal_area	Cortical area in lh superiorparietal	Cortical Area	NA
0,227996782	lh_VPL	Volume of the left VPL	Thalamic Nuclei	NA
0,227852135	SympChck_CSC_24P	Often misses school or other activities because he/she doesn't feel well (past	Symptom Checklist Parent report	SympChck-P
0,227424402	right_Whole_hippocampus	Volume of the right Whole hippocampus	Hippocampus Subfields	NA
0,226851112	CBCL_CBCL_69	69. Secretive, keeps things to self	Child Behavior Checklist	CBCL
0,22663654	APQ_SR_APQ_SR_28	28. You stay out later than you are supposed to and your parents don't know it	Alabama Parenting Questionnaire	APQ
0,226513317	CBCL_CBCL_111	111. Withdrawn, doesn't get inolved with others	Child Behavior Checklist	CBCL
0,226142615	PreInt_EduHx_tutor	Has your child had tutoring outside of school?	Interview-Education and Social History	Edu/Soc.Hist.

Correlation coefficient (R)	Variable name in data	Item text	Instrument name	Instrument abbreviation (if applicable)
-0,225790279	rh_medialorbitofrontal_GI	Gyrification of rh medialorbitofrontal GI	Local Gyrification Index	NA
-0,223290385	SDQ_SDQ_10	Constantly fidgeting or squirming	Strength and Difficulties Questionnaire	SDQ
0,222995172	left_Whole_hippocampus	Volume of the left Whole hippocampus	Hippocampus Subfields	NA
0,222805564	APQ_SR_APQ_SR_10	10. You stay out in the evening past the time you are supposed to be home	Alabama Parenting Questionnaire	APQ
-0,222445134	lh_precuneus_area	Cortical area in lh precuneus	Cortical Area	NA
-0,222035707	rh_pericalcarine_volume	Cortical volume in rh pericalcarine	Cortical Volume	NA
0,221067532	Left_Thalamus	Volume of the Left Thalamus	Subcortical Volume	NA
0,220228582	PreInt_EduHx_weakness_math	Math	Interview- Education and Social History	Edu/Soc.Hist.
-0,219868649	rh_caudalmiddlefrontal_volume	Cortical volume in rh caudalmiddlefrontal	Cortical Volume	NA
0,219678471	right_GC_ML_DG_body	Volume of the right GC ML DG body	Hippocampus Subfields	NA
-0,218956203	lh_inferiortemporal_volume	Cortical volume in lh inferiortemporal	Cortical Volume	NA
0,218818241	rightPosterior	Volume of the right Posterior	Hypothalamic Subunits	NA
-0,218477887	lh_caudalmiddlefrontal_thickness	Cortical thickness in lh caudalmiddlefrontal	Cortical Thickness	NA
0,218446527	APQ_SR_APQ_SR_19	19. You go out with a set time to be home	Alabama Parenting Questionnaire	APQ
0,216649419	left_GC_ML_DG_body	Volume of the left GC ML DG body	Hippocampus Subfields	NA
0,216418232	SCQ_SCQ_36	36. Does she/he seem interested in other children of approximately the same age whom she/he does not know?	Social Communication Questionnaire	SCQ
0,215823458	rightAnterior_superior	Volume of the right Anterior superior	Hypothalamic Subunits	NA
-0,215784055	rh_MGN	Volume of the right MGN	Thalamic Nuclei	NA

Correlation coefficient (R)	Variable name in data	Item text	Instrument name	Instrument abbreviation (if applicable)
-0,215528969	CBCL_CBCL_19	19. Demands a lot of attention	Child Behavior Checklist	CBCL
0,215194745	right_HATA	Volume of the right HATA	Hippocampus Subfields	NA
-0,21487462	rh_caudalmiddlefrontal_thickness	Cortical thickness in rh caudalmiddlefrontal	Cortical Thickness	NA
0,214219767	CC_Central	Volume of the CC Central	Subcortical Volume	NA
0,214162977	CBCL_CBCL_WD_T	Withdrawn/Depressed T Score	Child Behavior Checklist	CBCL
0,213923661	NLES_P_NLES_P_TotalEvents	Total # of Negative Events	Negative Life Events Scale	NLES
0,213714168	left_HATA	Volume of the left HATA	Hippocampus Subfields	NA
-0,213676692	lh_precentral_volume	Cortical volume in lh precentral	Cortical Volume	NA
0,21351308	APQ_SR_APQ_SR_17	17. Your parents do not know the friends you are with	Alabama Parenting Questionnaire	APQ
-0,212625304	lh_rostralanteriorcingulate_GI	Gyrification of lh rostralanteriorcingulate GI	Local Gyrification Index	NA
-0,212448806	Left_Accumbens_area	Volume of the Left Accumbens area	Subcortical Volume	NA
0,212432785	CBCL_CBCL_Int	Internalizing Raw Score	Child Behavior Checklist	CBCL
-0,212257932	SWAN_SWAN_13	13. Plays quietly (keeps noise level reasonable)	The Strengths and Weaknesses Assessment of Normal Behavior	SWAN
0,212173945	right_Lateral_nucleus	Volume of the right Lateral nucleus	Amygdala Nuclei	NA
0,211160576	NLES_P_NLES_P_16a	16a. A close family member to the child died such as a parent, close uncle, grandparent, or some other relative.	Negative Life Events Scale	NLES
0,210550375	left_Corticoamygdaloid_transitio	Volume of the left Corticoamygdaloid transitio	Amygdala Nuclei	NA
0,210257911	SympChck_CSC_03P	Has a loss of interest in previously enjoyable activities (past	Symptom Checklist Parent report	SympChck-P
0,20985349	ICU_P_ICU_P_06	6. Does not show emotions.	Inventory of Callous-	ICU

Correlation coefficient (R)	Variable name in data	Item text	Instrument name	Instrument abbreviation (if applicable)
			Unemotional Traits Parent	
0,209428539	right_Basal_nucleus	Volume of the right Basal nucleus	Amygdala Nuclei	NA
0,209219003	SRS_SRS_34	34. Avoids people who want to be emotionally close to him or her.	Social Responsiveness Scale	SRS
0,208908142	SDQ_SDQ_06	Rather solitary, prefers to play alone (for 11-17 year olds: Would rather be alone than with other youth)	Strength and Difficulties Questionnaire	SDQ
0,20867636	rightTubularSuperior	Volume of the right TubularSuperior	Hypothalamic Subunits	NA
0,208560381	CBCL_CBCL_56B	56B. Headaches	Child Behavior Checklist	CBCL
0,207248529	left_CA4_body	Volume of the left CA4 body	Hippocampus Subfields	NA
-0,206855313	lh_caudalmiddlefrontal_volume	Cortical volume in lh caudalmiddlefrontal	Cortical Volume	NA
-0,205995039	FGC_FGC_SRL_Zone	Sit & Reach fitness zone (left side)	FitnessGram Child	FGC
0,205789926	NLES_P_NLES_P_21a	21a. The child changed schools.	Negative Life Events Scale	NLES
0,205386423	CBCL_CBCL_103	103. Unhappy, sad, or depressed	Child Behavior Checklist	CBCL
-0,205318817	SympChck_CSC_18C	Is afraid of being alone at home or in a different room than parent/guardian (current)	Symptom Checklist Parent report	SympChck-P
0,205245906	APQ_SR_APQ_SR_14	14. Your mom asks you what your plans are for the coming day	Alabama Parenting Questionnaire	APQ
0,203963861	SympChck_CSC_42C	Has skipped a part or a whole day of school (current)	Symptom Checklist Parent report	SympChck-P
0,203371206	left_Accessory_Basal_nucleus	Volume of the left Accessory Basal nucleus	Amygdala Nuclei	NA
0,202322255	CBCL_CBCL_102	102. Underactive, slow moving, or lacks energy	Child Behavior Checklist	CBCL

Correlation coefficient (R)	Variable name in data	Item text	Instrument name	Instrument abbreviation (if applicable)
-0,201602782	rh_parahippocampal_thickness	Cortical thickness in rh parahippocampal	Cortical Thickness	NA
0,201581124	rh_Pc	Volume of the right Pc	Thalamic Nuclei	NA
0,201007577	SCQ_SCQ_30	30. Does she/he ever seem to want you to join in her/his enjoyment of something?	Social Communication Questionnaire	SCQ
0,199791031	left_Basal_nucleus	Volume of the left Basal nucleus	Amygdala Nuclei	NA
-0,198258869	APQ_SR_APQ_SR_18	18. Your parents hug or kiss you when you have done something well	Alabama Parenting Questionnaire	APQ
0,197737762	right_CA4_body	Volume of the right CA4 body	Hippocampus Subfields	NA
-0,197655187	APQ_P_APQ_P_37	37. You send your child to his/her room as punishment	Alabama Parenting Questionnaire	APQ
0,197566102	PreInt_EduHx_strength_history	Social studies/history	Interview-Education and Social History	Edu/Soc.Hist.
0,196135455	PreInt_EduHx_weakness_science	Science	Interview-Education and Social History	Edu/Soc.Hist.
-0,195875968	lh_caudalanteriorcingulate_thickness	Cortical thickness in lh caudalanteriorcingulate	Cortical Thickness	NA
-0,195747103	lh_isthmuscingulate_area	Cortical area in lh isthmuscingulate	Cortical Area	NA
-0,194961745	WIAT_WIAT_LCODC_P	Listening Comprehension Oral Discourse Comprehension Percentile Rank	Wechsler Individual Achievement Test	WIAT
0,19465113	SRS_SRS_23	23. Does not join group activities unless told to do so.	Social Responsiveness Scale	SRS
0,194377793	CBCL_CBCL_05	5. There is very little he/she enjoys	Child Behavior Checklist	CBCL
-0,19392706	SRS_SRS_55	55. Knows when he or she is talking too loud or making too much noise.	Social Responsiveness Scale	SRS
-0,193771216	CTOPP_CTOPP_RL_P	Rapid Letter Naming percentile score	Comprehensive Test of	CTOPP-2

Correlation coefficient (R)	Variable name in data	Item text	Instrument name	Instrument abbreviation (if applicable)
			Phonological Processing	
0,193710373	leftAnterior_inferior	Volume of the left Anterior inferior	Hypothalamic Subunits	NA
-0,19348828	WIAT_WIAT_LCODC_Stnd	Listening Comprehension Oral Discourse Comprehension Standard Score	Wechsler Individual Achievement Test	WIAT
0,192289163	SDQ_SDQ_Internalizing	Internalizing Score	Strength and Difficulties Questionnaire	SDQ
-0,192111589	FGC_FGC_SRR_Zone	Sit & Reach fitness zone (right side)	FitnessGram Child	FGC
0,191798542	x3rd_Ventricle	Volume of the x3rd Ventricle	Subcortical Volume	NA
-0,191795789	rh_superiorparietal_area	Cortical area in rh superiorparietal	Cortical Area	NA
0,191399837	SRS_SRS_MOT_T	Social Motivation T-Score	Social Responsiveness Scale	SRS
0,191349401	SympChck_CSC_30P	Feels extremely worried about gaining weight or becoming fat (past	Symptom Checklist Parent report	SympChck-P
0,190729934	CBCL_CBCL_77	77. Sleeps more than most kids during day and/or night	Child Behavior Checklist	CBCL
0,190435888	SDQ_SDQ_Peer_Problems	Peer Problems Scale	Strength and Difficulties Questionnaire	SDQ
0,190326518	Left_Amygdala	Volume of the Left Amygdala	Subcortical Volume	NA
0,189745712	SRS_SRS_MOT	Social Motivation Raw Score	Social Responsiveness Scale	SRS
-0,1894781	SympChck_CSC_06C	Has periods of unusual energy and activity (current)	Symptom Checklist Parent report	SympChck-P
0,18899386	PreInt_EduHx_learning_disability	Were any learning disabilities identified?	Interview-Education and Social History	Edu/Soc.Hist.
-0,188517567	CTOPP_CTOPP_RL_S	Rapid Letter Naming scaled score	Comprehensive Test of Phonological Processing	CTOPP-2
0,188015096	SRS_SRS_39	39. Has an unusally narrow range of interests.	Social Responsiveness Scale	SRS

Correlation coefficient (R)	Variable name in data	Item text	Instrument name	Instrument abbreviation (if applicable)
-0,188007285	APQ_SR_APQ_SR_PP	Positive Parenting Score	Alabama Parenting Questionnaire	APQ
-0,187848005	rh_transversetemporal_area	Cortical area in rh transversetemporal	Cortical Area	NA
-0,186490343	SRS_SRS_56	56. Walks in between two people who are talking.	Social Responsiveness Scale	SRS
0,186174101	NLES_P_NLES_P_Upset_Avg	Child's Average Upsetness/Negative Events	Negative Life Events Scale	NLES
0,186131328	Physical_Diastolic_BP	Diastolic BP (mmHg)	Physical Measures	Physical
0,185345652	SympChck_CSC_42P	Has skipped a part or a whole day of school (past	Symptom Checklist Parent report	SympChck-P
-0,185190535	SWAN_SWAN_17	17. Awaits turn (stands in line and takes turns)	The Strengths and Weaknesses Assessment of Normal Behavior	SWAN
0,185159727	FGC_FGC_TL	Trunk lift total	FitnessGram Child	FGC
0,185065752	PCIAT_PCIAT_01	1. How often does your child disobey time limits you set for online use?	Parent-Child Internet Addiction Test	PCIAT
0,183564388	SRS_SRS_64	64. Is too tense in social settings.	Social Responsiveness Scale	SRS
0,183482225	SympChck_CSC_03C	Has a loss of interest in previously enjoyable activities (current)	Symptom Checklist Parent report	SympChck-P
-0,183279849	APQ_P_APQ_P_OPD	Other Discipline Practices Score (Not factored into total score but provides item level information)	Alabama Parenting Questionnaire	APQ
-0,18324224	rh_inferiortemporal_volume	Cortical volume in rh inferiortemporal	Cortical Volume	NA
0,182657393	APQ_P_APQ_P_14	14. You ask your child what his/her plans are for the coming day	Alabama Parenting Questionnaire	APQ
0,182416873	CBCL_CBCL_51	51. Feels dizzy or lightheaded	Child Behavior Checklist	CBCL
-0,182275045	SDQ_SDQ_Hyperactivity	Hyperactivity Scale	Strength and Difficulties Questionnaire	SDQ

Correlation coefficient (R)	Variable name in data	Item text	Instrument name	Instrument abbreviation (if applicable)
-0,182146426	lh_parahippocampal_thickness	Cortical thickness in lh parahippocampal	Cortical Thickness	NA
0,181658591	PreInt_EduHx_reading	Books/reading	Interview-Education and Social History	Edu/Soc.Hist.
-0,18122903	rh_precuneus_area	Cortical area in rh precuneus	Cortical Area	NA
0,181131387	SCQ_SCQ_29	29. Does she/he ever offer to share things other than food with you?	Social Communication Questionnaire	SCQ
-0,181055183	APQ_P_APQ_P_INV	Involvement Score	Alabama Parenting Questionnaire	APQ
0,180426248	Right_Hippocampus	Volume of the Right Hippocampus	Subcortical Volume	NA
0,179651031	CBCL_CBCL_SC_T	Somatic Complaints T Score	Child Behavior Checklist	CBCL
0,178920755	SympChck_CSC_24C	Often misses school or other activities because he/she doesn't feel well (current)	Symptom Checklist Parent report	SympChck-P
0,178867363	rh_VAmc	Volume of the right VAmc	Thalamic Nuclei	NA
0,178841603	left_hippocampal_fissure	Volume of the left hippocampal fissure	Hippocampus Subfields	NA
0,178287556	PreInt_EduHx_NeuroPsych	Has your child ever had any neuropsychological testing?	Interview-Education and Social History	Edu/Soc.Hist.
-0,178223963	SympChck_CSC_17P	Often has trouble going to sleep without parent/guardian nearby (past	Symptom Checklist Parent report	SympChck-P
0,178125873	leftPosterior	Volume of the left Posterior	Hypothalamic Subunits	NA
0,176615308	Left_Lateral_Ventricle	Volume of the Left Lateral Ventricle	Subcortical Volume	NA
0,176586058	right_hippocampal_fissure	Volume of the right hippocampal fissure	Hippocampus Subfields	NA
-0,176125873	APQ_SR_APQ_SR_07	7. You play games or do other fun things with your mom	Alabama Parenting Questionnaire	APQ
-0,175776854	lh_insula_volume	Cortical volume in lh insula	Cortical Volume	NA

Correlation coefficient (R)	Variable name in data	Item text	Instrument name	Instrument abbreviation (if applicable)
-0,173994991	APQ_SR_APQ_SR_05	5. Your parents reward or give something extra to you for behaving well	Alabama Parenting Questionnaire	APQ
0,173616073	Right_Pallidum	Volume of the Right Pallidum	Subcortical Volume	NA
-0,173259471	CTOPP_CTOPP_RSN_Sum	Rapid Symbolic Naming (RD+RL) sum score	Comprehensive Test of Phonological Processing	CTOPP-2
-0,172894186	CTOPP_CTOPP_RSN_Comp	Rapid Symbolic Naming (RD+RL) composite score	Comprehensive Test of Phonological Processing	CTOPP-2
0,172837912	Left_Hippocampus	Volume of the Left Hippocampus	Subcortical Volume	NA
0,171265419	rh_Pt	Volume of the right Pt	Thalamic Nuclei	NA
0,171057488	Right_Amygdala	Volume of the Right Amygdala	Subcortical Volume	NA
-0,170453615	rh_bankssts_area	Cortical area in rh bankssts	Cortical Area	NA
-0,16957395	SCARED_P_SCARED_P_08	8. My child follows me wherever I go	Screen for Child Anxiety Related Disorders	SCARED
0,169470989	Left_Pallidum	Volume of the Left Pallidum	Subcortical Volume	NA
0,167700268	x4th_Ventricle	Volume of the x4th Ventricle	Subcortical Volume	NA
0,167464303	left_Hippocampal_tail	Volume of the left Hippocampal tail	Hippocampus Subfields	NA
-0,167432342	rh_superiortemporal_area	Cortical area in rh superiortemporal	Cortical Area	NA
0,16700044	NLES_P_NLES_P_02a	2a. The child's close friend had serious troubles, problems, illness, or injury	Negative Life Events Scale	NLES
0,166143435	CBCL_CBCL_Int_T	Internalizing T Score	Child Behavior Checklist	CBCL
0,165059012	SDQ_SDQ_13	Often unhappy, depressed or tearful	Strength and Difficulties Questionnaire	SDQ
0,164969549	APQ_SR_APQ_SR_36	36. Your parents take away a privilege or money from you as punishment	Alabama Parenting Questionnaire	APQ
-0,164912621	CTOPP_CTOPP_RSN_P	Rapid Symbolic Naming (RD+RL) percentile score	Comprehensive Test of Phonological Processing	CTOPP-2
-0,164742099	SWAN_SWAN_18	18. Enters into conversation and	The Strengths and Weaknesses	SWAN

Correlation coefficient (R)	Variable name in data	Item text	Instrument name	Instrument abbreviation (if applicable)
		games without interrupting or intruding	Assessment of Normal Behavior	
0,1647406	ICU_P_ICU_P_Total	Total Score	Inventory of Callous-Unemotional Traits Parent	ICU
-0,164730671	SDQ_SDQ_Externalizing	Externalizing Score	Strength and Difficulties Questionnaire	SDQ
-0,164656645	rh_cuneus_area	Cortical area in rh cuneus	Cortical Area	NA
-0,164177062	SWAN_SWAN_15	15. Modulates verbal activity (controls excessive talking)	The Strengths and Weaknesses Assessment of Normal Behavior	SWAN
-0,164029326	rh_postcentral_area	Cortical area in rh postcentral	Cortical Area	NA
-0,163179291	rh_rostralanteriorcingulate_GI	Gyrification of rh rostralanteriorcingulate GI	Local Gyrification Index	NA
0,163176668	SympChck_CSC_52P	Has a hard time expressing feelings on his/her face during social interactions (past	Symptom Checklist Parent report	SympChck-P
0,163148091	SympChck_CSC_52C	Has a hard time expressing feelings on his/her face during social interactions (current)	Symptom Checklist Parent report	SympChck-P
0,162908792	SRS_SRS_33	33. Is socially awkward, even when he or she is trying to be polite.	Social Responsiveness Scale	SRS
0,162672775	CBCL_CBCL_SC	Somatic Complaints Raw Score	Child Behavior Checklist	CBCL
-0,162634498	APQ_SR_APQ_SR_37	37. Your parents send you to your room as punishment	Alabama Parenting Questionnaire	APQ
0,161323313	SRS_SRS_36	36. Has difficulty relating to adults.	Social Responsiveness Scale	SRS
0,1609441	PCIAT_PCIAT_16	16. How often does your child throw tantrums with your interference about how long he or she spends online?	Parent-Child Internet Addiction Test	PCIAT

Correlation coefficient (R)	Variable name in data	Item text	Instrument name	Instrument abbreviation (if applicable)
0,160922931	SCQ_SCQ_23	23. Does she/he ever use gestures, other than pointing or pulling your hand, to let you know what she/he wants?	Social Communication Questionnaire	SCQ
-0,160177506	rh_isthmuscingulate_area	Cortical area in rh isthmuscingulate	Cortical Area	NA
-0,160069216	Right_Caudate	Volume of the Right Caudate	Subcortical Volume	NA
0,160061424	PreInt_EduHx_detention	Detentions (past year)	Interview-Education and Social History	Edu/Soc.Hist.
-0,159273651	APQ_SR_APQ_SR_33	33. Your parents spank you with their hand when you have done something wrong	Alabama Parenting Questionnaire	APQ
-0,158251191	FGC_FGC_TL_Zone	Trunk lift fitness zone	FitnessGram Child	FGC
-0,158037148	rh_middletemporal_area	Cortical area in rh middletemporal	Cortical Area	NA
0,158028413	Right_Lateral_Ventricle	Volume of the Right Lateral Ventricle	Subcortical Volume	NA
0,157667453	ICU_P_ICU_P_24	24. Does things to make others feel good.	Inventory of Callous-Unemotional Traits Parent	ICU
0,157296804	right_Central_nucleus	Volume of the right Central nucleus	Amygdala Nuclei	NA
-0,156782384	lh_cuneus_area	Cortical area in lh cuneus	Cortical Area	NA
0,156524785	SRS_SRS_18	18. Has difficulty making friends, even when trying his or her best.	Social Responsiveness Scale	SRS
0,156301806	right_Paralaminar_nucleus	Volume of the right Paralaminar nucleus	Amygdala Nuclei	NA
-0,156213059	APQ_SR_APQ_SR_16	16. Your parents praise you for behaving well	Alabama Parenting Questionnaire	APQ
-0,156174141	SRS_SRS_52	52. Knows when he or she is talking too loud or making too much noise.	Social Responsiveness Scale	SRS
0,156031337	NLES_P_NLES_P_07a	7a. The child's parent suffered from serious illness, injury, or extreme pain,	Negative Life Events Scale	NLES

Correlation coefficient (R)	Variable name in data	Item text	Instrument name	Instrument abbreviation (if applicable)
		something that required rest for one week in bed, hospitalization, or surgery.		
0,155897551	right_Hippocampal_tail	Volume of the right Hippocampal tail	Hippocampus Subfields	NA
-0,155571068	WIAT_WIAT_Num_P	Numerical Operations Percentile Rank	Wechsler Individual Achievement Test	WIAT
0,155079322	left_CA3_body	Volume of the left CA3 body	Hippocampus Subfields	NA
-0,154953249	lh_lingual_area	Cortical area in lh lingual	Cortical Area	NA
-0,154006281	SCQ_SCQ_16	16. Does she/he ever have any complicated movements of her/his whole body, such as spinning or repeatedly bouncing up and down?	Social Communication Questionnaire	SCQ
0,153990616	SympChck_CSC_33C	Has frequent eating binges, and it feels out of control (current)	Symptom Checklist Parent report	SympChck-P
-0,153896192	PreInt_EduHx_strength_math	Math	Interview- Education and Social History	Edu/Soc.Hist.
0,153479198	right_CA3_body	Volume of the right CA3 body	Hippocampus Subfields	NA
0,152518477	SCQ_SCQ_32	32. If she/he wants something or wants help, does she/he look at you and use gestures with sounds or words to get your attention?	Social Communication Questionnaire	SCQ
0,152394009	right_molecular_layer_HP_head	Volume of the right molecular layer HP head	Hippocampus Subfields	NA
-0,152110864	APQ_P_APQ_P_PP	Positive Parenting Score	Alabama Parenting Questionnaire	APQ
0,151850783	SWAN_SWAN_09	9. Remembers daily activities	The Strengths and Weaknesses Assessment of Normal Behavior	SWAN
0,151733768	SCARED_P_SCARED_P_02	2. My child gets headaches when	Screen for Child Anxiety Related Disorders	SCARED

Correlation coefficient (R)	Variable name in data	Item text	Instrument name	Instrument abbreviation (if applicable)
		he/she is at school		
-0,151520683	FGC_FGC_CU_Zone	Curl up fitness zone	FitnessGram Child	FGC
0,151516743	SympChck_CSC_30C	Feels extremely worried about gaining weight or becoming fat (current)	Symptom Checklist Parent report	SympChck-P
0,150694917	APQ_SR_APQ_SR_14A	14A. How about your dad?	Alabama Parenting Questionnaire	APQ
-0,150165077	CBCL_CBCL_57	57. Physically attacks people	Child Behavior Checklist	CBCL
0,150067473	right_Whole_hippocampal_head	Volume of the right Whole hippocampal head	Hippocampus Subfields	NA
0,150054357	ICU_P_ICU_P_20	20. Does not like to put the time into doing things well.	Inventory of Callous-Unemotional Traits Parent	ICU
-0,149551833	APQ_P_APQ_P_18	18. You hug or kiss your child when he/she has done something well	Alabama Parenting Questionnaire	APQ
0,148543349	ASSQ_ASSQ_18	lacks common sense	Autism Spectrum Screening Questionnaire	ASSQ
-0,14769583	PreInt_Demos_Fam_P2_LegalGuardian	Parent 2 has legal guardianship over the child	Interview-Demographics/Family	Demog.Fam.
-0,147620739	CTOPP_CTOPP_EL_P	Elision percentile score	Comprehensive Test of Phonological Processing	CTOPP-2
-0,147573745	lh_posteriorcingulate_area	Cortical area in lh posteriorcingulate	Cortical Area	NA
-0,147280623	CBCL_CBCL_95	95. Temper tantrums or hot temper	Child Behavior Checklist	CBCL
0,146452012	APQ_SR_APQ_SR_22	22. Your parents let you out of a punishment early (like lift restrictions earlier than they originally said)	Alabama Parenting Questionnaire	APQ
0,146122171	left_Whole_hippocampal_head	Volume of the left Whole hippocampal head	Hippocampus Subfields	NA
0,145569161	rh_PuL	Volume of the right PuL	Thalamic Nuclei	NA

Correlation coefficient (R)	Variable name in data	Item text	Instrument name	Instrument abbreviation (if applicable)
0,145521737	SCARED_P_SCARED_P_SC	Social Anxiety Disorder Score	Screen for Child Anxiety Related Disorders	SCARED
-0,145175937	rh_precentral_volume	Cortical volume in rh precentral	Cortical Volume	NA
-0,145073946	lh_frontalpole_GI	Gyrification of lh frontalpole GI	Local Gyrification Index	NA
-0,145060772	SympChck_CSC_04C	Often feels overly happy and silly, above and beyond a normal feeling (current)	Symptom Checklist Parent report	SympChck-P
0,144094671	left_Paralaminar_nucleus	Volume of the left Paralaminar nucleus	Amygdala Nuclei	NA
-0,143942696	CTOPP_CTOPP_EL_S	Elision scaled score	Comprehensive Test of Phonological Processing	CTOPP-2
-0,14385682	APQ_SR_APQ_SR_CP	Corporal Punishment Score	Alabama Parenting Questionnaire	APQ
0,143376091	SDQ_SDQ_08	Many worries or often seems worried	Strength and Difficulties Questionnaire	SDQ
0,143375833	SympChck_CSC_10P	Has unusual thoughts or beliefs that others cannot understand or believe (past	Symptom Checklist Parent report	SympChck-P
0,143064538	right_CA1_head	Volume of the right CA1 head	Hippocampus Subfields	NA
-0,142989235	rh_rostralmiddlefrontal_area	Cortical area in rh rostralmiddlefrontal	Cortical Area	NA
0,142373433	ICU_P_ICU_P_01	1. Expresses his/her feelings openly.	Inventory of Callous-Unemotional Traits Parent	ICU
-0,142301726	CBCL_CBCL_21	21. Destroys things belonging to his/her family or others	Child Behavior Checklist	CBCL
0,141009765	right_Cortical_nucleus	Volume of the right Cortical nucleus	Amygdala Nuclei	NA
0,140893283	SDQ_SDQ_Emotional_Problems	Emotional Problems Scale	Strength and Difficulties Questionnaire	SDQ
0,140819682	PreInt_EduHx_weakness_history	Social studies/history	Interview-Education and Social History	Edu/Soc.Hist.
0,140692292	SCARED_P_SCARED_P_22	22. When my child gets frightened,	Screen for Child Anxiety Related Disorders	SCARED

Correlation coefficient (R)	Variable name in data	Item text	Instrument name	Instrument abbreviation (if applicable)
		he/she sweats a lot		
-0,140514966	rh_frontalpole_GI	Gyrification of rh frontalpole GI	Local Gyrification Index	NA
-0,140188052	Physical_HeartRate	Heart rate (beats/min)	Physical Measures	Physical
0,140028595	APQ_P_APQ_P_Total	APQ Total Score	Alabama Parenting Questionnaire	APQ
-0,139742639	lh_bankssts_area	Cortical area in lh bankssts	Cortical Area	NA
0,139402693	lh_Pc	Volume of the left Pc	Thalamic Nuclei	NA
0,139347321	PreInt_Demos_Fam_P1_RelQuality	How is the quality of the relationship between you and the child?	Interview-Demographics/Family	Demog.Fam.
0,139250754	left_molecular_layer_HP_head	Volume of the left molecular layer HP head	Hippocampus Subfields	NA
-0,139051587	SympChck_CSC_06P	Has periods of unusual energy and activity (past	Symptom Checklist Parent report	SympChck-P
-0,138734641	SympChck_CSC_37C	Often acts before thinking (current)	Symptom Checklist Parent report	SympChck-P
0,138436544	SRS_SRS_37	37. Has difficulty relating to peers.	Social Responsiveness Scale	SRS
0,137600815	left_CA1_head	Volume of the left CA1 head	Hippocampus Subfields	NA
0,13747502	SCQ_SCQ_24	24. Does she/he nod her/his head to indicate yes?	Social Communication Questionnaire	SCQ
0,13705804	SympChck_CSC_19P	Often feels very nervous around people (past	Symptom Checklist Parent report	SympChck-P
-0,13698735	PreInt_EduHx_sports	Playing sports	Interview-Education and Social History	Edu/Soc.Hist.
-0,136906642	APQ_SR_APQ_SR_02	2. Your parents tell you that you are doing a good job	Alabama Parenting Questionnaire	APQ
0,136904505	ASSQ_ASSQ_27	has markedly unusual posture	Autism Spectrum Screening Questionnaire	ASSQ
0,136562834	CC_Mid_Anterior	Volume of the CC Mid Anterior	Subcortical Volume	NA
0,136462078	SRS_SRS_02	2. Expressions on his or her face don't match what he or she is saying.	Social Responsiveness Scale	SRS

Correlation coefficient (R)	Variable name in data	Item text	Instrument name	Instrument abbreviation (if applicable)
-0,135831177	APQ_SR_APQ_SR_23	23. You help plan family activities	Alabama Parenting Questionnaire	APQ
0,135585856	ICU_P_ICU_P_12	12. Seems very cold and uncaring.	Inventory of Callous-Unemotional Traits Parent	ICU
0,135530204	SRS_SRS_11	11. Has good self-confidence.	Social Responsiveness Scale	SRS
0,135002436	SCARED_P_SCARED_P_GD	Generalized Anxiety Disorder Score	Screen for Child Anxiety Related Disorders	SCARED
-0,134907505	lh_rostralmiddlefrontal_area	Cortical area in lh rostralmiddlefrontal	Cortical Area	NA
-0,134720871	lh_supramarginal_area	Cortical area in lh supramarginal	Cortical Area	NA
-0,134394681	CTOPP_CTOPP_RD_S	Rapid Digit Naming scaled score	Comprehensive Test of Phonological Processing	CTOPP-2
0,133677448	Left_choroid_plexus	Volume of the Left choroid plexus	Subcortical Volume	NA
0,133527177	APQ_P_APQ_P_24	24. You get so busy that you forget where your child is and what he/she is doing	Alabama Parenting Questionnaire	APQ
-0,13334246	rh_frontalpole_area	Cortical area in rh frontalpole	Cortical Area	NA
-0,133298917	rh_lingual_area	Cortical area in rh lingual	Cortical Area	NA
-0,132399243	rh_posteriorcingulate_area	Cortical area in rh posteriorcingulate	Cortical Area	NA
0,132060231	SRS_SRS_SCI_T	Social Communication and Interaction T-Score	Social Responsiveness Scale	SRS
-0,1319355	WIAT_WIAT_Num_Stnd	Numerical Operations Standard Score	Wechsler Individual Achievement Test	WIAT
-0,131182606	WIAT_WIAT_LC_P	Listening Comprehension Percentile Rank	Wechsler Individual Achievement Test	WIAT
0,130686908	right_CA1_body	Volume of the right CA1 body	Hippocampus Subfields	NA
-0,129499304	WIAT_WIAT_LC_Stnd	Listening Comprehension Standard Score	Wechsler Individual Achievement Test	WIAT

Correlation coefficient (R)	Variable name in data	Item text	Instrument name	Instrument abbreviation (if applicable)
0,128761729	CBCL_CBCL_90	90. Swearing or obscene language	Child Behavior Checklist	CBCL
-0,128658695	SRS_SRS_09	9. Clings to adults, seems too dependent on them.	Social Responsiveness Scale	SRS
-0,12859965	CBCL_CBCL_47	47. Nightmares	Child Behavior Checklist	CBCL
0,128087769	SRS_SRS_SCI	Social Communication and Interaction Raw Score	Social Responsiveness Scale	SRS
-0,128070648	SWAN_SWAN_Total	SWAN Average	The Strengths and Weaknesses Assessment of Normal Behavior	SWAN
-0,127971214	CTOPP_CTOPP_RD_P	Rapid Digit Naming percentile score	Comprehensive Test of Phonological Processing	CTOPP-2
-0,127573597	APQ_P_APQ_P_16	16. You praise your child for behaving well	Alabama Parenting Questionnaire	APQ
-0,127504763	SWAN_SWAN_16	16. Reflects on questions (controls blurting out answers)	The Strengths and Weaknesses Assessment of Normal Behavior	SWAN
-0,12711821	ColorVision_CV_Plate_09_R	Plate 09 Result	Ishihara Color Vision Test	ColorVision
-0,127096466	lh_paracentral_area	Cortical area in lh paracentral	Cortical Area	NA
0,126944765	CSF	Volume of the CSF	Subcortical Volume	NA
0,126857052	right_presubiculum_head	Volume of the right presubiculum head	Hippocampus Subfields	NA
-0,126556827	CBCL_CBCL_22	22. Disobedient at home	Child Behavior Checklist	CBCL
-0,12611593	SympChck_CSC_38C	Often becomes really upset and loses his/her temper (current)	Symptom Checklist Parent report	SympChck-P
0,125479808	left_CA1_body	Volume of the left CA1 body	Hippocampus Subfields	NA
0,125378955	rh_PuA	Volume of the right PuA	Thalamic Nuclei	NA
-0,124999117	lh_superiortemporal_area	Cortical area in lh superiortemporal	Cortical Area	NA
0,124976329	lh_insula_area	Cortical area in lh insula	Cortical Area	NA
-0,124779886	SympChck_CSC_28C	Often has accidents when he/she wets the	Symptom Checklist Parent report	SympChck-P

Correlation coefficient (R)	Variable name in data	Item text	Instrument name	Instrument abbreviation (if applicable)
		bed or self during the day (current)		
-0,124490356	PreInt_EduHx_school_sports	Sports	Interview-Education and Social History	Edu/Soc.Hist.
-0,124397022	rh_paracentral_area	Cortical area in rh paracentral	Cortical Area	NA
-0,124198503	APQ_P_APQ_P_01	1. You have a friendly talk with your child	Alabama Parenting Questionnaire	APQ
0,124167535	SympChck_CSC_01C	Feels sad and down most days for at least 1 week (current)	Symptom Checklist Parent report	SympChck-P
-0,12368804	PreInt_Demos_Home_living_01	Child lives with Both biological parents	Interview-Demographic and Household Information	Demog.Household.
-0,12366334	SympChck_CSC_14C	Worries about being separated from parent/guardian because of getting lost or kidnapped (current)	Symptom Checklist Parent report	SympChck-P
0,123488112	rightAnterior_inferior	Volume of the right Anterior inferior	Hypothalamic Subunits	NA
0,123064932	CBCL_CBCL_112	112. Worries	Child Behavior Checklist	CBCL
0,123043933	NLES_P_NLES_P_09a	9a. The child's relatives such as aunts, uncles, grandparents said bad things about his/her mother or father.	Negative Life Events Scale	NLES
0,122236713	ICU_P_ICU_P_15	15. Always tries his/her best.	Inventory of Callous-Unemotional Traits Parent	ICU
0,121852945	SCQ_SCQ_25	25. Does she/he shake her/his head to indicate no?	Social Communication Questionnaire	SCQ
-0,121306656	rh_caudalanteriorcingulate_volume	Cortical volume in rh caudalanteriorcingulate	Cortical Volume	NA
-0,121219524	rh_caudalmiddlefrontal_area	Cortical area in rh caudalmiddlefrontal	Cortical Area	NA
0,120638942	right_subiculum_head	Volume of the right subiculum head	Hippocampus Subfields	NA

Correlation coefficient (R)	Variable name in data	Item text	Instrument name	Instrument abbreviation (if applicable)
0,12034325	rh_PuI	Volume of the right PuI	Thalamic Nuclei	NA
0,120253541	SRS_SRS_Total_T	Total T-Score	Social Responsiveness Scale	SRS
0,119484684	SympChck_CSC_25P	Feels very nervous and unable to relax most days of the week (past	Symptom Checklist Parent report	SympChck-P
-0,118927169	rh_parsopercularis_area	Cortical area in rh parsopercularis	Cortical Area	NA
0,11877526	SCARED_P_SCARED_P_03	3. My child doesn’t like to be with people he/she does't know well	Screen for Child Anxiety Related Disorders	SCARED
0,118525592	lh_VAmc	Volume of the left VAmc	Thalamic Nuclei	NA
0,118353445	APQ_SR_APQ_SR_42	42. Your parents give you extra chores as punishment	Alabama Parenting Questionnaire	APQ
-0,118247579	lh_parsopercularis_area	Cortical area in lh parsopercularis	Cortical Area	NA
0,118146002	NLES_P_NLES_P_08a	8a. The child's mother or father talked about having serious money troubles (being worried about bills for ordinary things).	Negative Life Events Scale	NLES
0,118045974	SympChck_CSC_23P	Worries most days of the week (past	Symptom Checklist Parent report	SympChck-P
0,118045427	CBCL_CBCL_04	4. Fails to finish things he/she starts	Child Behavior Checklist	CBCL
0,117949609	SRS_SRS_COG_T	Social Cognition T-Score	Social Responsiveness Scale	SRS
0,117443267	CBCL_CBCL_56A	56A. Aches or pains (not stomach or headaches)	Child Behavior Checklist	CBCL
-0,117279544	PreInt_EduHx_martial_arts	Martial Arts	Interview-Education and Social History	Edu/Soc.Hist.
0,11722562	CBCL_CBCL_13	13. Confused or seems to be in a fog	Child Behavior Checklist	CBCL
-0,11716388	lh_LD	Volume of the left LD	Thalamic Nuclei	NA

Correlation coefficient (R)	Variable name in data	Item text	Instrument name	Instrument abbreviation (if applicable)
0,117021967	left_presubiculum_head	Volume of the left presubiculum head	Hippocampus Subfields	NA
-0,117013238	CBCL_CBCL_14	14. Cries a lot	Child Behavior Checklist	CBCL
-0,116775143	rh_insula_volume	Cortical volume in rh insula	Cortical Volume	NA
0,116294757	SRS_SRS_COG	Social Cognition Raw Score	Social Responsiveness Scale	SRS
-0,116227868	rh_rostralanteriorcingulate_volume	Cortical volume in rh rostralanteriorcingulate	Cortical Volume	NA
0,115999138	SRS_SRS_Total	Total Raw Score	Social Responsiveness Scale	SRS
-0,115533696	CBCL_CBCL_11	11. Clings to adults or too dependent	Child Behavior Checklist	CBCL
-0,114954125	CBCL_CBCL_36	36. Gets hurt a lot, accident-prone	Child Behavior Checklist	CBCL
0,114772398	rh_LGN	Volume of the right LGN	Thalamic Nuclei	NA
-0,114721396	lh_rostralanteriorcingulate_volume	Cortical volume in lh rostralanteriorcingulate	Cortical Volume	NA
0,114626839	left_subiculum_head	Volume of the left subiculum head	Hippocampus Subfields	NA
-0,114524086	PreInt_EduHx_homework	Homework	Interview-Education and Social History	Edu/Soc.Hist.
0,114510861	SCARED_P_SCARED_P_09	9. People tell me that my child looks nervous	Screen for Child Anxiety Related Disorders	SCARED
-0,113653221	CBCL_CBCL_AB_T	Aggressive Behavior T Score	Child Behavior Checklist	CBCL
0,113459821	right_Anterior_amygdaloid_area_AAA	Volume of the right Anterior amygdaloid area AAA	Amygdala Nuclei	NA
0,112972108	left_Anterior_amygdaloid_area_AAA	Volume of the left Anterior amygdaloid area AAA	Amygdala Nuclei	NA
0,112311456	NLES_P_NLES_P_12a	12a. The child's mother or father was intoxicated in the child's presence	Negative Life Events Scale	NLES
0,111866784	lh_Whole_thalamus	Volume of the left Whole thalamus	Thalamic Nuclei	NA
-0,111460783	CBCL_CBCL_108	108. Wets the bed	Child Behavior Checklist	CBCL

Correlation coefficient (R)	Variable name in data	Item text	Instrument name	Instrument abbreviation (if applicable)
0,111339793	SDQ_SDQ_03	Often complains of headaches, stomach-aches or sickness	Strength and Difficulties Questionnaire	SDQ
0,111293351	CBCL_CBCL_61	61. Poor school work	Child Behavior Checklist	CBCL
0,111184956	SRS_SRS_03	3. Seems self-confident when interacting with others.	Social Responsiveness Scale	SRS
-0,111028051	Right_Accumbens_area	Volume of the Right Accumbens area	Subcortical Volume	NA
0,110988993	ASSQ_ASSQ_03	lives somewhat in a world of his/her own with restricted idiosyncratic intellectual interests	Autism Spectrum Screening Questionnaire	ASSQ
0,110927889	SCQ_SCQ_31	31. Does she/he ever try to comfort you if you are sad or hurt?	Social Communication Questionnaire	SCQ
0,110636924	SRS_SRS_10	10. Takes things too literally and doesn't get the real meaning of a conversation.	Social Responsiveness Scale	SRS
0,110382294	CBCL_CBCL_53	53. Overeating	Child Behavior Checklist	CBCL
-0,110018546	CBCL_CBCL_68	68. Screams a lot	Child Behavior Checklist	CBCL
0,109819318	left_GC_ML_DG_head	Volume of the left GC ML DG head	Hippocampus Subfields	NA
0,109620841	SCQ_SCQ_20	20. Does she/he ever talk with you just to be friendly (rather than to get something)?	Social Communication Questionnaire	SCQ
0,109470371	SympChck_CSC_20P	Often feels very nervous when he/she has to do things in front of others (past	Symptom Checklist Parent report	SympChck-P
0,108965961	ICU_P_ICU_P_11	11. Does not care about doing things well.	Inventory of Callous-Unemotional Traits Parent	ICU
0,108917112	left_CA4_head	Volume of the left CA4 head	Hippocampus Subfields	NA
0,108841721	SRS_SRS_59	59. Is overly suspicious.	Social Responsiveness Scale	SRS

Correlation coefficient (R)	Variable name in data	Item text	Instrument name	Instrument abbreviation (if applicable)
0,108560776	SCQ_SCQ_40	40. Does she/he play cooperatively in games that need some form of joining in with a group of other children, such as hide-and-seek or ball games?	Social Communication Questionnaire	SCQ
-0,108519815	ICU_P_ICU_P_03	3. Is concerned about schoolwork.	Inventory of Callous-Unemotional Traits Parent	ICU
-0,108250434	Left_Caudate	Volume of the Left Caudate	Subcortical Volume	NA
0,107408023	SCARED_P_SCARED_P_30	30. My child is afraid of having anxiety (or panic) attacks	Screen for Child Anxiety Related Disorders	SCARED
0,107371927	SympChck_CSC_10C	Has unusual thoughts or beliefs that others cannot understand or believe (current)	Symptom Checklist Parent report	SympChck-P
-0,106836389	APQ_SR_APQ_SR_OPD	APQ Total Score	Alabama Parenting Questionnaire	APQ
0,106777826	SRS_SRS_12	12. Is able to communicate his or her feelings to others.	Social Responsiveness Scale	SRS
-0,105973917	ARI_P_ARI_P_06	Loses temper easily	Affective Reactivity Index	ARI
-0,105949965	ARI_P_ARI_P_02	Often loses his/her temper	Affective Reactivity Index	ARI
0,105885823	EstimatedTotalIntraCranialVol	Estimated Intracranial Volume	eTIV	NA
-0,105773353	APQ_SR_APQ_SR_04A	4A. How about your dad?	Alabama Parenting Questionnaire	APQ
-0,105562438	SDQ_SDQ_05	Often loses temper	Strength and Difficulties Questionnaire	SDQ
-0,105194982	CBCL_CBCL_20	20. Destroys his/her own things	Child Behavior Checklist	CBCL
0,104768978	left_Central_nucleus	Volume of the left Central nucleus	Amygdala Nuclei	NA
0,104600198	ASSQ_ASSQ_15	wishes to be sociable but fails to make relationships with peers	Autism Spectrum Screening Questionnaire	ASSQ

Correlation coefficient (R)	Variable name in data	Item text	Instrument name	Instrument abbreviation (if applicable)
0,104519903	ICU_P_ICU_P_23	23. Works hard on everything.	Inventory of Callous-Unemotional Traits Parent	ICU
-0,104500269	CBCL_CBCL_41	41. Impulsive or acts without thinking	Child Behavior Checklist	CBCL
-0,104143978	lh_frontalpole_area	Cortical area in lh frontalpole	Cortical Area	NA
0,10373932	SRS_SRS_COM_T	Social Communication T-Score	Social Responsiveness Scale	SRS
-0,103737483	rh_superiorfrontal_area	Cortical area in rh superiorfrontal	Cortical Area	NA
-0,103613905	lh_pericalcarine_area	Cortical area in lh pericalcarine	Cortical Area	NA
-0,103239842	CBCL_CBCL_27	27. Easily jealous	Child Behavior Checklist	CBCL
0,103222848	right_presubiculum_body	Volume of the right presubiculum body	Hippocampus Subfields	NA
0,102278426	CBCL_CBCL_56C	56C. Nausea, feels sick	Child Behavior Checklist	CBCL
0,102065389	SCARED_P_SCARED_P_12	12. When my child gets frightened, he/she feels like he/she is going crazy	Screen for Child Anxiety Related Disorders	SCARED
-0,101455238	rh_parstriangularis_area	Cortical area in rh parstriangularis	Cortical Area	NA
-0,101201236	lh_caudalanteriorcingulate_volume	Cortical volume in lh caudalanteriorcingulate	Cortical Volume	NA
0,101192783	SympChck_CSC_26P	Is bothered by thoughts which keep coming into his/her head for no reason (past	Symptom Checklist Parent report	SympChck-P
-0,100701899	PreInt_EduHx_dance	Dance	Interview-Education and Social History	Edu/Soc.Hist.
0,100224218	CBCL_CBCL_52	52. Feels too guilty	Child Behavior Checklist	CBCL
0,099916458	SympChck_CSC_41P	Frequently lies (past	Symptom Checklist Parent report	SympChck-P
0,099828939	SCARED_P_SCARED_P_SH	Significant School Avoidance Score	Screen for Child Anxiety Related Disorders	SCARED
0,099289082	CBCL_CBCL_55	55. Overweight	Child Behavior Checklist	CBCL

Correlation coefficient (R)	Variable name in data	Item text	Instrument name	Instrument abbreviation (if applicable)
-0,099150126	SympChck_CSC_18P	Is afraid of being alone at home or in a different room than parent/guardian (past	Symptom Checklist Parent report	SympChck-P
-0,098831454	FGC_FGC_SRL	Sit & Reach total (left side)	FitnessGram Child	FGC
0,098715176	SympChck_CSC_19C	Often feels very nervous around people (current)	Symptom Checklist Parent report	SympChck-P
0,098293828	rh_VA	Volume of the right VA	Thalamic Nuclei	NA
-0,098007461	lh_middletemporal_area	Cortical area in lh middletemporal	Cortical Area	NA
-0,097952025	SRS_SRS_47	47. Is too silly or laughs inappropriately.	Social Responsiveness Scale	SRS
-0,097698991	CBCL_CBCL_AB	Aggressive Behavior Raw Score	Child Behavior Checklist	CBCL
-0,097356723	WIAT_WIAT_MP_P	Math Problem Solving Percentile Rank	Wechsler Individual Achievement Test	WIAT
-0,097351761	SCARED_P_SCARED_P_04	4. My child gets scared if he/she sleeps away from home	Screen for Child Anxiety Related Disorders	SCARED
0,097044434	SCARED_P_SCARED_P_19	19. He/she child gets shaky	Screen for Child Anxiety Related Disorders	SCARED
0,096209248	right_GC_ML_DG_head	Volume of the right GC ML DG head	Hippocampus Subfields	NA
-0,096027699	SympChck_CSC_04P	Often feels overly happy and silly, above and beyond a normal feeling (past	Symptom Checklist Parent report	SympChck-P
0,095793785	SRS_SRS_29	29. Is regarded by other children as odd or weird.	Social Responsiveness Scale	SRS
0,095053344	ASSQ_ASSQ_11	uses language freely but fails to make adjustments to fit social contexts or the needs of different listeners	Autism Spectrum Screening Questionnaire	ASSQ
-0,094608412	SCARED_P_SCARED_P_20	20. My child has nightmares about something bad happening to him/her	Screen for Child Anxiety Related Disorders	SCARED

Correlation coefficient (R)	Variable name in data	Item text	Instrument name	Instrument abbreviation (if applicable)
0,0943534	PreInt_EduHx_repeated_grades	Were any grades repeated?	Interview- Education and Social History	Edu/Soc.Hist.
0,094198552	SCARED_P_SCARED_P_05	5. My child worries about other people liking him/her	Screen for Child Anxiety Related Disorders	SCARED
0,093652449	PreInt_FamHx_RDC_ffdk	Don't know father's father's current age	Family History/Research Diagnostic Criteria	Fam.Med.Hist
-0,09339161	CBCL_CBCL_23	23. Disobedient at school	Child Behavior Checklist	CBCL
-0,093136484	SympChck_CSC_05C	Has strong and explosive feelings of anger (current)	Symptom Checklist Parent report	SympChck-P
0,093063454	rh_insula_area	Cortical area in rh insula	Cortical Area	NA
0,093049566	ASSQ_ASSQ_10	is surprisingly good at some things and surprisingly poor at others	Autism Spectrum Screening Questionnaire	ASSQ
-0,09293423	lh_fusiform_area	Cortical area in lh fusiform	Cortical Area	NA
0,092727481	SCARED_P_SCARED_P_28	28. People tell me that my child worries too much	Screen for Child Anxiety Related Disorders	SCARED
-0,092643675	lh_parstriangularis_area	Cortical area in lh parstriangularis	Cortical Area	NA
-0,092488514	APQ_SR_APQ_SR_13	13. Your parents compliment you when you have done something well	Alabama Parenting Questionnaire	APQ
-0,09243251	SDQ_SDQ_Conduct_Problems	Conduct problems scale	Strength and Difficulties Questionnaire	SDQ
0,092282034	SDQ_SDQ_14	Generally liked by other children (for 11-17 year olds: Generally liked by other youth)	Strength and Difficulties Questionnaire	SDQ
-0,091769253	CBCL_CBCL_SP	Social Problems Raw Score	Child Behavior Checklist	CBCL
0,091697694	ASSQ_ASSQ_26	has markedly unusual facial expression	Autism Spectrum Screening Questionnaire	ASSQ
-0,09167597	APQ_P_APQ_P_15	15. You drive your child to a special activity	Alabama Parenting Questionnaire	APQ
-0,091637131	lh_temporalpole_volume	Cortical volume in lh temporalpole	Cortical Volume	NA

Correlation coefficient (R)	Variable name in data	Item text	Instrument name	Instrument abbreviation (if applicable)
-0,091544123	lh_caudalmiddlefrontal_area	Cortical area in lh caudalmiddlefrontal	Cortical Area	NA
0,091478467	SRS_SRS_21	21. Is able to imitate others' actions.	Social Responsiveness Scale	SRS
0,090796673	SWAN_SWAN_06	6. Engages in tasks that require sustained mental effort	The Strengths and Weaknesses Assessment of Normal Behavior	SWAN
-0,090707387	SympChck_CSC_37P	Often acts before thinking (past	Symptom Checklist Parent report	SympChck-P
0,090438676	lh_parahippocampal_area	Cortical area in lh parahippocampal	Cortical Area	NA
0,090430072	SRS_SRS_COM	Social Communication Raw Score	Social Responsiveness Scale	SRS
-0,090185502	lh_MDl	Volume of the left MDl	Thalamic Nuclei	NA
0,08992112	SRS_SRS_35	35. Has trouble keeping up with the flow of a normal conversation.	Social Responsiveness Scale	SRS
0,089915798	NLES_P_NLES_P_15a	15a. The child's mother or father lost a job	Negative Life Events Scale	NLES
-0,089304518	SDQ_SDQ_21	Thinks things out before acting	Strength and Difficulties Questionnaire	SDQ
-0,089045727	SCQ_SCQ_05	5. Does she/he ever get her/his pronouns mixed up (e.g., saying you or she/he for I)?	Social Communication Questionnaire	SCQ
-0,088486126	lh_precentral_area	Cortical area in lh precentral	Cortical Area	NA
0,088395481	left_CA3_head	Volume of the left CA3 head	Hippocampus Subfields	NA
-0,088192354	SympChck_CSC_38P	Often becomes really upset and loses his/her temper (past	Symptom Checklist Parent report	SympChck-P
0,087976925	ICU_P_ICU_P_10	10. Does not let feelings control him/her.	Inventory of Callous-Unemotional Traits Parent	ICU
0,087974684	right_CA4_head	Volume of the right CA4 head	Hippocampus Subfields	NA
0,087856992	rh_MDm	Volume of the right MDm	Thalamic Nuclei	NA
0,087261636	SCQ_SCQ_02	2. Do you have a to and fro "conversation"	Social Communication Questionnaire	SCQ

Correlation coefficient (R)	Variable name in data	Item text	Instrument name	Instrument abbreviation (if applicable)
		with her/him that involves taking turns or building on what you have said?		
0,087017143	SRS_SRS_49	49. Does extremely well at a few tasks, but does not do as well at most other tasks.	Social Responsiveness Scale	SRS
-0,086852321	rh_supramarginal_area	Cortical area in rh supramarginal	Cortical Area	NA
0,085202906	left_presubiculum_body	Volume of the left presubiculum body	Hippocampus Subfields	NA
-0,085087048	CBCL_CBCL_28	28. Breaks rules at home, school, or elsewhere	Child Behavior Checklist	CBCL
0,084884623	SCARED_P_SCARED_P_01	1. When my child feels frightened, it is hard to breathe	Screen for Child Anxiety Related Disorders	SCARED
0,084808657	PreInt_FamHx_RDC_ffmfdk	Don't know father's paternal grandmother's current age	Family History/Research Diagnostic Criteria	Fam.Med.Hist
-0,084777577	APQ_P_APQ_P_33	33. You spank your child with your hand when he/she has done something wrong	Alabama Parenting Questionnaire	APQ
-0,084153414	APQ_P_APQ_P_CP	Corporal Punishment Score	Alabama Parenting Questionnaire	APQ
0,083723826	SWAN_SWAN_05	5. Organizes tasks and activities	The Strengths and Weaknesses Assessment of Normal Behavior	SWAN
0,083539791	SRS_SRS_62	62. Give unusual or illogical reasons for doing things.	Social Responsiveness Scale	SRS
0,083403879	SympChck_CSC_23C	Worries most days of the week (current)	Symptom Checklist Parent report	SympChck-P
-0,083104268	rh_parsorbitalis_area	Cortical area in rh parsorbitalis	Cortical Area	NA
-0,082952679	WIAT_WIAT_MP_Stnd	Math Problem Solving Standard Score	Wechsler Individual Achievement Test	WIAT
-0,082819289	SympChck_CSC_05P	Has strong and explosive feelings of anger (past	Symptom Checklist Parent report	SympChck-P
-0,082652419	CBCL_CBCL_Ext_T	Externalizing T Score	Child Behavior Checklist	CBCL

Correlation coefficient (R)	Variable name in data	Item text	Instrument name	Instrument abbreviation (if applicable)
-0,082580426	PreInt_EduHx_bestfriend	Does your child have a best friend?	Interview-Education and Social History	Edu/Soc.Hist.
0,082379653	ASSQ_ASSQ_05	has a literal understanding of ambiguous and metaphoric language (i.e. takes things literally; troubles understanding expressions or metaphors)	Autism Spectrum Screening Questionnaire	ASSQ
0,082352738	SCQ_SCQ_33	33. Does she/he show a normal range of facial expressions?	Social Communication Questionnaire	SCQ
-0,082324812	FGC_FGC_SRR	Sit & Reach total (right side)	FitnessGram Child	FGC
-0,082015201	SDQ_SDQ_07	Generally well behaved, usually does what adults request	Strength and Difficulties Questionnaire	SDQ
-0,081850285	PreInt_EduHx_weakness_other	Other	Interview-Education and Social History	Edu/Soc.Hist.
0,081769234	ARI_P_ARI_P_04	Is angry most of the time	Affective Reactivity Index	ARI
0,081662445	CBCL_CBCL_100	100. Trouble sleeping	Child Behavior Checklist	CBCL
-0,08152803	CBCL_CBCL_OP	Other Problems Raw Score	Child Behavior Checklist	CBCL
0,081444317	PreInt_FamHx_RDC_fffdk	Don't know father's paternal grandfather's current age	Family History/Research Diagnostic Criteria	Fam.Med.Hist
0,081133242	left_Cortical_nucleus	Volume of the left Cortical nucleus	Amygdala Nuclei	NA
-0,080862801	APQ_SR_APQ_SR_15A	15A. How about your dad?	Alabama Parenting Questionnaire	APQ
-0,080828667	DTS_DTS_11	11. I am ashamed of myself when I feel distressed or upset	Distress Tolerance Scale	DTS
0,080359172	SRS_SRS_48	48. Has a sense of humor, understands jokes.	Social Responsiveness Scale	SRS
-0,080208567	rh_fusiform_area	Cortical area in rh fusiform	Cortical Area	NA
0,079989099	SympChck_CSC_45P	Has stolen or shoplifted items (past	Symptom Checklist Parent report	SympChck-P

Correlation coefficient (R)	Variable name in data	Item text	Instrument name	Instrument abbreviation (if applicable)
0,079974574	rh_PuM	Volume of the right PuM	Thalamic Nuclei	NA
-0,079766582	APQ_P_APQ_P_05	5. You reward or give something extra to your child for obeying you or behaving well	Alabama Parenting Questionnaire	APQ
-0,079377923	lh_precentral_thickness	Cortical thickness in lh precentral	Cortical Thickness	NA
0,079331187	ICU_P_ICU_P_07	7. Does not care about being on time.	Inventory of Callous-Unemotional Traits Parent	ICU
0,079268065	CBCL_CBCL_12	12. Complains of loneliness	Child Behavior Checklist	CBCL
-0,079036376	Barratt_Barratt_Total_Edu	Education total score	Barratt Simplified Measure of Social Status	BSMSS
0,078832356	left_fimbria	Volume of the left fimbria	Hippocampus Subfields	NA
-0,078539941	lh_parahippocampal_volume	Cortical volume in lh parahippocampal	Cortical Volume	NA
0,078451535	ARI_P_ARI_P_01	Is easily annoyed by others	Affective Reactivity Index	ARI
-0,078283963	ARI_S_ARI_S_Total_Score	Total Score	Affective Reactivity Index	ARI
-0,077911439	rh_parahippocampal_volume	Cortical volume in rh parahippocampal	Cortical Volume	NA
0,077892728	SympChck_CSC_09P	Hears, sees, or smells things that other people cannot (current)	Symptom Checklist Parent report	SympChck-P
0,077859235	CBCL_CBCL_TP_T	Thought Problems T Score	Child Behavior Checklist	CBCL
0,07719501	SympChck_CSC_11P	Has anxiety attacks, where out of the blue he/she suddenly feels scared (past	Symptom Checklist Parent report	SympChck-P
0,076838655	lh_CeM	Volume of the left CeM	Thalamic Nuclei	NA
0,07683528	SCQ_SCQ_09	9. Does her/his facial expression usually seem appropriate to the particular situation, as far as you can tell?	Social Communication Questionnaire	SCQ
0,076716706	SCARED_P_SCARED_P_35	35. My child worries about how well he/she does things	Screen for Child Anxiety Related Disorders	SCARED

Correlation coefficient (R)	Variable name in data	Item text	Instrument name	Instrument abbreviation (if applicable)
0,07669768	SRS_SRS_25	25. Doesn't seem to mind being out of step with or "not on the same wavelength" as others.	Social Responsiveness Scale	SRS
0,076656292	Pegboard_peg_drops_d	Dominant Hand - Number of drops	Grooved Pegboard	Pegboard
-0,076097083	ARI_S_ARI_S_02	I often lose my temper	Affective Reactivity Index	ARI
0,075891	SDQ_SDQ_04	Shares readily with other children, for example toys, treats, pencils (for 11-17 year olds: Shares readily with other youth, for example CD's, games, food)	Strength and Difficulties Questionnaire	SDQ
0,07558989	CBCL_CBCL_85	85. Strange ideas	Child Behavior Checklist	CBCL
0,074971375	SympChck_CSC_51P	Often has a difficult time making eye contact (past	Symptom Checklist Parent report	SympChck-P
0,074911975	APQ_SR_APQ_SR_20	20. Your mom talks to you about your friends	Alabama Parenting Questionnaire	APQ
-0,074834645	APQ_SR_APQ_SR_35	35. Your parents slap you when you have done something wrong	Alabama Parenting Questionnaire	APQ
0,074420957	SCQ_SCQ_19	19. Does she/he have any particular friends or a best friend?	Social Communication Questionnaire	SCQ
0,07422371	SRS_SRS_54	54. Seems to react to people as if they are objects.	Social Responsiveness Scale	SRS
0,074058465	PreInt_Demos_Home_living_03	Child lives with Biological mother	Interview-Demographic and Household Information	Demog.Household.
0,073425179	CBCL_CBCL_50	50. Too fearful or anxious	Child Behavior Checklist	CBCL
-0,073177467	lh_parsorbitalis_area	Cortical area in lh parsorbitalis	Cortical Area	NA
-0,072980225	CBCL_CBCL_07	7. Bragging, boasting	Child Behavior Checklist	CBCL
0,072962287	rh_parahippocampal_area	Cortical area in rh parahippocampal	Cortical Area	NA

Correlation coefficient (R)	Variable name in data	Item text	Instrument name	Instrument abbreviation (if applicable)
0,072761446	APQ_P_APQ_P_42	42. You give your child extra chores as punishment	Alabama Parenting Questionnaire	APQ
0,072198602	PreInt_EduHx_getalongkids_school	How does your child get along with other children at school?	Interview-Education and Social History	Edu/Soc.Hist.
-0,071997983	SWAN_SWAN_03	3. Listens when spoken to directly	The Strengths and Weaknesses Assessment of Normal Behavior	SWAN
0,071744095	ASSQ_ASSQ_02	is regarded as an 'eccentric professor' by the other children	Autism Spectrum Screening Questionnaire	ASSQ
0,071711114	right_CA3_head	Volume of the right CA3 head	Hippocampus Subfields	NA
-0,07161067	APQ_SR_APQ_SR_INV_D	Mother Involvement Score	Alabama Parenting Questionnaire	APQ
-0,071405485	lh_superiorfrontal_area	Cortical area in lh superiorfrontal	Cortical Area	NA
0,071389097	CBCL_CBCL_35	35. Feels worthless or inferior	Child Behavior Checklist	CBCL
-0,070969921	Optic_Chiasm	Volume of the Optic Chiasm	Subcortical Volume	NA
0,07090004	SympChck_CSC_02P	Often feels annoyed and irritated (past	Symptom Checklist Parent report	SympChck-P
0,070587622	lh_Pt	Volume of the left Pt	Thalamic Nuclei	NA
0,070511251	CBCL_CBCL_89	89. Suspicious	Child Behavior Checklist	CBCL
-0,070242651	rh_LD	Volume of the right LD	Thalamic Nuclei	NA
0,070168166	SCQ_SCQ_37	37. Does she/he respond positively when another child approaches her/him?	Social Communication Questionnaire	SCQ
0,070158785	SympChck_CSC_20C	Often feels very nervous when he/she has to do things in front of others (current)	Symptom Checklist Parent report	SympChck-P
0,070072347	SCARED_P_SCARED_P_11	11. My child gets stomachaches at school	Screen for Child Anxiety Related Disorders	SCARED
0,069480732	SCARED_P_SCARED_P_32	32. My child feels shy with people he/she doesn't know well	Screen for Child Anxiety Related Disorders	SCARED

Correlation coefficient (R)	Variable name in data	Item text	Instrument name	Instrument abbreviation (if applicable)
0,069461052	SympChck_CSC_26C	Is bothered by thoughts which keep coming into his/her head for no reason (current)	Symptom Checklist Parent report	SympChck-P
0,069441053	lh_VA	Volume of the left VA	Thalamic Nuclei	NA
0,069307857	SympChck_CSC_34P	Has trouble paying attention, and it affects school work or performance (past	Symptom Checklist Parent report	SympChck-P
-0,06921817	SRS_SRS_41	41. Wanders aimlessly from one activity to another.	Social Responsiveness Scale	SRS
0,069166618	PreInt_EduHx_suspension	Suspensions (past year)	Interview-Education and Social History	Edu/Soc.Hist.
-0,068788094	APQ_SR_APQ_SR_27	27. Your parents tell you that they like it when you help out around the house	Alabama Parenting Questionnaire	APQ
0,068752315	SRS_SRS_DSMRRB_T	Restricted Interests and Repetitive Behavior T-Score	Social Responsiveness Scale	SRS
-0,068742094	rh_precentral_area	Cortical area in rh precentral	Cortical Area	NA
0,068709255	SRS_SRS_RRB_T	Restricted Interests and Repetitive Behavior T-Score	Social Responsiveness Scale	SRS
0,068685377	SCARED_P_SCARED_P_Total	Total Score	Screen for Child Anxiety Related Disorders	SCARED
-0,068215262	rh_MV_Re_	Volume of the right MV Re	Thalamic Nuclei	NA
0,067952095	CBCL_CBCL_AD	Anxious/Depressed Raw Score	Child Behavior Checklist	CBCL
-0,067431074	SCQ_SCQ_10	10. Does she/he ever use your hand like a tool or as if it were part of her/his own body (e.g., pointing with your finder or putting your hand on a doorknob to get you to open the door?)	Social Communication Questionnaire	SCQ

Correlation coefficient (R)	Variable name in data	Item text	Instrument name	Instrument abbreviation (if applicable)
-0,067335316	PreInt_EduHx_CPSE	Does your child have CPSE services?	Interview-Education and Social History	Edu/Soc.Hist.
0,067294755	SRS_SRS_32	32. Has good personal hygiene.	Social Responsiveness Scale	SRS
0,067153463	SympChck_CSC_25C	Feels very nervous and unable to relax most days of the week (current)	Symptom Checklist Parent report	SympChck-P
-0,06702773	APQ_P_APQ_P_02	2. You let your child know when he/she is doing a good job with something	Alabama Parenting Questionnaire	APQ
0,066873789	CTOPP_CTOPP_BW_S	Blending Words scaled score	Comprehensive Test of Phonological Processing	CTOPP-2
0,066531149	CTOPP_CTOPP_BW_P	Blending Words percentile score	Comprehensive Test of Phonological Processing	CTOPP-2
0,066350214	SympChck_CSC_07P	Has times when he/she sleeps much less than usual but still feels rested (past	Symptom Checklist Parent report	SympChck-P
-0,066255268	lh_transversetemporal_area	Cortical area in lh transversetemporal	Cortical Area	NA
-0,06605895	CBCL_CBCL_97	97. Threatens people	Child Behavior Checklist	CBCL
-0,065096889	PreInt_DevHx_preg_symp_07	Took any prescription	Interview-Developmental History	Dev.Hist.
0,064796872	SRS_SRS_DSMRRB	Restricted Interests and Repetitive Behavior Raw Score	Social Responsiveness Scale	SRS
0,064796872	SRS_SRS_RRB	Restricted Interests and Repetitive Behavior Raw Score	Social Responsiveness Scale	SRS
-0,06477551	SDQ_SDQ_20	Often offers to help others (parents, teachers, children)	Strength and Difficulties Questionnaire	SDQ
-0,0644894	ARI_S_ARI_S_06	I lose my temper easily	Affective Reactivity Index	ARI

Correlation coefficient (R)	Variable name in data	Item text	Instrument name	Instrument abbreviation (if applicable)
-0,064148148	SympChck_CSC_43P	Frequently starts physical fights with peers (past	Symptom Checklist Parent report	SympChck-P
0,063062249	NLES_P_NLES_P_19a	19a. The child's father acted very worried, upset, or sad, not because of something the child did.	Negative Life Events Scale	NLES
-0,062856629	rh_pericalcarine_area	Cortical area in rh pericalcarine	Cortical Area	NA
-0,062581242	APQ_SR_APQ_SR_INV_M	Father Involvement Score	Alabama Parenting Questionnaire	APQ
0,062328466	SympChck_CSC_07C	Has times when he/she sleeps much less than usual but still feels rested (current)	Symptom Checklist Parent report	SympChck-P
0,062155115	NLES_P_NLES_P_10a	10a. The child's mother or father fought or argued with his/her relatives such as aunts, uncles, grandparents	Negative Life Events Scale	NLES
0,061992719	CBCL_CBCL_56F	56F. Stomachaches	Child Behavior Checklist	CBCL
0,061804806	SRS_SRS_58	58. Concentrates too much on parts of things rather than seeing the whole picture. For example, if asked to describe what happened in a story, he or she may talk only about the kind of clothes the characters were wearing.	Social Responsiveness Scale	SRS
0,061614683	SympChck_CSC_51C	Often has a difficult time making eye contact (current)	Symptom Checklist Parent report	SympChck-P
0,061528832	CBCL_CBCL_76	76. Sleeps less than most kids	Child Behavior Checklist	CBCL
0,061505895	CBCL_CBCL_65	65. Refuses to talk	Child Behavior Checklist	CBCL
0,061259201	ASSQ_ASSQ_13	makes naïve and embarrassing remarks	Autism Spectrum Screening Questionnaire	ASSQ
-0,061079914	CBCL_CBCL_Ext	Externalizing Raw Score	Child Behavior Checklist	CBCL

Correlation coefficient (R)	Variable name in data	Item text	Instrument name	Instrument abbreviation (if applicable)
-0,061030083	SCQ_SCQ_12	12. Does she/he ever seem to be more interested in parts of a toy or an object (e.g., spinning the wheels of a car), rather than in using the object as it was intended?	Social Communication Questionnaire	SCQ
0,06102958	SDQ_SDQ_11	Has at least one good friend	Strength and Difficulties Questionnaire	SDQ
0,060755604	CBCL_CBCL_AD_T	Anxious/Depressed T Score	Child Behavior Checklist	CBCL
0,060667305	SRS_SRS_07	7. Is aware of what others are thinking or feeling.	Social Responsiveness Scale	SRS
0,060594116	PreInt_EduHx_strength_science	Science	Interview- Education and Social History	Edu/Soc.Hist.
0,060411733	SWAN_SWAN_IN	Inattention Average	The Strengths and Weaknesses Assessment of Normal Behavior	SWAN
-0,060373337	APQ_SR_APQ_SR_15	15. Your mom drives you to a special activity	Alabama Parenting Questionnaire	APQ
0,060118246	rh_CL	Volume of the right CL	Thalamic Nuclei	NA
-0,059872025	Barratt_financialsupport	Who is providing financial support for the child?	Barratt Simplified Measure of Social Status	BSMSS
0,059816005	CC_Anterior	Volume of the CC Anterior	Subcortical Volume	NA
0,059785867	rh_CeM	Volume of the right CeM	Thalamic Nuclei	NA
-0,059678731	Barratt_Barratt_Total	Barratt Total Score	Barratt Simplified Measure of Social Status	BSMSS
0,059480489	CBCL_CBCL_17	17. Daydreams or gets lost in his/her thoughts	Child Behavior Checklist	CBCL
0,059199322	ASSQ_ASSQ_06	has a deviant style of communication with a formal, fussy, ‘old-fashioned’ or ‘robotlike’ language (i.e. talks differently than other children, in a	Autism Spectrum Screening Questionnaire	ASSQ

Correlation coefficient (R)	Variable name in data	Item text	Instrument name	Instrument abbreviation (if applicable)
		formal or stilted way)		
-0,059022242	DTS_DTS_06	6. I can tolerate being distressed or upset as well as most people	Distress Tolerance Scale	DTS
-0,058867242	Barratt_Barratt_P1_Edu	Parent 1 level of education	Barratt Simplified Measure of Social Status	BSMSS
0,058287342	PreInt_Demos_Fam_guardian_maritalstatus	Parent(s)/Guardian(s) Marital Status:	Interview-Demographics/Family	Demog.Fam.
-0,058158445	lh_MV_Re_	Volume of the left MV Re	Thalamic Nuclei	NA
-0,05803469	CBCL_CBCL_29	29. Fears certain animals, situations, or places, other than school	Child Behavior Checklist	CBCL
0,057400625	SWAN_SWAN_01	1. Gives close attention to detail and avoids careless mistakes	The Strengths and Weaknesses Assessment of Normal Behavior	SWAN
-0,057029379	lh_temporalpole_thickness	Cortical thickness in lh temporalpole	Cortical Thickness	NA
-0,056902024	SCQ_SCQ_18	18. Does she/he ever have any objects (other than a soft toy or comfort blanket) that she/he has to carry around?	Social Communication Questionnaire	SCQ
0,056896114	lh_rostralanteriorcingulate_area	Cortical area in lh rostralanteriorcingulate	Cortical Area	NA
0,056743895	SRS_SRS_65	65. Stares or gazes off into space.	Social Responsiveness Scale	SRS
0,056539984	APQ_P_APQ_P_20	20. You talks to your child about his/her friends	Alabama Parenting Questionnaire	APQ
0,05651974	CBCL_CBCL_09	9. Can't get his/her mind off certain thoughts; obsessions	Child Behavior Checklist	CBCL
0,055956581	CBCL_CBCL_49	49. Constipated, doesn't move bowels	Child Behavior Checklist	CBCL
-0,055727978	lh_entorhinal_thickness	Cortical thickness in lh entorhinal	Cortical Thickness	NA
-0,055602357	SympChck_CSC_22C	Has intense fears of specific animals, situations, or	Symptom Checklist Parent report	SympChck-P

Correlation coefficient (R)	Variable name in data	Item text	Instrument name	Instrument abbreviation (if applicable)
		anything else (current)		
-0,055310227	APQ_P_APQ_P_34	34. You ignore your child when he/she is misbehvaing	Alabama Parenting Questionnaire	APQ
-0,054999476	SympChck_CSC_40C	Actively disobeys or doesn't listen to adult rules (current)	Symptom Checklist Parent report	SympChck-P
-0,054902611	CBCL_CBCL_16	16. Cruelty, bullying, or meanness to others	Child Behavior Checklist	CBCL
-0,054721808	SDQ_SDQ_15	Easily distracted, concentration wanders	Strength and Difficulties Questionnaire	SDQ
-0,054626593	APQ_SR_APQ_SR_04	4. Your mom helps you with some of your special acivities (such as sports, boy/girl scouts, church youth groups)	Alabama Parenting Questionnaire	APQ
0,054525648	ICU_P_ICU_P_Uncaring	Uncaring Subscale Score	Inventory of Callous-Unemotional Traits Parent	ICU
0,054269403	CBCL_CBCL_46	46. Nervous movements or twitching	Child Behavior Checklist	CBCL
0,054123831	SympChck_CSC_02C	Often feels annoyed and irritated (current)	Symptom Checklist Parent report	SympChck-P
0,053926196	SDQ_SDQ_17	Kind to younger children	Strength and Difficulties Questionnaire	SDQ
0,053718427	CBCL_CBCL_75	75. Too shy or timid	Child Behavior Checklist	CBCL
0,053535979	NLES_P_NLES_P_13a	13a. The child's mother or father forgot to do important things for him/her that they promised they would do, such as take him/her on a trip, take him/her to nice places, or come to his/her school or athletic activities.	Negative Life Events Scale	NLES

Correlation coefficient (R)	Variable name in data	Item text	Instrument name	Instrument abbreviation (if applicable)
-0,05346708	APQ_P_APQ_P_23	23. Your child helps plan family activities	Alabama Parenting Questionnaire	APQ
0,052965605	ASSQ_ASSQ_25	is bullied by other children	Autism Spectrum Screening Questionnaire	ASSQ
-0,052819918	APQ_SR_APQ_SR_34	34. Your parents ignore you when you are misbehvaing	Alabama Parenting Questionnaire	APQ
-0,052775369	CBCL_CBCL_63	63. Prefers being with older kids	Child Behavior Checklist	CBCL
0,052619896	SRS_SRS_44	44. Doesn't understand how events relate to one another (cause and effect) the way other children his or her age do.	Social Responsiveness Scale	SRS
-0,052551463	APQ_SR_APQ_SR_26	26. Your mom goes to a meeting at school, like a PTA meeting, or a parent/teacher conference	Alabama Parenting Questionnaire	APQ
-0,052354637	CBCL_CBCL_37	37. Gets in many fights	Child Behavior Checklist	CBCL
-0,052246838	SCARED_P_SCARED_P_34	34. When my child gets frightened, he/she feels like throwing up	Screen for Child Anxiety Related Disorders	SCARED
0,051722377	lh_entorhinal_area	Cortical area in lh entorhinal	Cortical Area	NA
-0,051713394	SympChck_CSC_29P	Often has accidents when he/she soild the bed or self during the day (past	Symptom Checklist Parent report	SympChck-P
-0,051574976	rh_entorhinal_thickness	Cortical thickness in rh entorhinal	Cortical Thickness	NA
0,051336551	Pegboard_peg_drops_nd	Non-dominant Hand - Number of drops	Grooved Pegboard	Pegboard
0,051328997	ASSQ_ASSQ_20	has clumsy, ill coordinated, ungainly, awkward movements or gestures	Autism Spectrum Screening Questionnaire	ASSQ
-0,051172564	ICU_P_ICU_P_04	4. Does not care who he/she hurts to get what he/she wants.	Inventory of Callous-Unemotional Traits Parent	ICU

Correlation coefficient (R)	Variable name in data	Item text	Instrument name	Instrument abbreviation (if applicable)
-0,05113583	APQ_SR_APQ_SR_12	12. Your parents give up trying to get you to obey them because it's too much trouble	Alabama Parenting Questionnaire	APQ
0,050939644	SRS_SRS_31	31. Can't get his or her mind off something once he or she starts thinking about it.	Social Responsiveness Scale	SRS
0,050388201	SCARED_P_SCARED_P_24	24. My child gets really frightened for no reason at all	Screen for Child Anxiety Related Disorders	SCARED
0,049933508	CBCL_CBCL_48	48. Not liked by other kids	Child Behavior Checklist	CBCL
0,049828521	CBCL_CBCL_88	88. Sulks a lot	Child Behavior Checklist	CBCL
-0,049698258	APQ_P_APQ_P_04	4. You volunteer to help with special activities that your child is involved with (such as sports, boy/girl scouts, church youth groups)	Alabama Parenting Questionnaire	APQ
-0,049600108	SympChck_CSC_40P	Actively disobeys or doesn't listen to adult rules (past	Symptom Checklist Parent report	SympChck-P
-0,049536531	SympChck_CSC_28P	Often has accidents when he/she wets the bed or self during the day (past	Symptom Checklist Parent report	SympChck-P
-0,049438064	Right_Putamen	Volume of the Right Putamen	Subcortical Volume	NA
0,049412247	right_fimbria	Volume of the right fimbria	Hippocampus Subfields	NA
0,049203696	SympChck_CSC_12P	(past	Symptom Checklist Parent report	SympChck-P
-0,048975422	SCQ_SCQ_06	6. Does she/he ever use words that she/he seems to have invented or made up her/himself; put things in odd, indirect ways; or use metaphorical ways of saying things (e.g., saying hot rain for steam)?	Social Communication Questionnaire	SCQ

Correlation coefficient (R)	Variable name in data	Item text	Instrument name	Instrument abbreviation (if applicable)
-0,04886589	PreInt_DevHx_m_birthage	Mother's age at birth of child	Interview-Developmental History	Dev.Hist.
0,048522617	CBCL_CBCL_71	71. Self-conscious or easily embarrassed	Child Behavior Checklist	CBCL
-0,048298555	SympChck_CSC_47C	Makes noises that he/she can't control (repeating sounds, sniffing) (current)	Symptom Checklist Parent report	SympChck-P
0,048128682	SRS_SRS_30	30. Becomes upset in a situation with lots of things going on.	Social Responsiveness Scale	SRS
0,048081871	SRS_SRS_22	22. Plays appropriately with children his or her age.	Social Responsiveness Scale	SRS
0,047863862	PreInt_Demos_Fam_P1_Sex	Sex	Interview-Demographics/Family	Demog.Fam.
0,047771159	SDQ_SDQ_01	Considerate of other people's feelings	Strength and Difficulties Questionnaire	SDQ
-0,047627168	APQ_P_APQ_P_31	31. The punishment you give your child depends on your mood	Alabama Parenting Questionnaire	APQ
0,047523578	SRS_SRS_05	5. Doesn't recognize when others are trying to take advantage of him or her.	Social Responsiveness Scale	SRS
0,04748412	SympChck_CSC_21C	Is unable to speak in specific situations, such as school, despite being able to speak without a problem in other situations (current)	Symptom Checklist Parent report	SympChck-P
0,047369555	APQ_SR_APQ_SR_29	29. Your parents leave the house and don't tell you where they are going	Alabama Parenting Questionnaire	APQ
-0,047112753	SRS_SRS_20	20. Shows unusual sensory interests (e.g., mouthing or spinning objects)	Social Responsiveness Scale	SRS

Correlation coefficient (R)	Variable name in data	Item text	Instrument name	Instrument abbreviation (if applicable)
		or strange ways of playing with toys		
0,046980934	lh_LP	Volume of the left LP	Thalamic Nuclei	NA
0,046954085	CBCL_CBCL_80	80. Stares blankly	Child Behavior Checklist	CBCL
0,046616521	CBCL_CBCL_TP	Thought Problems Raw Score	Child Behavior Checklist	CBCL
0,046526834	NLES_P_NLES_P_11a	11a. The child's mother or father acted badly in front of the child's friends (did things like yelled at them or criticized them).	Negative Life Events Scale	NLES
0,046518098	SympChck_CSC_11C	Has anxiety attacks, where out of the blue he/she suddenly feels scared (current)	Symptom Checklist Parent report	SympChck-P
0,045521998	ASSQ_ASSQ_12	lacks empathy (i.e. tends to see things only from his/her own perspective, and has troubles seeing things from other's perspective)	Autism Spectrum Screening Questionnaire	ASSQ
0,045439796	SDQ_SDQ_24	Many fears, easily scared	Strength and Difficulties Questionnaire	SDQ
0,045372268	PreInt_EduHx_recent_grades	Recent typical academic performance:	Interview-Education and Social History	Edu/Soc.Hist.
0,045157571	SRS_SRS_16	16. Avoids eye contact or has unusual eye contact.	Social Responsiveness Scale	SRS
0,044954263	SympChck_CSC_41C	Frequently lies (current)	Symptom Checklist Parent report	SympChck-P
-0,044502775	CBCL_CBCL_78	78. Inattentive or easily distracted	Child Behavior Checklist	CBCL
0,044484769	PreInt_FamHx_RDC_mmmdk	Don't know mother's maternal grandmother's current age	Family History/Research Diagnostic Criteria	Fam.Med.Hist
-0,044394367	EHQ_EHQ_08	Using a Broom (upper hand)	Edinburgh Handedness Questionnaire	EHQ

Correlation coefficient (R)	Variable name in data	Item text	Instrument name	Instrument abbreviation (if applicable)
0,043877711	PreInt_DevHx_growth_concerns	Have there been any concerns about your child's growth?	Interview-Developmental History	Dev.Hist.
-0,043876646	SDQ_SDQ_12	Often fights with other children or bullies them (for 11-17 year olds: Often fights with other youth or bullies them)	Strength and Difficulties Questionnaire	SDQ
-0,043752833	ASSQ_ASSQ_09	expresses sounds involuntarily; clears throat, grunts, smacks, cries or screams	Autism Spectrum Screening Questionnaire	ASSQ
-0,04334836	SympChck_CSC_39C	Argues or talks back to adults, more than others his/her age (current)	Symptom Checklist Parent report	SympChck-P
0,043226544	SympChck_CSC_15P	Worries excessively about harm occuring to parents/guardians (past	Symptom Checklist Parent report	SympChck-P
-0,043023283	PreInt_EduHx_afterschool_other	Other	Interview-Education and Social History	Edu/Soc.Hist.
-0,042996189	CTOPP_CTOPP_NR_S	Nonword Repetition scaled score	Comprehensive Test of Phonological Processing	CTOPP-2
0,042609093	lh_PuL	Volume of the left PuL	Thalamic Nuclei	NA
0,04242172	ICU_P_ICU_P_05	5. Feels bad or guilty when he/she has done something wrong.	Inventory of Callous-Unemotional Traits Parent	ICU
-0,042278948	lh_PuM	Volume of the left PuM	Thalamic Nuclei	NA
-0,042194927	ASSQ_ASSQ_19	is poor at games; no idea of cooperating in a team, scores ‘own goals’	Autism Spectrum Screening Questionnaire	ASSQ
-0,041837515	CBCL_CBCL_87	87. Sudden changes in mood or feelings	Child Behavior Checklist	CBCL
-0,041798819	SCQ_SCQ_17	17. Does she/he ever injure her/himself deliberately, such as by biting her/his arm or	Social Communication Questionnaire	SCQ

Correlation coefficient (R)	Variable name in data	Item text	Instrument name	Instrument abbreviation (if applicable)
		banging her/his head?		
-0,041164765	CBCL_CBCL_C	C Score Raw Score	Child Behavior Checklist	CBCL
-0,041068746	SCQ_SCQ_08	8. Does she/he ever have things that she/he seems to have to do in a very particular way or order or rituals that she/he insists that you go though?	Social Communication Questionnaire	SCQ
0,040926957	right_Medial_nucleus	Volume of the right Medial nucleus	Amygdala Nuclei	NA
0,040818693	SympChck_CSC_46P	Notices muscles moving uncontrollably (blinking a lot, shrugging) (past	Symptom Checklist Parent report	SympChck-P
0,040739757	PreInt_DevHx_delivery	Delivery:	Interview-Developmental History	Dev.Hist.
-0,040707582	PreInt_DevHx_temp_09	Easily adaptable	Interview-Developmental History	Dev.Hist.
-0,040666536	WIAT_WIAT_Word_P	Word Reading Percentile Rank	Wechsler Individual Achievement Test	WIAT
0,040652192	ASSQ_ASSQ_08	has a different voice or speech	Autism Spectrum Screening Questionnaire	ASSQ
0,040388338	PreInt_FamHx_RDC_fmmdk	Don't know father's maternal grandmother's current age	Family History/Research Diagnostic Criteria	Fam.Med.Hist
-0,040297615	Barratt_Barratt_Total_Occ	Occupation total score	Barratt Simplified Measure of Social Status	BSMSS
0,04020568	Basic_Demos_Sex	Sex	Basic Demographic Information	Basic_Demos
0,040056551	ICU_P_ICU_P_21	21. The feelings of others are unimportant to him/her.	Inventory of Callous-Unemotional Traits Parent	ICU
-0,039867302	ICU_P_ICU_P_17	17. Tries not to hurt others' feelings.	Inventory of Callous-Unemotional Traits Parent	ICU
0,03982655	SympChck_CSC_21P	Is unable to speak in specific situations, such	Symptom Checklist Parent report	SympChck-P

Correlation coefficient (R)	Variable name in data	Item text	Instrument name	Instrument abbreviation (if applicable)
		as school, despite being able to speak without a problem in other situations (past		
0,039786376	PreInt_FamHx_RDC_fmfdk	Don't know father's maternal grandfather's current age	Family History/Research Diagnostic Criteria	Fam.Med.Hist
0,039782016	CBCL_CBCL_39	39. Hangs around with others who get in trouble	Child Behavior Checklist	CBCL
0,039619354	APQ_P_APQ_P_29	29. You don't tell your child where you are going	Alabama Parenting Questionnaire	APQ
0,039501318	PreInt_FamHx_RDC_fmdk	Don't know father's mother's current age	Family History/Research Diagnostic Criteria	Fam.Med.Hist
-0,039496673	PreInt_DevHx_preg_symp_11	Swollen ankles	Interview-Developmental History	Dev.Hist.
0,039188072	ASSQ_ASSQ_01	is old-fashioned or precocious	Autism Spectrum Screening Questionnaire	ASSQ
0,039068566	ARI_S_ARI_S_01	I am easily annoyed by others	Affective Reactivity Index	ARI
-0,038262873	PreInt_EduHx_music	Music	Interview-Education and Social History	Edu/Soc.Hist.
0,038260915	rh_entorhinal_area	Cortical area in rh entorhinal	Cortical Area	NA
0,038038701	SympChck_CSC_09C	Hears, sees, or smells things that other people cannot (past	Symptom Checklist Parent report	SympChck-P
0,038016064	SRS_SRS_61	61. Is inflexible, has a hard time changing his or her mind.	Social Responsiveness Scale	SRS
-0,037913787	WIAT_WIAT_Word_Stnd	Word Reading Standard Score	Wechsler Individual Achievement Test	WIAT
0,037891374	EHQ_EHQ_11	Holding a Computer Mouse	Edinburgh Handedness Questionnaire	EHQ
0,037799198	CBCL_CBCL_56E	56E. Rashes or other skin problems	Child Behavior Checklist	CBCL
-0,037741936	APQ_P_APQ_P_26	26. you attend PTA meetings, parent/teacher	Alabama Parenting Questionnaire	APQ

Correlation coefficient (R)	Variable name in data	Item text	Instrument name	Instrument abbreviation (if applicable)
		conderences, or other meetings at your child's school		
-0,037261529	SRS_SRS_43	43. Separates easily from caregivers.	Social Responsiveness Scale	SRS
0,036606395	SRS_SRS_15	15. Is able to understand the meaning of other people's tone of voice and facial expressions.	Social Responsiveness Scale	SRS
-0,036527234	SympChck_CSC_35C	Is often easily distracted (current)	Symptom Checklist Parent report	SympChck-P
0,036140632	SympChck_CSC_50C	Is preoccupied with very specific objects, routines, or interests (current)	Symptom Checklist Parent report	SympChck-P
-0,036117334	SRS_SRS_63	63. Touches others in an unusual way (e.g., he or she may touch someone just to make contact and then walk away without saying anything).	Social Responsiveness Scale	SRS
0,03609296	CBCL_CBCL_32	32. Feels he/she has to be perfect	Child Behavior Checklist	CBCL
0,036017019	rh_temporalpole_thickness	Cortical thickness in rh temporalpole	Cortical Thickness	NA
-0,036014259	APQ_P_APQ_P_36	36. You take away privileges or money from your child as punishment	Alabama Parenting Questionnaire	APQ
0,035953483	PreInt_FamHx_RDC_mmfdk	Don't know mother's paternal grandmother's current age	Family History/Research Diagnostic Criteria	Fam.Med.Hist
-0,035952016	CBCL_CBCL_81	81. Steals at home	Child Behavior Checklist	CBCL
0,035733063	ICU_P_ICU_P_02	2. Does not seem to know “right” from “wrong”.	Inventory of Callous-Unemotional Traits Parent	ICU
-0,035713942	SympChck_CSC_31C	Is underweight for his/her age and height (current)	Symptom Checklist Parent report	SympChck-P
0,035637597	rh_LP	Volume of the right LP	Thalamic Nuclei	NA

Correlation coefficient (R)	Variable name in data	Item text	Instrument name	Instrument abbreviation (if applicable)
-0,035568189	Right_Inf_Lat_Vent	Volume of the Right Inf Lat Vent	Subcortical Volume	NA
0,03550022	rh_rostralanteriorcingulate_area	Cortical area in rh rostralanteriorcingulate	Cortical Area	NA
0,035109238	SDQ_SDQ_Generating_Impact	Generating Impact Scores	Strength and Difficulties Questionnaire	SDQ
-0,035051742	rh_lateralorbitofrontal_area	Cortical area in rh lateralorbitofrontal	Cortical Area	NA
-0,035043901	APQ_SR_APQ_SR_09	9. Your mom asks you about your day in school	Alabama Parenting Questionnaire	APQ
0,035017991	SCARED_P_SCARED_P_37	37. My child worries about things that have already happened	Screen for Child Anxiety Related Disorders	SCARED
-0,034899267	ARI_P_ARI_P_Total_Score	Total Score	Affective Reactivity Index	ARI
0,034709685	SCARED_P_SCARED_P_36	36. My child is scared to go to school	Screen for Child Anxiety Related Disorders	SCARED
-0,034684006	SDQ_SDQ_25	Good attention span, sees chores or homework through to the end	Strength and Difficulties Questionnaire	SDQ
0,034492537	PreInt_EduHx_EI	Does your child have EI services?	Interview-Education and Social History	Edu/Soc.Hist.
0,034394807	SRS_SRS_51	51. Has difficulty answering questions directly and ends up talking around the subject.	Social Responsiveness Scale	SRS
-0,034342339	SCARED_P_SCARED_P_16	16. My child has nightmares about something bad happening to his/her parents	Screen for Child Anxiety Related Disorders	SCARED
0,033815623	APQ_SR_APQ_SR_01	1. You have a friendly talk with your mom	Alabama Parenting Questionnaire	APQ
-0,033812279	WIAT_WIAT_Pseudo_P	Pseudo-word Decoding Percentile Rank	Wechsler Individual Achievement Test	WIAT
-0,033544362	CBCL_CBCL_64	64. Prefers being with younger kids	Child Behavior Checklist	CBCL

Correlation coefficient (R)	Variable name in data	Item text	Instrument name	Instrument abbreviation (if applicable)
0,03326166	Right_Cerebellum_Cortex	Volume of the Right Cerebellum Cortex	Subcortical Volume	NA
0,033121203	SDQ_SDQ_19	Picked on or bullied by other children (for 11-17 year olds: Picked on or bullied by other youth)	Strength and Difficulties Questionnaire	SDQ
-0,033109779	DTS_DTS_12	12. My feelings of distress or being upset scare me.	Distress Tolerance Scale	DTS
0,033041782	SympChck_CSC_50P	Is preoccupied with very specific objects, routines, or interests (past	Symptom Checklist Parent report	SympChck-P
-0,032964857	WIAT_WIAT_LCRV_P	Listening Comprehension Receptive Vocabulary Percentile Rank	Wechsler Individual Achievement Test	WIAT
0,032853326	SCQ_SCQ_27	27. Does she/he smile back if someone smiles at her/him?	Social Communication Questionnaire	SCQ
-0,032625519	DTS_DTS_Total	DTS Total Score	Distress Tolerance Scale	DTS
0,032455936	SWAN_SWAN_07	7. Keeps track of things necessary for activities (doesn't lose them)	The Strengths and Weaknesses Assessment of Normal Behavior	SWAN
-0,032410504	SympChck_CSC_14P	Worries about being separated from parent/guardian because of getting lost or kidnapped (past	Symptom Checklist Parent report	SympChck-P
0,032130593	NLES_P_NLES_P_03a	3a. The child suffered from a serious physical illness, injury, or extreme pain (something that required rest of one week in bed, hospitalization, or surgery).	Negative Life Events Scale	NLES
0,032008413	APQ_SR_APQ_SR_03	3. Your parents threaten to punish you and then not do it	Alabama Parenting Questionnaire	APQ
-0,031866631	lh_medialorbitofrontal_area	Cortical area in lh medialorbitofrontal	Cortical Area	NA

Correlation coefficient (R)	Variable name in data	Item text	Instrument name	Instrument abbreviation (if applicable)
0,031795202	SRS_SRS_04	4. When under stress, he or she shows rigid or inflexible patterns of behavior that seem odd.	Social Responsiveness Scale	SRS
0,031776681	CBCL_CBCL_44	44. Bites fingernails	Child Behavior Checklist	CBCL
-0,031643433	PreInt_DevHx_newborn_problems	Newborn period:	Interview-Developmental History	Dev.Hist.
-0,031470383	lh_inferiortemporal_area	Cortical area in lh inferiortemporal	Cortical Area	NA
0,031362169	SRS_SRS_14	14. Is not well coordinated.	Social Responsiveness Scale	SRS
-0,031249783	WIAT_WIAT_LCRV_Std	Listening Comprehension Receptive Vocabulary Standard Score	Wechsler Individual Achievement Test	WIAT
0,031225198	rh_temporalpole_area	Cortical area in rh temporalpole	Cortical Area	NA
-0,031147163	SympChck_CSC_22P	Has intense fears of specific animals, situations, or anything else (past	Symptom Checklist Parent report	SympChck-P
0,030798233	ICU_P_ICU_P_16	16. Apologizes (“says he/she is sorry”) to persons he/she has hurt.	Inventory of Callous-Unemotional Traits Parent	ICU
-0,030670343	SRS_SRS_01	1. Seems much more fidgety in social situations than when alone.	Social Responsiveness Scale	SRS
-0,029997732	APQ_SR_APQ_SR_26A	26A. How about your dad?	Alabama Parenting Questionnaire	APQ
0,02981912	rh_AV	Volume of the right AV	Thalamic Nuclei	NA
0,029605587	PreInt_DevHx_temp_11	Problems with social relatedness	Interview-Developmental History	Dev.Hist.
0,029327745	CBCL_CBCL_91	91. Talks about killing self	Child Behavior Checklist	CBCL
0,029044802	rh_temporalpole_volume	Cortical volume in rh temporalpole	Cortical Volume	NA
0,02877933	CBCL_CBCL_56D	56D.A. Problems with eyes (not if corrected by glasses	Child Behavior Checklist	CBCL

Correlation coefficient (R)	Variable name in data	Item text	Instrument name	Instrument abbreviation (if applicable)
0,028195803	SympChck_CSC_12C	(current)	Symptom Checklist Parent report	SympChck-P
0,028094004	SDQ_SDQ_09	Helpful if someone is hurt, upset or feeling ill	Strength and Difficulties Questionnaire	SDQ
-0,028019581	APQ_SR_APQ_SR_25	25. Your parents do not punish you when you have done something wrong	Alabama Parenting Questionnaire	APQ
0,027946447	NLES_P_NLES_P_20a	20a. The child's mother acted very worried, upset, or sad, not because of something the child did.	Negative Life Events Scale	NLES
0,027846843	left_parasubiculum	Volume of the left parasubiculum	Hippocampus Subfields	NA
0,027636039	SRS_SRS_26	26. Offers comfort to others when they are sad.	Social Responsiveness Scale	SRS
-0,027546759	rh_inferiortemporal_area	Cortical area in rh inferiortemporal	Cortical Area	NA
0,027499588	CBCL_CBCL_30	30. Fears going to school	Child Behavior Checklist	CBCL
-0,027234871	FGC_FGC_PU_Zone	Push-up fitness zone	FitnessGram Child	FGC
0,027068842	SWAN_SWAN_04	4. Follows through on instructions and finishes school work and chores	The Strengths and Weaknesses Assessment of Normal Behavior	SWAN
0,026928611	SRS_SRS_38	38. Responds appropriately to mood changes in others (e.g., when a friend's or playmate's mood changes from happy to sad).	Social Responsiveness Scale	SRS
-0,026487904	SympChck_CSC_15C	Worries excessively about harm occuring to parents/guardians (current)	Symptom Checklist Parent report	SympChck-P
-0,02635986	PreInt_EduHx_weakness_english	English	Interview-Education and Social History	Edu/Soc.Hist.
-0,026073473	SympChck_CSC_39P	Argues or talks back to adults, more than others his/her age (past	Symptom Checklist Parent report	SympChck-P

Correlation coefficient (R)	Variable name in data	Item text	Instrument name	Instrument abbreviation (if applicable)
0,026070163	ASSQ_ASSQ_04	accumulates facts on certain subjects (good rote memory) but does not really understand the meaning	Autism Spectrum Screening Questionnaire	ASSQ
-0,026029563	SympChck_CSC_16P	Often does not want to go to school, due to worry about parent/guardian separation (past	Symptom Checklist Parent report	SympChck-P
0,02588755	PreInt_FamHx_RDC_mffdk	Mother's paternal grandmother current age range, or age range at death:	Family History/Research Diagnostic Criteria	Fam.Med.Hist
-0,025807449	CBCL_CBCL_92	92. Talks or walks in sleep	Child Behavior Checklist	CBCL
0,025760998	ICU_P_ICU_P_08	8. Is concerned about the feelings of others.	Inventory of Callous-Unemotional Traits Parent	ICU
-0,025745442	DTS_DTS_02	2. When I feel distressed or upset, all I can think about is how bad I feel	Distress Tolerance Scale	DTS
0,025449916	ARI_P_ARI_P_05	Gets angry frequently	Affective Reactivity Index	ARI
0,024992912	NLES_P_NLES_P_06a	6a. People in the child's family (such as his/her parents, brothers or sisters) physically hit each other hard or hurt each other.	Negative Life Events Scale	NLES
-0,024570821	WIAT_WIAT_Spell_P	Spelling Percentile Rank	Wechsler Individual Achievement Test	WIAT
-0,024466752	SympChck_CSC_49C	Feels really upset when there is an unexpected change in his/her schedule (current)	Symptom Checklist Parent report	SympChck-P
-0,023905057	rh_medialorbitofrontal_area	Cortical area in rh medialorbitofrontal	Cortical Area	NA
0,023841937	PreInt_EduHx_family_religious	Was your child raised in a particular religious faith?	Interview-Education and Social History	Edu/Soc.Hist.

Correlation coefficient (R)	Variable name in data	Item text	Instrument name	Instrument abbreviation (if applicable)
0,023490012	ASSQ_ASSQ_14	has a deviant style of gaze (e.g. may range from not looking people in the eye, to the other extreme of staring directly at people to the point it makes them uncomfortable)	Autism Spectrum Screening Questionnaire	ASSQ
-0,023320051	SympChck_CSC_47P	Makes noises that he/she can't control (repeating sounds, sniffing) (past	Symptom Checklist Parent report	SympChck-P
-0,022890674	APQ_P_APQ_P_ID	Inconsistent Discipline Score	Alabama Parenting Questionnaire	APQ
-0,022673521	CBCL_CBCL_SP_T	Social Problems T Score	Child Behavior Checklist	CBCL
0,02261128	PreInt_EduHx_IEP	Does your child have an Individualized Education Plan (IEP)?	Interview-Education and Social History	Edu/Soc.Hist.
0,022234281	SRS_SRS_28	28. Thinks or talks about the same thing over and over.	Social Responsiveness Scale	SRS
0,022142219	APQ_SR_APQ_SR_08	8. You talk your parents out of punishing you after you have done something wrong	Alabama Parenting Questionnaire	APQ
-0,021981977	SCQ_SCQ_38	38. If you come into a room and start talkking to her/him without calling her/his name, does she/he usually look up and pay attention to you?	Social Communication Questionnaire	SCQ
-0,021912234	CBCL_CBCL_01	1. Acts too young for his/her age	Child Behavior Checklist	CBCL
-0,02153732	DTS_DTS_absorption	Absorption subscale	Distress Tolerance Scale	DTS
-0,02126562	PreInt_DevHx_temp_05	Sleeping difficulties	Interview-Developmental History	Dev.Hist.
0,02111732	SCQ_SCQ_26	26. Does she/he usually look at you directly in the face when doing	Social Communication Questionnaire	SCQ

Correlation coefficient (R)	Variable name in data	Item text	Instrument name	Instrument abbreviation (if applicable)
		things with you or talking with you?		
0,021071817	CBCL_CBCL_43	43. Lying or cheating	Child Behavior Checklist	CBCL
0,020920851	PreInt_FamHx_RDC_mfmdk	Don't know mother's maternal grandmother's current age	Family History/Research Diagnostic Criteria	Fam.Med.Hist
0,020624789	lh_PuI	Volume of the left PuI	Thalamic Nuclei	NA
-0,020531647	PreInt_DevHx_temp_02	Difficult to soothe when upset	Interview-Developmental History	Dev.Hist.
0,020419398	PreInt_DevHx_preg_symp_04	Diabetes	Interview-Developmental History	Dev.Hist.
0,020400349	CBCL_CBCL_Total	Total Raw Score	Child Behavior Checklist	CBCL
-0,020169395	SDQ_SDQ_26	Overall, do you think that your child has difficulties in one or more of the following areas: emotions, concentration, behavior or being able to get on with other people?	Strength and Difficulties Questionnaire	SDQ
0,020068214	PreInt_EduHx_videocomputergames	Playing video/computer games	Interview-Education and Social History	Edu/Soc.Hist.
-0,019996262	APQ_P_APQ_P_13	13. You compliment your child when he/she has done something well	Alabama Parenting Questionnaire	APQ
0,0199684	SRS_SRS_17	17. Recognizes when something is unfair.	Social Responsiveness Scale	SRS
0,019485412	ASSQ_ASSQ_23	has special routines; insists on no change (i.e. may need to have exactly the same change; troubles with even the slightest change in his/her environment, or routines or activities)	Autism Spectrum Screening Questionnaire	ASSQ
0,019298033	ICU_P_ICU_P_18	18. Shows no remorse when	Inventory of Callous-	ICU

Correlation coefficient (R)	Variable name in data	Item text	Instrument name	Instrument abbreviation (if applicable)
		he/she has done something wrong.	Unemotional Traits Parent	
0,018642913	CBCL_CBCL_56H	56H.A. Other	Child Behavior Checklist	CBCL
0,018526524	CBCL_CBCL_66	66. Repeats certain acts over and over; compulsions	Child Behavior Checklist	CBCL
-0,018387483	PreInt_DevHx_preg_dur	Duration of pregnancy (weeks)	Interview-Developmental History	Dev.Hist.
0,018292141	PreInt_FamHx_RDC_mfdk	Don't know mother's mother's current age	Family History/Research Diagnostic Criteria	Fam.Med.Hist
0,017770818	SRS_SRS_50	50. Has repetitive, odd behaviors such as hand flapping or rocking.	Social Responsiveness Scale	SRS
0,01751826	PreInt_EduHx_afterschoolteams	Does your child belong to any groups, sports teams, or organizations?	Interview-Education and Social History	Edu/Soc.Hist.
0,017505703	CBCL_CBCL_Total_T	Total T Score	Child Behavior Checklist	CBCL
-0,017299529	SCQ_SCQ_14	14. Does she/he ever seem to be unusually interested in the sight, feel, sound, taske, or smell of things or people?	Social Communication Questionnaire	SCQ
-0,017218146	APQ_P_APQ_P_40	40. You calmly explain to your child why his/her behavior was wrong when he/she misbehaves	Alabama Parenting Questionnaire	APQ
-0,017139685	Left_Cerebellum_Cortex	Volume of the Left Cerebellum Cortex	Subcortical Volume	NA
0,017129736	SRS_SRS_53	53. Talks to people with an unusual tone of voice (e.g., talks like a robot or like he or she is giving a lecture).	Social Responsiveness Scale	SRS
-0,017020409	SympChck_CSC_35P	Is often easily distracted (past	Symptom Checklist Parent report	SympChck-P
-0,017003628	APQ_SR_APQ_SR_40	40. Your parents calmly explain to you why your	Alabama Parenting Questionnaire	APQ

Correlation coefficient (R)	Variable name in data	Item text	Instrument name	Instrument abbreviation (if applicable)
		behavior was wrong when you misbehave		
0,016971986	PreInt_DevHx_temp_03	Colic	Interview-Developmental History	Dev.Hist.
-0,016622446	CBCL_CBCL_AP_T	Attention Problems T Score	Child Behavior Checklist	CBCL
-0,016468806	APQ_P_APQ_P_03	3. You threaten to punish your child and then do not actually punish him/her	Alabama Parenting Questionnaire	APQ
-0,015904401	DTS_DTS_05	5. There's nothing worse than feeling distressed or upset	Distress Tolerance Scale	DTS
-0,015637303	right_parasubiculum	Volume of the right parasubiculum	Hippocampus Subfields	NA
0,015423921	CBCL_CBCL_33	33. Feels or complains that no one loves him/her	Child Behavior Checklist	CBCL
-0,015417967	lh_temporalpole_area	Cortical area in lh temporalpole	Cortical Area	NA
0,01517131	SympChck_CSC_34C	Has trouble paying attention, and it affects school work or performance (current)	Symptom Checklist Parent report	SympChck-P
0,015089693	CBCL_CBCL_34	34. Feels others are out to get him/her	Child Behavior Checklist	CBCL
0,014961858	PreInt_DevHx_preg_symp_01	Spotting or vaginal bleeding	Interview-Developmental History	Dev.Hist.
0,014871264	PreInt_DevHx_birthweight_lbs	Birth weight of child (lbs)	Interview-Developmental History	Dev.Hist.
-0,014778265	DTS_DTS_10	10. Being distressed or upset is always a major ordeal for me	Distress Tolerance Scale	DTS
0,014773421	NLES_P_NLES_P_18a	18a. A close friend of the child moved away	Negative Life Events Scale	NLES
-0,014712824	SympChck_CSC_48C	Has unusual physical mannerisms (rocked body or flapping hands) (current)	Symptom Checklist Parent report	SympChck-P

Correlation coefficient (R)	Variable name in data	Item text	Instrument name	Instrument abbreviation (if applicable)
0,014659	CBCL_CBCL_62	62. Poorly coordinated or clumsy	Child Behavior Checklist	CBCL
-0,014566927	WIAT_WIAT_Spell_Stnd	Spelling Standard Score	Wechsler Individual Achievement Test	WIAT
0,014561431	APQ_SR_APQ_SR_31	31. The punishment your parents give depends on their mood	Alabama Parenting Questionnaire	APQ
0,014252331	SRS_SRS_57	57. Gets teased a lot.	Social Responsiveness Scale	SRS
-0,014210514	WIAT_WIAT_Pseudo_Stnd	Pseudo-word Decoding Standard Score	Wechsler Individual Achievement Test	WIAT
-0,01413417	ColorVision_CV_Score	Color Vision Score	Ishihara Color Vision Test	ColorVision
0,014046509	SDQ_SDQ_Prosocial	Prosocial Scale	Strength and Difficulties Questionnaire	SDQ
0,014021348	ICU_P_ICU_P_13	13. Easily admits to being wrong.	Inventory of Callous-Unemotional Traits Parent	ICU
-0,014004166	lh_entorhinal_volume	Cortical volume in lh entorhinal	Cortical Volume	NA
-0,013910217	SCQ_SCQ_13	13. Does she/he ever have any special interests that are unusual in their intensity but otherwise appropriate for her/his age and peer group (e.g., trains or dinosaurs)?	Social Communication Questionnaire	SCQ
0,013739291	PreInt_Demos_Home_living_17	Child lives with Other	Interview-Demographic and Household Information	Demog.Household.
-0,013688751	SCARED_P_SCARED_P_31	31. My child worries that something bad will happen to his/her parents	Screen for Child Anxiety Related Disorders	SCARED
0,013680415	CBCL_CBCL_86	86. Stubborn, sullen, or irritable	Child Behavior Checklist	CBCL
0,013580029	SRS_SRS_13	13. Is awkward is turn-taking interactions with peers (e.g., doesn't seem to	Social Responsiveness Scale	SRS

Correlation coefficient (R)	Variable name in data	Item text	Instrument name	Instrument abbreviation (if applicable)
		understand the give-and-take of conversations)		
0,013454985	SDQ_SDQ_18	Often lies or cheats	Strength and Difficulties Questionnaire	SDQ
0,013180207	PreInt_DevHx_temp_01	Easy to soothe when upset	Interview-Developmental History	Dev.Hist.
-0,012982499	CBCL_CBCL_26	26. Doesn't seem to feel guilty after misbehaving	Child Behavior Checklist	CBCL
-0,012892562	Left_Inf_Lat_Vent	Volume of the Left Inf Lat Vent	Subcortical Volume	NA
-0,012836195	DTS_DTS_01	1. Feeling distressed or upset is unbearable to me.	Distress Tolerance Scale	DTS
0,012813015	APQ_P_APQ_P_25	25. Your child is not punished when he/she has done something wrong	Alabama Parenting Questionnaire	APQ
0,012478846	CBCL_CBCL_83	83. Stores up too many things he/she doesn't need	Child Behavior Checklist	CBCL
0,01243497	PreInt_EduHx_current_religious	Is he/she religious now?	Interview-Education and Social History	Edu/Soc.Hist.
0,012297048	SCQ_SCQ_04	4. Does she/he ever use socially inappropriate questions or statements? For example, does she/he ever regularly ask personal questions or make personal comments at awkward times?	Social Communication Questionnaire	SCQ
0,012122657	SDQ_SDQ_16	Nervous in new situations, easily loses confidence	Strength and Difficulties Questionnaire	SDQ
-0,011917201	DTS_DTS_regulation	Regulation subscale	Distress Tolerance Scale	DTS
0,011850579	CBCL_CBCL_25	25. Doesn't get along well with other kids	Child Behavior Checklist	CBCL
-0,011673037	ASSQ_ASSQ_07	invents idiosyncratic words and expressions (i.e. makes up his or	Autism Spectrum Screening Questionnaire	ASSQ

Correlation coefficient (R)	Variable name in data	Item text	Instrument name	Instrument abbreviation (if applicable)
		her own words, expressions or names for things)		
0,011606529	PreInt_DevHx_temp_08	Baby was "limp" or stiff	Interview-Developmental History	Dev.Hist.
0,011573273	CBCL_CBCL_03	3. Argues a lot	Child Behavior Checklist	CBCL
0,011290659	CBCL_CBCL_RBB	Rule Breaking Behavior Raw Score	Child Behavior Checklist	CBCL
-0,011261319	PreInt_EduHx_strength_english	English	Interview-Education and Social History	Edu/Soc.Hist.
0,010795603	PreInt_EduHx_strength_other	Other	Interview-Education and Social History	Edu/Soc.Hist.
0,010609678	lh_CL	Volume of the left CL	Thalamic Nuclei	NA
0,010520555	SympChck_CSC_46C	Notices muscles moving uncontrollably (blinking a lot, shrugging) (current)	Symptom Checklist Parent report	SympChck-P
-0,0104572	SympChck_CSC_27P	Feels like he/she has to do certain things in a very specific way (handwashing, checking, doing things multiple times) (past	Symptom Checklist Parent report	SympChck-P
0,010315305	rh_entorhinal_volume	Cortical volume in rh entorhinal	Cortical Volume	NA
-0,010174833	SCQ_SCQ_15	15. Does she/he ever have any mannerisms or odd ways of moving her/his hands or fingers, such as flapping or moving her/his fingers in front or her/his eyes?	Social Communication Questionnaire	SCQ
0,010173925	SWAN_SWAN_02	2. Sustains attention on tasks or play activities	The Strengths and Weaknesses Assessment of Normal Behavior	SWAN
-0,00998829	PreInt_FamHx_RDC_omoves1	Number of times changed address (age 0-5):	Family History/Research Diagnostic Criteria	Fam.Med.Hist
0,009782746	SympChck_CSC_48P	Has unusual physical mannerisms (rocked body or	Symptom Checklist Parent report	SympChck-P

Correlation coefficient (R)	Variable name in data	Item text	Instrument name	Instrument abbreviation (if applicable)
		flapping hands) (past		
0,009667489	CBCL_CBCL_38	38. Gets teased a lot	Child Behavior Checklist	CBCL
-0,009483204	APQ_P_APQ_P_12	12. You give up trying to get your child to obey you because it's too much trouble	Alabama Parenting Questionnaire	APQ
0,009165056	APQ_SR_APQ_SR_ID	Inconsistent Discipline Score	Alabama Parenting Questionnaire	APQ
-0,009127278	CBCL_CBCL_AP	Attention Problems Raw Score	Child Behavior Checklist	CBCL
0,009090882	NLES_P_NLES_P_14a	14a. The child's mother or father was arrested or sent to jail.	Negative Life Events Scale	NLES
-0,008987039	SDQ_SDQ_Difficulties_Total	Total Difficulties Score	Strength and Difficulties Questionnaire	SDQ
0,00878097	CELF_CELF_ExceedCutoff	Meets criterion score?	Clinical Evaluation of Language Fundamentals, Fifth Edition	CELF-5
-0,008585599	DTS_DTS_03	3. I can’t handle feeling distressed or upset.	Distress Tolerance Scale	DTS
0,008568514	ASSQ_ASSQ_21	has involuntary face or body movements (i.e. any tics?)	Autism Spectrum Screening Questionnaire	ASSQ
-0,008552127	PreInt_DevHx_dev_normal	All developmental milestones within normal limits	Interview-Developmental History	Dev.Hist.
-0,008512869	rh_MDl	Volume of the right MDl	Thalamic Nuclei	NA
0,0084145	CBCL_CBCL_84	84. Strange behavior	Child Behavior Checklist	CBCL
0,008208451	CBCL_CBCL_31	31. Fears he/she might think or do something bad	Child Behavior Checklist	CBCL
0,008000303	SympChck_CSC_44C	Bullies, threatens, or intimidates others (current)	Symptom Checklist Parent report	SympChck-P
0,007805442	SCQ_SCQ_11	11. Does she/he ever have any interests that preoccupy her/him and might seem off to other people (e.g., traffic lights,	Social Communication Questionnaire	SCQ

Correlation coefficient (R)	Variable name in data	Item text	Instrument name	Instrument abbreviation (if applicable)
		drainpipes, or timetables?)		
0,007735687	PreInt_EduHx_school_difficulty	Did your child experience any difficulty starting school?	Interview-Education and Social History	Edu/Soc.Hist.
-0,007600562	Left_Putamen	Volume of the Left Putamen	Subcortical Volume	NA
0,007345585	SRS_SRS_45	45. Focuses his or her attention to where others are looking or listening.	Social Responsiveness Scale	SRS
-0,007330331	rh_precentral_thickness	Cortical thickness in rh precentral	Cortical Thickness	NA
-0,006988736	DTS_DTS_tolerance	Tolerance subscale	Distress Tolerance Scale	DTS
0,006561735	ColorVision_CV_Plate_04_R	Plate 04 Result	Ishihara Color Vision Test	ColorVision
-0,006507704	SCQ_SCQ_03	3. Does she/he ever use odd phrases or say the same thing over and over in almost exactly the same way (either phrases that she/he hears other people use or ones that she/he makes up?	Social Communication Questionnaire	SCQ
-0,006419428	Basic_Demos_Study_Site	Study Site	Basic Demographic Information	Basic_Demos
-0,00625448	SRS_SRS_42	42. Seems overly sensitive to sounds, textures, or smells.	Social Responsiveness Scale	SRS
-0,00592984	lh_PuA	Volume of the left PuA	Thalamic Nuclei	NA
-0,005761349	CBCL_CBCL_08	8. Can't concentrate, can't pay attention for long	Child Behavior Checklist	CBCL
-0,0056563	left_Medial_nucleus	Volume of the left Medial nucleus	Amygdala Nuclei	NA
0,005447854	PreInt_DevHx_temp_10	Slow to warm up	Interview-Developmental History	Dev.Hist.
0,00539195	SRS_SRS_AWR_T	Social Awareness T-Score	Social Responsiveness Scale	SRS
-0,005324804	PreInt_DevHx_complications	Compications at birth	Interview-Developmental History	Dev.Hist.

Correlation coefficient (R)	Variable name in data	Item text	Instrument name	Instrument abbreviation (if applicable)
-0,004764892	PreInt_DevHx_temp_07	Overly sensitive to sound	Interview-Developmental History	Dev.Hist.
-0,004706389	lh_lateralorbitofrontal_area	Cortical area in lh lateralorbitofrontal	Cortical Area	NA
-0,004687506	SRS_SRS_24	24. Has more difficulty than other children with changes in his or her routine.	Social Responsiveness Scale	SRS
0,004685778	CBCL_CBCL_24	24. Doesn't eat well	Child Behavior Checklist	CBCL
-0,004673829	Barratt_Barratt_P1_Occ	Parent 1 level of occupation	Barratt Simplified Measure of Social Status	BSMSS
0,004639948	ICU_P_ICU_P_09	9. Does not care if he/she is in trouble.	Inventory of Callous-Unemotional Traits Parent	ICU
0,004322133	SDQ_SDQ_22	Steals from home, school or elsewhere	Strength and Difficulties Questionnaire	SDQ
-0,004275165	lh_LGN	Volume of the left LGN	Thalamic Nuclei	NA
0,004217493	SRS_SRS_08	8. Behaves in ways that seem strange or bizarre.	Social Responsiveness Scale	SRS
0,004170568	CBCL_CBCL_56G	56G. Vomiting, throwing up	Child Behavior Checklist	CBCL
0,004040725	lh_caudalanteriorcingulate_area	Cortical area in lh caudalanteriorcingulate	Cortical Area	NA
-0,003910693	SympChck_CSC_49P	Feels really upset when there is an unexpected change in his/her schedule (past	Symptom Checklist Parent report	SympChck-P
-0,003861435	PreInt_DevHx_preg_symp_12	Family stress	Interview-Developmental History	Dev.Hist.
0,003381107	APQ_SR_APQ_SR_01A	1A. How about your dad?	Alabama Parenting Questionnaire	APQ
0,003300054	PreInt_DevHx_preg_symp_02	Emotional problems	Interview-Developmental History	Dev.Hist.
0,003226859	SWAN_SWAN_08	8. Ignores extraneous stimuli	The Strengths and Weaknesses Assessment of Normal Behavior	SWAN
-0,003157406	SympChck_CSC_44P	Bullies, threatens, or intimidates others (past	Symptom Checklist Parent report	SympChck-P

Correlation coefficient (R)	Variable name in data	Item text	Instrument name	Instrument abbreviation (if applicable)
0,003119021	rh_caudalanteriorcingulate_area	Cortical area in rh caudalanteriorcingulate	Cortical Area	NA
-0,003050397	APQ_SR_APQ_SR_38	38. Your parents hit you with a belt, switch, or other object when you have done something wrong	Alabama Parenting Questionnaire	APQ
0,002871519	SRS_SRS_AWR	Social Awareness Raw Score	Social Responsiveness Scale	SRS
-0,002813369	APQ_SR_APQ_SR_24	24. Your parents get so busy that they forget where you are and what you are doing	Alabama Parenting Questionnaire	APQ
0,002609938	PreInt_DevHx_lost_skills	Has your child lost any skills or abilities?	Interview-Developmental History	Dev.Hist.
-0,002469092	SympChck_CSC_31P	Is underweight for his/her age and height (past	Symptom Checklist Parent report	SympChck-P
0,002375496	SCQ_SCQ_07	7. Does she/he ever say the same thing over and over again?	Social Communication Questionnaire	SCQ
-0,002371145	APQ_SR_APQ_SR_39	39. Your parents yell or scream at you when you have done something wrong	Alabama Parenting Questionnaire	APQ
0,002251476	lh_AV	Volume of the left AV	Thalamic Nuclei	NA
0,001846271	ASSQ_ASSQ_24	shows idiosyncratic attachment to objects (i.e. may get strangely attached to objects as if they were people)	Autism Spectrum Screening Questionnaire	ASSQ
-0,00178673	SRS_SRS_19	19. Gets frustrated trying to get ideas across in conversations.	Social Responsiveness Scale	SRS
-0,001739846	PreInt_DevHx_preg_symp_05	High blood pressure	Interview-Developmental History	Dev.Hist.
-0,001686612	Left_vessel	Volume of the Left vessel	Subcortical Volume	NA
0,00165988	SCARED_P_SCARED_P_17	17. My child worries about going to school	Screen for Child Anxiety Related Disorders	SCARED

Correlation coefficient (R)	Variable name in data	Item text	Instrument name	Instrument abbreviation (if applicable)
0,001588695	PreInt_DevHx_temp_04	Eating difficulties	Interview-Developmental History	Dev.Hist.
-0,001559672	APQ_P_APQ_P_08	8. Your child talks you out of being punished after he/she has done something wrong	Alabama Parenting Questionnaire	APQ
0,001317407	ARI_P_ARI_P_03	Stays angry for a long time	Affective Reactivity Index	ARI
-0,001294253	APQ_SR_APQ_SR_Total	Other Discipline Practices Score (Not factored into total score but provides item level information)	Alabama Parenting Questionnaire	APQ
0,001275098	PreInt_Demos_Home_living_06	Child lives with Grandparents	Interview-Demographic and Household Information	Demog.Household.
-0,001025639	APQ_P_APQ_P_09	9. You ask your child about his/her day in school	Alabama Parenting Questionnaire	APQ
-0,001006073	SympChck_CSC_27C	Feels like he/she has to do certain things in a very specific way (handwashing, checking, doing things multiple times) (current)	Symptom Checklist Parent report	SympChck-P
-0,000970823	ASSQ_ASSQ_22	has difficulties in completing simple daily activities because of compulsory repetition of certain actions or thoughts (i.e. any habits that s/he just has to do?)	Autism Spectrum Screening Questionnaire	ASSQ
-0,000838745	APQ_P_APQ_P_39	39. You yell or scream at your child when he/she has done something wrong	Alabama Parenting Questionnaire	APQ
-0,000833481	lh_MDm	Volume of the left MDm	Thalamic Nuclei	NA
-0,000768162	CBCL_CBCL_94	94. Teases a lot	Child Behavior Checklist	CBCL
0,000575133	APQ_P_APQ_P_35	35. You slap your child when he/she has done something wrong	Alabama Parenting Questionnaire	APQ

Correlation coefficient (R)	Variable name in data	Item text	Instrument name	Instrument abbreviation (if applicable)
-0,000541947	APQ_P_APQ_P_22	22. You let your child out of a punishment early (like lift restrictions earlier than you originally said)	Alabama Parenting Questionnaire	APQ
-0,000189511	ASSQ_ASSQ_16	can be with other children but only on his/her terms	Autism Spectrum Screening Questionnaire	ASSQ

Table S4. List of all variables and mode loadings for mode 2

Correlation coefficient (R)	Variable name in data	Item text	Instrument name	Instrument abbreviation (if applicable)
-0,598935867	SRS_SRS_SCI_T	Social Communication and Interaction T-Score	Social Responsiveness Scale	SRS
-0,592476289	SRS_SRS_SCI	Social Communication and Interaction Raw Score	Social Responsiveness Scale	SRS
-0,592232689	SRS_SRS_Total_T	Total T-Score	Social Responsiveness Scale	SRS
-0,585752675	SRS_SRS_Total	Total Raw Score	Social Responsiveness Scale	SRS
0,575773056	lh_WhiteSurfArea_area	Cortical area in lh WhiteSurfArea	Cortical Area	NA
0,571263842	rh_WhiteSurfArea_area	Cortical area in rh WhiteSurfArea	Cortical Area	NA
-0,563984655	SRS_SRS_COM_T	Social Communication T-Score	Social Responsiveness Scale	SRS
-0,549659277	SRS_SRS_COM	Social Communication Raw Score	Social Responsiveness Scale	SRS
-0,535434733	SRS_SRS_COG_T	Social Cognition T-Score	Social Responsiveness Scale	SRS
-0,533833999	SRS_SRS_COG	Social Cognition Raw Score	Social Responsiveness Scale	SRS
-0,525072816	CBCL_CBCL_Total	Total Raw Score	Child Behavior Checklist	CBCL
-0,5241908	CBCL_CBCL_Total_T	Total T Score	Child Behavior Checklist	CBCL
-0,511271219	SDQ_SDQ_Difficulties_Total	Total Difficulties Score	Strength and Difficulties Questionnaire	SDQ
-0,493951842	CBCL_CBCL_C	C Score Raw Score	Child Behavior Checklist	CBCL
-0,492514623	CBCL_CBCL_AP_T	Attention Problems T Score	Child Behavior Checklist	CBCL
-0,492018352	CBCL_CBCL_AP	Attention Problems Raw Score	Child Behavior Checklist	CBCL
-0,486119498	SRS_SRS_MOT	Social Motivation Raw Score	Social Responsiveness Scale	SRS
-0,479143607	SRS_SRS_MOT_T	Social Motivation T-Score	Social Responsiveness Scale	SRS
-0,468959511	SRS_SRS_RRB_T	Restricted Interests and	Social Responsiveness Scale	SRS

Correlation coefficient (R)	Variable name in data	Item text	Instrument name	Instrument abbreviation (if applicable)
		Repetitive Behavior T-Score		
-0,468306342	SRS_SRS_DSMRRB_T	Restricted Interests and Repetitive Behavior T-Score	Social Responsiveness Scale	SRS
0,465156349	rh_rostralmiddlefrontal_area	Cortical area in rh rostralmiddlefrontal	Cortical Area	NA
-0,464181085	ICU_P_ICU_P_Total	Total Score	Inventory of Callous-Unemotional Traits Parent	ICU
-0,460411609	SRS_SRS_DSMRRB	Restricted Interests and Repetitive Behavior Raw Score	Social Responsiveness Scale	SRS
-0,460411609	SRS_SRS_RRB	Restricted Interests and Repetitive Behavior Raw Score	Social Responsiveness Scale	SRS
-0,455588324	CBCL_CBCL_Ext	Externalizing Raw Score	Child Behavior Checklist	CBCL
0,45470675	rh_lateralorbitofrontal_volume	Cortical volume in rh lateralorbitofrontal	Cortical Volume	NA
-0,453508286	CBCL_CBCL_Ext_T	Externalizing T Score	Child Behavior Checklist	CBCL
0,4522388	lh_rostralmiddlefrontal_area	Cortical area in lh rostralmiddlefrontal	Cortical Area	NA
0,446677006	lh_lateralorbitofrontal_volume	Cortical volume in lh lateralorbitofrontal	Cortical Volume	NA
-0,441893649	CBCL_CBCL_SP_T	Social Problems T Score	Child Behavior Checklist	CBCL
-0,44181994	SDQ_SDQ_Externalizing	Externalizing Score	Strength and Difficulties Questionnaire	SDQ
-0,439018611	SDQ_SDQ_Generating_Impact	Generating Impact Scores	Strength and Difficulties Questionnaire	SDQ
-0,4384675	CBCL_CBCL_AB	Aggressive Behavior Raw Score	Child Behavior Checklist	CBCL
0,433510693	rh_parsorbitalis_area	Cortical area in rh parsorbitalis	Cortical Area	NA
0,430673486	rh_rostralmiddlefrontal_volume	Cortical volume in rh rostralmiddlefrontal	Cortical Volume	NA

Correlation coefficient (R)	Variable name in data	Item text	Instrument name	Instrument abbreviation (if applicable)
0,427155017	rh_superiorfrontal_area	Cortical area in rh superiorfrontal	Cortical Area	NA
-0,426754638	SDQ_SDQ_Internalizing	Internalizing Score	Strength and Difficulties Questionnaire	SDQ
-0,424475234	CBCL_CBCL_AB_T	Aggressive Behavior T Score	Child Behavior Checklist	CBCL
-0,42410125	CBCL_CBCL_RBB	Rule Breaking Behavior Raw Score	Child Behavior Checklist	CBCL
-0,419257905	CBCL_CBCL_WD	Withdrawn/Depressed Raw Score	Child Behavior Checklist	CBCL
-0,418059236	CBCL_CBCL_SP	Social Problems Raw Score	Child Behavior Checklist	CBCL
-0,417394608	CBCL_CBCL_Int	Internalizing Raw Score	Child Behavior Checklist	CBCL
0,417305766	rh_medialorbitofrontal_area	Cortical area in rh medialorbitofrontal	Cortical Area	NA
0,413801324	lh_rostralmiddlefrontal_volume	Cortical volume in lh rostralmiddlefrontal	Cortical Volume	NA
0,413565573	rh_meanGI_RH_GI	Mean GI in right hemisphere	Local Gyrification Index	NA
0,413519459	rh_medialorbitofrontal_volume	Cortical volume in rh medialorbitofrontal	Cortical Volume	NA
0,411307252	lh_lateralorbitofrontal_area	Cortical area in lh lateralorbitofrontal	Cortical Area	NA
-0,410695183	SWAN_SWAN_IN	Inattention Average	The Strengths and Weaknesses Assessment of Normal Behavior	SWAN
-0,402709696	CBCL_CBCL_Int_T	Internalizing T Score	Child Behavior Checklist	CBCL
-0,402280864	SRS_SRS_51	51. Has difficulty answering questions directly and ends up talking around the subject.	Social Responsiveness Scale	SRS
-0,401080263	SRS_SRS_AWR_T	Social Awareness T-Score	Social Responsiveness Scale	SRS
0,399197985	lh_precuneus_GI	Gyrification of lh precuneus GI	Local Gyrification Index	NA
0,3968959	lh_meanGI_LH_GI	Mean GI in left hemisphere	Local Gyrification Index	NA
-0,396294518	CBCL_CBCL_WD_T	Withdrawn/Depressed T Score	Child Behavior Checklist	CBCL
0,396012422	rh_superiorfrontal_GI	Gyrification of rh superiorfrontal GI	Local Gyrification Index	NA

Correlation coefficient (R)	Variable name in data	Item text	Instrument name	Instrument abbreviation (if applicable)
-0,395626717	SRS_SRS_AWR	Social Awareness Raw Score	Social Responsiveness Scale	SRS
-0,39386523	SWAN_SWAN_06	6. Engages in tasks that require sustained mental effort	The Strengths and Weaknesses Assessment of Normal Behavior	SWAN
-0,393091077	SDQ_SDQ_Conduct_Problems	Conduct problems scale	Strength and Difficulties Questionnaire	SDQ
-0,390587233	SRS_SRS_62	62. Give unusual or illogical reasons for doing things.	Social Responsiveness Scale	SRS
-0,389287939	SRS_SRS_44	44. Doesn't understand how events relate to one another (cause and effect) the way other children his or her age do.	Social Responsiveness Scale	SRS
-0,389012075	SCQ_SCQ_Total	Total Score	Social Communication Questionnaire	SCQ
0,387407205	rh_rostralmiddlefrontal_GI	Gyrification of rh rostralmiddlefrontal GI	Local Gyrification Index	NA
0,38729368	lh_parsorbitalis_area	Cortical area in lh parsorbitalis	Cortical Area	NA
0,38530072	rh_lateralorbitofrontal_area	Cortical area in rh lateralorbitofrontal	Cortical Area	NA
0,383637008	lh_precentral_GI	Gyrification of lh precentral GI	Local Gyrification Index	NA
0,382465671	rh_middletemporal_area	Cortical area in rh middletemporal	Cortical Area	NA
-0,381780273	SRS_SRS_58	58. Concentrates too much on parts of things rather than seeing the whole picture. For example, if asked to describe what happened in a story, he or she may talk only about the kind of clothes the characters were wearing.	Social Responsiveness Scale	SRS
0,381696147	rh_precuneus_GI	Gyrification of rh precuneus GI	Local Gyrification Index	NA
0,377392169	rh_rostralanteriorcingulate_GI	Gyrification of rh rostralanteriorcingulate GI	Local Gyrification Index	NA

Correlation coefficient (R)	Variable name in data	Item text	Instrument name	Instrument abbreviation (if applicable)
-0,376083568	CBCL_CBCL_61	61. Poor school work	Child Behavior Checklist	CBCL
-0,375908168	SWAN_SWAN_Total	SWAN Average	The Strengths and Weaknesses Assessment of Normal Behavior	SWAN
-0,374583596	ICU_P_ICU_P_Uncaring	Uncaring Subscale Score	Inventory of Callous-Unemotional Traits Parent	ICU
0,373148707	lh_lateraloccipital_area	Cortical area in lh lateraloccipital	Cortical Area	NA
0,373047431	rh_parsorbitalis_volume	Cortical volume in rh parsorbitalis	Cortical Volume	NA
0,372622847	lh_parstriangularis_area	Cortical area in lh parstriangularis	Cortical Area	NA
0,372609119	rh_middletemporal_volume	Cortical volume in rh middletemporal	Cortical Volume	NA
-0,372603499	SDQ_SDQ_Peer_Problems	Peer Problems Scale	Strength and Difficulties Questionnaire	SDQ
-0,370046399	SDQ_SDQ_Hyperactivity	Hyperactivity Scale	Strength and Difficulties Questionnaire	SDQ
0,368397687	WIAT_WIAT_LC_Stnd	Listening Comprehension Standard Score	Wechsler Individual Achievement Test	WIAT
0,368220761	WIAT_WIAT_LC_P	Listening Comprehension Percentile Rank	Wechsler Individual Achievement Test	WIAT
0,36761654	rh_rostralanteriorcingulate_area	Cortical area in rh rostralanteriorcingulate	Cortical Area	NA
0,365986666	lh_superiorfrontal_area	Cortical area in lh superiorfrontal	Cortical Area	NA
0,365595021	WIAT_WIAT_MP_P	Math Problem Solving Percentile Rank	Wechsler Individual Achievement Test	WIAT
0,365245854	rh_parsopercularis_GI	Gyrification of rh parsopercularis GI	Local Gyrification Index	NA
-0,36459649	SDQ_SDQ_26	Overall, do you think that your child has difficulties in one or more of the following areas: emotions, concentration, behavior or being able to get on	Strength and Difficulties Questionnaire	SDQ

Correlation coefficient (R)	Variable name in data	Item text	Instrument name	Instrument abbreviation (if applicable)
		with other people?		
0,364285078	rh_fusiform_volume	Cortical volume in rh fusiform	Cortical Volume	NA
0,363009283	lh_rostralanteriorcingulate_volume	Cortical volume in lh rostralanteriorcingulate	Cortical Volume	NA
0,363009133	rh_fusiform_area	Cortical area in rh fusiform	Cortical Area	NA
-0,361933801	SRS_SRS_10	10. Takes things too literally and doesn't get the real meaning of a conversation.	Social Responsiveness Scale	SRS
0,3608365	lh_fusiform_area	Cortical area in lh fusiform	Cortical Area	NA
0,360605377	lh_superiortemporal_area	Cortical area in lh superiortemporal	Cortical Area	NA
0,360494137	rh_superiorfrontal_volume	Cortical volume in rh superiorfrontal	Cortical Volume	NA
-0,360409221	SWAN_SWAN_04	4. Follows through on instructions and finishes school work and chores	The Strengths and Weaknesses Assessment of Normal Behavior	SWAN
0,359917335	WIAT_WIAT_MP_Stnd	Math Problem Solving Standard Score	Wechsler Individual Achievement Test	WIAT
0,359893892	rh_precentral_GI	Gyrification of rh precentral GI	Local Gyrification Index	NA
0,359807022	rh_parstriangularis_area	Cortical area in rh parstriangularis	Cortical Area	NA
-0,359756992	SRS_SRS_22	22. Plays appropriately with children his or her age.	Social Responsiveness Scale	SRS
0,358691391	lh_parsorbitalis_volume	Cortical volume in lh parsorbitalis	Cortical Volume	NA
0,355060736	lh_insula_volume	Cortical volume in lh insula	Cortical Volume	NA
-0,354733639	ICU_P_ICU_P_Unemotional	Unemotional Subscale Score	Inventory of Callous-Unemotional Traits Parent	ICU
0,354249595	lh_superiorfrontal_GI	Gyrification of lh superiorfrontal GI	Local Gyrification Index	NA
-0,353805989	ARI_P_ARI_P_Total_Score	Total Score	Affective Reactivity Index	ARI
-0,351424346	CBCL_CBCL_TP_T	Thought Problems T Score	Child Behavior Checklist	CBCL

Correlation coefficient (R)	Variable name in data	Item text	Instrument name	Instrument abbreviation (if applicable)
-0,350327042	CBCL_CBCL_04	4. Fails to finish things he/she starts	Child Behavior Checklist	CBCL
0,35013909	rh_rostralanteriorcingulate_volume	Cortical volume in rh rostralanteriorcingulate	Cortical Volume	NA
-0,350028519	CBCL_CBCL_41	41. Impulsive or acts without thinking	Child Behavior Checklist	CBCL
0,349927948	rh_frontalpole_area	Cortical area in rh frontalpole	Cortical Area	NA
0,349257009	rh_lateraloccipital_area	Cortical area in rh lateraloccipital	Cortical Area	NA
0,349048328	lh_rostralmiddlefrontal_GI	Gyrification of lh rostralmiddlefrontal GI	Local Gyrification Index	NA
0,348782941	rh_caudalanteriorcingulate_GI	Gyrification of rh caudalanteriorcingulate GI	Local Gyrification Index	NA
0,348482441	rh_inferiortemporal_area	Cortical area in rh inferiortemporal	Cortical Area	NA
0,348454831	rh_posteriorcingulate_GI	Gyrification of rh posteriorcingulate GI	Local Gyrification Index	NA
-0,34809309	SRS_SRS_36	36. Has difficulty relating to adults.	Social Responsiveness Scale	SRS
0,347857925	lh_superiortemporal_GI	Gyrification of lh superiortemporal GI	Local Gyrification Index	NA
-0,347110787	CBCL_CBCL_78	78. Inattentive or easily distracted	Child Behavior Checklist	CBCL
0,346789186	lh_cuneus_GI	Gyrification of lh cuneus GI	Local Gyrification Index	NA
0,345897395	lh_rostralanteriorcingulate_area	Cortical area in lh rostralanteriorcingulate	Cortical Area	NA
0,345567147	lh_isthmuscingulate_GI	Gyrification of lh isthmuscingulate GI	Local Gyrification Index	NA
-0,345511748	CBCL_CBCL_08	8. Can't concentrate, can't pay attention for long	Child Behavior Checklist	CBCL
-0,345510542	SympChck_CSC_40C	Actively disobeys or doesn't listen to adult rules (current)	Symptom Checklist Parent report	SympChck-P
-0,345232708	SRS_SRS_35	35. Has trouble keeping up with the flow of a normal conversation.	Social Responsiveness Scale	SRS

Correlation coefficient (R)	Variable name in data	Item text	Instrument name	Instrument abbreviation (if applicable)
0,344811648	lh_parsoperculari s_GI	Gyrification of lh parsopercularis GI	Local Gyrification Index	NA
-0,344598986	SRS_SRS_19	19. Gets frustrated trying to get ideas across in conversations.	Social Responsiveness Scale	SRS
0,344372312	rh_paracentral_G I	Gyrification of rh paracentral GI	Local Gyrification Index	NA
-0,344351746	SWAN_SWAN_0 3	3. Listens when spoken to directly	The Strengths and Weaknesses Assessment of Normal Behavior	SWAN
-0,343983917	CBCL_CBCL_86	86. Stubborn, sullen, or irritable	Child Behavior Checklist	CBCL
0,342589377	rh_superiortempo ral_area	Cortical area in rh superiortemporal	Cortical Area	NA
0,342445928	rh_medialorbitofr ontal_GI	Gyrification of rh medialorbitofront al GI	Local Gyrification Index	NA
0,341705476	lh_transversetem poral_GI	Gyrification of lh transversetempor al GI	Local Gyrification Index	NA
0,34113855	lh_pericalcarine_ GI	Gyrification of lh pericalcarine GI	Local Gyrification Index	NA
0,340940028	lh_cuneus_area	Cortical area in lh cuneus	Cortical Area	NA
0,34045801	WIAT_WIAT_Nu m_P	Numerical Operations Percentile Rank	Wechsler Individual Achievement Test	WIAT
-0,339908765	SRS_SRS_48	48. Has a sense of humor, understands jokes.	Social Responsiveness Scale	SRS
0,339153975	lh_rostralanterior cingulate_GI	Gyrification of lh rostralanteriorcin gulate GI	Local Gyrification Index	NA
0,339073225	WIAT_WIAT_LC ODC_Stnd	Listening Comprehension Oral Discourse Comprehension Standard Score	Wechsler Individual Achievement Test	WIAT
-0,338481245	SDQ_SDQ_21	Thinks things out before acting	Strength and Difficulties Questionnaire	SDQ
0,338064616	rh_transversetem poral_GI	Gyrification of rh transversetempor al GI	Local Gyrification Index	NA
0,338056326	lh_fusiform_volu me	Cortical volume in lh fusiform	Cortical Volume	NA
0,337925276	lh_lingual_GI	Gyrification of lh lingual GI	Local Gyrification Index	NA
0,337658689	WIAT_WIAT_LC ODC_P	Listening Comprehension	Wechsler Individual	WIAT

Correlation coefficient (R)	Variable name in data	Item text	Instrument name	Instrument abbreviation (if applicable)
		Oral Discourse Comprehension Percentile Rank	Achievement Test	
0,337569731	lh_middletemporal_area	Cortical area in lh middletemporal	Cortical Area	NA
-0,337305947	SWAN_SWAN_02	2. Sustains attention on tasks or play activities	The Strengths and Weaknesses Assessment of Normal Behavior	SWAN
0,337262677	rh_isthmuscingulate_GI	Gyrification of rh isthmuscingulate GI	Local Gyrification Index	NA
-0,337203618	SRS_SRS_12	12. Is able to communicate his or her feelings to others.	Social Responsiveness Scale	SRS
0,336309149	lh_superiortemporal_volume	Cortical volume in lh superiortemporal	Cortical Volume	NA
-0,335855789	CBCL_CBCL_28	28. Breaks rules at home, school, or elsewhere	Child Behavior Checklist	CBCL
0,335133811	lh_medialorbitofrontal_area	Cortical area in lh medialorbitofrontal	Cortical Area	NA
0,334903851	lh_parstriangularis_volume	Cortical volume in lh parstriangularis	Cortical Volume	NA
0,334694276	lh_posteriorcingulate_GI	Gyrification of lh posteriorcingulate GI	Local Gyrification Index	NA
0,334483898	rh_parstriangularis_GI	Gyrification of rh parstriangularis GI	Local Gyrification Index	NA
-0,334471633	SympChck_CSC_52C	Has a hard time expressing feelings on his/her face during social interactions (current)	Symptom Checklist Parent report	SympChck-P
-0,334422573	SWAN_SWAN_05	5. Organizes tasks and activities	The Strengths and Weaknesses Assessment of Normal Behavior	SWAN
-0,334239813	CBCL_CBCL_TP	Thought Problems Raw Score	Child Behavior Checklist	CBCL
-0,334005416	SRS_SRS_11	11. Has good self-confidence.	Social Responsiveness Scale	SRS
0,333280232	rh_superiortemporal_GI	Gyrification of rh superiortemporal GI	Local Gyrification Index	NA

Correlation coefficient (R)	Variable name in data	Item text	Instrument name	Instrument abbreviation (if applicable)
-0,331301608	SympChck_CSC_52P	Has a hard time expressing feelings on his/her face during social interactions (past	Symptom Checklist Parent report	SympChck-P
-0,330754545	SDQ_SDQ_25	Good attention span, sees chores or homework through to the end	Strength and Difficulties Questionnaire	SDQ
-0,330373436	SRS_SRS_39	39. Has an unusally narrow range of interests.	Social Responsiveness Scale	SRS
-0,330217902	SRS_SRS_49	49. Does extremely well at a few tasks, but does not do as well at most other tasks.	Social Responsiveness Scale	SRS
-0,328932833	ICU_P_ICU_P_01	1. Expresses his/her feelings openly.	Inventory of Callous-Unemotional Traits Parent	ICU
-0,327885177	SRS_SRS_13	13. Is awkward is turn-taking interactions with peers (e.g., doesn't seem to understand the give-and-take of conversations)	Social Responsiveness Scale	SRS
0,327747765	lh_lingual_area	Cortical area in lh lingual	Cortical Area	NA
0,327622534	rh_superiortemporal_volume	Cortical volume in rh superiortemporal	Cortical Volume	NA
-0,327194558	SRS_SRS_37	37. Has difficulty relating to peers.	Social Responsiveness Scale	SRS
-0,327096837	SWAN_SWAN_01	1. Gives close attention to detail and avoids careless mistakes	The Strengths and Weaknesses Assessment of Normal Behavior	SWAN
0,327013294	lh_paracentral_GI	Gyrification of lh paracentral GI	Local Gyrification Index	NA
0,32642564	rh_parstriangularis_volume	Cortical volume in rh parstriangularis	Cortical Volume	NA
-0,324953822	SDQ_SDQ_15	Easily distracted, concentration wanders	Strength and Difficulties Questionnaire	SDQ

Correlation coefficient (R)	Variable name in data	Item text	Instrument name	Instrument abbreviation (if applicable)
-0,32466399	SDQ_SDQ_07	Generally well behaved, usually does what adults request	Strength and Difficulties Questionnaire	SDQ
0,323399631	lh_postcentral_GI	Gyrification of lh postcentral GI	Local Gyrification Index	NA
-0,322473766	PreInt_EduHx_recent_grades	Recent typical academic performance:	Interview-Education and Social History	Edu/Soc.Hist.
0,322198676	WIAT_WIAT_Num_Stnd	Numerical Operations Standard Score	Wechsler Individual Achievement Test	WIAT
-0,321642686	CBCL_CBCL_03	3. Argues a lot	Child Behavior Checklist	CBCL
-0,320804759	SDQ_SDQ_Emotional_Problems	Emotional Problems Scale	Strength and Difficulties Questionnaire	SDQ
0,320668689	rh_inferiortemporal_volume	Cortical volume in rh inferiortemporal	Cortical Volume	NA
0,320453113	rh_postcentral_GI	Gyrification of rh postcentral GI	Local Gyrification Index	NA
-0,320439337	SRS_SRS_60	60. Is emotionally distant, doesn't show his or her feelings.	Social Responsiveness Scale	SRS
0,320424145	rh_insula_volume	Cortical volume in rh insula	Cortical Volume	NA
0,319969605	lh_inferiortemporal_area	Cortical area in lh inferiortemporal	Cortical Area	NA
0,319502532	rh_lingual_area	Cortical area in rh lingual	Cortical Area	NA
0,319123816	lh_lateraloccipital_volume	Cortical volume in lh lateraloccipital	Cortical Volume	NA
0,318775347	rh_lingual_GI	Gyrification of rh lingual GI	Local Gyrification Index	NA
0,318638399	SDQ_SDQ_Prosocial	Prosocial Scale	Strength and Difficulties Questionnaire	SDQ
0,318215458	lh_insula_GI	Gyrification of lh insula GI	Local Gyrification Index	NA
-0,31774495	CBCL_CBCL_AD	Anxious/Depressed Raw Score	Child Behavior Checklist	CBCL
-0,317738644	SRS_SRS_33	33. Is socially awkward, even when he or she is trying to be polite.	Social Responsiveness Scale	SRS
-0,317052781	SRS_SRS_64	64. Is too tense in social settings.	Social Responsiveness Scale	SRS
-0,316686049	SRS_SRS_30	30. Becomes upset in a situation with lots	Social Responsiveness Scale	SRS

Correlation coefficient (R)	Variable name in data	Item text	Instrument name	Instrument abbreviation (if applicable)
		of things going on.		
0,316264484	lh_middletemporal_volume	Cortical volume in lh middletemporal	Cortical Volume	NA
0,31605089	lh_inferiortemporal_volume	Cortical volume in lh inferiortemporal	Cortical Volume	NA
-0,315538995	SRS_SRS_61	61. Is inflexible, has a hard time changing his or her mind.	Social Responsiveness Scale	SRS
0,314194234	lh_caudalmiddlefrontal_GI	Gyrification of lh caudalmiddlefrontal GI	Local Gyrification Index	NA
0,314137866	rh_caudalmiddlefrontal_GI	Gyrification of rh caudalmiddlefrontal GI	Local Gyrification Index	NA
-0,314032156	CBCL_CBCL_OP	Other Problems Raw Score	Child Behavior Checklist	CBCL
-0,313704833	CBCL_CBCL_22	22. Disobedient at home	Child Behavior Checklist	CBCL
0,313681036	WIAT_WIAT_Word_P	Word Reading Percentile Rank	Wechsler Individual Achievement Test	WIAT
-0,313481565	ICU_P_ICU_P_02	2. Does not seem to know “right” from “wrong”.	Inventory of Callous-Unemotional Traits Parent	ICU
-0,313214677	SympChck_CSC_41C	Frequently lies (current)	Symptom Checklist Parent report	SympChck-P
-0,31313378	CBCL_CBCL_01	1. Acts too young for his/her age	Child Behavior Checklist	CBCL
0,312622831	rh_insula_GI	Gyrification of rh insula GI	Local Gyrification Index	NA
0,312610157	rh_pericalcarine_GI	Gyrification of rh pericalcarine GI	Local Gyrification Index	NA
-0,312353176	SWAN_SWAN_09	9. Remembers daily activities	The Strengths and Weaknesses Assessment of Normal Behavior	SWAN
0,311776585	rh_parsopercularis_area	Cortical area in rh parsopercularis	Cortical Area	NA
-0,311429598	CBCL_CBCL_AD_T	Anxious/Depressed T Score	Child Behavior Checklist	CBCL
-0,310225437	ARI_P_ARI_P_01	Is easily annoyed by others	Affective Reactivity Index	ARI
-0,310184757	SRS_SRS_23	23. Does not join group activities unless told to do so.	Social Responsiveness Scale	SRS
0,30975569	lh_parsopercularis_area	Cortical area in lh parsopercularis	Cortical Area	NA

Correlation coefficient (R)	Variable name in data	Item text	Instrument name	Instrument abbreviation (if applicable)
0,309457965	rh_cuneus_GI	Gyrification of rh cuneus GI	Local Gyrification Index	NA
-0,309442008	CBCL_CBCL_26	26. Doesn't seem to feel guilty after misbehaving	Child Behavior Checklist	CBCL
0,309142756	WIAT_WIAT_LCRV_Std	Listening Comprehension Receptive Vocabulary Standard Score	Wechsler Individual Achievement Test	WIAT
0,308263416	lh_caudalanteriorcingulate_GI	Gyrification of lh caudalanteriorcingulate GI	Local Gyrification Index	NA
-0,307377358	SRS_SRS_08	8. Behaves in ways that seem strange or bizarre.	Social Responsiveness Scale	SRS
-0,3073005	SRS_SRS_24	24. Has more difficulty than other children with changes in his or her routine.	Social Responsiveness Scale	SRS
-0,307263989	SDQ_SDQ_16	Nervous in new situations, easily loses confidence	Strength and Difficulties Questionnaire	SDQ
0,305956487	WIAT_WIAT_LCRV_P	Listening Comprehension Receptive Vocabulary Percentile Rank	Wechsler Individual Achievement Test	WIAT
0,305475485	lh_fusiform_GI	Gyrification of lh fusiform GI	Local Gyrification Index	NA
0,30543971	rh_posteriorcingulate_volume	Cortical volume in rh posteriorcingulate	Cortical Volume	NA
-0,305169536	SympChck_CSC_39C	Argues or talks back to adults, more than others his/her age (current)	Symptom Checklist Parent report	SympChck-P
-0,304993426	SWAN_SWAN_07	7. Keeps track of things necessary for activities (doesn't lose them)	The Strengths and Weaknesses Assessment of Normal Behavior	SWAN
-0,304612087	ARI_P_ARI_P_02	Often loses his/her temper	Affective Reactivity Index	ARI
0,304265238	lh_superiorfrontal_volume	Cortical volume in lh superiorfrontal	Cortical Volume	NA
-0,304137182	SympChck_CSC_34C	Has trouble paying attention, and it affects school work or performance (current)	Symptom Checklist Parent report	SympChck-P

Correlation coefficient (R)	Variable name in data	Item text	Instrument name	Instrument abbreviation (if applicable)
-0,303884626	CBCL_CBCL_95	95. Temper tantrums or hot temper	Child Behavior Checklist	CBCL
0,303497896	WIAT_WIAT_Word_Stnd	Word Reading Standard Score	Wechsler Individual Achievement Test	WIAT
0,302694957	rh_caudalanteriorcingulate_area	Cortical area in rh caudalanteriorcingulate	Cortical Area	NA
0,302475035	rh_lateraloccipital_volume	Cortical volume in rh lateraloccipital	Cortical Volume	NA
0,302295141	rh_posteriorcingulate_area	Cortical area in rh posteriorcingulate	Cortical Area	NA
-0,302271155	ARI_P_ARI_P_04	Is angry most of the time	Affective Reactivity Index	ARI
-0,301752629	SRS_SRS_29	29. Is regarded by other children as odd or weird.	Social Responsiveness Scale	SRS
0,301311044	WIAT_WIAT_Spell_P	Spelling Percentile Rank	Wechsler Individual Achievement Test	WIAT
-0,301149794	SRS_SRS_31	31. Can't get his or her mind off something once he or she starts thinking about it.	Social Responsiveness Scale	SRS
-0,301148298	PreInt_EduHx_getalongkids_school	How does your child get along with other children at school?	Interview-Education and Social History	Edu/Soc.Hist.
0,300959182	lh_inferiorparietal_GI	Gyrification of lh inferiorparietal GI	Local Gyrification Index	NA
0,300468306	rh_cuneus_area	Cortical area in rh cuneus	Cortical Area	NA
-0,299231976	SympChck_CSC_02C	Often feels annoyed and irritated (current)	Symptom Checklist Parent report	SympChck-P
0,297762618	lh_medialorbitofrontal_volume	Cortical volume in lh medialorbitofrontal	Cortical Volume	NA
0,29768626	rh_lateralorbitofrontal_GI	Gyrification of rh lateralorbitofrontal GI	Local Gyrification Index	NA
-0,297407443	CBCL_CBCL_13	13. Confused or seems to be in a fog	Child Behavior Checklist	CBCL
-0,296017499	ICU_P_ICU_P_15	15. Always tries his/her best.	Inventory of Callous-Unemotional Traits Parent	ICU

Correlation coefficient (R)	Variable name in data	Item text	Instrument name	Instrument abbreviation (if applicable)
-0,295490681	ARI_P_ARI_P_06	Loses temper easily	Affective Reactivity Index	ARI
0,295007011	WIAT_WIAT_Spell_Stnd	Spelling Standard Score	Wechsler Individual Achievement Test	WIAT
-0,294859308	SympChck_CSC_37C	Often acts before thinking (current)	Symptom Checklist Parent report	SympChck-P
-0,292411737	ICU_P_ICU_P_20	20. Does not like to put the time into doing things well.	Inventory of Callous-Unemotional Traits Parent	ICU
-0,292331994	ASSQ_ASSQ_18	lacks common sense	Autism Spectrum Screening Questionnaire	ASSQ
0,292283656	lh_medialorbitofrontal_GI	Gyrification of lh medialorbitofrontal GI	Local Gyrification Index	NA
-0,292096453	SympChck_CSC_38C	Often becomes really upset and loses his/her temper (current)	Symptom Checklist Parent report	SympChck-P
0,291888299	lh_parstriangularis_GI	Gyrification of lh parstriangularis GI	Local Gyrification Index	NA
-0,291672435	ICU_P_ICU_P_08	8. Is concerned about the feelings of others.	Inventory of Callous-Unemotional Traits Parent	ICU
-0,291065242	ASSQ_ASSQ_13	makes naïve and embarrassing remarks	Autism Spectrum Screening Questionnaire	ASSQ
0,289520224	lh_parsopercularis_volume	Cortical volume in lh parsopercularis	Cortical Volume	NA
0,289046468	rh_caudalanteriorcingulate_volume	Cortical volume in rh caudalanteriorcingulate	Cortical Volume	NA
0,288688886	rh_frontalpole_GI	Gyrification of rh frontalpole GI	Local Gyrification Index	NA
-0,288080757	SympChck_CSC_34P	Has trouble paying attention, and it affects school work or performance (past	Symptom Checklist Parent report	SympChck-P
-0,288044469	ICU_P_ICU_P_17	17. Tries not to hurt others' feelings.	Inventory of Callous-Unemotional Traits Parent	ICU
-0,287953946	CBCL_CBCL_05	5. There is very little he/she enjoys	Child Behavior Checklist	CBCL

Correlation coefficient (R)	Variable name in data	Item text	Instrument name	Instrument abbreviation (if applicable)
0,287844752	lh_bankssts_GI	Gyrification of lh bankssts GI	Local Gyrification Index	NA
-0,287497312	SRS_SRS_59	59. Is overly suspicious.	Social Responsiveness Scale	SRS
0,286903171	CTOPP_CTOPP_EL_P	Elision percentile score	Comprehensive Test of Phonological Processing	CTOPP-2
0,286745272	lh_lateralorbitofrontal_GI	Gyrification of lh lateralorbitofrontal GI	Local Gyrification Index	NA
0,286626266	CTOPP_CTOPP_EL_S	Elision scaled score	Comprehensive Test of Phonological Processing	CTOPP-2
0,28658329	rh_lingual_volume	Cortical volume in rh lingual	Cortical Volume	NA
-0,286387288	PCIAT_PCIAT_02	2. How often does your child neglect household chores to spend more time online?	Parent-Child Internet Addiction Test	PCIAT
-0,286089083	SympChck_CSC_35C	Is often easily distracted (current)	Symptom Checklist Parent report	SympChck-P
0,285783915	rh_inferiorparietal_GI	Gyrification of rh inferiorparietal GI	Local Gyrification Index	NA
-0,285690162	SympChck_CSC_37P	Often acts before thinking (past	Symptom Checklist Parent report	SympChck-P
-0,285268968	SDQ_SDQ_05	Often loses temper	Strength and Difficulties Questionnaire	SDQ
-0,284120623	SympChck_CSC_40P	Actively disobeys or doesn't listen to adult rules (past	Symptom Checklist Parent report	SympChck-P
-0,283599978	SWAN_SWAN_18	18. Enters into conversation and games without interrupting or intruding	The Strengths and Weaknesses Assessment of Normal Behavior	SWAN
-0,28316766	ICU_P_ICU_P_14	14. It is easy to tell how he/she is feeling.	Inventory of Callous-Unemotional Traits Parent	ICU
-0,283088676	SRS_SRS_04	4. When under stress, he or she shows rigid or inflexible patterns of behavior that seem odd.	Social Responsiveness Scale	SRS
-0,282676289	SWAN_SWAN_16	16. Reflects on questions	The Strengths and Weaknesses	SWAN

Correlation coefficient (R)	Variable name in data	Item text	Instrument name	Instrument abbreviation (if applicable)
		(controls blurting out answers)	Assessment of Normal Behavior	
-0,282544436	SRS_SRS_47	47. Is too silly or laughs inappropriately.	Social Responsiveness Scale	SRS
-0,28245634	SDQ_SDQ_14	Generally liked by other children (for 11-17 year olds: Generally liked by other youth)	Strength and Difficulties Questionnaire	SDQ
-0,282298005	SWAN_SWAN_HY	Hyperactivity Average	The Strengths and Weaknesses Assessment of Normal Behavior	SWAN
-0,282267977	CBCL_CBCL_09	9. Can't get his/her mind off certain thoughts; obsessions	Child Behavior Checklist	CBCL
0,282011108	rh_fusiform_GI	Gyrification of rh fusiform GI	Local Gyrification Index	NA
0,281718241	lh_lingual_volume	Cortical volume in lh lingual	Cortical Volume	NA
-0,281407394	CBCL_CBCL_43	43. Lying or cheating	Child Behavior Checklist	CBCL
0,281357535	lh_cuneus_volume	Cortical volume in lh cuneus	Cortical Volume	NA
-0,281288575	SRS_SRS_16	16. Avoids eye contact or has unusual eye contact.	Social Responsiveness Scale	SRS
-0,281087082	SympChck_CSC_35P	Is often easily distracted (past	Symptom Checklist Parent report	SympChck-P
-0,281051417	PCIAT_PCIAT_Total	Total Score	Parent-Child Internet Addiction Test	PCIAT
0,280193901	lh_pericalcarine_area	Cortical area in lh pericalcarine	Cortical Area	NA
-0,278963642	CBCL_CBCL_33	33. Feels or complains that no one loves him/her	Child Behavior Checklist	CBCL
-0,278851557	ICU_P_ICU_P_21	21. The feelings of others are unimportant to him/her.	Inventory of Callous-Unemotional Traits Parent	ICU
0,278790283	lh_supramarginal_GI	Gyrification of lh supramarginal GI	Local Gyrification Index	NA
0,27794995	lh_superiorparietal_GI	Gyrification of lh superiorparietal GI	Local Gyrification Index	NA
0,277594602	rh_cuneus_volume	Cortical volume in rh cuneus	Cortical Volume	NA
0,277244395	lh_insula_area	Cortical area in lh insula	Cortical Area	NA

Correlation coefficient (R)	Variable name in data	Item text	Instrument name	Instrument abbreviation (if applicable)
-0,277154277	ICU_P_ICU_P_12	12. Seems very cold and uncaring.	Inventory of Callous-Unemotional Traits Parent	ICU
0,27648932	WIAT_WIAT_Pseudo_P	Pseudo-word Decoding Percentile Rank	Wechsler Individual Achievement Test	WIAT
-0,276364886	ICU_P_ICU_P_23	23. Works hard on everything.	Inventory of Callous-Unemotional Traits Parent	ICU
-0,276300849	SympChck_CSC_21C	Is unable to speak in specific situations, such as school, despite being able to speak without a problem in other situations (current)	Symptom Checklist Parent report	SympChck-P
-0,275972919	CBCL_CBCL_25	25. Doesn't get along well with other kids	Child Behavior Checklist	CBCL
-0,275662902	SympChck_CSC_51P	Often has a difficult time making eye contact (past	Symptom Checklist Parent report	SympChck-P
-0,275063443	SRS_SRS_34	34. Avoids people who want to be emotionally close to him or her.	Social Responsiveness Scale	SRS
0,274787269	lh_parahippocampal_GI	Gyrification of lh parahippocampal GI	Local Gyrification Index	NA
-0,274367734	SCARED_P_SCARED_P_Total	Total Score	Screen for Child Anxiety Related Disorders	SCARED
-0,273294745	SDQ_SDQ_18	Often lies or cheats	Strength and Difficulties Questionnaire	SDQ
-0,273188805	SympChck_CSC_21P	Is unable to speak in specific situations, such as school, despite being able to speak without a problem in other situations (past	Symptom Checklist Parent report	SympChck-P
0,272806176	rh_parsopercularis_volume	Cortical volume in rh parsopercularis	Cortical Volume	NA
-0,272772137	SympChck_CSC_02P	Often feels annoyed and irritated (past	Symptom Checklist Parent report	SympChck-P

Correlation coefficient (R)	Variable name in data	Item text	Instrument name	Instrument abbreviation (if applicable)
-0,272112566	SRS_SRS_40	40. Is imaginative, good at pretending (without losing touch with reality).	Social Responsiveness Scale	SRS
-0,270821879	ICU_P_ICU_P_16	16. Apologizes ("says he/she is sorry") to persons he/she has hurt.	Inventory of Callous-Unemotional Traits Parent	ICU
-0,270683442	CBCL_CBCL_103	103. Unhappy, sad, or depressed	Child Behavior Checklist	CBCL
-0,270542439	SWAN_SWAN_08	8. Ignores extraneous stimuli	The Strengths and Weaknesses Assessment of Normal Behavior	SWAN
0,270277619	rh_superiorparietal_GI	Gyrification of rh superiorparietal GI	Local Gyrification Index	NA
-0,269864943	SRS_SRS_28	28. Thinks or talks about the same thing over and over.	Social Responsiveness Scale	SRS
0,269608279	WIAT_WIAT_Pseudo_Stnd	Pseudo-word Decoding Standard Score	Wechsler Individual Achievement Test	WIAT
0,268656466	rh_pericalcarine_area	Cortical area in rh pericalcarine	Cortical Area	NA
-0,26822329	SympChck_CSC_51C	Often has a difficult time making eye contact (current)	Symptom Checklist Parent report	SympChck-P
-0,267598096	ICU_P_ICU_P_11	11. Does not care about doing things well.	Inventory of Callous-Unemotional Traits Parent	ICU
0,266920022	lh_frontalpole_area	Cortical area in lh frontalpole	Cortical Area	NA
-0,266548936	SWAN_SWAN_17	17. Awaits turn (stands in line and takes turns)	The Strengths and Weaknesses Assessment of Normal Behavior	SWAN
-0,266438595	PCIAT_PCIAT_03	3. How often does your child prefer to spend time online rather than with the rest of your family?	Parent-Child Internet Addiction Test	PCIAT
-0,266073022	ICU_P_ICU_P_05	5. Feels bad or guilty when he/she has done something wrong.	Inventory of Callous-Unemotional Traits Parent	ICU

Correlation coefficient (R)	Variable name in data	Item text	Instrument name	Instrument abbreviation (if applicable)
0,265927668	SDQ_SDQ_01	Considerate of other people's feelings	Strength and Difficulties Questionnaire	SDQ
0,265816894	lh_middletemporal_GI	Gyrification of lh middletemporal GI	Local Gyrification Index	NA
-0,265793108	CBCL_CBCL_87	87. Sudden changes in mood or feelings	Child Behavior Checklist	CBCL
-0,265302052	SympChck_CSC_20C	Often feels very nervous when he/she has to do things in front of others (current)	Symptom Checklist Parent report	SympChck-P
0,264814967	rh_insula_area	Cortical area in rh insula	Cortical Area	NA
0,264511617	rh_bankssts_GI	Gyrification of rh bankssts GI	Local Gyrification Index	NA
-0,264168889	SRS_SRS_18	18. Has difficulty making friends, even when trying his or her best.	Social Responsiveness Scale	SRS
-0,263864156	CBCL_CBCL_23	23. Disobedient at school	Child Behavior Checklist	CBCL
-0,263421454	CBCL_CBCL_19	19. Demands a lot of attention	Child Behavior Checklist	CBCL
0,263022051	rh_transversetemporal_area	Cortical area in rh transversetemporal	Cortical Area	NA
-0,262668178	PreInt_Demos_Fam_P1_RelQuality	How is the quality of the relationship between you and the child?	Interview-Demographics/Family	Demog.Fam.
-0,261916323	SympChck_CSC_41P	Frequently lies (past	Symptom Checklist Parent report	SympChck-P
-0,261740743	SRS_SRS_01	1. Seems much more fidgety in social situations than when alone.	Social Responsiveness Scale	SRS
-0,261526246	CBCL_CBCL_80	80. Stares blankly	Child Behavior Checklist	CBCL
0,26127365	lh_frontalpole_GI	Gyrification of lh frontalpole GI	Local Gyrification Index	NA
-0,261247993	ICU_P_ICU_P_24	24. Does things to make others feel good.	Inventory of Callous-Unemotional Traits Parent	ICU
-0,261150005	SympChck_CSC_39P	Argues or talks back to adults, more than others his/her age (past	Symptom Checklist Parent report	SympChck-P
-0,260719981	PreInt_EduHx_IEP	Does your child have an Individualized	Interview-Education and Social History	Edu/Soc.Hist.

Correlation coefficient (R)	Variable name in data	Item text	Instrument name	Instrument abbreviation (if applicable)
		Education Plan (IEP)?		
-0,259955185	SCARED_P_SCARED_P_SC	Social Anxiety Disorder Score	Screen for Child Anxiety Related Disorders	SCARED
-0,259350145	CBCL_CBCL_68	68. Screams a lot	Child Behavior Checklist	CBCL
-0,258845364	CBCL_CBCL_71	71. Self-conscious or easily embarrassed	Child Behavior Checklist	CBCL
-0,258725517	SympChck_CSC_20P	Often feels very nervous when he/she has to do things in front of others (past	Symptom Checklist Parent report	SympChck-P
-0,256794915	SRS_SRS_03	3. Seems self-confident when interacting with others.	Social Responsiveness Scale	SRS
-0,256496702	CBCL_CBCL_10	10. Can't sit still, restless or hyperactive	Child Behavior Checklist	CBCL
-0,255306939	ASSQ_ASSQ_10	is surprisingly good at some things and surprisingly poor at others	Autism Spectrum Screening Questionnaire	ASSQ
-0,255268448	SympChck_CSC_50C	Is preoccupied with very specific objects, routines, or interests (current)	Symptom Checklist Parent report	SympChck-P
0,255021349	rh_middletemporal_GI	Gyrification of rh middletemporal GI	Local Gyrification Index	NA
-0,254997054	ARI_P_ARI_P_03	Stays angry for a long time	Affective Reactivity Index	ARI
-0,254938565	CBCL_CBCL_89	89. Suspicious	Child Behavior Checklist	CBCL
-0,2543447	SRS_SRS_57	57. Gets teased a lot.	Social Responsiveness Scale	SRS
0,254185991	rh_transversetemporal_volume	Cortical volume in rh transversetemporal	Cortical Volume	NA
0,254087431	lh_lateraloccipital_GI	Gyrification of lh lateraloccipital GI	Local Gyrification Index	NA
-0,254000167	SRS_SRS_27	27. Avoids starting social interactions with peers or adults.	Social Responsiveness Scale	SRS
0,253300312	lh_inferiorparietal_area	Cortical area in lh inferiorparietal	Cortical Area	NA

Correlation coefficient (R)	Variable name in data	Item text	Instrument name	Instrument abbreviation (if applicable)
-0,252746049	SympChck_CSC_38P	Often becomes really upset and loses his/her temper (past	Symptom Checklist Parent report	SympChck-P
0,252566843	lh_precuneus_area	Cortical area in lh precuneus	Cortical Area	NA
0,252479984	lh_posteriorcingulate_area	Cortical area in lh posteriorcingulate	Cortical Area	NA
-0,252276002	ICU_P_ICU_P_18	18. Shows no remorse when he/she has done something wrong.	Inventory of Callous-Unemotional Traits Parent	ICU
0,251517564	APQ_P_APQ_P_01	1. You have a friendly talk with your child	Alabama Parenting Questionnaire	APQ
-0,251458891	CBCL_CBCL_69	69. Secretive, keeps things to self	Child Behavior Checklist	CBCL
-0,251446347	ICU_P_ICU_P_22	22. Hides his/her feelings from others.	Inventory of Callous-Unemotional Traits Parent	ICU
-0,251033498	SRS_SRS_05	5. Doesn't recognize when others are trying to take advantage of him or her.	Social Responsiveness Scale	SRS
0,249940686	rh_supramarginal_GI	Gyrification of rh supramarginal GI	Local Gyrification Index	NA
-0,249761702	SDQ_SDQ_13	Often unhappy, depressed or tearful	Strength and Difficulties Questionnaire	SDQ
-0,248392712	ICU_P_ICU_P_04	4. Does not care who he/she hurts to get what he/she wants.	Inventory of Callous-Unemotional Traits Parent	ICU
0,248201409	rh_lateraloccipital_GI	Gyrification of rh lateraloccipital GI	Local Gyrification Index	NA
-0,248189968	CBCL_CBCL_88	88. Sulks a lot	Child Behavior Checklist	CBCL
-0,247929669	SRS_SRS_41	41. Wanders aimlessly from one activity to another.	Social Responsiveness Scale	SRS
-0,247855194	ASSQ_ASSQ_15	wishes to be sociable but fails to make relationships with peers	Autism Spectrum Screening Questionnaire	ASSQ
-0,246981523	ICU_P_ICU_P_09	9. Does not care if he/she is in trouble.	Inventory of Callous-Unemotional Traits Parent	ICU

Correlation coefficient (R)	Variable name in data	Item text	Instrument name	Instrument abbreviation (if applicable)
-0,246574034	SRS_SRS_09	9. Clings to adults, seems too dependent on them.	Social Responsiveness Scale	SRS
0,24579094	lh_posteriorcingulate_volume	Cortical volume in lh posteriorcingulate	Cortical Volume	NA
-0,245658346	ICU_P_ICU_P_13	13. Easily admits to being wrong.	Inventory of Callous-Unemotional Traits Parent	ICU
-0,245521737	CBCL_CBCL_65	65. Refuses to talk	Child Behavior Checklist	CBCL
-0,245461616	SympChck_CSC_50P	Is preoccupied with very specific objects, routines, or interests (past	Symptom Checklist Parent report	SympChck-P
-0,245260767	SRS_SRS_38	38. Responds appropriately to mood changes in others (e.g., when a friend's or playmate's mood changes from happy to sad).	Social Responsiveness Scale	SRS
0,244893816	APQ_P_APQ_P_INV	Involvement Score	Alabama Parenting Questionnaire	APQ
0,244733295	rh_parahippocampal_GI	Gyrification of rh parahippocampal GI	Local Gyrification Index	NA
-0,243876807	ASSQ_ASSQ_11	uses language freely but fails to make adjustments to fit social contexts or the needs of different listeners	Autism Spectrum Screening Questionnaire	ASSQ
-0,242479342	CBCL_CBCL_38	38. Gets teased a lot	Child Behavior Checklist	CBCL
-0,242240368	SRS_SRS_17	17. Recognizes when something is unfair.	Social Responsiveness Scale	SRS
-0,239493933	CBCL_CBCL_21	21. Destroys things belonging to his/her family or others	Child Behavior Checklist	CBCL
-0,239394326	CBCL_CBCL_54	54. Overtired without good reason	Child Behavior Checklist	CBCL
-0,239380468	SCQ_SCQ_04	4. Does she/he ever use socially inappropriate questions or statements? For example, does she/he ever	Social Communication Questionnaire	SCQ

Correlation coefficient (R)	Variable name in data	Item text	Instrument name	Instrument abbreviation (if applicable)
		regularly ask personal questions or make personal comments at awkward times?		
-0,238648565	SympChck_CSC_49C	Feels really upset when there is an unexpected change in his/her schedule (current)	Symptom Checklist Parent report	SympChck-P
-0,237918985	SRS_SRS_07	7. Is aware of what others are thinking or feeling.	Social Responsiveness Scale	SRS
-0,237408776	CBCL_CBCL_SC_T	Somatic Complaints T Score	Child Behavior Checklist	CBCL
0,237079531	rh_caudalmiddlefrontal_area	Cortical area in rh caudalmiddlefrontal	Cortical Area	NA
-0,237042635	SRS_SRS_65	65. Stares or gazes off into space.	Social Responsiveness Scale	SRS
-0,236816894	ICU_P_ICU_P_07	7. Does not care about being on time.	Inventory of Callous-Unemotional Traits Parent	ICU
-0,235783253	SRS_SRS_02	2. Expressions on his or her face don't match what he or she is saying.	Social Responsiveness Scale	SRS
-0,235282218	CBCL_CBCL_111	111. Withdrawn, doesn't get inolved with others	Child Behavior Checklist	CBCL
-0,234217891	SDQ_SDQ_19	Picked on or bullied by other children (for 11-17 year olds: Picked on or bullied by other youth)	Strength and Difficulties Questionnaire	SDQ
-0,233853474	SRS_SRS_15	15. Is able to understand the meaning of other people's tone of voice and facial expressions.	Social Responsiveness Scale	SRS
-0,23329399	SRS_SRS_26	26. Offers comfort to others when they are sad.	Social Responsiveness Scale	SRS
0,232935136	lh_frontalpole_volume	Cortical volume in lh frontalpole	Cortical Volume	NA

Correlation coefficient (R)	Variable name in data	Item text	Instrument name	Instrument abbreviation (if applicable)
-0,232466069	SympChck_CSC_05C	Has strong and explosive feelings of anger (current)	Symptom Checklist Parent report	SympChck-P
-0,23221	APQ_P_APQ_P_ID	Inconsistent Discipline Score	Alabama Parenting Questionnaire	APQ
-0,231644086	CBCL_CBCL_SC	Somatic Complaints Raw Score	Child Behavior Checklist	CBCL
-0,231582332	SWAN_SWAN_14	14. Settles down and rests (controls excessive talking)	The Strengths and Weaknesses Assessment of Normal Behavior	SWAN
-0,230844531	SympChck_CSC_04P	Often feels overly happy and silly, above and beyond a normal feeling (past	Symptom Checklist Parent report	SympChck-P
-0,229606292	PCIAT_PCIAT_08	8. How often does your child seem withdrawn from others since discovering the Internet?	Parent-Child Internet Addiction Test	PCIAT
-0,229017726	ASSQ_ASSQ_25	is bullied by other children	Autism Spectrum Screening Questionnaire	ASSQ
-0,228929136	SWAN_SWAN_10	10. Sits still (controls movement of hands or feet or controls squirming)	The Strengths and Weaknesses Assessment of Normal Behavior	SWAN
-0,228870663	SCARED_P_SCARED_P_03	3. My child doesn’t like to be with people he/she does't know well	Screen for Child Anxiety Related Disorders	SCARED
-0,228711225	SRS_SRS_14	14. Is not well coordinated.	Social Responsiveness Scale	SRS
-0,22829999	PCIAT_PCIAT_09	9. How often does your child become defensive or secretive when asked what he or she does online?	Parent-Child Internet Addiction Test	PCIAT
0,228229151	lh_caudalanteriorcingulate_area	Cortical area in lh caudalanteriorcingulate	Cortical Area	NA
-0,227883063	ARI_P_ARI_P_05	Gets angry frequently	Affective Reactivity Index	ARI
-0,227795616	ASSQ_ASSQ_12	lacks empathy (i.e. tends to see things only from his/her own	Autism Spectrum Screening Questionnaire	ASSQ

Correlation coefficient (R)	Variable name in data	Item text	Instrument name	Instrument abbreviation (if applicable)
		perspective, and has troubles seeing things from other's perspective)		
0,227645536	lh_isthmuscingulate_area	Cortical area in lh isthmuscingulate	Cortical Area	NA
0,226895524	CELF_CELF_ExceedCutoff	Meets criterion score?	Clinical Evaluation of Language Fundamentals, Fifth Edition	CELF-5
-0,22651255	ASSQ_ASSQ_04	accumulates facts on certain subjects (good rote memory) but does not really understand the meaning	Autism Spectrum Screening Questionnaire	ASSQ
0,226322458	lh_inferiorparietal_volume	Cortical volume in lh inferiorparietal	Cortical Volume	NA
-0,226062643	PCIAT_PCIAT_06	6. How often do your child's grades suffer because of the amount of time he or she spends online?	Parent-Child Internet Addiction Test	PCIAT
0,225985927	rh_parsorbitalis_GI	Gyrification of rh parsorbitalis GI	Local Gyrification Index	NA
0,225228214	rh_frontalpole_volume	Cortical volume in rh frontalpole	Cortical Volume	NA
-0,225157	SWAN_SWAN_11	11. Stays seated (when required by class rules or social conventions)	The Strengths and Weaknesses Assessment of Normal Behavior	SWAN
-0,223981057	CBCL_CBCL_27	27. Easily jealous	Child Behavior Checklist	CBCL
-0,223888986	SCQ_SCQ_03	3. Does she/he ever use odd phrases or say the same thing over and over in almost exactly the same way (either phrases that she/he hears other people use or ones that she/he makes up?	Social Communication Questionnaire	SCQ
-0,223817195	DTS_DTS_Total	DTS Total Score	Distress Tolerance Scale	DTS

Correlation coefficient (R)	Variable name in data	Item text	Instrument name	Instrument abbreviation (if applicable)
-0,222431257	CBCL_CBCL_62	62. Poorly coordinated or clumsy	Child Behavior Checklist	CBCL
-0,22216406	SWAN_SWAN_15	15. Modulates verbal activity (controls excessive talking)	The Strengths and Weaknesses Assessment of Normal Behavior	SWAN
0,221312738	Barratt_Barratt_Total_Edu	Education total score	Barratt Simplified Measure of Social Status	BSMSS
0,221205594	lh_precuneus_volume	Cortical volume in lh precuneus	Cortical Volume	NA
-0,2211112	CBCL_CBCL_94	94. Teases a lot	Child Behavior Checklist	CBCL
-0,220548711	SRS_SRS_32	32. Has good personal hygiene.	Social Responsiveness Scale	SRS
-0,220441845	ASSQ_ASSQ_19	is poor at games; no idea of cooperating in a team, scores ‘own goals’	Autism Spectrum Screening Questionnaire	ASSQ
-0,219713573	PreInt_EduHx_reading	Books/reading	Interview- Education and Social History	Edu/Soc.Hist.
-0,219682734	SRS_SRS_21	21. Is able to imitate others' actions.	Social Responsiveness Scale	SRS
0,219632389	APQ_P_APQ_P_23	23. Your child helps plan family activities	Alabama Parenting Questionnaire	APQ
0,219308812	lh_inferiortemporal_GI	Gyrification of lh inferiortemporal GI	Local Gyrification Index	NA
-0,218467701	ASSQ_ASSQ_16	can be with other children but only on his/her terms	Autism Spectrum Screening Questionnaire	ASSQ
0,218315779	Barratt_Barratt_Total	Barratt Total Score	Barratt Simplified Measure of Social Status	BSMSS
-0,21827526	SCARED_P_SCARED_P_05	5. My child worries about other people liking him/her	Screen for Child Anxiety Related Disorders	SCARED
-0,218190273	PCIAT_PCIAT_18	18. How often does your child become angry or belligerent when your place time limits on how much time he or shes is allowed to spend online?	Parent-Child Internet Addiction Test	PCIAT
-0,218161571	CBCL_CBCL_97	97. Threatens people	Child Behavior Checklist	CBCL

Correlation coefficient (R)	Variable name in data	Item text	Instrument name	Instrument abbreviation (if applicable)
0,217628499	lh_transversetemporal_volume	Cortical volume in lh transversetemporal	Cortical Volume	NA
-0,217523474	PCIAT_PCIAT_11	11. How often does your child spend time along in his or her room playing on the computer?	Parent-Child Internet Addiction Test	PCIAT
-0,217368535	PCIAT_PCIAT_01	1. How often does your child disobey time limits you set for online use?	Parent-Child Internet Addiction Test	PCIAT
-0,217124114	CBCL_CBCL_20	20. Destroys his/her own things	Child Behavior Checklist	CBCL
0,216910892	APQ_P_APQ_P_07	7. You play games or do other fun things with your child	Alabama Parenting Questionnaire	APQ
-0,216867695	CBCL_CBCL_66	66. Repeats certain acts over and over; compulsions	Child Behavior Checklist	CBCL
-0,216690883	SympChck_CSC_03C	Has a loss of interest in previously enjoyable activities (current)	Symptom Checklist Parent report	SympChck-P
0,216373274	lh_pericalcarine_volume	Cortical volume in lh pericalcarine	Cortical Volume	NA
-0,216357069	CBCL_CBCL_90	90. Swearing or obscene language	Child Behavior Checklist	CBCL
-0,216254532	CBCL_CBCL_17	17. Daydreams or gets lost in his/her thoughts	Child Behavior Checklist	CBCL
-0,215623508	SDQ_SDQ_11	Has at least one good friend	Strength and Difficulties Questionnaire	SDQ
-0,214488942	CBCL_CBCL_39	39. Hangs around with others who get in trouble	Child Behavior Checklist	CBCL
0,214411386	lh_transversetemporal_area	Cortical area in lh transversetemporal	Cortical Area	NA
-0,214359007	CBCL_CBCL_42	42. Would rather be along than with others	Child Behavior Checklist	CBCL
-0,213913649	SympChck_CSC_04C	Often feels overly happy and silly, above and	Symptom Checklist Parent report	SympChck-P

Correlation coefficient (R)	Variable name in data	Item text	Instrument name	Instrument abbreviation (if applicable)
		beyond a normal feeling (current)		
-0,213742601	APQ_P_APQ_P_12	12. You give up trying to get your child to obey you because it's too much trouble	Alabama Parenting Questionnaire	APQ
0,21338564	rh_pericalcarine_volume	Cortical volume in rh pericalcarine	Cortical Volume	NA
-0,213307555	DTS_DTS_tolerance	Tolerance subscale	Distress Tolerance Scale	DTS
0,213160826	lh_isthmuscingulate_volume	Cortical volume in lh isthmuscingulate	Cortical Volume	NA
-0,212729558	SDQ_SDQ_02	Restless, overactive, cannot stay still for long	Strength and Difficulties Questionnaire	SDQ
0,21255922	lh_postcentral_area	Cortical area in lh postcentral	Cortical Area	NA
-0,212210583	ASSQ_ASSQ_14	has a deviant style of gaze (e.g. may range from not looking people in the eye, to the other extreme of staring directly at people to the point it makes them uncomfortable)	Autism Spectrum Screening Questionnaire	ASSQ
0,21211889	lh_temporalpole_area	Cortical area in lh temporalpole	Cortical Area	NA
0,212005105	lh_caudalmiddlefrontal_area	Cortical area in lh caudalmiddlefrontal	Cortical Area	NA
-0,211739936	CBCL_CBCL_37	37. Gets in many fights	Child Behavior Checklist	CBCL
-0,211715872	SCQ_SCQ_07	7. Does she/he ever say the same thing over and over again?	Social Communication Questionnaire	SCQ
0,21090971	CTOPP_CTOPP_BW_S	Blending Words scaled score	Comprehensive Test of Phonological Processing	CTOPP-2
-0,210886186	SWAN_SWAN_13	13. Plays quietly (keeps noise level reasonable)	The Strengths and Weaknesses Assessment of Normal Behavior	SWAN
-0,210830885	SRS_SRS_56	56. Walks in between two people who are talking.	Social Responsiveness Scale	SRS

Correlation coefficient (R)	Variable name in data	Item text	Instrument name	Instrument abbreviation (if applicable)
-0,210712125	SCARED_P_SCARED_P_GD	Generalized Anxiety Disorder Score	Screen for Child Anxiety Related Disorders	SCARED
-0,210310191	PreInt_TxHx_Past_DX	Has your chid ever been diagnosed with a psychiatric or learning disorder?	Interview-Treatment History	Treat.hist.
0,210067196	rh_inferiortemporal_GI	Gyrification of rh inferiortemporal GI	Local Gyrification Index	NA
0,209891785	CTOPP_CTOPP_BW_P	Blending Words percentile score	Comprehensive Test of Phonological Processing	CTOPP-2
0,209249624	lh_precentral_area	Cortical area in lh precentral	Cortical Area	NA
-0,209128385	APQ_P_APQ_P_03	3. You threaten to punish your child and then do not actually punish him/her	Alabama Parenting Questionnaire	APQ
-0,208643294	CBCL_CBCL_34	34. Feels others are out to get him/her	Child Behavior Checklist	CBCL
-0,207922476	SRS_SRS_20	20. Shows unusual sensory interests (e.g., mouthing or spinning objects) or strange ways of playing with toys	Social Responsiveness Scale	SRS
0,207883317	lh_bankssts_volume	Cortical volume in lh bankssts	Cortical Volume	NA
-0,207812011	SympChck_CSC_10P	Has unusual thoughts or beliefs that others cannot understand or believe (past	Symptom Checklist Parent report	SympChck-P
-0,207006845	CBCL_CBCL_48	48. Not liked by other kids	Child Behavior Checklist	CBCL
-0,206454912	CBCL_CBCL_16	16. Cruelty, bullying, or meanness to others	Child Behavior Checklist	CBCL
0,206148479	rh_bankssts_area	Cortical area in rh bankssts	Cortical Area	NA
0,205462623	SDQ_SDQ_09	Helpful if someone is hurt, upset or feeling ill	Strength and Difficulties Questionnaire	SDQ
-0,205286081	SympChck_CSC_05P	Has strong and explosive feelings of anger (past	Symptom Checklist Parent report	SympChck-P

Correlation coefficient (R)	Variable name in data	Item text	Instrument name	Instrument abbreviation (if applicable)
-0,204248328	PCIAT_PCIAT_17	17. How often does your child choose to spend time online rather than doing once enjoyed hobbies and/or outside interests?	Parent-Child Internet Addiction Test	PCIAT
-0,204011291	PCIAT_PCIAT_05	5. How often do you complain about the amount of time your child spends online?	Parent-Child Internet Addiction Test	PCIAT
-0,203872671	SCQ_SCQ_38	38. If you come into a room and start talkking to her/him without calling her/his name, does she/he usually look up and pay attention to you?	Social Communication Questionnaire	SCQ
-0,203605468	SympChck_CSC_10C	Has unusual thoughts or beliefs that others cannot understand or believe (current)	Symptom Checklist Parent report	SympChck-P
-0,203376482	Age	Age	Demographics	Demographics
-0,202857058	PCIAT_PCIAT_19	19. How often does your child choose to spend more time online than going out with friends?	Parent-Child Internet Addiction Test	PCIAT
-0,20247344	SympChck_CSC_44C	Bullies, threatens, or intimidates others (current)	Symptom Checklist Parent report	SympChck-P
-0,20167269	APQ_P_APQ_P_39	39. You yell or scream at your child when he/she has done something wrong	Alabama Parenting Questionnaire	APQ
0,20147189	rh_entorhinal_volume	Cortical volume in rh entorhinal	Cortical Volume	NA
-0,200716	SympChck_CSC_49P	Feels really upset when there is an unexpected change in his/her schedule (past	Symptom Checklist Parent report	SympChck-P
0,198895973	CTOPP_CTOPP_RD_S	Rapid Digit Naming scaled score	Comprehensive Test of Phonological Processing	CTOPP-2
-0,198883249	CBCL_CBCL_75	75. Too shy or timid	Child Behavior Checklist	CBCL

Correlation coefficient (R)	Variable name in data	Item text	Instrument name	Instrument abbreviation (if applicable)
-0,198620279	SCQ_SCQ_39	39. Does she/he ever play imaginative games with another child in such a way that you can tell that each child understands what the other is pretending?	Social Communication Questionnaire	SCQ
-0,198592113	NLES_P_NLES_P_TotalEvents	Total # of Negative Events	Negative Life Events Scale	NLES
-0,198381508	PCIAT_PCIAT_13	13. How often does your child snap, yell, or act annoyed if bothered while online?	Parent-Child Internet Addiction Test	PCIAT
-0,19818211	Pegboard_peg_time_nd	Non-dominant Hand - Completion time (sec)	Grooved Pegboard	Pegboard
-0,196954559	SRS_SRS_06	6. Would rather be alone than with others.	Social Responsiveness Scale	SRS
0,196190066	rh_bankssts_volume	Cortical volume in rh bankssts	Cortical Volume	NA
0,196137084	Barratt_Barratt_P1_Edu	Parent 1 level of education	Barratt Simplified Measure of Social Status	BSMSS
-0,195537495	SDQ_SDQ_12	Often fights with other children or bullies them (for 11-17 year olds: Often fights with other youth or bullies them)	Strength and Difficulties Questionnaire	SDQ
0,195495418	lh_postcentral_volume	Cortical volume in lh postcentral	Cortical Volume	NA
0,195206416	lh_LGN	Volume of the left LGN	Thalamic Nuclei	NA
-0,195033422	SympChck_CSC_01C	Feels sad and down most days for at least 1 week (current)	Symptom Checklist Parent report	SympChck-P
-0,194392163	ASSQ_ASSQ_26	has markedly unusual facial expression	Autism Spectrum Screening Questionnaire	ASSQ
-0,193921883	PCIAT_PCIAT_20	20. How often does your child feel depressed, moody, or nervous when off-line which seems to go	Parent-Child Internet Addiction Test	PCIAT

Correlation coefficient (R)	Variable name in data	Item text	Instrument name	Instrument abbreviation (if applicable)
		away once back online?		
-0,193823064	Physical_Weight	Weight (lbs)	Physical Measures	Physical
-0,192784431	ASSQ_ASSQ_20	has clumsy, ill coordinated, ungainly, awkward movements or gestures	Autism Spectrum Screening Questionnaire	ASSQ
-0,192713024	Physical_Height	Height (in)	Physical Measures	Physical
-0,192503196	SRS_SRS_45	45. Focuses his or her attention to where others are looking or listening.	Social Responsiveness Scale	SRS
0,192091903	CTOPP_CTOPP_RSN_Comp	Rapid Symbolic Naming (RD+RL) composite score	Comprehensive Test of Phonological Processing	CTOPP-2
0,191893824	CTOPP_CTOPP_RSN_Sum	Rapid Symbolic Naming (RD+RL) sum score	Comprehensive Test of Phonological Processing	CTOPP-2
-0,191486902	Pegboard_peg_time_d	Dominant Hand - Completion time (sec)	Grooved Pegboard	Pegboard
0,19148005	left_Basal_nucleus	Volume of the left Basal nucleus	Amygdala Nuclei	NA
-0,191291847	SDQ_SDQ_10	Constantly fidgeting or squirming	Strength and Difficulties Questionnaire	SDQ
-0,191287227	ICU_P_ICU_P_06	6. Does not show emotions.	Inventory of Callous-Unemotional Traits Parent	ICU
0,191226453	CTOPP_CTOPP_RD_P	Rapid Digit Naming percentile score	Comprehensive Test of Phonological Processing	CTOPP-2
0,191104363	lh_bankssts_area	Cortical area in lh bankssts	Cortical Area	NA
0,190870058	rh_precentral_area	Cortical area in rh precentral	Cortical Area	NA
-0,190834504	SympChck_CSC_19C	Often feels very nervous around people (current)	Symptom Checklist Parent report	SympChck-P
-0,190709399	PreInt_Demos_Fam_guardian_maritalstatus	Parent(s)/Guardian(s) Marital Status:	Interview-Demographics/Family	Demog.Fam.
-0,19049614	DTS_DTS_regulation	Regulation subscale	Distress Tolerance Scale	DTS
0,190351314	lh_caudalanteriorcingulate_volume	Cortical volume in lh	Cortical Volume	NA

Correlation coefficient (R)	Variable name in data	Item text	Instrument name	Instrument abbreviation (if applicable)
		caudalanteriorcin gulate		
-0,190268954	APQ_P_APQ_P_OPD	Other Discipline Practices Score (Not factored into total score but provides item level information)	Alabama Parenting Questionnaire	APQ
-0,189961439	DTS_DTS_10	10. Being distressed or upset is always a major ordeal for me	Distress Tolerance Scale	DTS
-0,189925934	SCQ_SCQ_40	40. Does she/he play cooperatively in games that need some form of joining in with a group of other children, such as hide-and-seek or ball games?	Social Communication Questionnaire	SCQ
-0,189890143	CBCL_CBCL_12	12. Complains of loneliness	Child Behavior Checklist	CBCL
-0,189760941	SRS_SRS_52	52. Knows when he or she is talking too loud or making too much noise.	Social Responsiveness Scale	SRS
0,189222547	Barratt_Barratt_Total_Occ	Occupation total score	Barratt Simplified Measure of Social Status	BSMSS
0,189076744	lh_parsorbitalis_GI	Gyrification of lh parsorbitalis GI	Local Gyrification Index	NA
-0,187902108	SympChck_CSC_03P	Has a loss of interest in previously enjoyable activities (past	Symptom Checklist Parent report	SympChck-P
-0,187896394	PCIAT_PCIAT_15	15. How often does your child seem preoccupied with being back online when off-line?	Parent-Child Internet Addiction Test	PCIAT
-0,187357762	ASSQ_ASSQ_22	has difficulties in completing simple daily activities because of compulsory repetition of certain actions or thoughts (i.e. any habits that s/he just has to do?)	Autism Spectrum Screening Questionnaire	ASSQ

Correlation coefficient (R)	Variable name in data	Item text	Instrument name	Instrument abbreviation (if applicable)
-0,187138661	SympChck_CSC_45P	Has stolen or shoplifted items (past	Symptom Checklist Parent report	SympChck-P
-0,187129797	right_subiculum_body	Volume of the right subiculum body	Hippocampus Subfields	NA
-0,186761448	PCIAT_PCIAT_16	16. How often does your child throw tantrums with your interference about how long he or she spends online?	Parent-Child Internet Addiction Test	PCIAT
0,186336954	Pegboard_peg_z_d	Dominant Hand - z-score	Grooved Pegboard	Pegboard
-0,186079832	NLES_P_NLES_P_Aware	# of Negative Events the Child was aware of	Negative Life Events Scale	NLES
0,18593796	CTOPP_CTOPP_RSN_P	Rapid Symbolic Naming (RD+RL) percentile score	Comprehensive Test of Phonological Processing	CTOPP-2
0,185873099	rh_precuneus_area	Cortical area in rh precuneus	Cortical Area	NA
-0,185485983	PCIAT_PCIAT_10	10. How often have you caught your child sneaking online against your wishes?	Parent-Child Internet Addiction Test	PCIAT
-0,185387585	NLES_P_NLES_P_Upset_Total	Child's Total Upsetness/Negative Events	Negative Life Events Scale	NLES
-0,184911324	ICU_P_ICU_P_19	19. Is very expressive and emotional.	Inventory of Callous-Unemotional Traits Parent	ICU
-0,184650313	APQ_P_APQ_P_CP	Corporal Punishment Score	Alabama Parenting Questionnaire	APQ
-0,184535809	SympChck_CSC_36P	Has difficulty remaining seated at home or school (past	Symptom Checklist Parent report	SympChck-P
0,184314228	right_Basal_nucleus	Volume of the right Basal nucleus	Amygdala Nuclei	NA
0,184273138	left_Whole_amygdala	Volume of the left Whole amygdala	Amygdala Nuclei	NA
-0,183510323	ASSQ_ASSQ_24	shows idiosyncratic attachment to objects (i.e. may get strangely attached to	Autism Spectrum Screening Questionnaire	ASSQ

Correlation coefficient (R)	Variable name in data	Item text	Instrument name	Instrument abbreviation (if applicable)
		objects as if they were people)		
-0,183392278	SympChck_CSC_01P	Feels sad and down most days for at least 1 week (past	Symptom Checklist Parent report	SympChck-P
0,183383973	lh_entorhinal_GI	Gyrification of lh entorhinal GI	Local Gyrification Index	NA
-0,182657961	DTS_DTS_03	3. I can't handle feeling distressed or upset.	Distress Tolerance Scale	DTS
-0,182452215	CBCL_CBCL_102	102. Underactive, slow moving, or lacks energy	Child Behavior Checklist	CBCL
-0,182117921	SCQ_SCQ_36	36. Does she/he seem interested in other children of approximately the same age whom she/he does not know?	Social Communication Questionnaire	SCQ
-0,181991182	CBCL_CBCL_11	11. Clings to adults or too dependent	Child Behavior Checklist	CBCL
0,181959757	SDQ_SDQ_04	Shares readily with other children, for example toys, treats, pencils (for 11-17 year olds: Shares readily with other youth, for example CD's, games, food)	Strength and Difficulties Questionnaire	SDQ
0,181889225	PreInt_Demos_Home_living_01	Child lives with Both biological parents	Interview-Demographic and Household Information	Demog.Household.
-0,181824703	SRS_SRS_54	54. Seems to react to people as if they are objects.	Social Responsiveness Scale	SRS
0,181311569	rh_inferiorparietal_volume	Cortical volume in rh inferiorparietal	Cortical Volume	NA
-0,18090145	SympChck_CSC_19P	Often feels very nervous around people (past	Symptom Checklist Parent report	SympChck-P
-0,180539807	CBCL_CBCL_35	35. Feels worthless or inferior	Child Behavior Checklist	CBCL
0,179871374	rh_entorhinal_GI	Gyrification of rh entorhinal GI	Local Gyrification Index	NA

Correlation coefficient (R)	Variable name in data	Item text	Instrument name	Instrument abbreviation (if applicable)
0,179672835	CTOPP_CTOPP_NR_S	Nonword Repetition scaled score	Comprehensive Test of Phonological Processing	CTOPP-2
0,179164568	left_Accessory_Basal_nucleus	Volume of the left Accessory Basal nucleus	Amygdala Nuclei	NA
-0,179026266	CELF_CELF_CriterionScore	CELF-5 Criterion Score	Clinical Evaluation of Language Fundamentals, Fifth Edition	CELF-5
0,178690998	rh_inferiorparietal_area	Cortical area in rh inferiorparietal	Cortical Area	NA
0,178073082	APQ_SR_APQ_SR_INV_D	Mother Involvement Score	Alabama Parenting Questionnaire	APQ
-0,177897138	DTS_DTS_05	5. There's nothing worse than feeling distressed or upset	Distress Tolerance Scale	DTS
-0,177388295	APQ_P_APQ_P_22	22. You let your child out of a punishment early (like lift restrictions earlier than you originally said)	Alabama Parenting Questionnaire	APQ
-0,177123337	SCQ_SCQ_26	26. Does she/he usually look at you directly in the face when doing things with you or talking with you?	Social Communication Questionnaire	SCQ
-0,17588185	SCQ_SCQ_08	8. Does she/he ever have things that she/he seems to have to do in a very particular way or order or rituals that she/he insists that you go though?	Social Communication Questionnaire	SCQ
-0,175624459	SympChck_CSC_06C	Has periods of unusual energy and activity (current)	Symptom Checklist Parent report	SympChck-P
-0,175589218	APQ_P_APQ_P_PM	Poor Monitoring/Supervision Score	Alabama Parenting Questionnaire	APQ
-0,175189113	SDQ_SDQ_08	Many worries or often seems worried	Strength and Difficulties Questionnaire	SDQ
-0,17503887	DTS_DTS_01	1. Feeling distressed or	Distress Tolerance Scale	DTS

Correlation coefficient (R)	Variable name in data	Item text	Instrument name	Instrument abbreviation (if applicable)
		upset is unbearable to me.		
0,174341322	right_Whole_amygdala	Volume of the right Whole amygdala	Amygdala Nuclei	NA
-0,174333462	SympChck_CSC_06P	Has periods of unusual energy and activity (past	Symptom Checklist Parent report	SympChck-P
0,174187114	CTOPP_CTOPP_RL_S	Rapid Letter Naming scaled score	Comprehensive Test of Phonological Processing	CTOPP-2
0,174040299	lh_entorhinal_volume	Cortical volume in lh entorhinal	Cortical Volume	NA
0,173551626	CTOPP_CTOPP_RL_P	Rapid Letter Naming percentile score	Comprehensive Test of Phonological Processing	CTOPP-2
-0,173193007	SRS_SRS_43	43. Separates easily from caregivers.	Social Responsiveness Scale	SRS
-0,173014925	SRS_SRS_53	53. Talks to people with an unusual tone of voice (e.g., talks like a robot or like he or she is giving a lecture).	Social Responsiveness Scale	SRS
-0,172255976	SRS_SRS_55	55. Knows when he or she is talking too loud or making too much noise.	Social Responsiveness Scale	SRS
-0,170788578	SCARED_P_SCARED_P_32	32. My child feels shy with people he/she doesn't know well	Screen for Child Anxiety Related Disorders	SCARED
0,17051334	rh_temporalpole_GI	Gyrification of rh temporalpole GI	Local Gyrification Index	NA
-0,169830592	SDQ_SDQ_06	Rather solitary, prefers to play alone (for 11-17 year olds: Would rather be alone than with other youth)	Strength and Difficulties Questionnaire	SDQ
-0,169827395	SRS_SRS_42	42. Seems overly sensitive to sounds, textures, or smells.	Social Responsiveness Scale	SRS
0,169766933	lh_superiorparietal_area	Cortical area in lh superiorparietal	Cortical Area	NA
-0,169467715	SCARED_P_SCARED_P_22	22. When my child gets frightened,	Screen for Child Anxiety Related Disorders	SCARED

Correlation coefficient (R)	Variable name in data	Item text	Instrument name	Instrument abbreviation (if applicable)
		he/she sweats a lot		
0,169263394	rh_isthmuscingulate_volume	Cortical volume in rh isthmuscingulate	Cortical Volume	NA
0,168652291	rh_isthmuscingulate_area	Cortical area in rh isthmuscingulate	Cortical Area	NA
0,168624894	rh_caudalmiddlefrontal_volume	Cortical volume in rh caudalmiddlefrontal	Cortical Volume	NA
-0,168510508	ASSQ_ASSQ_27	has markedly unusual posture	Autism Spectrum Screening Questionnaire	ASSQ
-0,167287347	SWAN_SWAN_12	12. Modulates motor activity (inhibits inappropriate running or climbing)	The Strengths and Weaknesses Assessment of Normal Behavior	SWAN
-0,166833406	CBCL_CBCL_64	64. Prefers being with younger kids	Child Behavior Checklist	CBCL
-0,166438435	SCQ_SCQ_33	33. Does she/he show a normal range of facial expressions?	Social Communication Questionnaire	SCQ
-0,166398777	CBCL_CBCL_84	84. Strange behavior	Child Behavior Checklist	CBCL
0,166115324	lh_temporalpole_volume	Cortical volume in lh temporalpole	Cortical Volume	NA
-0,165524482	ASSQ_ASSQ_03	lives somewhat in a world of his/her own with restricted idiosyncratic intellectual interests	Autism Spectrum Screening Questionnaire	ASSQ
-0,165392977	SCARED_P_SCARED_P_SH	Significant School Avoidance Score	Screen for Child Anxiety Related Disorders	SCARED
-0,165091034	SympChck_CSC_12P	(past	Symptom Checklist Parent report	SympChck-P
-0,164645361	PreInt_EduHx_detention	Detentions (past year)	Interview-Education and Social History	Edu/Soc.Hist.
-0,163685947	CBCL_CBCL_31	31. Fears he/she might think or do something bad	Child Behavior Checklist	CBCL
0,163260929	rh_entorhinal_area	Cortical area in rh entorhinal	Cortical Area	NA
-0,163191573	SDQ_SDQ_22	Steals from home, school or elsewhere	Strength and Difficulties Questionnaire	SDQ

Correlation coefficient (R)	Variable name in data	Item text	Instrument name	Instrument abbreviation (if applicable)
0,163179263	left_Anterior_amygdaloid_area_AAA	Volume of the left Anterior amygdaloid area AAA	Amygdala Nuclei	NA
0,163097348	SDQ_SDQ_17	Kind to younger children	Strength and Difficulties Questionnaire	SDQ
-0,162892778	SympChck_CSC_12C	(current)	Symptom Checklist Parent report	SympChck-P
-0,162465334	SympChck_CSC_44P	Bullies, threatens, or intimidates others (past	Symptom Checklist Parent report	SympChck-P
0,161844893	rh_temporalpole_area	Cortical area in rh temporalpole	Cortical Area	NA
-0,161776351	SCQ_SCQ_29	29. Does she/he ever offer to share things other than food with you?	Social Communication Questionnaire	SCQ
-0,161638378	CBCL_CBCL_50	50. Too fearful or anxious	Child Behavior Checklist	CBCL
-0,16163824	PreInt_Demos_Home_living_03	Child lives with Biological mother	Interview-Demographic and Household Information	Demog.Household.
-0,161618521	CBCL_CBCL_57	57. Physically attacks people	Child Behavior Checklist	CBCL
0,161443023	left_Paralaminar_nucleus	Volume of the left Paralaminar nucleus	Amygdala Nuclei	NA
-0,161274501	SCARED_P_SCARED_P_08	8. My child follows me wherever I go	Screen for Child Anxiety Related Disorders	SCARED
-0,160667596	SCQ_SCQ_06	6. Does she/he ever use words that she/he seems to have invented or made up her/himself; put things in odd, indirect ways; or use metaphorical ways of saying things (e.g., saying hot rain for steam)?	Social Communication Questionnaire	SCQ
-0,160619728	SCQ_SCQ_30	30. Does she/he ever seem to want you to join in her/his enjoyment of something?	Social Communication Questionnaire	SCQ
-0,160192859	ICU_P_ICU_P_03	3. Is concerned about schoolwork.	Inventory of Callous-	ICU

Correlation coefficient (R)	Variable name in data	Item text	Instrument name	Instrument abbreviation (if applicable)
			Unemotional Traits Parent	
-0,160059204	DTS_DTS_absorption	Absorption subscale	Distress Tolerance Scale	DTS
-0,159874932	CBCL_CBCL_63	63. Prefers being with older kids	Child Behavior Checklist	CBCL
-0,159758845	PCIAT_PCIAT_14	14. How often does your child seem more tired and fatigued than he or she did before the Internet came along?	Parent-Child Internet Addiction Test	PCIAT
-0,159446811	CBCL_CBCL_85	85. Strange ideas	Child Behavior Checklist	CBCL
-0,159042249	SympChck_CSC_26C	Is bothered by thoughts which keep coming into his/her head for no reason (current)	Symptom Checklist Parent report	SympChck-P
-0,158602695	APQ_P_APQ_P_17	17. You do not know the friends your child is with	Alabama Parenting Questionnaire	APQ
-0,158357624	SCQ_SCQ_05	5. Does she/he ever get her/his pronouns mixed up (e.g., saying you or she/he for I)?	Social Communication Questionnaire	SCQ
0,157920227	right_Lateral_nucleus	Volume of the right Lateral nucleus	Amygdala Nuclei	NA
-0,157805376	ARI_S_ARI_S_Total_Score	Total Score	Affective Reactivity Index	ARI
0,157638795	WIAT_WIAT_LCODC_Raw	Listening Comprehension Oral Discourse Comprehension Raw Score	Wechsler Individual Achievement Test	WIAT
0,156660965	lh_entorhinal_area	Cortical area in lh entorhinal	Cortical Area	NA
-0,156494798	APQ_P_APQ_P_37	37. You send your child to his/her room as punishment	Alabama Parenting Questionnaire	APQ
0,156429376	Right_Accumbens_area	Volume of the Right Accumbens area	Subcortical Volume	NA
-0,156207344	SCQ_SCQ_35	35. Does she/he play any pretend or make-believe games?	Social Communication Questionnaire	SCQ

Correlation coefficient (R)	Variable name in data	Item text	Instrument name	Instrument abbreviation (if applicable)
-0,156202126	SympChck_CSC_07C	Has times when he/she sleeps much less than usual but still feels rested (current)	Symptom Checklist Parent report	SympChck-P
-0,156048182	CBCL_CBCL_14	14. Cries a lot	Child Behavior Checklist	CBCL
0,155498359	right_Paralaminar_nucleus	Volume of the right Paralaminar nucleus	Amygdala Nuclei	NA
0,155146239	lh_precentral_volume	Cortical volume in lh precentral	Cortical Volume	NA
0,154813632	APQ_SR_APQ_SR_04A	4A. How about your dad?	Alabama Parenting Questionnaire	APQ
-0,154750381	PreInt_EduHx_NeuroPsych	Has your child ever had any neuropsychological testing?	Interview-Education and Social History	Edu/Soc.Hist.
-0,154649264	SCQ_SCQ_18	18. Does she/he ever have any objects (other than a soft toy or comfort blanket) that she/he has to carry around?	Social Communication Questionnaire	SCQ
-0,154600176	PreInt_DevHx_puberty	Has your child shown adult sexual body development (puberty)?	Interview-Developmental History	Dev.Hist.
0,154411356	right_Anterior_amygdaloid_area_AAA	Volume of the right Anterior amygdaloid area AAA	Amygdala Nuclei	NA
0,154388921	rh_precuneus_volume	Cortical volume in rh precuneus	Cortical Volume	NA
-0,154215397	CBCL_CBCL_83	83. Stores up too many things he/she doesn't need	Child Behavior Checklist	CBCL
0,154177536	CELF_CELF_Total	CELF-5 Total Score	Clinical Evaluation of Language Fundamentals, Fifth Edition	CELF-5
-0,154162992	SCARED_P_SCARED_P_SP	Separation Anxiety SOC Score	Screen for Child Anxiety Related Disorders	SCARED
-0,153784535	SCQ_SCQ_14	14. Does she/he ever seem to be unusually interested in the sight, feel, sound, taske, or smell of things or people?	Social Communication Questionnaire	SCQ

Correlation coefficient (R)	Variable name in data	Item text	Instrument name	Instrument abbreviation (if applicable)
0,153727374	rh_paracentral_area	Cortical area in rh paracentral	Cortical Area	NA
0,153291231	left_Lateral_nucleus	Volume of the left Lateral nucleus	Amygdala Nuclei	NA
-0,153147258	SympChck_CSC_42C	Has skipped a part or a whole day of school (current)	Symptom Checklist Parent report	SympChck-P
0,152920642	rh_fusiform_thickness	Cortical thickness in rh fusiform	Cortical Thickness	NA
0,152705743	Left_Amygdala	Volume of the Left Amygdala	Subcortical Volume	NA
-0,152640454	PreInt_EduHx_suspension	Suspensions (past year)	Interview-Education and Social History	Edu/Soc.Hist.
-0,152340297	SCQ_SCQ_20	20. Does she/he ever talk with you just to be friendly (rather than to get something)?	Social Communication Questionnaire	SCQ
-0,151248269	DTS_DTS_02	2. When I feel distressed or upset, all I can think about is how bad I feel	Distress Tolerance Scale	DTS
0,151208202	APQ_P_APQ_P_04	4. You volunteer to help with special activities that your child is involved with (such as sports, boy/girl scouts, church youth groups)	Alabama Parenting Questionnaire	APQ
-0,150701949	ASSQ_ASSQ_21	has involuntary face or body movements (i.e. any tics?)	Autism Spectrum Screening Questionnaire	ASSQ
0,150524069	rh_postcentral_volume	Cortical volume in rh postcentral	Cortical Volume	NA
-0,150334511	SCQ_SCQ_11	11. Does she/he ever have any interests that preoccupy her/him and might seem off to other people (e.g., traffic lights, drainpipes, or timetables?)	Social Communication Questionnaire	SCQ
-0,149886747	SCARED_P_SCARED_P_09	9. People tell me that my child looks nervous	Screen for Child Anxiety Related Disorders	SCARED
-0,149348624	SympChck_CSC_07P	Has times when he/she sleeps much less than	Symptom Checklist Parent report	SympChck-P

Correlation coefficient (R)	Variable name in data	Item text	Instrument name	Instrument abbreviation (if applicable)
		usual but still feels rested (past		
-0,149250631	right_hippocampal_fissure	Volume of the right hippocampal fissure	Hippocampus Subfields	NA
0,149160927	lh_superiorparietal_volume	Cortical volume in lh superiorparietal	Cortical Volume	NA
0,148431861	ICU_P_ICU_P_10	10. Does not let feelings control him/her.	Inventory of Callous-Unemotional Traits Parent	ICU
-0,148285854	SCQ_SCQ_37	37. Does she/he respond positively when another child approaches her/him?	Social Communication Questionnaire	SCQ
-0,148067345	ASSQ_ASSQ_09	expresses sounds involuntarily; clears throat, grunts, smacks, cries or screams	Autism Spectrum Screening Questionnaire	ASSQ
-0,148031846	ASSQ_ASSQ_08	has a different voice or speech	Autism Spectrum Screening Questionnaire	ASSQ
0,14790108	SDQ_SDQ_20	Often offers to help others (parents, teachers, children)	Strength and Difficulties Questionnaire	SDQ
0,147682282	lh_supramarginal_area	Cortical area in lh supramarginal	Cortical Area	NA
-0,14755241	SympChck_CSC_36C	Has difficulty remaining seated at home or school (current)	Symptom Checklist Parent report	SympChck-P
-0,147113272	SCQ_SCQ_34	34. Does she/he ever spontaenously join in and try to copy the actions in social games, such as The Mulberry Bush or London Bridge is Falling Down?	Social Communication Questionnaire	SCQ
-0,146974082	SCQ_SCQ_12	12. Does she/he ever seem to be more interested in parts of a toy or an object (e.g., spinning the wheels of a car), rather than in using the object	Social Communication Questionnaire	SCQ

Correlation coefficient (R)	Variable name in data	Item text	Instrument name	Instrument abbreviation (if applicable)
		as it was intended?		
0,146932903	left_Medial_nucleus	Volume of the left Medial nucleus	Amygdala Nuclei	NA
-0,146583863	ARI_S_ARI_S_02	I often lose my temper	Affective Reactivity Index	ARI
0,146405981	lh_MGN	Volume of the left MGN	Thalamic Nuclei	NA
0,146307058	PreInt_DevHx_m_birthage	Mother's age at birth of child	Interview-Developmental History	Dev.Hist.
0,146253599	Right_Amygdala	Volume of the Right Amygdala	Subcortical Volume	NA
-0,146200681	CBCL_CBCL_56B	56B. Headaches	Child Behavior Checklist	CBCL
0,146184182	lh_paracentral_area	Cortical area in lh paracentral	Cortical Area	NA
0,146161752	rh_LGN	Volume of the right LGN	Thalamic Nuclei	NA
-0,146052774	SympChck_CSC_47C	Makes noises that he/she can't control (repeating sounds, sniffing) (current)	Symptom Checklist Parent report	SympChck-P
-0,145793106	PreInt_EduHx_repeated_grades	Were any grades repeated?	Interview-Education and Social History	Edu/Soc.Hist.
-0,145389083	APQ_P_APQ_P_36	36. You take away privileges or money from your child as punishment	Alabama Parenting Questionnaire	APQ
0,145256919	lh_temporalpole_GI	Gyrification of lh temporalpole GI	Local Gyrification Index	NA
-0,145197874	SDQ_SDQ_24	Many fears, easily scared	Strength and Difficulties Questionnaire	SDQ
0,144787779	rh_superiortemporal_thickness	Cortical thickness in rh superiortemporal	Cortical Thickness	NA
-0,144587618	CBCL_CBCL_77	77. Sleeps more than most kids during day and/or night	Child Behavior Checklist	CBCL
0,144512421	PreInt_EduHx_sports	Playing sports	Interview-Education and Social History	Edu/Soc.Hist.
-0,144241098	PCIAT_PCIAT_04	4. How often does your child form new relationships with fellow online users?	Parent-Child Internet Addiction Test	PCIAT
0,143981913	CTOPP_CTOPP_EL_R	Elision raw score	Comprehensive Test of	CTOPP-2

Correlation coefficient (R)	Variable name in data	Item text	Instrument name	Instrument abbreviation (if applicable)
			Phonological Processing	
-0,143533908	NLES_P_NLES_P_06a	6a. People in the child's family (such as his/her parents, brothers or sisters) physically hit each other hard or hurt each other.	Negative Life Events Scale	NLES
-0,143332691	SympChck_CSC_33C	Has frequent eating binges, and it feels out of control (current)	Symptom Checklist Parent report	SympChck-P
0,142352294	rh_temporalpole_volume	Cortical volume in rh temporalpole	Cortical Volume	NA
0,142224608	lh_supramarginal_volume	Cortical volume in lh supramarginal	Cortical Volume	NA
0,141919162	PreInt_EduHx_bestfriend	Does your child have a best friend?	Interview-Education and Social History	Edu/Soc.Hist.
0,141638694	FGC_FGC_CU_Zone	Curl up fitness zone	FitnessGram Child	FGC
-0,141584098	SCQ_SCQ_31	31. Does she/he ever try to comfort you if you are sad or hurt?	Social Communication Questionnaire	SCQ
0,141057012	Left_Accumbens_area	Volume of the Left Accumbens area	Subcortical Volume	NA
0,141025686	PreInt_EduHx_afterschoolteams	Does your child belong to any groups, sports teams, or organizations?	Interview-Education and Social History	Edu/Soc.Hist.
-0,139904773	APQ_P_APQ_P_08	8. Your child talks you out of being punished after he/she has done something wrong	Alabama Parenting Questionnaire	APQ
-0,139489693	SRS_SRS_50	50. Has repetitive, odd behaviors such as hand flapping or rocking.	Social Responsiveness Scale	SRS
0,139446405	lh_caudalmiddlefrontal_volume	Cortical volume in lh caudalmiddlefrontal	Cortical Volume	NA
-0,139302027	CBCL_CBCL_91	91. Talks about killing self	Child Behavior Checklist	CBCL

Correlation coefficient (R)	Variable name in data	Item text	Instrument name	Instrument abbreviation (if applicable)
-0,13909899	SCQ_SCQ_27	27. Does she/he smile back if someone smiles at her/him?	Social Communication Questionnaire	SCQ
0,138829684	PreInt_Demos_Fam_P2_LegalGuardian	Parent 2 has legal guardianship over the child	Interview-Demographics/Family	Demog.Fam.
-0,13866266	SCQ_SCQ_32	32. If she/he wants something or wants help, does she/he look at you and use gestures with sounds or words to get your attention?	Social Communication Questionnaire	SCQ
-0,138181154	ASSQ_ASSQ_23	has special routines; insists on no change (i.e. may need to have exactly the same change; troubles with even the slightest change in his/her environment, or routines or activities)	Autism Spectrum Screening Questionnaire	ASSQ
0,13799377	rh_lateralorbitofrontal_thickness	Cortical thickness in rh lateralorbitofrontal	Cortical Thickness	NA
-0,137888197	APQ_SR_APQ_SR_PM	Poor Monitoring/Supervision Score	Alabama Parenting Questionnaire	APQ
-0,137670194	CBCL_CBCL_36	36. Gets hurt a lot, accident-prone	Child Behavior Checklist	CBCL
-0,137597434	NLES_P_NLES_P_21a	21a. The child changed schools.	Negative Life Events Scale	NLES
0,137428951	right_Accessory_Basal_nucleus	Volume of the right Accessory Basal nucleus	Amygdala Nuclei	NA
-0,136921782	CBCL_CBCL_24	24. Doesn't eat well	Child Behavior Checklist	CBCL
0,136576281	lh_insula_thickness	Cortical thickness in lh insula	Cortical Thickness	NA
-0,136519598	PreInt_EduHx_learning_disability	Were any learning disabilities identified?	Interview-Education and Social History	Edu/Soc.Hist.
-0,136511709	ARI_S_ARI_S_06	I lose my temper easily	Affective Reactivity Index	ARI
-0,136107871	SDQ_SDQ_03	Often complains of headaches,	Strength and Difficulties Questionnaire	SDQ

Correlation coefficient (R)	Variable name in data	Item text	Instrument name	Instrument abbreviation (if applicable)
		stomach-aches or sickness		
-0,135756235	CBCL_CBCL_112	112. Worries	Child Behavior Checklist	CBCL
-0,135753079	SCQ_SCQ_19	19. Does she/he have any particular friends or a best friend?	Social Communication Questionnaire	SCQ
-0,135732715	CBCL_CBCL_30	30. Fears going to school	Child Behavior Checklist	CBCL
-0,135450756	Physical_BMI	BMI (kg/m^2)	Physical Measures	Physical
-0,134800153	SCARED_P_SCARED_P_37	37. My child worries about things that have already happened	Screen for Child Anxiety Related Disorders	SCARED
0,134542291	lh_lateralorbitofrontal_thickness	Cortical thickness in lh lateralorbitofrontal	Cortical Thickness	NA
-0,133685656	DTS_DTS_11	11. I am ashamed of myself when I feel distressed or upset	Distress Tolerance Scale	DTS
-0,133451754	SCQ_SCQ_09	9. Does her/his facial expression usually seem appropriate to the particular situation, as far as you can tell?	Social Communication Questionnaire	SCQ
-0,13278445	APQ_P_APQ_P_35	35. You slap your child when he/she has done something wrong	Alabama Parenting Questionnaire	APQ
-0,132593633	NLES_P_NLES_P_09a	9a. The child's relatives such as aunts, uncles, grandparents said bad things about his/her mother or father.	Negative Life Events Scale	NLES
-0,132419983	SympChck_CSC_48P	Has unusual physical mannerisms (rocked body or flapping hands) (past	Symptom Checklist Parent report	SympChck-P
0,132369533	PreInt_EduHx_school_sports	Sports	Interview-Education and Social History	Edu/Soc.Hist.
0,132272441	APQ_P_APQ_P_14	14. You ask your child what his/her plans are for the coming day	Alabama Parenting Questionnaire	APQ

Correlation coefficient (R)	Variable name in data	Item text	Instrument name	Instrument abbreviation (if applicable)
0,132229735	Right_Putamen	Volume of the Right Putamen	Subcortical Volume	NA
-0,132207205	right_GC_ML_DG_body	Volume of the right GC ML DG body	Hippocampus Subfields	NA
-0,131703908	NLES_P_NLES_P_14a	14a. The child's mother or father was arrested or sent to jail.	Negative Life Events Scale	NLES
-0,131687283	SympChck_CSC_26P	Is bothered by thoughts which keep coming into his/her head for no reason (past	Symptom Checklist Parent report	SympChck-P
-0,131395622	SympChck_CSC_30C	Feels extremely worried about gaining weight or becoming fat (current)	Symptom Checklist Parent report	SympChck-P
-0,131295534	right_CA4_body	Volume of the right CA4 body	Hippocampus Subfields	NA
-0,130808839	CBCL_CBCL_44	44. Bites fingernails	Child Behavior Checklist	CBCL
0,130337788	Barratt_financialsupport	Who is providing financial support for the child?	Barratt Simplified Measure of Social Status	BSMSS
0,129411332	lh_parahippocampal_volume	Cortical volume in lh parahippocampal	Cortical Volume	NA
0,129011956	rh_MGN	Volume of the right MGN	Thalamic Nuclei	NA
-0,128980291	DTS_DTS_12	12. My feelings of distress or being upset scare me.	Distress Tolerance Scale	DTS
-0,128891543	PreInt_EduHx_weakness_math	Math	Interview-Education and Social History	Edu/Soc.Hist.
-0,128759773	PreInt_EduHx_imaginativeplay	Imaginative play	Interview-Education and Social History	Edu/Soc.Hist.
-0,128671122	NLES_P_NLES_P_13a	13a. The child's mother or father forgot to do important things for him/her that they promised they would do, such as take him/her on a trip, take him/her to nice places, or come to his/her school or athletic activities.	Negative Life Events Scale	NLES
-0,128513217	CBCL_CBCL_51	51. Feels dizzy or lightheaded	Child Behavior Checklist	CBCL

Correlation coefficient (R)	Variable name in data	Item text	Instrument name	Instrument abbreviation (if applicable)
-0,128500231	SympChck_CSC_25C	Feels very nervous and unable to relax most days of the week (current)	Symptom Checklist Parent report	SympChck-P
-0,12841336	APQ_P_APQ_P_06	6. You child fails to leave a note to let you know where he/she is going	Alabama Parenting Questionnaire	APQ
-0,128378812	SCARED_P_SCARED_P_24	24. My child gets really frightened for no reason at all	Screen for Child Anxiety Related Disorders	SCARED
-0,128161204	SympChck_CSC_14P	Worries about being separated from parent/guardian because of getting lost or kidnapped (past	Symptom Checklist Parent report	SympChck-P
0,127954302	lh_parahippocampal_thickness	Cortical thickness in lh parahippocampal	Cortical Thickness	NA
-0,127317234	CC_Posterior	Volume of the CC Posterior	Subcortical Volume	NA
0,127010091	APQ_SR_APQ_SR_07	7. You play games or do other fun things with your mom	Alabama Parenting Questionnaire	APQ
0,126891721	rh_paracentral_volume	Cortical volume in rh paracentral	Cortical Volume	NA
-0,126392412	APQ_P_APQ_P_33	33. You spank your child with your hand when he/she has done something wrong	Alabama Parenting Questionnaire	APQ
-0,126119602	APQ_P_APQ_P_24	24. You get so busy that you forget where your child is and what he/she is doing	Alabama Parenting Questionnaire	APQ
0,12580334	APQ_SR_APQ_SR_INV_M	Father Involvement Score	Alabama Parenting Questionnaire	APQ
-0,125778153	ASSQ_ASSQ_05	has a literal understanding of ambiguous and metaphoric language (i.e. takes things literally; troubles understanding expressions or metaphors)	Autism Spectrum Screening Questionnaire	ASSQ
0,12491621	lh_paracentral_volume	Cortical volume in lh paracentral	Cortical Volume	NA

Correlation coefficient (R)	Variable name in data	Item text	Instrument name	Instrument abbreviation (if applicable)
-0,124715845	SympChck_CSC_11C	Has anxiety attacks, where out of the blue he/she suddenly feels scared (current)	Symptom Checklist Parent report	SympChck-P
-0,124477053	CBCL_CBCL_81	81. Steals at home	Child Behavior Checklist	CBCL
0,122839521	rh_postcentral_thickness	Cortical thickness in rh postcentral	Cortical Thickness	NA
0,12263415	APQ_P_APQ_P_20	20. You talks to your child about his/her friends	Alabama Parenting Questionnaire	APQ
-0,12261938	left_subiculum_body	Volume of the left subiculum body	Hippocampus Subfields	NA
-0,122424338	SympChck_CSC_15C	Worries excessively about harm occuring to parents/guardians (current)	Symptom Checklist Parent report	SympChck-P
-0,122246313	PreInt_EduHx_CPSE	Does your child have CPSE services?	Interview- Education and Social History	Edu/Soc.Hist.
-0,121759542	ASSQ_ASSQ_07	invents idiosyncratic words and expressions (i.e. makes up his or her own words, expressions or names for things)	Autism Spectrum Screening Questionnaire	ASSQ
0,12146432	Right_Caudate	Volume of the Right Caudate	Subcortical Volume	NA
-0,1210392	SCQ_SCQ_13	13. Does she/he ever have any special interests that are unusual in their intensity but otherwise appropriate for her/his age and peer group (e.g., trains or dinosaurs)?	Social Communication Questionnaire	SCQ
-0,1209469	SympChck_CSC_25P	Feels very nervous and unable to relax most days of the week (past	Symptom Checklist Parent report	SympChck-P
0,120307739	lh_inferiortemporal_thickness	Cortical thickness in lh inferiortemporal	Cortical Thickness	NA
-0,120198346	PreInt_EduHx_EI	Does your child have EI services?	Interview- Education and Social History	Edu/Soc.Hist.

Correlation coefficient (R)	Variable name in data	Item text	Instrument name	Instrument abbreviation (if applicable)
-0,119847561	SympChck_CSC_43P	Frequently starts physical fights with peers (past	Symptom Checklist Parent report	SympChck-P
-0,119424546	PreInt_EduHx_weakness_english	English	Interview- Education and Social History	Edu/Soc.Hist.
-0,11935324	SympChck_CSC_48C	Has unusual physical mannerisms (rocked body or flapping hands) (current)	Symptom Checklist Parent report	SympChck-P
0,119308248	rh_middletemporal_thickness	Cortical thickness in rh middletemporal	Cortical Thickness	NA
-0,119172654	NLES_P_NLES_P_08a	8a. The child's mother or father talked about having serious money troubles (being worried about bills for ordinary things).	Negative Life Events Scale	NLES
-0,118907552	APQ_P_APQ_P_29	29. You don't tell your child where you are going	Alabama Parenting Questionnaire	APQ
-0,11883101	SRS_SRS_63	63. Touches others in an unusual way (e.g., he or she may touch someone just to make contact and then walk away without saying anything).	Social Responsiveness Scale	SRS
0,11778849	rh_insula_thickness	Cortical thickness in rh insula	Cortical Thickness	NA
0,117407986	APQ_SR_APQ_SR_23	23. You help plan family activities	Alabama Parenting Questionnaire	APQ
-0,11738673	CBCL_CBCL_76	76. Sleeps less than most kids	Child Behavior Checklist	CBCL
-0,116717494	CBCL_CBCL_46	46. Nervous movements or twitching	Child Behavior Checklist	CBCL
0,116441557	CTOPP_CTOPP_BW_R	Blending Words raw score	Comprehensive Test of Phonological Processing	CTOPP-2
0,116264854	Left_Cerebellum_Cortex	Volume of the Left Cerebellum Cortex	Subcortical Volume	NA
-0,116107234	CC_Mid_Posterior	Volume of the CC Mid Posterior	Subcortical Volume	NA

Correlation coefficient (R)	Variable name in data	Item text	Instrument name	Instrument abbreviation (if applicable)
-0,116021879	right_Whole_hippocampal_body	Volume of the right Whole hippocampal body	Hippocampus Subfields	NA
-0,116021738	PreInt_DevHx_preg_symp_02	Emotional problems	Interview-Developmental History	Dev.Hist.
0,115795231	APQ_SR_APQ_SR_15A	15A. How about your dad?	Alabama Parenting Questionnaire	APQ
-0,114876438	SCQ_SCQ_15	15. Does she/he ever have any mannerisms or odd ways of moving her/his hands or fingers, such as flapping or moving her/his fingers in front or her/his eyes?	Social Communication Questionnaire	SCQ
-0,114767308	ARI_S_ARI_S_01	I am easily annoyed by others	Affective Reactivity Index	ARI
-0,114668414	SympChck_CSC_23P	Worries most days of the week (past	Symptom Checklist Parent report	SympChck-P
-0,114630784	SympChck_CSC_27P	Feels like he/she has to do certain things in a very specific way (handwashing, checking, doing things multiple times) (past	Symptom Checklist Parent report	SympChck-P
-0,114527039	PreInt_FamHx_RDC_ffdk	Don't know father's father's current age	Family History/Research Diagnostic Criteria	Fam.Med.Hist
-0,114488726	SympChck_CSC_23C	Worries most days of the week (current)	Symptom Checklist Parent report	SympChck-P
0,11424225	lh_superiortemporal_thickness	Cortical thickness in lh superiortemporal	Cortical Thickness	NA
0,113599449	left_Cortical_nucleus	Volume of the left Cortical nucleus	Amygdala Nuclei	NA
-0,113504371	SympChck_CSC_24P	Often misses school or other activities because he/she doesn't feel well (past	Symptom Checklist Parent report	SympChck-P
-0,113177182	PreInt_DevHx_temp_11	Problems with social relatedness	Interview-Developmental History	Dev.Hist.
-0,112872988	left_hippocampal_fissure	Volume of the left hippocampal fissure	Hippocampus Subfields	NA

Correlation coefficient (R)	Variable name in data	Item text	Instrument name	Instrument abbreviation (if applicable)
-0,112644459	CBCL_CBCL_07	7. Bragging, boasting	Child Behavior Checklist	CBCL
0,111875973	lh_PuM	Volume of the left PuM	Thalamic Nuclei	NA
-0,110810858	SCARED_P_SCARED_P_31	31. My child worries that something bad will happen to his/her parents	Screen for Child Anxiety Related Disorders	SCARED
0,110791294	WIAT_WIAT_Pseudo_Raw	Pseudo-word Decoding Raw Score	Wechsler Individual Achievement Test	WIAT
0,110554748	APQ_SR_APQ_SR_01	1. You have a friendly talk with your mom	Alabama Parenting Questionnaire	APQ
0,110414564	rh_precentral_volume	Cortical volume in rh precentral	Cortical Volume	NA
0,110320291	lh_MDm	Volume of the left MDm	Thalamic Nuclei	NA
0,110274594	PreInt_EduHx_music	Music	Interview- Education and Social History	Edu/Soc.Hist.
-0,109745591	APQ_P_APQ_P_42	42. You give your child extra chores as punishment	Alabama Parenting Questionnaire	APQ
-0,108492802	SCQ_SCQ_25	25. Does she/he shake her/his head to indicate no?	Social Communication Questionnaire	SCQ
-0,108468364	SCARED_P_SCARED_P_35	35. My child worries about how well he/she does things	Screen for Child Anxiety Related Disorders	SCARED
-0,108393665	SympChck_CSC_15P	Worries excessively about harm occuring to parents/guardians (past	Symptom Checklist Parent report	SympChck-P
0,108387683	Left_Caudate	Volume of the Left Caudate	Subcortical Volume	NA
0,108260077	rh_inferiortemporal_thickness	Cortical thickness in rh inferiortemporal	Cortical Thickness	NA
0,108194859	lh_fusiform_thickness	Cortical thickness in lh fusiform	Cortical Thickness	NA
0,107753074	PreInt_EduHx_strength_english	English	Interview- Education and Social History	Edu/Soc.Hist.
0,10766237	left_Corticoamygdaloid_transitio	Volume of the left Corticoamygdaloid transitio	Amygdala Nuclei	NA
-0,107361227	SCARED_P_SCARED_P_01	1. When my child feels frightened, it is hard to breathe	Screen for Child Anxiety Related Disorders	SCARED

Correlation coefficient (R)	Variable name in data	Item text	Instrument name	Instrument abbreviation (if applicable)
0,107325912	rh_MDl	Volume of the right MDl	Thalamic Nuclei	NA
0,107313171	Right_Cerebellum_Cortex	Volume of the Right Cerebellum Cortex	Subcortical Volume	NA
0,107105097	rh_LP	Volume of the right LP	Thalamic Nuclei	NA
-0,106856874	NLES_P_NLES_P_07a	7a. The child's parent suffered from serious illness, injury, or extreme pain, something that required rest for one week in bed, hospitalization, or surgery.	Negative Life Events Scale	NLES
-0,106849553	SympChck_CSC_27C	Feels like he/she has to do certain things in a very specific way (handwashing, checking, doing things multiple times) (current)	Symptom Checklist Parent report	SympChck-P
-0,106780859	rh_precentral_thickness	Cortical thickness in rh precentral	Cortical Thickness	NA
0,106773898	APQ_SR_APQ_SR_26A	26A. How about your dad?	Alabama Parenting Questionnaire	APQ
-0,106192288	PreInt_FamHx_RDC_mffdk	Mother's paternal grandmother current age range, or age range at death:	Family History/Research Diagnostic Criteria	Fam.Med.Hist
0,105138992	APQ_SR_APQ_SR_01A	1A. How about your dad?	Alabama Parenting Questionnaire	APQ
0,105052462	APQ_P_APQ_P_15	15. You drive your child to a special activity	Alabama Parenting Questionnaire	APQ
0,104861948	leftPosterior	Volume of the left Posterior	Hypothalamic Subunits	NA
-0,104692509	SympChck_CSC_24C	Often misses school or other activities because he/she doesn't feel well (current)	Symptom Checklist Parent report	SympChck-P
-0,104459969	SympChck_CSC_11P	Has anxiety attacks, where out of the blue he/she suddenly feels scared (past	Symptom Checklist Parent report	SympChck-P
0,104369393	right_fimbria	Volume of the right fimbria	Hippocampus Subfields	NA

Correlation coefficient (R)	Variable name in data	Item text	Instrument name	Instrument abbreviation (if applicable)
-0,104146301	SympChck_CSC_47P	Makes noises that he/she can't control (repeating sounds, sniffing) (past	Symptom Checklist Parent report	SympChck-P
0,103790959	rh_parahippocampal_thickness	Cortical thickness in rh parahippocampal	Cortical Thickness	NA
0,103768029	rh_MDm	Volume of the right MDm	Thalamic Nuclei	NA
0,103691603	right_Medial_nucleus	Volume of the right Medial nucleus	Amygdala Nuclei	NA
0,103478732	APQ_P_APQ_P_40	40. You calmly explain to your child why his/her behavior was wrong when he/she misbehaves	Alabama Parenting Questionnaire	APQ
-0,102789557	SCQ_SCQ_16	16. Does she/he ever have any complicated movements of her/his whole body, such as spinning or repeatedly bouncing up and down?	Social Communication Questionnaire	SCQ
-0,102318495	SCQ_SCQ_17	17. Does she/he ever injure her/himself deliberately, such as by biting her/his arm or banging her/his head?	Social Communication Questionnaire	SCQ
0,102269868	right_Cortical_nucleus	Volume of the right Cortical nucleus	Amygdala Nuclei	NA
0,10217945	lh_LP	Volume of the left LP	Thalamic Nuclei	NA
-0,102116307	SCQ_SCQ_24	24. Does she/he nod her/his head to indicate yes?	Social Communication Questionnaire	SCQ
-0,102053095	CBCL_CBCL_56F	56F. Stomachaches	Child Behavior Checklist	CBCL
-0,101963231	PreInt_DevHx_preg_symp_12	Family stress	Interview-Developmental History	Dev.Hist.
-0,101850735	APQ_P_APQ_P_05	5. You reward or give something extra to your child for obeying you or behaving well	Alabama Parenting Questionnaire	APQ

Correlation coefficient (R)	Variable name in data	Item text	Instrument name	Instrument abbreviation (if applicable)
-0,101439385	SCARED_P_SCARED_P_12	12. When my child gets frightened, he/she feels like he/she is going crazy	Screen for Child Anxiety Related Disorders	SCARED
-0,101419744	Left_Lateral_Ventricle	Volume of the Left Lateral Ventricle	Subcortical Volume	NA
-0,101307747	SCARED_P_SCARED_P_30	30. My child is afraid of having anxiety (or panic) attacks	Screen for Child Anxiety Related Disorders	SCARED
-0,101172581	CBCL_CBCL_53	53. Overeating	Child Behavior Checklist	CBCL
-0,101045698	SympChck_CSC_09P	Hears, sees, or smells things that other people cannot (current)	Symptom Checklist Parent report	SympChck-P
-0,100630135	PreInt_EduHx_weakness_science	Science	Interview-Education and Social History	Edu/Soc.Hist.
-0,100437337	CBCL_CBCL_56C	56C. Nausea, feels sick	Child Behavior Checklist	CBCL
-0,100303456	APQ_P_APQ_P_31	31. The punishment you give your child depends on your mood	Alabama Parenting Questionnaire	APQ
0,100237766	lh_MDl	Volume of the left MDl	Thalamic Nuclei	NA
-0,099798986	NLES_P_NLES_P_Upset_Avg	Child's Average Upsetness/Negative Events	Negative Life Events Scale	NLES
0,099756593	left_presubiculum_head	Volume of the left presubiculum head	Hippocampus Subfields	NA
0,099457732	lh_rostralanteriorcingulate_thickness	Cortical thickness in lh rostralanteriorcingulate	Cortical Thickness	NA
0,099177267	rh_entorhinal_thickness	Cortical thickness in rh entorhinal	Cortical Thickness	NA
-0,099155904	PreInt_DevHx_temp_07	Overly sensitive to sound	Interview-Developmental History	Dev.Hist.
-0,099114126	SympChck_CSC_42P	Has skipped a part or a whole day of school (past	Symptom Checklist Parent report	SympChck-P
0,098962752	Barratt_Barratt_P1_Occ	Parent 1 level of occupation	Barratt Simplified Measure of Social Status	BSMSS
-0,098549082	SympChck_CSC_09C	Hears, sees, or smells things that	Symptom Checklist Parent report	SympChck-P

Correlation coefficient (R)	Variable name in data	Item text	Instrument name	Instrument abbreviation (if applicable)
		other people cannot (past		
-0,098470712	SympChck_CSC_14C	Worries about being separated from parent/guardian because of getting lost or kidnapped (current)	Symptom Checklist Parent report	SympChck-P
0,098416428	left_subiculum_head	Volume of the left subiculum head	Hippocampus Subfields	NA
-0,098340726	APQ_SR_APQ_SR_30	30. You come home from school more than an hour past the time your parents expect you to be home	Alabama Parenting Questionnaire	APQ
0,098171943	left_CA1_head	Volume of the left CA1 head	Hippocampus Subfields	NA
0,09789986	PreInt_EduHx_strength_math	Math	Interview- Education and Social History	Edu/Soc.Hist.
-0,09765005	APQ_SR_APQ_SR_38	38. Your parents hit you with a belt, switch, or other object when you have done something wrong	Alabama Parenting Questionnaire	APQ
0,097433408	rh_parahippocampal_volume	Cortical volume in rh parahippocampal	Cortical Volume	NA
-0,097302659	ASSQ_ASSQ_06	has a deviant style of communication with a formal, fussy, 'old-fashioned' or 'robotlike' language (i.e. talks differently than other children, in a formal or stilted way)	Autism Spectrum Screening Questionnaire	ASSQ
0,09718689	FGC_FGC_PU_Zone	Push-up fitness zone	FitnessGram Child	FGC
-0,096961238	SympChck_CSC_30P	Feels extremely worried about gaining weight or becoming fat (past	Symptom Checklist Parent report	SympChck-P
-0,096673148	APQ_P_APQ_P_30	30. Your child comes home from school more than an hour past	Alabama Parenting Questionnaire	APQ

Correlation coefficient (R)	Variable name in data	Item text	Instrument name	Instrument abbreviation (if applicable)
		the time you expect him/her to be home		
-0,096556685	CBCL_CBCL_49	49. Constipated, doesn't move bowels	Child Behavior Checklist	CBCL
0,096044434	APQ_SR_APQ_SR_02	2. Your parents tell you that you are doing a good job	Alabama Parenting Questionnaire	APQ
-0,096017673	APQ_SR_APQ_SR_39	39. Your parents yell or scream at you when you have done something wrong	Alabama Parenting Questionnaire	APQ
-0,095621631	NLES_P_NLES_P_16a	16a. A close family member to the child died such as a parent, close uncle, grandparent, or some other relative.	Negative Life Events Scale	NLES
-0,095113139	APQ_P_APQ_P_10	10. Your child stays out in the evening past the time that he/she is supposed to be home	Alabama Parenting Questionnaire	APQ
0,09500431	WIAT_WIAT_LCRV_Raw	Listening Comprehension Receptive Vocabulary Raw Score	Wechsler Individual Achievement Test	WIAT
-0,094594572	DTS_DTS_06	6. I can tolerate being distressed or upset as well as most people	Distress Tolerance Scale	DTS
-0,094350137	PreInt_EduHx_weakness_history	Social studies/history	Interview-Education and Social History	Edu/Soc.Hist.
-0,09423781	APQ_SR_APQ_SR_06	6. You fail to leave a note or let your parents know where you are going	Alabama Parenting Questionnaire	APQ
0,09411594	left_Whole_hippocampal_head	Volume of the left Whole hippocampal head	Hippocampus Subfields	NA
-0,094066494	NLES_P_NLES_P_10a	10a. The child's mother or father fought or argued with his/her relatives such as aunts, uncles, grandparents	Negative Life Events Scale	NLES

Correlation coefficient (R)	Variable name in data	Item text	Instrument name	Instrument abbreviation (if applicable)
0,093605775	lh_temporalpole_thickness	Cortical thickness in lh temporalpole	Cortical Thickness	NA
0,093561203	APQ_SR_APQ_SR_18	18. Your parents hug or kiss you when you have done something well	Alabama Parenting Questionnaire	APQ
-0,093373049	SCARED_P_SCARED_P_36	36. My child is scared to go to school	Screen for Child Anxiety Related Disorders	SCARED
-0,092922514	APQ_P_APQ_P_25	25. Your child is not punished when he/she has done something wrong	Alabama Parenting Questionnaire	APQ
0,091989783	left_molecular_layer_HP_head	Volume of the left molecular layer HP head	Hippocampus Subfields	NA
-0,091984779	APQ_SR_APQ_SR_10	10. You stay out in the evening past the time you are supposed to be home	Alabama Parenting Questionnaire	APQ
0,091976968	rh_cuneus_thickness	Cortical thickness in rh cuneus	Cortical Thickness	NA
-0,09145049	Physical_Systolic_BP	Systolic BP (mmHg)	Physical Measures	Physical
-0,091437361	SympChck_CSC_18P	Is afraid of being alone at home or in a different room than parent/guardian (past	Symptom Checklist Parent report	SympChck-P
-0,091271924	CBCL_CBCL_56A	56A. Aches or pains (not stomach or headaches)	Child Behavior Checklist	CBCL
0,091212566	lh_parsorbitalis_thickness	Cortical thickness in lh parsorbitalis	Cortical Thickness	NA
-0,090974882	left_GC_ML_DG_body	Volume of the left GC ML DG body	Hippocampus Subfields	NA
0,09088153	lh_entorhinal_thickness	Cortical thickness in lh entorhinal	Cortical Thickness	NA
0,090863409	CTOPP_CTOPP_NR_R	Nonword Repetition raw score	Comprehensive Test of Phonological Processing	CTOPP-2
0,090780299	PreInt_DevHx_temp_01	Easy to soothe when upset	Interview-Developmental History	Dev.Hist.
-0,090504237	SympChck_CSC_46C	Notices muscles moving uncontrollably (blinking a lot, shrugging) (current)	Symptom Checklist Parent report	SympChck-P

Correlation coefficient (R)	Variable name in data	Item text	Instrument name	Instrument abbreviation (if applicable)
0,090465637	lh_frontalpole_thickness	Cortical thickness in lh frontalpole	Cortical Thickness	NA
0,090427647	APQ_P_APQ_P_09	9. You ask your child about his/her day in school	Alabama Parenting Questionnaire	APQ
0,090392895	left_Central_nucleus	Volume of the left Central nucleus	Amygdala Nuclei	NA
-0,090195308	PreInt_FamHx_RDC_fmdk	Don't know father's mother's current age	Family History/Research Diagnostic Criteria	Fam.Med.Hist
-0,090002605	PreInt_FamHx_RDC_fmfdk	Don't know father's maternal grandfather's current age	Family History/Research Diagnostic Criteria	Fam.Med.Hist
-0,089753033	APQ_P_APQ_P_28	28. You don't check that your child comes home at the time he/she was supposed to	Alabama Parenting Questionnaire	APQ
0,089532576	lh_Whole_thalamus	Volume of the left Whole thalamus	Thalamic Nuclei	NA
0,089186436	APQ_SR_APQ_SR_Total	Other Discipline Practices Score (Not factored into total score but provides item level information)	Alabama Parenting Questionnaire	APQ
0,0890422	Left_Putamen	Volume of the Left Putamen	Subcortical Volume	NA
0,088625528	PreInt_DevHx_dev_normal	All developmental milestones within normal limits	Interview-Developmental History	Dev.Hist.
-0,088202823	right_molecular_layer_HP_body	Volume of the right molecular layer HP body	Hippocampus Subfields	NA
0,088011127	rh_L_Sg	Volume of the right L Sg	Thalamic Nuclei	NA
-0,087982658	CC_Anterior	Volume of the CC Anterior	Subcortical Volume	NA
-0,087719916	SCARED_P_SCARED_P_17	17. My child worries about going to school	Screen for Child Anxiety Related Disorders	SCARED
-0,087396781	SympChck_CSC_46P	Notices muscles moving uncontrollably (blinking a lot, shrugging) (past	Symptom Checklist Parent report	SympChck-P
-0,086793793	CBCL_CBCL_52	52. Feels too guilty	Child Behavior Checklist	CBCL
0,086770049	rightAnterior_inferior	Volume of the right Anterior inferior	Hypothalamic Subunits	NA

Correlation coefficient (R)	Variable name in data	Item text	Instrument name	Instrument abbreviation (if applicable)
-0,086544189	SCARED_P_SCARED_P_19	19. He/she child gets shaky	Screen for Child Anxiety Related Disorders	SCARED
-0,08642834	NLES_P_NLES_P_15a	15a. The child's mother or father lost a job	Negative Life Events Scale	NLES
-0,086150342	SympChck_CSC_29P	Often has accidents when he/she soild the bed or self during the day (past	Symptom Checklist Parent report	SympChck-P
-0,085945018	APQ_P_APQ_P_32	32. Your child is at home without adult supervision	Alabama Parenting Questionnaire	APQ
0,085915752	WIAT_WIAT_MP_Raw	Math Problem Solving Raw Score	Wechsler Individual Achievement Test	WIAT
0,085548084	lh_L_Sg	Volume of the left L Sg	Thalamic Nuclei	NA
-0,08533736	PreInt_DevHx_temp_02	Difficult to soothe when upset	Interview-Developmental History	Dev.Hist.
-0,084761748	SympChck_CSC_22C	Has intense fears of specific animals, situations, or anything else (current)	Symptom Checklist Parent report	SympChck-P
-0,084328689	SCARED_P_SCARED_P_02	2. My child gets headaches when he/she is at school	Screen for Child Anxiety Related Disorders	SCARED
-0,084243577	SCARED_P_SCARED_P_16	16. My child has nightmares about something bad happening to his/her parents	Screen for Child Anxiety Related Disorders	SCARED
0,084178899	lh_middletemporal_thickness	Cortical thickness in lh middletemporal	Cortical Thickness	NA
-0,083980175	SCQ_SCQ_02	2. Do you have a to and fro "conversation" with her/him that involves taking turns or building on what you have said?	Social Communication Questionnaire	SCQ
0,083547879	rh_temporalpole_thickness	Cortical thickness in rh temporalpole	Cortical Thickness	NA
-0,083208324	APQ_SR_APQ_SR_17	17. Your parents do not know the friends you are with	Alabama Parenting Questionnaire	APQ

Correlation coefficient (R)	Variable name in data	Item text	Instrument name	Instrument abbreviation (if applicable)
0,083192198	right_CA1_head	Volume of the right CA1 head	Hippocampus Subfields	NA
-0,083173781	PreInt_EduHx_tutor	Has your child had tutoring outside of school?	Interview-Education and Social History	Edu/Soc.Hist.
0,082015429	lh_parahippocampal_area	Cortical area in lh parahippocampal	Cortical Area	NA
0,082003823	APQ_P_APQ_P_18	18. You hug or kiss your child when he/she has done something well	Alabama Parenting Questionnaire	APQ
-0,081897615	SCARED_P_SCARED_P_11	11. My child gets stomachaches at school	Screen for Child Anxiety Related Disorders	SCARED
0,081189088	PreInt_DevHx_birthweight_lbs	Birth weight of child (lbs)	Interview-Developmental History	Dev.Hist.
-0,080969521	PreInt_EduHx_crafts	Drawing/painting/crafts	Interview-Education and Social History	Edu/Soc.Hist.
-0,080934103	rh_superiorfrontal_thickness	Cortical thickness in rh superiorfrontal	Cortical Thickness	NA
-0,080612978	NLES_P_NLES_P_20a	20a. The child's mother acted very worried, upset, or sad, not because of something the child did.	Negative Life Events Scale	NLES
-0,079945066	left_CA4_body	Volume of the left CA4 body	Hippocampus Subfields	NA
-0,079642604	PreInt_Demos_Fam_P1_Sex	Sex	Interview-Demographics/Family	Demog.Fam.
-0,079620063	APQ_SR_APQ_SR_28	28. You stay out later than you are supposed to and your parents don't know it	Alabama Parenting Questionnaire	APQ
0,079204529	rh_postcentral_area	Cortical area in rh postcentral	Cortical Area	NA
0,078448732	Left_Pallidum	Volume of the Left Pallidum	Subcortical Volume	NA
0,07819849	lh_parsopercularis_thickness	Cortical thickness in lh parsopercularis	Cortical Thickness	NA
0,078156286	lh_LD	Volume of the left LD	Thalamic Nuclei	NA
0,078138047	rh_rostralanteriorcingulate_thickness	Cortical thickness in rh rostralanteriorcingulate	Cortical Thickness	NA

Correlation coefficient (R)	Variable name in data	Item text	Instrument name	Instrument abbreviation (if applicable)
-0,077997096	PreInt_DevHx_temp_10	Slow to warm up	Interview-Developmental History	Dev.Hist.
0,077840111	FGC_FGC_TL_Zone	Trunk lift fitness zone	FitnessGram Child	FGC
-0,077747924	PreInt_FamHx_RDC_mmfdk	Don't know mother's paternal grandmother's current age	Family History/Research Diagnostic Criteria	Fam.Med.Hist
-0,077705985	APQ_P_APQ_P_34	34. You ignore your child when he/she is misbehvaing	Alabama Parenting Questionnaire	APQ
-0,076949911	SCARED_P_SCARED_P_04	4. My child gets scared if he/she sleeps away from home	Screen for Child Anxiety Related Disorders	SCARED
-0,07642891	rh_PuL	Volume of the right PuL	Thalamic Nuclei	NA
0,076337537	lh_PuI	Volume of the left PuI	Thalamic Nuclei	NA
0,076320401	APQ_P_APQ_P_02	2. You let your child know when he/she is doing a good job with something	Alabama Parenting Questionnaire	APQ
0,076164711	lh_bankssts_thickness	Cortical thickness in lh bankssts	Cortical Thickness	NA
-0,076090456	PreInt_FamHx_RDC_mfmdk	Don't know mother's maternal grandmother's current age	Family History/Research Diagnostic Criteria	Fam.Med.Hist
0,075700912	left_parasubiculum	Volume of the left parasubiculum	Hippocampus Subfields	NA
-0,075118143	APQ_P_APQ_P_Total	APQ Total Score	Alabama Parenting Questionnaire	APQ
0,075089487	APQ_SR_APQ_SR_41	41. Your parents use time out (make you sit or stand in a corner) as punishment	Alabama Parenting Questionnaire	APQ
-0,074938244	PreInt_FamHx_RDC_fffdk	Don't know father's paternal grandfather's current age	Family History/Research Diagnostic Criteria	Fam.Med.Hist
-0,074701823	PreInt_FamHx_RDC_fmmdk	Don't know father's maternal grandmother's current age	Family History/Research Diagnostic Criteria	Fam.Med.Hist
-0,074368799	Right_Lateral_Ventricle	Volume of the Right Lateral Ventricle	Subcortical Volume	NA
-0,074105295	rh_caudalmiddlefrontal_thickness	Cortical thickness in rh	Cortical Thickness	NA

Correlation coefficient (R)	Variable name in data	Item text	Instrument name	Instrument abbreviation (if applicable)
		caudalmiddlefrontal		
0,073898017	Basic_Demos_Study_Site	Study Site	Basic Demographic Information	Basic_Demos
0,073536896	lh_medialorbitofrontal_thickness	Cortical thickness in lh medialorbitofrontal	Cortical Thickness	NA
-0,073533839	SCARED_P_SCARED_P_20	20. My child has nightmares about something bad happening to him/her	Screen for Child Anxiety Related Disorders	SCARED
-0,072635456	SympChck_CSC_28P	Often has accidents when he/she wets the bed or self during the day (past	Symptom Checklist Parent report	SympChck-P
-0,072565414	SympChck_CSC_16P	Often does not want to go to school, due to worry about parent/guardian separation (past	Symptom Checklist Parent report	SympChck-P
-0,072228029	APQ_SR_APQ_SR_CP	Corporal Punishment Score	Alabama Parenting Questionnaire	APQ
-0,072218716	APQ_SR_APQ_SR_36	36. Your parents take away a privilege or money from you as punishment	Alabama Parenting Questionnaire	APQ
0,071947269	PreInt_DevHx_temp_09	Easily adaptable	Interview-Developmental History	Dev.Hist.
-0,07177497	lh_caudalmiddlefrontal_thickness	Cortical thickness in lh caudalmiddlefrontal	Cortical Thickness	NA
0,071700974	Left_Thalamus	Volume of the Left Thalamus	Subcortical Volume	NA
-0,071672796	PreInt_FamHx_RDC_ffmfdk	Don't know father's paternal grandmother's current age	Family History/Research Diagnostic Criteria	Fam.Med.Hist
0,071641746	rh_supramarginal_thickness	Cortical thickness in rh supramarginal	Cortical Thickness	NA
-0,071640112	rh_VM	Volume of the right VM	Thalamic Nuclei	NA
0,071609483	PreInt_EduHx_dance	Dance	Interview-Education and Social History	Edu/Soc.Hist.

Correlation coefficient (R)	Variable name in data	Item text	Instrument name	Instrument abbreviation (if applicable)
0,071417311	rh_MeanThickness_thickness	Cortical thickness in rh MeanThickness	Cortical Thickness	NA
0,071171309	Left_Hippocampus	Volume of the Left Hippocampus	Subcortical Volume	NA
-0,071137796	SRS_SRS_25	25. Doesn't seem to mind being out of step with or "not on the same wavelength" as others.	Social Responsiveness Scale	SRS
0,070583908	WIAT_WIAT_Word_Raw	Word Reading Raw Score	Wechsler Individual Achievement Test	WIAT
0,069793395	right_Corticoamygdaloid_transitio	Volume of the right Corticoamygdaloid transitio	Amygdala Nuclei	NA
-0,069575072	APQ_SR_APQ_SR_35	35. Your parents slap you when you have done something wrong	Alabama Parenting Questionnaire	APQ
-0,069543262	CBCL_CBCL_29	29. Fears certain animals, situations, or places, other than school	Child Behavior Checklist	CBCL
0,069350275	rh_medialorbitofrontal_thickness	Cortical thickness in rh medialorbitofrontal	Cortical Thickness	NA
0,069279273	rh_parahippocampal_area	Cortical area in rh parahippocampal	Cortical Area	NA
-0,069163972	Pegboard_peg_drops_nd	Non-dominant Hand - Number of drops	Grooved Pegboard	Pegboard
-0,068975233	SCARED_P_SCARED_P_28	28. People tell me that my child worries too much	Screen for Child Anxiety Related Disorders	SCARED
-0,068831748	right_CA3_body	Volume of the right CA3 body	Hippocampus Subfields	NA
-0,068594432	APQ_SR_APQ_SR_34	34. Your parents ignore you when you are misbehvaing	Alabama Parenting Questionnaire	APQ
0,068518735	APQ_P_APQ_P_11	11. You help your child with his/her homework	Alabama Parenting Questionnaire	APQ
0,066998887	rh_PuI	Volume of the right PuI	Thalamic Nuclei	NA
0,066916941	rh_LD	Volume of the right LD	Thalamic Nuclei	NA
0,066094132	lh_cuneus_thickness	Cortical thickness in lh cuneus	Cortical Thickness	NA

Correlation coefficient (R)	Variable name in data	Item text	Instrument name	Instrument abbreviation (if applicable)
-0,066020522	CBCL_CBCL_47	47. Nightmares	Child Behavior Checklist	CBCL
0,065462345	rh_PuM	Volume of the right PuM	Thalamic Nuclei	NA
-0,065379321	NLES_P_NLES_P_11a	11a. The child's mother or father acted badly in front of the child's friends (did things like yelled at them or criticized them).	Negative Life Events Scale	NLES
0,065370154	APQ_SR_APQ_SR_13	13. Your parents compliment you when you have done something well	Alabama Parenting Questionnaire	APQ
0,065309383	rh_Whole_thalamus	Volume of the right Whole thalamus	Thalamic Nuclei	NA
0,065288904	right_Central_nucleus	Volume of the right Central nucleus	Amygdala Nuclei	NA
-0,064734963	left_Whole_hippocampal_body	Volume of the left Whole hippocampal body	Hippocampus Subfields	NA
-0,064731434	PreInt_EduHx_school_difficulty	Did your child experience any difficulty starting school?	Interview-Education and Social History	Edu/Soc.Hist.
-0,064380346	SympChck_CSC_28C	Often has accidents when he/she wets the bed or self during the day (current)	Symptom Checklist Parent report	SympChck-P
0,064358448	lh_transversetemporal_thickness	Cortical thickness in lh transversetemporal	Cortical Thickness	NA
0,063953887	APQ_SR_APQ_SR_15	15. Your mom drives you to a special activity	Alabama Parenting Questionnaire	APQ
0,063373046	rh_bankssts_thickness	Cortical thickness in rh bankssts	Cortical Thickness	NA
-0,063353586	APQ_SR_APQ_SR_29	29. Your parents leave the house and don't tell you where they are going	Alabama Parenting Questionnaire	APQ
0,063250824	rh_transversetemporal_thickness	Cortical thickness in rh transversetemporal	Cortical Thickness	NA

Correlation coefficient (R)	Variable name in data	Item text	Instrument name	Instrument abbreviation (if applicable)
-0,063209873	SympChck_CSC_17C	Often has trouble going to sleep without parent/guardian nearby (current)	Symptom Checklist Parent report	SympChck-P
0,063158506	rh_supramarginal_volume	Cortical volume in rh supramarginal	Cortical Volume	NA
-0,062511341	SympChck_CSC_22P	Has intense fears of specific animals, situations, or anything else (past	Symptom Checklist Parent report	SympChck-P
-0,06245581	lh_superiorfrontal_thickness	Cortical thickness in lh superiorfrontal	Cortical Thickness	NA
0,062357145	lh_MeanThickness_thickness	Cortical thickness in lh MeanThickness	Cortical Thickness	NA
-0,061940659	PCIAT_PCIAT_07	7. How often does your child check his or her e-mail before doing something else?	Parent-Child Internet Addiction Test	PCIAT
-0,06153254	Age2	Age squared	Demographics	Demographics
0,061514752	APQ_SR_APQ_SR_04	4. Your mom helps you with some of your special acivities (such as sports, boy/girl scouts, church youth groups)	Alabama Parenting Questionnaire	APQ
-0,061112108	CBCL_CBCL_92	92. Talks or walks in sleep	Child Behavior Checklist	CBCL
0,06038284	lh_lingual_thickness	Cortical thickness in lh lingual	Cortical Thickness	NA
0,060282925	rightTubularSuperior	Volume of the right TubularSuperior	Hypothalamic Subunits	NA
-0,060098445	right_Hippocampal_tail	Volume of the right Hippocampal tail	Hippocampus Subfields	NA
-0,060049135	PreInt_Demos_Home_living_06	Child lives with Grandparents	Interview-Demographic and Household Information	Demog.Household.
0,060021541	right_Whole_hippocampal_head	Volume of the right Whole hippocampal head	Hippocampus Subfields	NA
-0,059540616	CBCL_CBCL_100	100. Trouble sleeping	Child Behavior Checklist	CBCL

Correlation coefficient (R)	Variable name in data	Item text	Instrument name	Instrument abbreviation (if applicable)
-0,059183685	right_presubiculum_body	Volume of the right presubiculum body	Hippocampus Subfields	NA
-0,059160381	PreInt_DevHx_temp_03	Colic	Interview-Developmental History	Dev.Hist.
0,058334111	rh_lingual_thickness	Cortical thickness in rh lingual	Cortical Thickness	NA
0,058314123	APQ_SR_APQ_SR_14A	14A. How about your dad?	Alabama Parenting Questionnaire	APQ
-0,058253349	ASSQ_ASSQ_01	is old-fashioned or precocious	Autism Spectrum Screening Questionnaire	ASSQ
-0,058046592	APQ_SR_APQ_SR_32	32. You are at home without an adult being with you	Alabama Parenting Questionnaire	APQ
0,057834067	right_molecular_layer_HP_head	Volume of the right molecular layer HP head	Hippocampus Subfields	NA
-0,05768797	CBCL_CBCL_55	55. Overweight	Child Behavior Checklist	CBCL
0,057686667	rh_Pt	Volume of the right Pt	Thalamic Nuclei	NA
-0,057666137	CBCL_CBCL_56E	56E. Rashes or other skin problems	Child Behavior Checklist	CBCL
0,057663618	APQ_SR_APQ_SR_27	27. Your parents tell you that they like it when you help out around the house	Alabama Parenting Questionnaire	APQ
-0,057607167	Left_choroid_plexus	Volume of the Left choroid plexus	Subcortical Volume	NA
0,057583976	left_fimbria	Volume of the left fimbria	Hippocampus Subfields	NA
0,057309052	Optic_Chiasm	Volume of the Optic Chiasm	Subcortical Volume	NA
0,056953218	APQ_P_APQ_P_PP	Positive Parenting Score	Alabama Parenting Questionnaire	APQ
0,056776999	Left_vessel	Volume of the Left vessel	Subcortical Volume	NA
0,056644213	APQ_SR_APQ_SR_PP	Positive Parenting Score	Alabama Parenting Questionnaire	APQ
-0,05663051	PreInt_DevHx_growth_concerns	Have there been any concerns about your child's growth?	Interview-Developmental History	Dev.Hist.
-0,056079322	x4th_Ventricle	Volume of the x4th Ventricle	Subcortical Volume	NA

Correlation coefficient (R)	Variable name in data	Item text	Instrument name	Instrument abbreviation (if applicable)
-0,055415571	rh_paracentral_thickness	Cortical thickness in rh paracentral	Cortical Thickness	NA
-0,055214669	left_Hippocampal_tail	Volume of the left Hippocampal tail	Hippocampus Subfields	NA
0,055115369	rh_superiorparietal_area	Cortical area in rh superiorparietal	Cortical Area	NA
-0,055018939	APQ_SR_APQ_SR_12	12. Your parents give up trying to get you to obey them because it's too much trouble	Alabama Parenting Questionnaire	APQ
0,054394627	rh_supramarginal_area	Cortical area in rh supramarginal	Cortical Area	NA
-0,054049494	Physical_Diastolic_BP	Diastolic BP (mmHg)	Physical Measures	Physical
0,05376372	rh_parsorbitalis_thickness	Cortical thickness in rh parsorbitalis	Cortical Thickness	NA
-0,05351425	APQ_P_APQ_P_21	21. Your child goes out after dark without an adult	Alabama Parenting Questionnaire	APQ
-0,053228845	CBCL_CBCL_56G	56G. Vomiting, throwing up	Child Behavior Checklist	CBCL
-0,053193917	APQ_SR_APQ_SR_24	24. Your parents get so busy that they forget where you are and what you are doing	Alabama Parenting Questionnaire	APQ
-0,053052277	PreInt_FamHx_RDC_mfdk	Don't know mother's mother's current age	Family History/Research Diagnostic Criteria	Fam.Med.Hist
-0,053026551	SCQ_SCQ_10	10. Does she/he ever use your hand like a tool or as if it were part of her/his own body (e.g., pointing with your finder or putting your hand on a doorknob to get you to open the door?)	Social Communication Questionnaire	SCQ
0,052846505	right_parasubiculum	Volume of the right parasubiculum	Hippocampus Subfields	NA
0,052647315	rh_inferiorparietal_thickness	Cortical thickness in rh inferiorparietal	Cortical Thickness	NA
-0,052440599	CBCL_CBCL_32	32. Feels he/she has to be perfect	Child Behavior Checklist	CBCL
-0,052330334	SympChck_CSC_17P	Often has trouble going to sleep without	Symptom Checklist Parent report	SympChck-P

Correlation coefficient (R)	Variable name in data	Item text	Instrument name	Instrument abbreviation (if applicable)
		parent/guardian nearby (past		
-0,051569114	PreInt_EduHx_dancing	Dancing	Interview-Education and Social History	Edu/Soc.Hist.
0,051014091	lh_precuneus_thickness	Cortical thickness in lh precuneus	Cortical Thickness	NA
-0,050702408	PreInt_DevHx_delivery	Delivery:	Interview-Developmental History	Dev.Hist.
0,050653563	PreInt_EduHx_afterschool_other	Other	Interview-Education and Social History	Edu/Soc.Hist.
0,04878864	rh_superiorparietal_volume	Cortical volume in rh superiorparietal	Cortical Volume	NA
0,048308965	rh_CM	Volume of the right CM	Thalamic Nuclei	NA
-0,048305882	PCIAT_PCIAT_12	12. How often does your child receive strange phone calls from new "online" friends?	Parent-Child Internet Addiction Test	PCIAT
-0,047650879	left_molecular_layer_HP_body	Volume of the left molecular layer HP body	Hippocampus Subfields	NA
0,047373605	rh_VA	Volume of the right VA	Thalamic Nuclei	NA
0,047310389	lh_CL	Volume of the left CL	Thalamic Nuclei	NA
0,04694884	left_GC_ML_DG_head	Volume of the left GC ML DG head	Hippocampus Subfields	NA
0,046841821	wholeRight	Volume of the wholeRight	Hypothalamic Subunits	NA
-0,046814337	Pegboard_peg_drops_d	Dominant Hand - Number of drops	Grooved Pegboard	Pegboard
-0,046682621	lh_VM	Volume of the left VM	Thalamic Nuclei	NA
0,046639504	lh_PuA	Volume of the left PuA	Thalamic Nuclei	NA
0,046410295	APQ_SR_APQ_SR_11	11. Your mom helps you with your homework	Alabama Parenting Questionnaire	APQ
0,046314203	rh_lateraloccipital_thickness	Cortical thickness in rh lateraloccipital	Cortical Thickness	NA
0,046167568	leftAnterior_inferior	Volume of the left Anterior inferior	Hypothalamic Subunits	NA
0,04616357	rh_frontalpole_thickness	Cortical thickness in rh frontalpole	Cortical Thickness	NA
-0,045317246	APQ_SR_APQ_SR_08	8. You talk your parents out of punishing you after you have	Alabama Parenting Questionnaire	APQ

Correlation coefficient (R)	Variable name in data	Item text	Instrument name	Instrument abbreviation (if applicable)
		done something wrong		
0,045213501	lh_lateraloccipital_thickness	Cortical thickness in lh lateraloccipital	Cortical Thickness	NA
-0,045058146	APQ_SR_APQ_SR_33	33. Your parents spank you with their hand when you have done something wrong	Alabama Parenting Questionnaire	APQ
0,04499758	rh_precuneus_thickness	Cortical thickness in rh precuneus	Cortical Thickness	NA
-0,044777467	APQ_SR_APQ_SR_OPD	APQ Total Score	Alabama Parenting Questionnaire	APQ
0,044774521	EstimatedTotalIntraCranialVol	Estimated Intracranial Volume	eTIV	NA
0,044644412	lh_Pt	Volume of the left Pt	Thalamic Nuclei	NA
0,044556106	lh_VA	Volume of the left VA	Thalamic Nuclei	NA
0,044436762	lh_CM	Volume of the left CM	Thalamic Nuclei	NA
0,044033577	right_subiculum_head	Volume of the right subiculum head	Hippocampus Subfields	NA
-0,043084726	SCQ_SCQ_22	22. Does she/he ever spontaneously point at things around her/him just to show you things (not because she/he wants them)?	Social Communication Questionnaire	SCQ
0,043032415	right_presubiculum_head	Volume of the right presubiculum head	Hippocampus Subfields	NA
0,042679218	Right_Thalamus	Volume of the Right Thalamus	Subcortical Volume	NA
-0,042607467	NLES_P_NLES_P_12a	12a. The child's mother or father was intoxicated in the child's presence	Negative Life Events Scale	NLES
0,042251	lh_inferiorparietal_thickness	Cortical thickness in lh inferiorparietal	Cortical Thickness	NA
0,041419954	APQ_SR_APQ_SR_40	40. Your parents calmly explain to you why your behavior was wrong when you misbehave	Alabama Parenting Questionnaire	APQ

Correlation coefficient (R)	Variable name in data	Item text	Instrument name	Instrument abbreviation (if applicable)
0,041109908	APQ_SR_APQ_SR_20	20. Your mom talks to you about your friends	Alabama Parenting Questionnaire	APQ
0,040916626	left_CA4_head	Volume of the left CA4 head	Hippocampus Subfields	NA
0,040344476	rh_posteriorcingulate_thickness	Cortical thickness in rh posteriorcingulate	Cortical Thickness	NA
0,039965647	lh_postcentral_thickness	Cortical thickness in lh postcentral	Cortical Thickness	NA
0,039278408	PreInt_Demos_Home_fam_01_age	Person 1- age	Interview-Demographic and Household Information	Demog.Household.
-0,039145757	ASSQ_ASSQ_02	is regarded as an 'eccentric professor' by the other children	Autism Spectrum Screening Questionnaire	ASSQ
0,038866042	PreInt_EduHx_martial_arts	Martial Arts	Interview-Education and Social History	Edu/Soc.Hist.
-0,038793536	PreInt_FamHx_RDC_mmmdk	Don't know mother's maternal grandmother's current age	Family History/Research Diagnostic Criteria	Fam.Med.Hist
-0,038303242	Basic_Demos_Sex	Sex	Basic Demographic Information	Basic_Demos
-0,037680335	lh_caudalanteriorcingulate_thickness	Cortical thickness in lh caudalanteriorcingulate	Cortical Thickness	NA
-0,037379913	PreInt_DevHx_lost_skills	Has your child lost any skills or abilities?	Interview-Developmental History	Dev.Hist.
0,037364032	rh_PuA	Volume of the right PuA	Thalamic Nuclei	NA
0,037311701	APQ_P_APQ_P_16	16. You praise your child for behaving well	Alabama Parenting Questionnaire	APQ
0,0372796	leftAnterior_superior	Volume of the left Anterior superior	Hypothalamic Subunits	NA
-0,037178292	lh_CeM	Volume of the left CeM	Thalamic Nuclei	NA
-0,0368767	APQ_SR_APQ_SR_ID	Inconsistent Discipline Score	Alabama Parenting Questionnaire	APQ
0,036759911	FGC_FGC_SRL_Zone	Sit & Reach fitness zone (left side)	FitnessGram Child	FGC
0,036338948	FGC_FGC_SRR_Zone	Sit & Reach fitness zone (right side)	FitnessGram Child	FGC
-0,036298723	CBCL_CBCL_56D	56D.A. Problems with eyes (not if	Child Behavior Checklist	CBCL

Correlation coefficient (R)	Variable name in data	Item text	Instrument name	Instrument abbreviation (if applicable)
		corrected by glasses		
0,035949661	PreInt_EduHx_weakness_other	Other	Interview-Education and Social History	Edu/Soc.Hist.
0,035772747	lh_parstriangularis_thickness	Cortical thickness in lh parstriangularis	Cortical Thickness	NA
-0,035723835	SympChck_CSC_18C	Is afraid of being alone at home or in a different room than parent/guardian (current)	Symptom Checklist Parent report	SympChck-P
-0,035236919	APQ_SR_APQ_SR_42	42. Your parents give you extra chores as punishment	Alabama Parenting Questionnaire	APQ
0,035164959	lh_VLp	Volume of the left VLp	Thalamic Nuclei	NA
0,035148149	PreInt_EduHx_strength_science	Science	Interview-Education and Social History	Edu/Soc.Hist.
0,03507636	APQ_SR_APQ_SR_14	14. Your mom asks you what your plans are for the coming day	Alabama Parenting Questionnaire	APQ
0,035023374	Right_Hippocampus	Volume of the Right Hippocampus	Subcortical Volume	NA
0,034851695	right_GC_ML_DG_head	Volume of the right GC ML DG head	Hippocampus Subfields	NA
-0,034216046	PreInt_DevHx_complications	Compications at birth	Interview-Developmental History	Dev.Hist.
0,034168524	Right_Pallidum	Volume of the Right Pallidum	Subcortical Volume	NA
0,034129514	lh_VPL	Volume of the left VPL	Thalamic Nuclei	NA
-0,033551816	NLES_P_NLES_P_19a	19a. The child's father acted very worried, upset, or sad, not because of something the child did.	Negative Life Events Scale	NLES
0,033211667	APQ_SR_APQ_SR_26	26. Your mom goes to a meeting at school, like a PTA meeting, or a parent/teacher conference	Alabama Parenting Questionnaire	APQ
0,033083204	Physical_HeartRate	Heart rate (beats/min)	Physical Measures	Physical

Correlation coefficient (R)	Variable name in data	Item text	Instrument name	Instrument abbreviation (if applicable)
0,032735187	rh_caudalanteriorcingulate_thickness	Cortical thickness in rh caudalanteriorcingulate	Cortical Thickness	NA
-0,032288542	lh_pericalcarine_thickness	Cortical thickness in lh pericalcarine	Cortical Thickness	NA
0,032283299	rightAnterior_superior	Volume of the right Anterior superior	Hypothalamic Subunits	NA
-0,031999718	SCQ_SCQ_23	23. Does she/he ever use gestures, other than pointing or pulling your hand, to let you know what she/he wants?	Social Communication Questionnaire	SCQ
-0,031910744	left_CA3_body	Volume of the left CA3 body	Hippocampus Subfields	NA
-0,031851452	PreInt_Demos_Home_living_17	Child lives with Other	Interview-Demographic and Household Information	Demog.Household.
0,031671571	Left_Cerebellum_White_Matter	Volume of the Left Cerebellum White Matter	Subcortical Volume	NA
0,031501885	lh_VLa	Volume of the left VLa	Thalamic Nuclei	NA
-0,031498575	PreInt_DevHx_newborn_problems	Newborn period:	Interview-Developmental History	Dev.Hist.
-0,031026917	CSF	Volume of the CSF	Subcortical Volume	NA
0,029779597	lh_supramarginal_thickness	Cortical thickness in lh supramarginal	Cortical Thickness	NA
-0,029637421	FGC_FGC_SRR	Sit & Reach total (right side)	FitnessGram Child	FGC
-0,029468393	Medulla	Volume of the Medulla	Brainstem	NA
0,029260693	rh_parsopercularis_thickness	Cortical thickness in rh parsopercularis	Cortical Thickness	NA
-0,029251093	lh_precentral_thickness	Cortical thickness in lh precentral	Cortical Thickness	NA
0,028643249	APQ_P_APQ_P_26	26. you attend PTA meetings, parent/teacher conderences, or other meetings at your child's school	Alabama Parenting Questionnaire	APQ
0,02837054	WIAT_WIAT_Num_Raw	Numerical Operations Raw Score	Wechsler Individual Achievement Test	WIAT

Correlation coefficient (R)	Variable name in data	Item text	Instrument name	Instrument abbreviation (if applicable)
-0,028300227	PreInt_EduHx_homework	Homework	Interview-Education and Social History	Edu/Soc.Hist.
0,028295069	PreInt_DevHx_preg_symp_07	Took any prescription	Interview-Developmental History	Dev.Hist.
0,028245508	WIAT_WIAT_Spell_Raw	Spelling Raw Score	Wechsler Individual Achievement Test	WIAT
-0,028068335	rh_Pf	Volume of the right Pf	Thalamic Nuclei	NA
0,027973765	lh_posteriorcingulate_thickness	Cortical thickness in lh posteriorcingulate	Cortical Thickness	NA
-0,027502123	PreInt_EduHx_current_religious	Is he/she religious now?	Interview-Education and Social History	Edu/Soc.Hist.
-0,027401572	APQ_SR_APQ_SR_22	22. Your parents let you out of a punishment early (like lift restrictions earlier than they originally said)	Alabama Parenting Questionnaire	APQ
0,027266929	rh_AV	Volume of the right AV	Thalamic Nuclei	NA
-0,026398851	PreInt_DevHx_preg_symp_01	Spotting or vaginal bleeding	Interview-Developmental History	Dev.Hist.
-0,026334369	SympChck_CSC_31P	Is underweight for his/her age and height (past	Symptom Checklist Parent report	SympChck-P
0,025991167	PreInt_EduHx_strength_history	Social studies/history	Interview-Education and Social History	Edu/Soc.Hist.
0,025985796	rh_parstriangularis_thickness	Cortical thickness in rh parstriangularis	Cortical Thickness	NA
0,025687648	PreInt_Demos_Fam_P1_Age	Age	Interview-Demographics/Family	Demog.Fam.
0,025280178	ColorVision_CV_Plate_09_R	Plate 09 Result	Ishihara Color Vision Test	ColorVision
-0,025232768	right_Whole_hippocampus	Volume of the right Whole hippocampus	Hippocampus Subfields	NA
0,024720512	lh_VAmc	Volume of the left VAmc	Thalamic Nuclei	NA
-0,024648106	lh_PuL	Volume of the left PuL	Thalamic Nuclei	NA
-0,024549313	rh_CeM	Volume of the right CeM	Thalamic Nuclei	NA
-0,024044867	lh_Pf	Volume of the left Pf	Thalamic Nuclei	NA

Correlation coefficient (R)	Variable name in data	Item text	Instrument name	Instrument abbreviation (if applicable)
0,023985333	right_HATA	Volume of the right HATA	Hippocampus Subfields	NA
-0,023806774	x3rd_Ventricle	Volume of the x3rd Ventricle	Subcortical Volume	NA
0,023562863	right_CA4_head	Volume of the right CA4 head	Hippocampus Subfields	NA
0,023417227	lh_superiorparietal_thickness	Cortical thickness in lh superiorparietal	Cortical Thickness	NA
0,023176573	Right_Inf_Lat_Vent	Volume of the Right Inf Lat Vent	Subcortical Volume	NA
-0,022972444	PreInt_DevHx_temp_04	Eating difficulties	Interview-Developmental History	Dev.Hist.
0,022822617	rh_superiorparietal_thickness	Cortical thickness in rh superiorparietal	Cortical Thickness	NA
-0,022313264	PreInt_DevHx_preg_symp_04	Diabetes	Interview-Developmental History	Dev.Hist.
0,022076463	Midbrain	Volume of the Midbrain	Brainstem	NA
-0,021315896	FGC_FGC_CU	Curl up total	FitnessGram Child	FGC
0,021217622	rh_VLa	Volume of the right VLa	Thalamic Nuclei	NA
0,020809994	rh_isthmuscingulate_thickness	Cortical thickness in rh isthmuscingulate	Cortical Thickness	NA
0,020260575	rightPosterior	Volume of the right Posterior	Hypothalamic Subunits	NA
-0,020192075	PreInt_DevHx_preg_dur	Duration of pregnancy (weeks)	Interview-Developmental History	Dev.Hist.
-0,020075271	ColorVision_CV_Score	Color Vision Score	Ishihara Color Vision Test	ColorVision
-0,019895094	rh_pericalcarine_thickness	Cortical thickness in rh pericalcarine	Cortical Thickness	NA
0,019657975	Left_Inf_Lat_Vent	Volume of the Left Inf Lat Vent	Subcortical Volume	NA
0,019602726	NLES_P_NLES_P_02a	2a. The child's close friend had serious troubles, problems, illness, or injury	Negative Life Events Scale	NLES
0,019250512	rh_VLp	Volume of the right VLp	Thalamic Nuclei	NA
-0,019108787	PreInt_DevHx_temp_05	Sleeping difficulties	Interview-Developmental History	Dev.Hist.
0,01883224	wholeLeft	Volume of the wholeLeft	Hypothalamic Subunits	NA
-0,018484058	leftTubularInferior	Volume of the left TubularInferior	Hypothalamic Subunits	NA
0,017070596	rh_MV_Re_	Volume of the right MV Re	Thalamic Nuclei	NA

Correlation coefficient (R)	Variable name in data	Item text	Instrument name	Instrument abbreviation (if applicable)
-0,017006265	right_CA3_head	Volume of the right CA3 head	Hippocampus Subfields	NA
0,016426191	rightTubularInferior	Volume of the right TubularInferior	Hypothalamic Subunits	NA
-0,015767033	PreInt_DevHx_temp_08	Baby was "limp" or stiff	Interview-Developmental History	Dev.Hist.
-0,015658224	leftTubularSuperior	Volume of the left TubularSuperior	Hypothalamic Subunits	NA
0,015557712	APQ_SR_APQ_SR_09	9. Your mom asks you about your day in school	Alabama Parenting Questionnaire	APQ
0,015545976	APQ_P_APQ_P_19	19. Your child goed out with a set time to be home	Alabama Parenting Questionnaire	APQ
-0,015366389	PreInt_FamHx_RDC_omoves1	Number of times changed address (age 0-5):	Family History/Research Diagnostic Criteria	Fam.Med.Hist
-0,015272282	lh_paracentral_thickness	Cortical thickness in lh paracentral	Cortical Thickness	NA
-0,014729216	APQ_SR_APQ_SR_19	19. You go out with a set time to be home	Alabama Parenting Questionnaire	APQ
0,014339987	lh_MV_Re_	Volume of the left MV Re	Thalamic Nuclei	NA
-0,01426056	rh_VPL	Volume of the right VPL	Thalamic Nuclei	NA
-0,014035148	PreInt_DevHx_preg_symp_05	High blood pressure	Interview-Developmental History	Dev.Hist.
-0,013781971	CBCL_CBCL_108	108. Wets the bed	Child Behavior Checklist	CBCL
0,013362448	left_Whole_hippocampus	Volume of the left Whole hippocampus	Hippocampus Subfields	NA
-0,012796816	right_CA1_body	Volume of the right CA1 body	Hippocampus Subfields	NA
-0,011975303	SCARED_P_SCARED_P_34	34. When my child gets frightened, he/she feels like throwing up	Screen for Child Anxiety Related Disorders	SCARED
0,011880102	APQ_SR_APQ_SR_25	25. Your parents do not punish you when you have done something wrong	Alabama Parenting Questionnaire	APQ
0,011656803	rh_CL	Volume of the right CL	Thalamic Nuclei	NA
-0,011496918	SympChck_CSC_31C	Is underweight for his/her age	Symptom Checklist Parent report	SympChck-P

Correlation coefficient (R)	Variable name in data	Item text	Instrument name	Instrument abbreviation (if applicable)
		and height (current)		
-0,011442523	lh_rostralmiddlefrontal_thickness	Cortical thickness in lh rostralmiddlefrontal	Cortical Thickness	NA
0,011287002	APQ_P_APQ_P_13	13. You compliment your child when he/she has done something well	Alabama Parenting Questionnaire	APQ
0,011067776	EHQ_EHQ_08	Using a Broom (upper hand)	Edinburgh Handedness Questionnaire	EHQ
-0,010799964	SCP	Volume of the SCP	Brainstem	NA
0,010072777	Pons	Volume of the Pons	Brainstem	NA
-0,00990071	APQ_P_APQ_P_41	41. You use time out him/her sit or stand in a corner) as punishment	Alabama Parenting Questionnaire	APQ
0,009886455	SCQ_SCQ_21	21. Does she/he ever spontaneously copy you (or other people) or what you are doing (such as vacuuming, gardening, or mending things)?	Social Communication Questionnaire	SCQ
0,009615722	EHQ_EHQ_11	Holding a Computer Mouse	Edinburgh Handedness Questionnaire	EHQ
0,009517752	left_CA1_body	Volume of the left CA1 body	Hippocampus Subfields	NA
-0,008894653	ColorVision_CV_Plate_04_R	Plate 04 Result	Ishihara Color Vision Test	ColorVision
0,008811793	PreInt_EduHx_videocomputergames	Playing video/computer games	Interview-Education and Social History	Edu/Soc.Hist.
-0,008598124	Left_VentralDC	Volume of the Left VentralDC	Subcortical Volume	NA
-0,008463696	CC_Mid_Anterior	Volume of the CC Mid Anterior	Subcortical Volume	NA
-0,008409542	PreInt_DevHx_preg_symp_11	Swollen ankles	Interview-Developmental History	Dev.Hist.
-0,008231638	rh_rostralmiddlefrontal_thickness	Cortical thickness in rh rostralmiddlefrontal	Cortical Thickness	NA
0,00815582	CC_Central	Volume of the CC Central	Subcortical Volume	NA

Correlation coefficient (R)	Variable name in data	Item text	Instrument name	Instrument abbreviation (if applicable)
-0,008057862	CBCL_CBCL_56H	56H.A. Other	Child Behavior Checklist	CBCL
-0,007249769	lh_isthmuscingulate_thickness	Cortical thickness in lh isthmuscingulate	Cortical Thickness	NA
0,007106576	Whole_brainstem	Volume of the Whole_brainstem	Brainstem	NA
-0,00661672	PreInt_EduHx_strength_other	Other	Interview-Education and Social History	Edu/Soc.Hist.
-0,005881607	lh_Pc	Volume of the left Pc	Thalamic Nuclei	NA
-0,005818474	APQ_SR_APQ_SR_05	5. Your parents reward or give something extra to you for behaving well	Alabama Parenting Questionnaire	APQ
0,0057461	APQ_SR_APQ_SR_16	16. Your parents praise you for behaving well	Alabama Parenting Questionnaire	APQ
0,005669862	PreInt_EduHx_family_religious	Was your child raised in a particular religious faith?	Interview-Education and Social History	Edu/Soc.Hist.
0,005262512	rh_VAmc	Volume of the right VAmc	Thalamic Nuclei	NA
-0,004950771	left_presubiculum_body	Volume of the left presubiculum body	Hippocampus Subfields	NA
-0,004949217	left_HATA	Volume of the left HATA	Hippocampus Subfields	NA
0,004927065	FGC_FGC_TL	Trunk lift total	FitnessGram Child	FGC
-0,004785096	rh_Pc	Volume of the right Pc	Thalamic Nuclei	NA
0,004432254	lh_AV	Volume of the left AV	Thalamic Nuclei	NA
-0,004335434	Right_Cerebellum_White_Matter	Volume of the Right Cerebellum White Matter	Subcortical Volume	NA
-0,003904115	APQ_SR_APQ_SR_03	3. Your parents threaten to punish you and then not do it	Alabama Parenting Questionnaire	APQ
0,003285421	Brain_Stem	Volume of the Brain Stem	Subcortical Volume	NA
-0,003118953	NLES_P_NLES_P_03a	3a. The child suffered from a serious physical illness, injury, or extreme pain (something that required rest of one week in bed, hospitalization, or surgery).	Negative Life Events Scale	NLES

Correlation coefficient (R)	Variable name in data	Item text	Instrument name	Instrument abbreviation (if applicable)
-0,002627156	APQ_SR_APQ_SR_31	31. The punishment your parents give depends on their mood	Alabama Parenting Questionnaire	APQ
-0,002238659	FGC_FGC_SRL	Sit & Reach total (left side)	FitnessGram Child	FGC
-0,001846757	APQ_SR_APQ_SR_37	37. Your parents send you to your room as punishment	Alabama Parenting Questionnaire	APQ
-0,001330838	NLES_P_NLES_P_18a	18a. A close friend of the child moved away	Negative Life Events Scale	NLES
0,001034299	left_CA3_head	Volume of the left CA3 head	Hippocampus Subfields	NA

Table S5. Linear models of associations of age, sex, and diagnosis with each mode. Diagnosis category included as a fixed factor

	**Mode1**	**Mode2**
Age	38.24 (.44)	-8.40 (.99)
	p = 0.00	p = 0.00
Age^2	-3.29 (.43)	1.88 (.97)
	p = 0.00	p = .06
Sex	-.01 (.02)	.14 (.05)
	p = .66	p = .01
ADHD	.01 (.04)	-.67 (.08)
	p = .81	p = 0.00
Anxiety	-.01 (.04)	-.23 (.09)
	p = .91	p = .02
Mood	.27 (.06)	-.74 (.14)
	p = 0.000	p = 0.000
Other	.02 (.06)	-.52 (.14)
	p = .73	p = .001
Other ND	.04 (.04)	-.56 (.09)
	p = .26	p = 0.00
Constant	.13 (.03)	-.10 (.07)
	p = .001	p = .18
Observations	1,727	1,727
Adjusted R^2	.83	.09
Residual Std. Error (df = 1718)	.43	.96

Note. Beta coefficient (standard error). ADHD; attention-deficit hyperactivity disorders. ND; neurodevelopmental disorders.

Table S6. Linear models of associations of each diagnosis with mode. Age, age^2, and sex are included as covariates

	Mode 1					Mode 2				
	ADHD	Anxiety	Mood	Other	Other ND	ADHD	Anxiety	Mood	Other	Other ND
Age	27.26 (.42) p = 0.00	20.30 (.43) p = 0.00	15.52 (.45) p = 0.00	15.98 (.39) p = 0.00	20.51 (.42) p = 0.00	-4.98 (.96) p = 0.000	-4.48 (.97) p = 0.000	-2.23 (1.13) p = .05	-4.31 (.99) p = 0.000	-5.69 (.95) p = 0.00
Age^2	-2.67 (.42) p = 0.00	-1.94 (.42) p = 0.000	-1.96 (.39) p = 0.000	-1.88 (.39) p = 0.000	-2.10 (.42) p = 0.000	2.25 (.96) p = .02	1.67 (.97) p = .09	2.07 (.99) p = .04	1.53 (1.00) p = .13	.84 (.96) p = .39
Sex	.01 (.03) p = .77	.01 (.04) p = .84	-.01 (.05) p = .88	-.02 (.05) p = .64	.02 (.04) p = .62	.18 (.07) p = .01	.19 (.09) p = .04	.20 (.13) p = .11	.29 (.13) p = .03	.18 (.08) p = .04
ADHD	.003 (.03) p = .93					-.68 (.08) p = 0.00				
Anxiety		-.004 (.04) p = .92					-.23 (.09) p = .02			
Mood			.32 (.07) p = 0.000					-.81 (.16) p = 0.000		
Other				.03 (.06) p = .63					-.55 (.14) p = .001	
Other ND					.04 (.04) p = .32					-.56 (.09) p = 0.00
Constant	.02 (.03) p = .51	.17 (.04) p = 0.000	.28 (.04) p = 0.00	.04 (.04) p = .31	.03 (.04) p = .42	-.11 (.08) p = .17	-.14 (.08) p = .09	-.15 (.10) p = .13	-.15 (.10) p = .12	-.10 (.08) p = .21
Observations	940	455	251	255	578	940	455	251	255	578
Adjusted R^2	.82	.84	.88	.87	.81	.10	.08	.18	.13	.12
Residual Std. Error	.42 (df = 935)	.42 (df = 450)	.39 (df = 246)	.39 (df = 250)	.42 (df = 573)	.96 (df = 935)	.96 (df = 450)	.98 (df = 246)	.99 (df = 250)	.95 (df = 573)

Note. Beta coefficient (standard error). ADHD; attention-deficit hyperactivity disorders. ND; neurodevelopmental disorders.

Table S7. Pairwise post hoc comparisons using emmeans and Tukey adjustment

Comparison	Beta	SE	df	LL	UL	t-value	corr p
**Mode 1**							
No dx - ADHD	-0.03	0.04	1719	-0.13	0.08	-0.73	0.978
No dx - Anxiety	-0.01	0.04	1719	-0.13	0.11	-0.28	1.000
No dx - Mood	-0.27	0.06	1719	-0.46	-0.09	-4.22	3.70 e-4
No dx - Other	0	0.06	1719	-0.17	0.18	0.06	1.00
No dx - Other ND	-0.06	0.04	1719	-0.17	0.05	-1.58	0.611
ADHD - Anxiety	0.01	0.03	1719	-0.08	0.1	0.46	0.997
ADHD - Mood	-0.25	0.06	1719	-0.41	-0.08	-4.2	4.07 e-4
ADHD - Other	0.03	0.06	1719	-0.13	0.19	0.54	0.994
ADHD - Other ND	-0.04	0.03	1719	-0.11	0.04	-1.3	0.787
Anxiety - Mood	-0.26	0.06	1719	-0.44	-0.09	-4.24	3.42 e-4
Anxiety - Other	0.02	0.06	1719	-0.15	0.18	0.26	1.00
Anxiety - Other ND	-0.05	0.03	1719	-0.15	0.05	-1.43	0.710
Mood - Other	0.28	0.08	1719	0.06	0.5	3.58	0.005
Mood - Other ND	0.21	0.06	1719	0.04	0.38	3.49	0.007
Other - Other ND	-0.06	0.06	1719	-0.23	0.1	-1.13	0.868
**Mode 2**							
No dx - ADHD	0.68	0.08	1719	0.46	0.91	8.64	3.70 e-12
No dx - Anxiety	0.24	0.09	1719	-0.02	0.51	2.66	0.084
No dx - Mood	0.74	0.14	1719	0.33	1.15	5.15	4.27 e-6
No dx - Other	0.51	0.14	1719	0.12	0.9	3.71	0.003
No dx - Other ND	0.57	0.09	1719	0.32	0.81	6.59	8.65 e-10
ADHD - Anxiety	-0.44	0.07	1719	-0.64	-0.24	-6.3	5.70 e-9
ADHD - Mood	0.05	0.13	1719	-0.32	0.43	0.42	1.000
ADHD - Other	-0.17	0.12	1719	-0.52	0.18	-1.42	0.713
ADHD - Other ND	-0.12	0.06	1719	-0.29	0.05	-1.96	0.365
Anxiety - Mood	0.49	0.14	1719	0.1	0.88	3.61	0.004
Anxiety - Other	0.26	0.13	1719	-0.11	0.64	2.01	0.338
Anxiety - Other ND	0.32	0.08	1719	0.1	0.54	4.16	4.71 e-4
Mood - Other	-0.23	0.17	1719	-0.72	0.26	-1.34	0.764
Mood - Other ND	-0.17	0.13	1719	-0.56	0.21	-1.28	0.794
Other - Other ND	0.06	0.13	1719	-0.31	0.42	0.45	0.998

Note. Dx; diagnosis ADHD; attention-deficit hyperactivity disorders. ND; neurodevelopmental disorders. SE; standard error. df; degrees of freedom. LL; lower confidence level (2.5%). UL; upper confidence level (97.5%). corr p; p-value adjusted with Tukey.

Table S8. Linear models of associations of age, sex, and number of diagnoses (0-10) with each mode. Number of diagnoses included as a continuous variable

	Mode1	Mode2
Age	38.48 (.43)	-6.60 (.96)
	p = 0.00	p = 0.00
Age2	-3.25 (.43)	2.23 (.95)
	p = 0.00	p = .02
Sex	-.02 (.02)	.09 (.05)
	p = .45	p = .06
No. of diagnoses (0-10)	.03 (.01)	-.28 (.02)
	p = .02	p = 0.00
Constant	.15 (.02)	-.57 (.04)
	p = 0.00	p = 0.00
Observations	1,727	1,727
Adjusted R^2	.83	.12
Residual Std. Error (df = 1722)	.43	.95

Note. Beta coefficient (standard error). ADHD; attention-deficit hyperactivity disorders. ND; neurodevelopmental disorders.

Table S9. Linear models of associations of age, sex, and number of diagnoses (1-10) with each mode. Number of diagnoses included as a continuous variable, excluding no diagnosis (0)

	Mode1	Mode2
Age	36.35 (.44)	-6.09 (.96)
	p = 0.00	p = 0.00
Age2	-2.77 (.43)	1.58 (.94)
	p = 0.00	p = .10
Sex	-.02 (.02)	.07 (.05)
	p = .32	p = .19
No. of diagnoses (1-10)	.03 (.01)	-.26 (.03)
	p = .02	p = 0.00
Constant	.17 (.02)	-.58 (.04)
	p = 0.00	p = 0.00
Observations	1,539	1,539
Adjusted R^2	.82	.10
Residual Std. Error (df = 1534)	.43	.94

Note. Beta coefficient (standard error). ADHD; attention-deficit hyperactivity disorders. ND; neurodevelopmental disorders.

Supplementary references

1. Leys, C., et al., *Detecting outliers: Do not use standard deviation around the mean, use absolute deviation around the median.* Journal of Experimental Social Psychology, 2013. **49**(4): p. 764-766.
2. Fischl, B., *FreeSurfer.* NeuroImage, 2012. **62**(2): p. 774-781.
3. Iglesias, J.E., et al., *A computational atlas of the hippocampal formation using ex vivo, ultra-high resolution MRI: Application to adaptive segmentation of in vivo MRI.* NeuroImage, 2015. **115**: p. 117-137.
4. Iglesias, J.E., et al., *A probabilistic atlas of the human thalamic nuclei combining ex vivo MRI and histology.* Neuroimage, 2018. **183**: p. 314-326.
5. Saygin, Z.M., et al., *High-resolution magnetic resonance imaging reveals nuclei of the human amygdala: manual segmentation to automatic atlas.* NeuroImage, 2017. **155**: p. 370-382.
6. Billot, B., et al., *Automated segmentation of the hypothalamus and associated subunits in brain MRI.* NeuroImage, 2020. **223**: p. 117287.
7. Iglesias, J.E., et al., *Bayesian segmentation of brainstem structures in MRI.* NeuroImage, 2015. **113**: p. 184-195.
8. Pulli, E.P., et al., *Feasibility of FreeSurfer Processing for T1-Weighted Brain Images of 5-Year-Olds: Semiautomated Protocol of FinnBrain Neuroimaging Lab.* Frontiers in Neuroscience, 2022. **16**.
9. Miller, K.L., et al., *Multimodal population brain imaging in the UK Biobank prospective epidemiological study.* Nat Neurosci, 2016. **19**(11): p. 1523-1536.
